# Supplementary material for: Identifying the core genome of the nucleus-forming bacteriophage family and characterization of Erwinia phage RAY
Source: Cell Rep. Author manuscript; Available in PMC 2023 Jun 27. (PMC10299810; doi:10.1016/j.celrep.2023.112432)
Supplement: 1 [file NIHMS1905355-supplement-1.pdf]

Cell Reports, Volume *42*

## Supplemental information

# Identifying the core genome of the nucleus-forming bacteriophage family and characterization of *Erwinia* phage RAY

**Amy Prichard, Jina Lee, Thomas G. Laughlin, Amber Lee, Kyle P. Thomas, Annika E. Sy, Tara Spencer, Aileen Asavavimol, Allison Cafferata, Mia Cameron, Nicholas Chiu, Demyan Davydov, Isha Desai, Gabriel Diaz, Melissa Guereca, Kiley Hearst, Leyi Huang, Emily Jacobs, Annika Johnson, Samuel Kahn, Ryan Koch, Adamari Martinez, Meliné Norquist, Tyler Pau, Gino Prasad, Katrina Saam, Milan Sandhu, Angel Jose Sarabia, Siena Schumaker, Aaron Sonin, Ariya Uyeno, Alison Zhao, Kevin D. Corbett, Kit Pogliano, Justin Meyer, Julianne H. Grose, Elizabeth Villa, Rachel Dutton, and Joe Pogliano**

**Table S1. List of chimallin-encoding phage used in our analyses, Related to Figures 1 and 2.** These phage were used for our determination of the Chimalliviridae core genome.

| Host | Phage Name | Host | Phage Name |
|---|---|---|---|
| Aeromonas | PS1 | Erwinia | pEa_SNUABM_37 |
| Aeromonas | pAEv1810 | Erwinia | AH06 |
| Aeromonas | CF8 | Erwinia | vB_EamM_RAY |
| Aeromonas | LAh10 | Erwinia | vB_EamM_Joad |
| Bacillus | vB_BspM_AgentSmith | Erwinia | AH04 |
| Burkholderia | FLC6 | Escherichia | vB_EcoM_Goslar |
| Burkholderia | FLC9 | Klebsiella | vB_KvM-Eowyn |
| Cronobacter | CR5 | Klebsiella | vB_KpM_FBKp24 |
| Edwardsiella | pEt-SU | Klebsiella | KpLz-2_45 |
| Erwinia | pEa_SNUABM_29 | Klebsiella | Miami |
| Erwinia | pEa_SNUABM_11 | Klebsiella | N1M2 |
| Erwinia | vB_EamM_Asesino | Kosakonia | Kc263 |
| Erwinia | vB_EamM_Huxley | Photobacterium | PDCC-1 |
| Erwinia | pEa_SNUABM_8 | Proteus | 10 |
| Erwinia | vB_EamM_ChrisDB | Pseudomonas | pPa_SNUABM_DT01 |
| Erwinia | vB_EamM_Caitlin | Pseudomonas | 201phi2-1 |
| Erwinia | phiEaH2 | Pseudomonas | PhiPA3 |
| Erwinia | Wellington | Pseudomonas | phiKZ |
| Erwinia | Derbicus | Pseudomonas | PA1C |
| Erwinia | vB_EamM_Phobos | Pseudomonas | Psa21 |
| Erwinia | pEa_SNUABM_54 | Pseudomonas | Phabio |
| Erwinia | PhiEaH1 | Serratia | Moabite |
| Pseudomonas | Noxifer | Vibrio | JM-2012 |
| Pseudomonas | EL | Vibrio | vB_VmeM-Yong MS32 |
| Pseudomonas | OBP | Vibrio | BONAISHI |
| Ralstonia | RSL2 | Vibrio | vB_pir03 |
| Ralstonia | RSF1 | Vibrio | vB_VpaM_sm033 |
| Ralstonia | RP31 | Vibrio | pVa-21 |
| Salmonella | STsAS | Vibrio | Aphrodite1 |
| Salmonella | SPAsTU | Vibrio | VP4B |
| Salmonella | SPN3US | Vibrio | pTD1 |
| Salmonella | vB_SalM_SA002 | Xanthomonas | Xoo-sp14 |
| Serratia | PCH45 | Xanthomonas | vB_XciM_LucasX |

**Table S2. VICTOR predictions of taxonomic groups, Related to Figure 1.** Predicted family, subfamily, genus, and species categories from VICTOR were used to guide our inclusion of phages in our analyses.

| Genomes | species | genus | subfamily | family |
|---|---|---|---|---|
| Mycoplasma phage P1 (NC_002515) | 41 | 11 | 3 | 1 |
| Clostridium phage phiCP7R (NC_017980) | 46 | 14 | 4 | 2 |
| Clostridium phage phiCPV4 (NC_018083) | 46 | 14 | 4 | 2 |
| Escherichia phage T4 (NC_000866) | 40 | 10 | 2 | 3 |
| Bacillus phage G (NC_023719) | 51 | 16 | 6 | 3 |
| Erwinia phage vB_EamM_Alexandra (NC_047995) | 69 | 23 | 10 | 3 |
| Cronobacter phage vB_CsaP_Ss1 (KM058087) | 5 | 1 | 1 | 4 |
| Pectobacterium phage ZF40 (NC_019522) | 47 | 15 | 5 | 4 |
| Erwinia phage phiEa21-4 (NC_011811) | 63 | 19 | 7 | 4 |
| Erwinia phage phiEa104 (NC_015292) | 63 | 19 | 7 | 4 |
| Erwinia phage vB_Eam-MM7 (NC_041978) | 63 | 19 | 7 | 4 |
| Yersinia phage vB_YenP_ISAO8 (NC_028850) | 53 | 22 | 9 | 4 |
| Yersinia phage phiR8-01 (NC_047951) | 68 | 22 | 9 | 4 |
| Salmonella phage ZCSE2 (NC_048179) | 74 | 26 | 11 | 4 |
| Salmonella phage SE4 (NC_048764) | 74 | 26 | 11 | 4 |
| Salmonella phage BP63 (NC_031250) | 77 | 26 | 11 | 4 |
| Salmonella phage UPF_BP2 (NC_048649) | 77 | 26 | 11 | 4 |
| Salmonella phage SE13 (NC_048763) | 77 | 26 | 11 | 4 |
| Salmonella phage yarpen (NC_048863) | 77 | 26 | 11 | 4 |
| Salmonella phage birk (NC_048864) | 77 | 26 | 11 | 4 |
| Staphylococcus phage PALS_2 (MN091626) | 21 | 5 | 8 | 5 |
| Yersinia phage phiR1-37 (NC_016163) | 44 | 12 | 8 | 5 |
| Bacillus phage PBS1 (NC_043027) | 67 | 21 | 8 | 5 |
| Burkholderia phage FLC9 (LC667451) | 8 | 2 | 12 | 5 |
| Klebsiella phage vB_KvM-Eowyn (LR881104) | 9 | 3 | 12 | 5 |
| Vibrio phage vB_VmeM-Yong XC31 (MK308674) | 15 | 4 | 12 | 5 |
| Pseudomonas phage pPa_SNUABM_DT01 (MW735835) | 30 | 6 | 12 | 5 |
| Bacillus phage vB_BspM_AgentSmith (MW749006) | 31 | 7 | 12 | 5 |
| Xanthomonas phage Xoo-sp14 (MT939492) | 28 | 8 | 12 | 5 |
| Xanthomonas phage vB_XciM_LucasX (MW825358) | 33 | 8 | 12 | 5 |
| Erwinia phage pEa_SNUABM_54 (MW879341) | 36 | 9 | 12 | 5 |
| Vibrio phage JM-2012 (NC_017975) | 45 | 13 | 12 | 5 |
| Ralstonia phage RP31 (AP017925) | 1 | 17 | 12 | 5 |
| Burkholderia phage FLC6 (LC592711) | 7 | 17 | 12 | 5 |
| Ralstonia phage RSF1 (NC_028899) | 54 | 17 | 12 | 5 |
| Ralstonia phage RSL2 (NC_028950) | 55 | 17 | 12 | 5 |
| Salmonella phage vB_SalM_SA002 (MN445183) | 23 | 18 | 12 | 5 |
| Proteus phage 10 (MT661596) | 25 | 18 | 12 | 5 |
| Erwinia phage pEa_SNUABM_37 (MW845760) | 35 | 18 | 12 | 5 |
| Erwinia phage AH06 (MZ501268) | 39 | 18 | 12 | 5 |
| Erwinia phage vB_EamM_RAY (NC_041973) | 62 | 18 | 12 | 5 |
| Pseudomonas phage 201phi2-1 (EU197055) | 2 | 20 | 12 | 5 |

| | | | | |
|---|---|---|---|---|
| Pseudomonas phage PhiPA3 (HQ630627) | 3 | 20 | 12 | 5 |
| Pseudomonas phage Phabio (MF042360) | 10 | 20 | 12 | 5 |
| Pseudomonas phage Psa21 (MK552327) | 16 | 20 | 12 | 5 |
| Pseudomonas phage PA1C (MK599315) | 17 | 20 | 12 | 5 |
| Pseudomonas phage phiKZ (NC_004629) | 42 | 20 | 12 | 5 |
| Pseudomonas phage Noxifer (NC_041994) | 64 | 20 | 12 | 5 |
| Escherichia phage vB_EcoM_Goslar (NC_048170) | 71 | 24 | 12 | 5 |
| Vibrio phage pVa-21 (KY499642) | 6 | 25 | 12 | 5 |
| Salmonella phage STsAS (MH221128) | 12 | 25 | 12 | 5 |
| Salmonella phage SPAsTU (MH221129) | 13 | 25 | 12 | 5 |
| Erwinia phage pEa_SNUABM_29 (MW812339) | 32 | 25 | 12 | 5 |
| Erwinia phage pEa_SNUABM_11 (MW845758) | 34 | 25 | 12 | 5 |
| Erwinia phage phiEaH2 (NC_019929) | 48 | 25 | 12 | 5 |
| Cronobacter phage CR5 (NC_021531) | 49 | 25 | 12 | 5 |
| Salmonella phage SPN3US (NC_027402) | 52 | 25 | 12 | 5 |
| Erwinia phage vB_EamM_Phobos (NC_031043) | 56 | 25 | 12 | 5 |
| Erwinia phage vB_EamM_Asesino (NC_031107) | 57 | 25 | 12 | 5 |
| Erwinia phage vB_EamM_Caitlin (NC_031120) | 58 | 25 | 12 | 5 |
| Erwinia phage pEa_SNUABM_8 (MW760841) | 59 | 25 | 12 | 5 |
| Erwinia phage vB_EamM_ChrisDB (NC_031126) | 59 | 25 | 12 | 5 |
| Erwinia phage vB_EamM_Huxley (NC_031127) | 60 | 25 | 12 | 5 |
| Erwinia phage Wellington (NC_048016) | 70 | 25 | 12 | 5 |
| Erwinia phage Derbicus (NC_048173) | 72 | 25 | 12 | 5 |
| Serratia phage PCH45 (MN334766) | 22 | 27 | 12 | 5 |
| Klebsiella phage vB_KpM_FBKp24 (MW394391) | 29 | 27 | 12 | 5 |
| Erwinia phage PhiEaH1 (NC_023610) | 50 | 27 | 12 | 5 |
| Serratia phage Moabite (NC_048792) | 75 | 27 | 12 | 5 |
| Klebsiella phage KpLz-2_45 (NC_061418) | 78 | 27 | 12 | 5 |
| Pseudomonas phage OBP (JN627160) | 4 | 28 | 12 | 5 |
| Erwinia phage vB_EamM_Joad (MF459647) | 11 | 28 | 12 | 5 |
| Aeromonas phage CF8 (MK774614) | 18 | 28 | 12 | 5 |
| Aeromonas phage LAh10 (MK838116) | 19 | 28 | 12 | 5 |
| Aeromonas phage PS1 (MN032614) | 20 | 28 | 12 | 5 |
| Klebsiella phage N1M2 (MN642089) | 24 | 28 | 12 | 5 |
| Klebsiella phage Miami (MT701590) | 26 | 28 | 12 | 5 |
| Kosakonia phage Kc263 (MZ348422) | 37 | 28 | 12 | 5 |
| Erwinia phage AH04 (MZ501267) | 38 | 28 | 12 | 5 |
| Pseudomonas phage EL (NC_007623) | 43 | 28 | 12 | 5 |
| Vibrio phage pTD1 (NC_041916) | 61 | 28 | 12 | 5 |
| Vibrio phage Aphrodite1 (NC_042100) | 65 | 28 | 12 | 5 |
| Vibrio phage VP4B (NC_042136) | 66 | 28 | 12 | 5 |
| Edwardsiella phage pEtSU (NC_048182) | 73 | 28 | 12 | 5 |
| Photobacterium phage PDCC-1 (NC_048821) | 76 | 28 | 12 | 5 |
| Aeromonas phage pAEv1810 (OL964756) | 79 | 28 | 12 | 5 |
| Vibrio phage BONAISHI (MH595538) | 14 | 29 | 12 | 5 |
| Vibrio phage vB_pir03 (MT811961) | 27 | 29 | 12 | 5 |

| | | | | |
|---|---|---|---|---|
| Vibrio phage vB_VpaM_sm033 (OV032902) | 80 | 29 | 12 | 5 |

**Table S3. Core genome blocks by function, Related to Figure 2.** The core genome numbers were determined with homologies within and across the genomes of ΦKZ, Goslar, and RAY. Each block is color coded and marked by number, and the last column indicates putative functions from PSI-BLAST hits, many of which are hypothetical. If a core gene has multiple homologs in one phage (for instance, cg42), they will be lettered by numerical order (for instance, RAY gp017 = cg42A, RAY gp018 = cg42B, and RAY gp019 = cg42C). Genes unique to chimallin-encoding phage are marked with an asterisk.

| Block Number | Core Genome Number | Goslar | ΦKZ | RAY | Putative Function |
|---|---|---|---|---|---|
| 1 | cg1 | gp192 | gp049 | gp219* | hypothetical protein |
| | cg2 | gp191 | gp050 | gp220 | virion DNAP |
| | cg3 | gp190 | gp052 | gp221* | hypothetical protein |
| | cg4 | gp189 | gp054 | gp222* | Nuclear Shell Protein |
| | cg5 | gp188 | gp055 | gp070, gp223 | putative DNA-directed RNA polymerase beta subunit |
| | cg6 | gp184 | gp059 | gp229* | hypothetical protein |
| 2 | cg7 | gp180 | gp062 | gp236* | hypothetical protein |
| | cg8 | gp178 | gp065 | gp238 | putative nuclease |
| | cg9 | gp177 | gp066 | gp239* | hypothetical protein |
| | cg10 | gp176 | gp067 | gp240 | hypothetical protein |
| | cg11 | gp175 | gp068 | gp243 | putative nvRNAP (non-virion RNAP) sigma factor |
| | cg12 | gp174 | gp069 | gp244* | hypothetical protein |
| | cg13 | gp173 | gp070 | gp245 | hypothetical protein |
| | cg14 | gp172 | n/a | gp246* | hypothetical protein |
| | cg15 | gp171 | gp071, gp073 | gp248 | putative DNA directed RNA polymerase beta subunit |
| | cg16 | gp165 | gp074 | gp249 | putative DNA directed RNA polymerase beta subunit |
| | cg17 | gp164 | gp075 | gp250 | putative RAD2/SF2 helicase |
| 3 | cg18 | gp081 | gp077 | gp267 | hypothetical protein |
| | cg19 | gp079 | gp079 | gp269* | hypothetical protein |

| | | | | | |
|---|---|---|---|---|---|
| | cg20 | gp078 | gp080 | gp270 | putative DNA-directed RNA polymerase beta prime subunit |
| | cg21 | gp068 | gp082 | gp285 | putative DNA polymerase |
| | cg22 | gp067 | gp084 | gp286 | putative virion structural protein |
| 4 | cg23 | gp063 | gp087 | gp290 | putative virion structural protein |
| | cg24 | gp062 | gp088 | gp291 | putative virion structural protein |
| | cg25 | gp061 | gp089 | gp292 | hypothetical protein |
| | cg26 | gp060 | gp090 | gp293* | putative virion structural protein |
| | cg27 | gp058 | gp093, gp162, gp163 | gp295, gp298* | virion structural protein/internal head |
| 5 | cg28 | gp051 | gp098 | gp304 | hypothetical protein |
| | cg29 | gp050 | gp099 | gp305 | putative virion structural protein |
| | cg30 | gp049 | gp100 | gp306* | hypothetical protein |
| | cg31 | gp048 | gp101 | gp307 | putative virion structural protein |
| | cg32 | gp008 | gp188 | gp311 | putative thymidylate kinase |
| | cg33 | gp043 | gp118 | gp315 | putative DnaB helicase |
| | cg34 | gp041 | gp120 | gp317 | major capsid protein |
| | cg35 | gp040 | gp122 | gp001* | hypothetical |
| | cg36 | gp039 | gp123 | gp002 | putative RNA polymerase beta subunit |
| | cg37 | gp036 | gp129 | gp006 | putative virion structural protein |
| | cg38 | gp035 | gp128 | gp007 | putative virion structural protein |
| | cg39 | gp032 | gp139 | gp010 | virion structural protein |
| | cg40 | gp010 | gp140 | gp012 | hypothetical |
| | cg41 | gp030 | gp130 | gp016* | virion structural protein |
| | cg42 | gp025, gp028, gp029 | gp131, gp132, gp134, gp135 | gp017, gp018, gp019 | virion structural protein/tail fiber |

| | | | | | |
|---|---|---|---|---|---|
| | cg43 | gp013 | gp164 | gp021 | structural protein |
| | cg44 | gp012 | gp165 | gp023 | putative SbcC-like protein |
| 6 | cg45 | gp249 | gp161 | gp144* | hypothetical |
| | cg46 | gp248 | gp157 | gp145* | virion structural protein |
| | cg47 | gp246 | gp155 | gp147 | putative ribonuclease HI |
| | cg48 | gp244 | gp153 | gp149* | hypothetical |
| | cg49 | gp243 | gp152 | gp150 | putative UvsX protein |
| | cg50 | gp240 | gp149 | gp154 | virion structural protein |
| | cg51 | gp238 | gp147 | gp156* | hypothetical |
| | cg52 | gp235 | gp171 | gp159* | hypothetical |
| | cg53 | gp234 | gp174 | gp160 | hypothetical |
| | cg54 | gp233 | gp182 | gp161 | virion structural protein |
| | cg55 | gp232 | gp181 | gp162 | putative lysozyme domain protein |
| | cg56 | gp231 | gp180 | gp163 | putative DNA-direct RNA polymerase beta subunit 2 |
| | cg57 | gp228 | gp178 | gp164 | putative RNA polymerase beta subunit |
| | cg58 | gp226 | gp177 | gp167* | hypothetical protein |
| | cg59 | gp225 | gp176 | gp168 | hypothetical protein |
| | cg60 | gp223 | gp175 | gp170 | putative virion structural protein |
| 7 | cg61 | gp218 | gp030 | gp178 | putative major virion structural protein |
| | cg62 | gp217 | gp029 | gp179 | putative tail sheath protein |
| | cg63 | gp216 | gp028 | gp180 | putative virion structural protein |
| | cg64 | gp215 | gp027 | gp181 | putative virion structural protein |
| | cg65 | gp214 | gp026 | gp182* | putative structural protein |
| | cg66 | gp213 | gp025 | gp183 | putative terminase large subunit function |
| | cg67 | gp211 | gp032 | gp187 | hypothetical protein |
| | cg68 | gp202 | gp042 | gp209 | hypothetical protein |

**Table S4. GFP fusion proteins used in this study, Related to STAR Methods.**

| Bacterial strain | Plasmid backbone | Fusion protein | Known or putative function |
|---|---|---|---|
| E. amylovora ATCC 29780 | pHERD30T | GFP-RAYgp222 | Major nuclear shell protein (chimallin/ChmA) |
| E. amylovora ATCC 29780 | pHERD30T | HNS-GFP | Histone-like nucleoid structuring protein (H-NS) |
| E. amylovora ATCC 29780 | pHERD30T | GFP-RAYgp002 | Non-virion RNA polymerase subunit β 1 |
| E. amylovora ATCC 29780 | pHERD30T | RAYgp248-GFP | Non-virion RNA polymerase subunit β 2 |
| E. amylovora ATCC 29780 | pHERD30T | GFP-RAYgp223 | Non-virion RNA polymerase subunit β’ 1 |
| E. amylovora ATCC 29780 | pHERD30T | RAYgp249-GFP | Non-virion RNA polymerase subunit β’ 2 |
| E. amylovora ATCC 29780 | pHERD30T | RAYgp220-GFP | DNA polymerase |
| E. amylovora ATCC 29780 | pHERD30T | RAYgp116-GFP | HslUV-like protease |
| E. amylovora ATCC 29780 | pHERD30T | GFP-RAYgp150 | UvsX/RecA |
| E. amylovora ATCC 29780 | pHERD30T | RAYgp153-GFP | DNA processing (DprA) |
| E. amylovora ATCC 29780 | pHERD30T | RAYgp250-GFP | Non-virion SF2 helicase |
| E. amylovora ATCC 29780 | pHERD30T | RAYgp315-GFP | Replicative helicase (DnaB) |
| E. amylovora ATCC 29780 | pHERD30T | RAYgp039-GFP | Stringent starvation (SspB) |
| E. amylovora ATCC 29780 | pHERD30T | RAYgp049-GFP | tRNA ligase (RtcB) |
| E. amylovora ATCC 29780 | pHERD30T | RAYgp064-GFP | exonuclease |
| E. amylovora ATCC 29780 | pHERD30T | RAYgp311-GFP | Thymidylate kinase (TMK) |
| E. amylovora ATCC 29780 | pHERD30T | RAYgp094-GFP | XRE family transcriptional regulator/repressor |
| E. amylovora ATCC 29780 | pHERD30T | RAYgp317-GFP | Major capsid protein (MCP) |
| E. amylovora ATCC 29780 | pHERD30T | RAYgp179-GFP | Tail sheath |
| E. amylovora ATCC 29780 | pHERD30T | RAYgp154-GFP | Virion RNA polymerase subunit β 2 |
| E. amylovora ATCC 29780 | pHERD30T | GFP-RAYgp163 | Virion RNA polymerase subunit β’ 1 |
| E. amylovora ATCC 29780 | pHERD30T | RAYgp270-GFP | Virion RNA polymerase subunit β’ 2 |
| E. amylovora ATCC 29780 | pHERD30T | GFP-RAYgp131 | Virion SF2 helicase |
| E. amylovora ATCC 29780 | pHERD30T | RAYgp299-GFP | Head protein |
| E. amylovora ATCC 29780 | pHERD30T | GFP-RAYgp210 | Phage tubulin (PhuZ) |
| E. amylovora ATCC 29780 | pHERD30T | GFP-RAYgp210D198A | Catalytically dead phage tubulin (dPhuZ) |

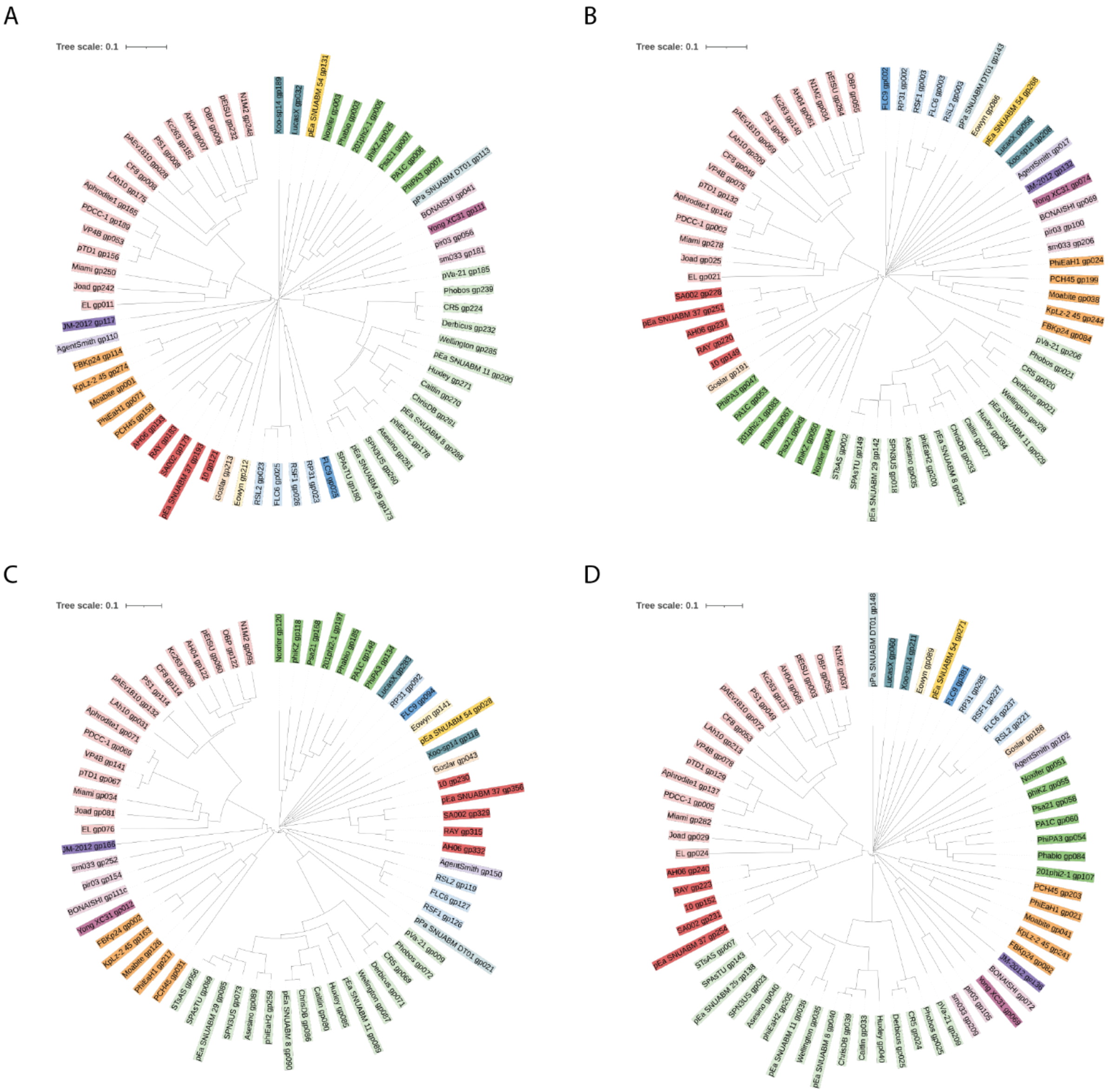


**Figure S1. Phylogenetic trees including Chimalliviridae members based on well-conserved phage proteins, Related to Figure 1.** (A) A protein tree of terminase large subunit homologs from Chimalliviridae. (B) A protein tree of DNA polymerase homologs from Chimalliviridae. (C) A protein tree of replicative (DnaB-like) helicases from Chimalliviridae. (D) A protein tree of non-virion RNA polymerase β’ subunit 1 homologs from Chimalliviridae. All homologs were found by the top PSI-BLAST hit. Chimalliviridae are color-coded by predicted genus as in Figure 1.

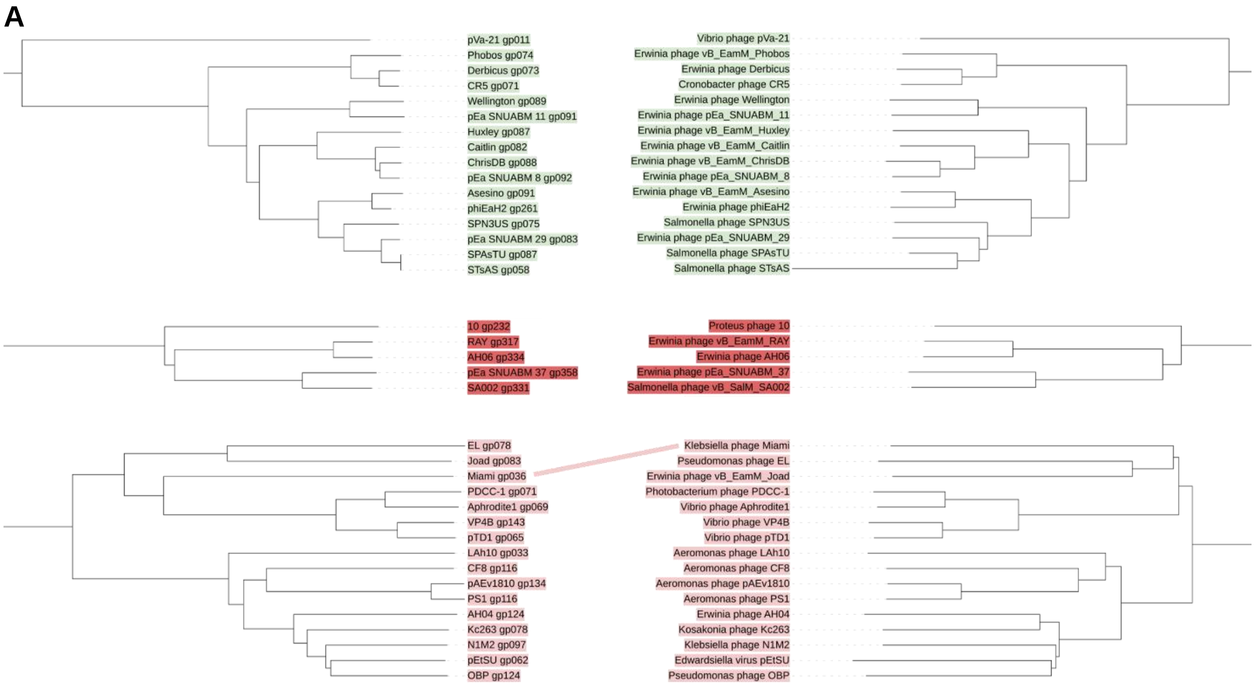


Major Capsid Protein vs. Whole Genome Tree

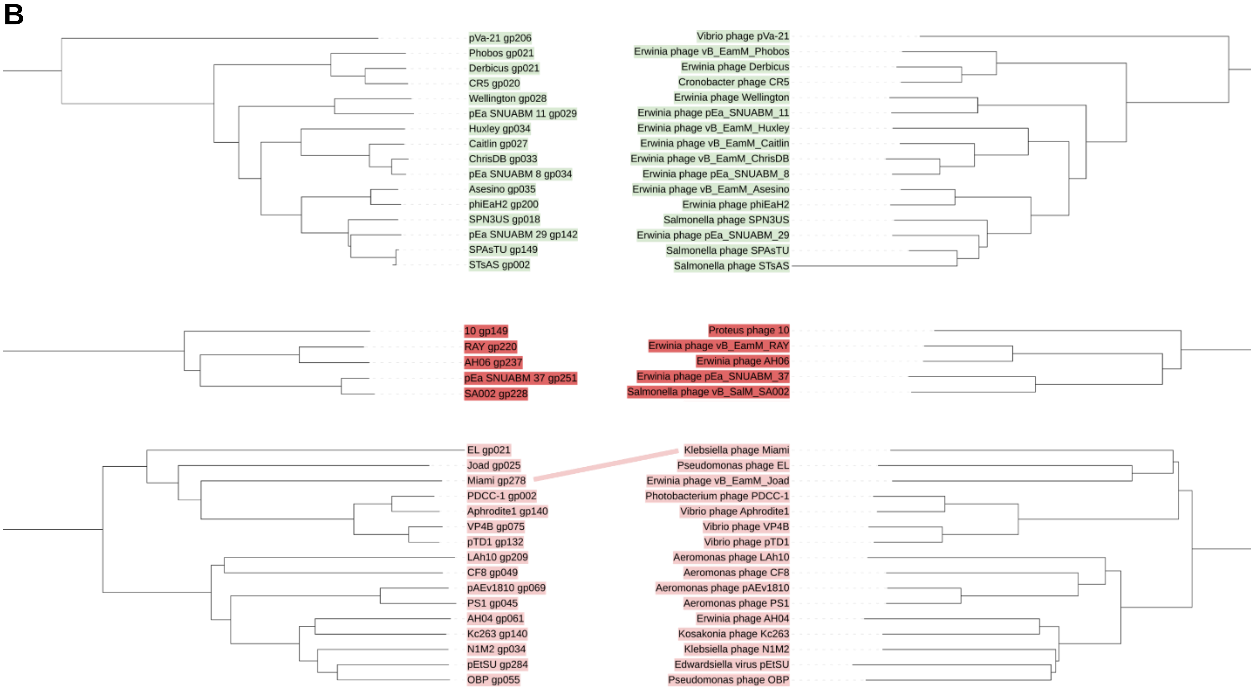


DNA Polymerase vs. Whole Genome Tree

**C**

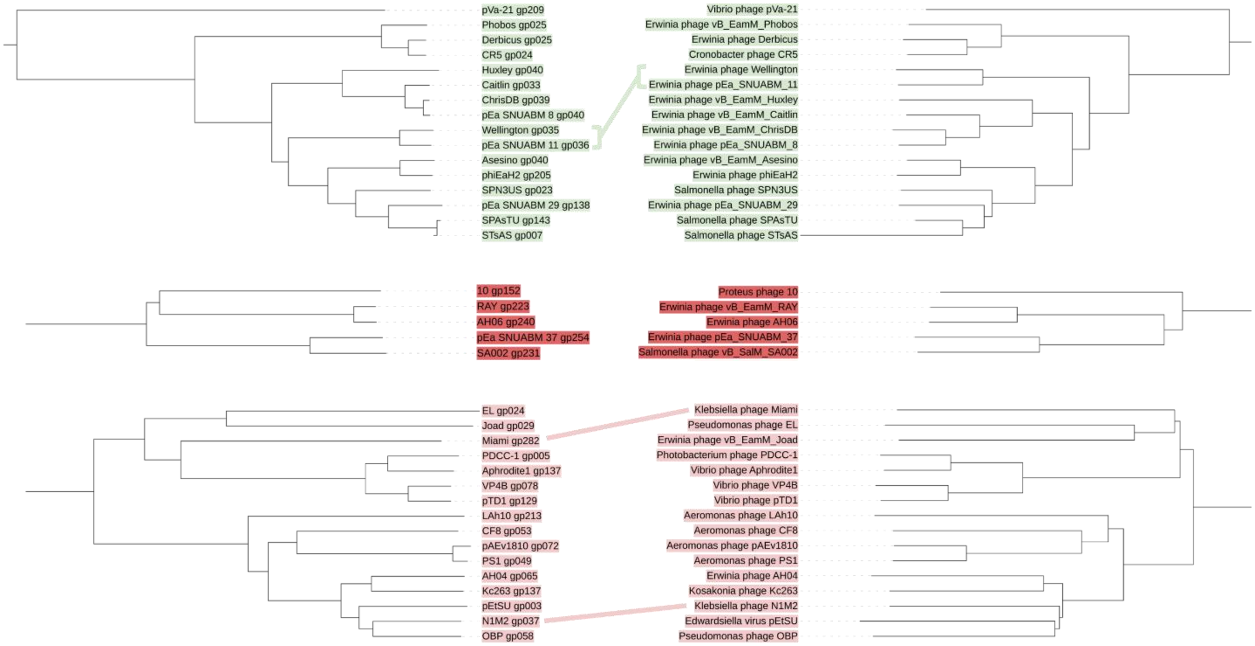


nvRNAP β’ Subunit 1 vs. Whole Genome Tree

**D**

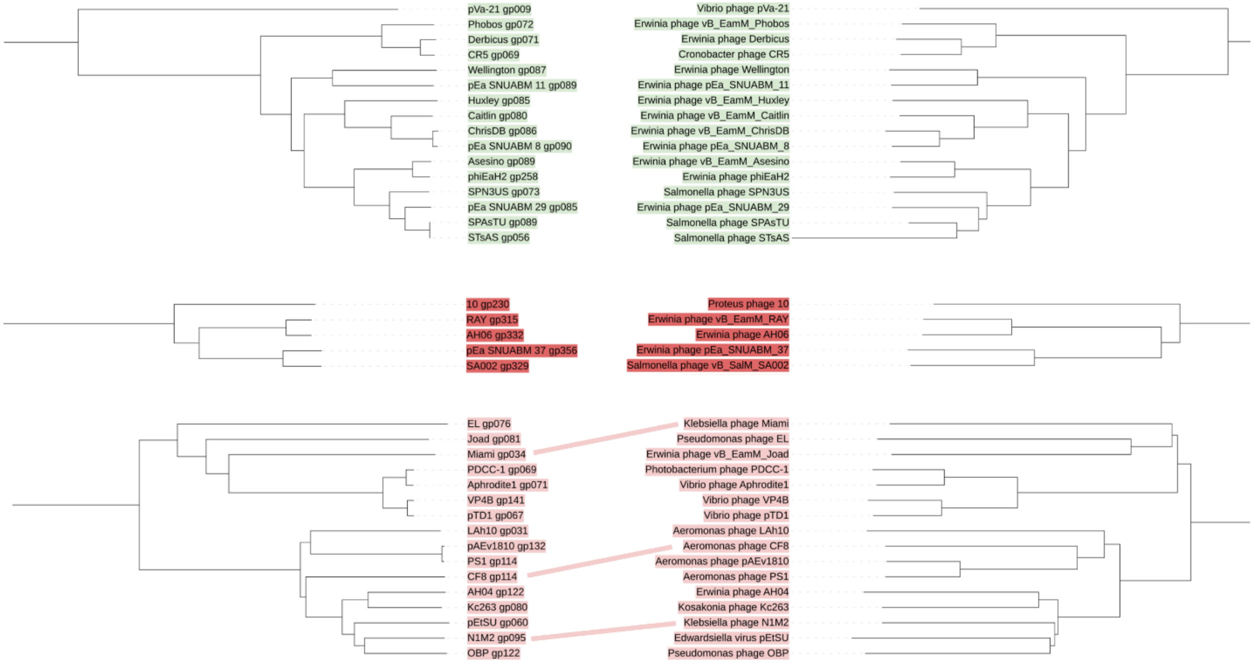


DnaB-like Helicase vs. Whole Genome Tree

E

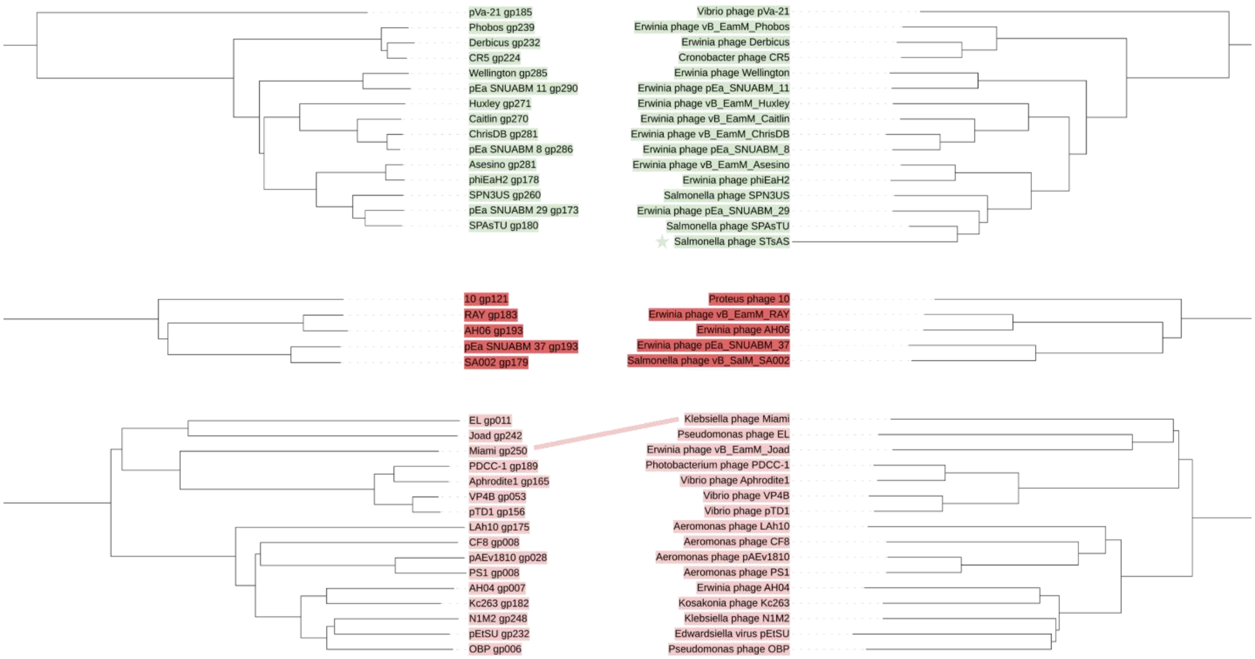


Terminase Large Subunit vs. Whole Genome Tree

**Figure S2. Congruence between protein and whole genome based phylogenetics trees, Related to Figure 1.** Mirrored trees comparing the whole genome phylogeny (right) with the phylogeny of individual proteins (left) were made for (A) the major capsid protein, (B) the DNA polymerase, (C) an RNA polymerase subunit, (D) the replicative helicase, and (E) the terminase large subunit. Minor discrepancies are pointed out with lines connecting the phage that have different branching patterns. In the case of the terminase, one phage was missing a homolog and is marked with a star. Clades are color-coded by predicted genus as in Figure 1. The light green, red, and light red clades were chosen for comparison because they were the largest clades and had the greatest potential to show how congruent the trees were (light green and light red) or were the clade containing RAY (red).

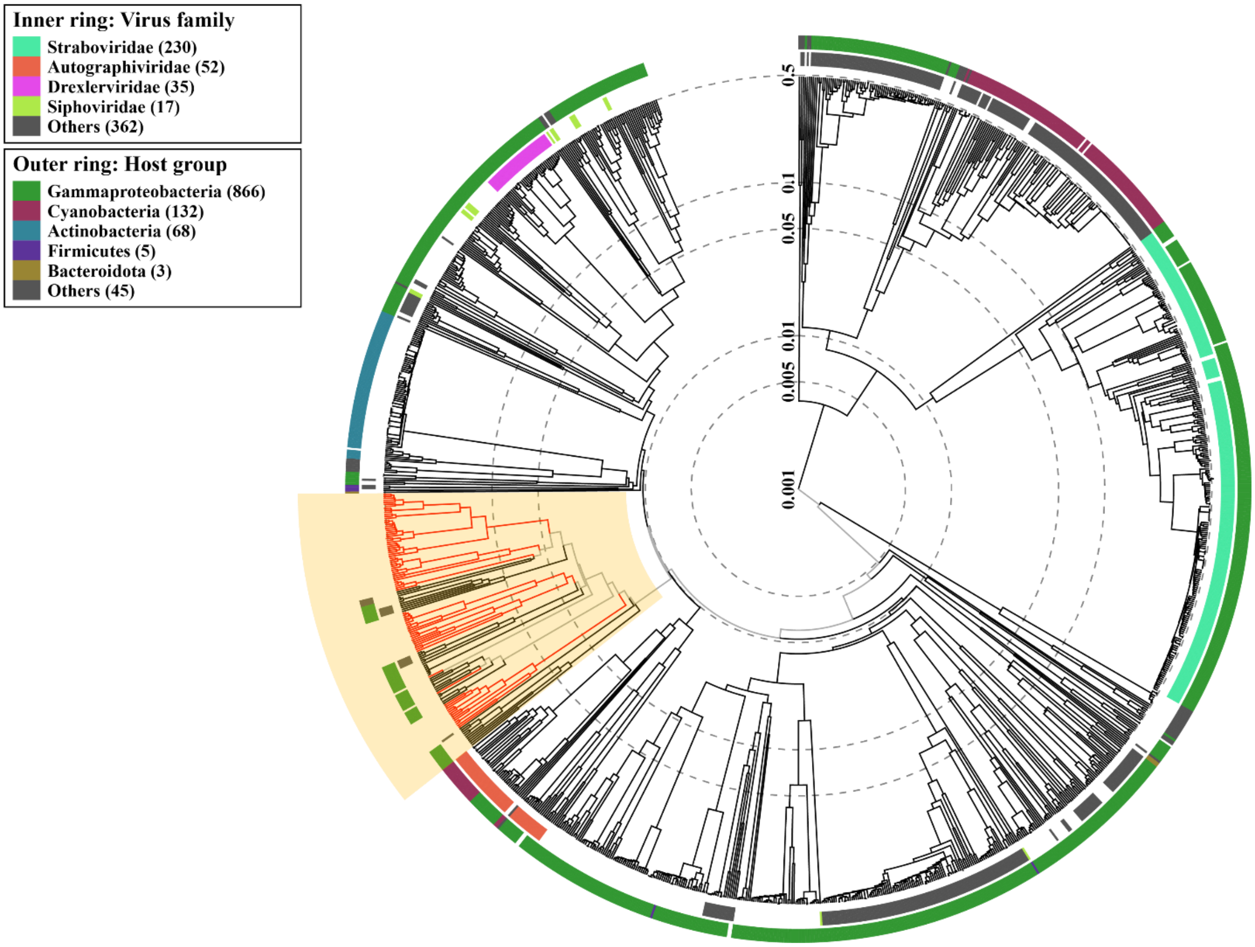


**Figure S3. ViPTree data showing clustering of metagenomes, Related to Figure 1 and Supplemental File 1.** Metagenome whole genome (complete or partial) sequences that encode chimallin homologs from the NCBI database (highlighted with red lines) cluster within the monophyletic clade of Chimalliviridae (highlighted in beige).

**A**

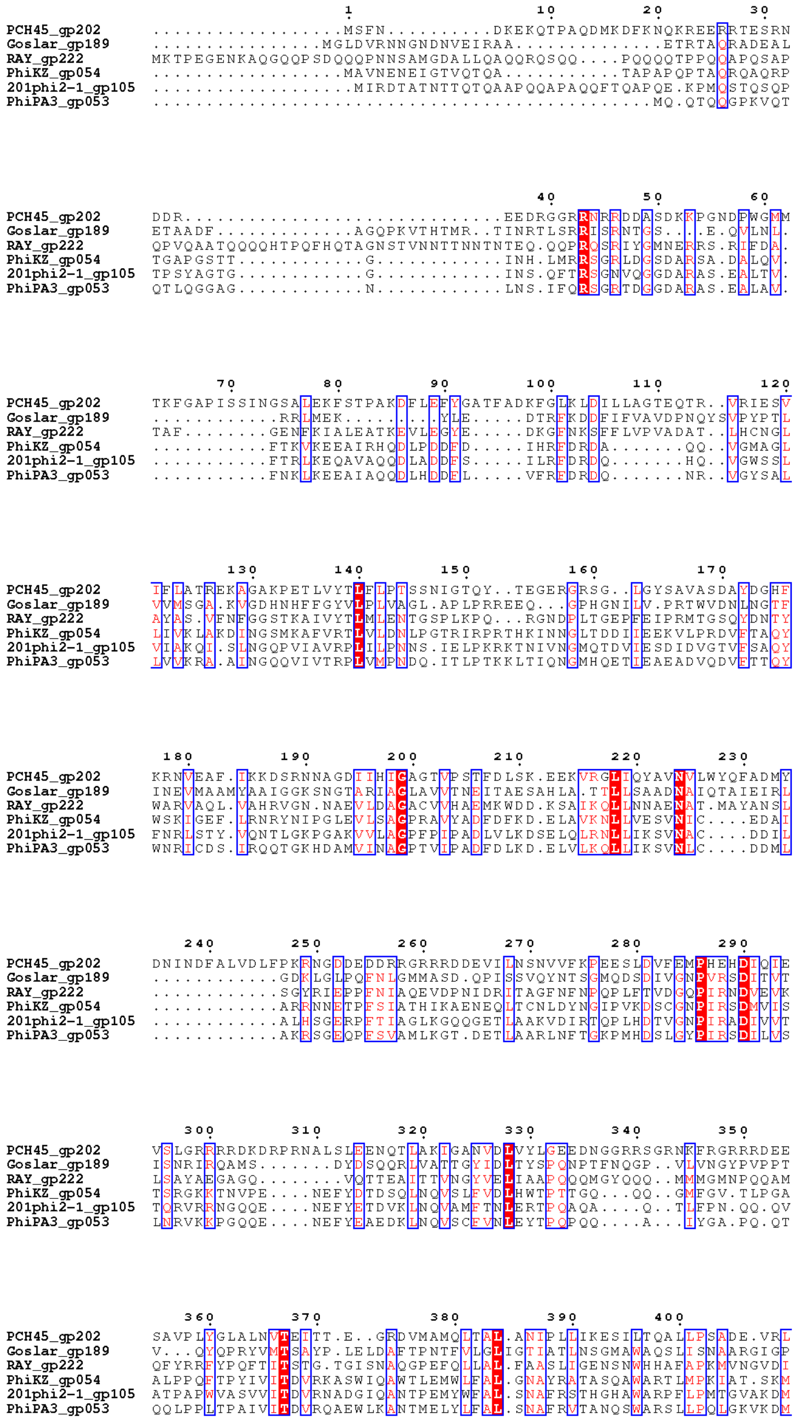
PCH45_gp202
Goslar_gp189
RAY_gp222
PhiKZ_gp054
201phi2-1_gp105
PhiPA3_gp053

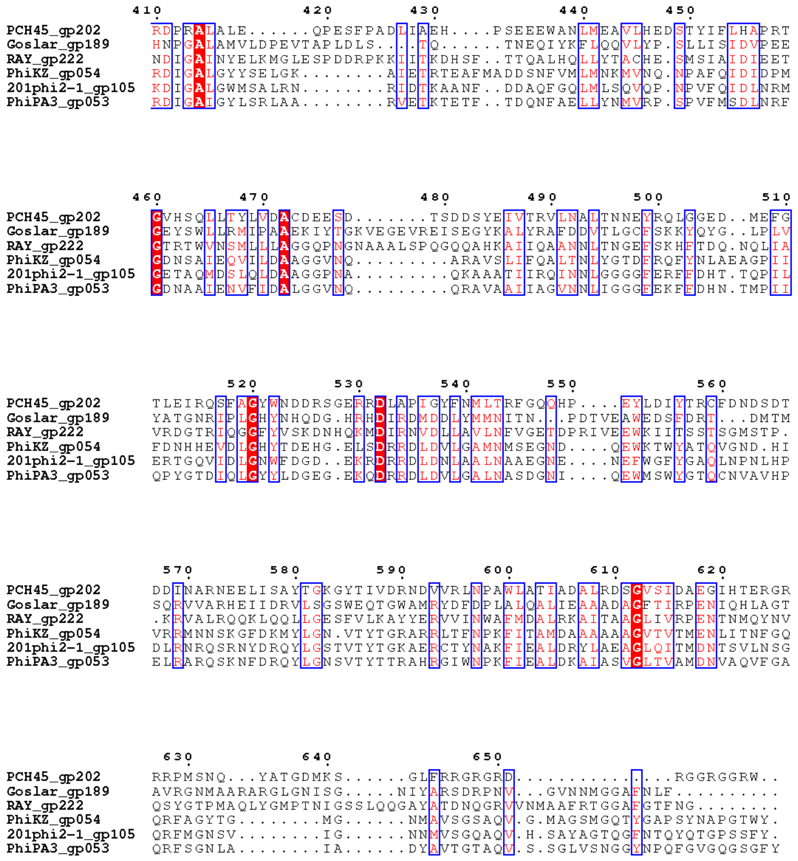
410 420 430 440 450
PCH45_gp202 RDPRALALE......QPESFPADLIAEH....PSEEEWANLMEAVLHEDSTYIFLHAPRT
Goslar_gp189 HNPGALAMVLDPEVTAPLDLS....TQ......TNEQIYKFLQQVLYP.SLLISIDVPEE
RAY_gp222 NDIGAINYELKMGLESPDDRPKKIITKDHSF..TTQALHQLLYTACHE.SMSIAIDIEET
PhiKZ_gp054 RDIGALGYYSELGK........AIETRTEAFMADDSNFVMLMNKMVNQ.NPAFQIDIDPM
201phi2-1_gp105 KDIGALGWMSALRN........RIDTKAANF..DDAQFGQLMLSQVQP.NPVFQIDLNRM
PhiPA3_gp053 RDIGAIGYLSRLAA........RVETKTETF..TDQNFAELLYNMVRP.SPVFMSDLNRF
460 470 480 490 500 510
PCH45_gp202 GVHSQLLTYLVDACDEESD.......TSDDSYEIVTRVLNALTNNEYRQLGGED..MEFG
Goslar_gp189 GEYSWLLRMIPAAEKIYTGKVEGEVREISEGYKALYRAFDDVTLGCFSKKYQYG..LPLV
RAY_gp222 GTRTWVNSMLLLAGGQPNGNAAALSPQGQQAHKAIIQAANNLTNGEFSKHFTDQ.NQLIA
PhiKZ_gp054 GDNSAIEQVILDAAGGVNQ.........ARAVSLIFQALTNLYGTDFRQFYNLAEAGPII
201phi2-1_gp105 GETAQMDSLQLDAAGGPNA.........QKAAATIIRQINNLGGGGFERFFDHT.TQPIL
PhiPA3_gp053 GDNAAIENVFIDALGGVNQ.........QRAVAAIIAGVNNLIGGGFEKFFDHN.TMPII
520 530 540 550 560
PCH45_gp202 TLEIRQSFAGYWNDDRSGERRDLAPIGYFNMLTRFGQQHP....EYLDIYTRCFDNDSDT
Goslar_gp189 YATGNRIPLGHYNHQDG.HRHDIRDMDDLYMMNITN...PDTVEAWEDSFDRT...DMTM
RAY_gp222 VRDGTRIQGGFYVSKDNHQKMDIRNVDLLAVLNFVGETDPRIVEEWKIITSSTSGMSTP.
PhiKZ_gp054 FDNHHEVDLGHYTDEHG.ELSDRRDLDVLGAMNMSEGND....QEWKTWYATQVGND.HI
201phi2-1_gp105 ERTGQVIDLGNWFDGD..EKRDRRDLDNLAALNAAEGNE....NEFWGFYGAQLNPNLHP
PhiPA3_gp053 QPYGTDIQLGYYLDGEG.EKQDRRDLDVLGALNASDGNI....QEWMSWYGTQCNVAVHP
570 580 590 600 610 620
PCH45_gp202 DDINARNEELISAYTGKGYTIVDRNDVVRLNPAWLATIADALRDSGVSIDAEGIHTERGR
Goslar_gp189 SQRVVARHEIIDRVLSGSWEQTGWAMRYDFDPLALQALIEAAADAGFTIRPENIQHLAGT
RAY_gp222 .KRVALRQQKLQQLLGESFVLKAYYERVVINWAFMDALRKAITAAGLIVRPENTNMQYNV
PhiKZ_gp054 VRRMNNSKGFDKMYLGN.VTYTGRARRLTFNPKFITAMDAAAAAAGVTVTMENLITNFGQ
201phi2-1_gp105 DLRNRQSRNYDRQYLGSTVTYTGKAERCTYNAKFIEALDRYLAEAGLQITMDNTSVLNSG
PhiPA3_gp053 ELRARQSKNFDRQYLGNSVTYTTRAHRGIWNPKFIEALDKAIASVGLTVAMDNVAQVFGA
630 640 650
PCH45_gp202 RRPMSNQ...YATGDMKS......GLFRRGRGRD............RGGRGGRW..
Goslar_gp189 AVRGNMAARARGLGNISG.....NIYARSDRPNV...GVNNMGGAFNLF.........
RAY_gp222 QSYGTPMAQLYGMPTNIGSSLQQGAYATDNQGRVVNMAAFRTGGAFGTFNG........
PhiKZ_gp054 QRFAGYTG........MG......NMAVSGSAQV.G.MAGSMGQTYGAPSYNAPGTWY.
201phi2-1_gp105 QRFMGNSV........IG......NNMVSGQAQV.H.SAYAGTQGFNTQYQTGPSSFY.
PhiPA3_gp053 QRFSGNLA........IA......DYAVTGTAQV.S.SGLVSNGGYNPQFGVGQGSGFY

**B**

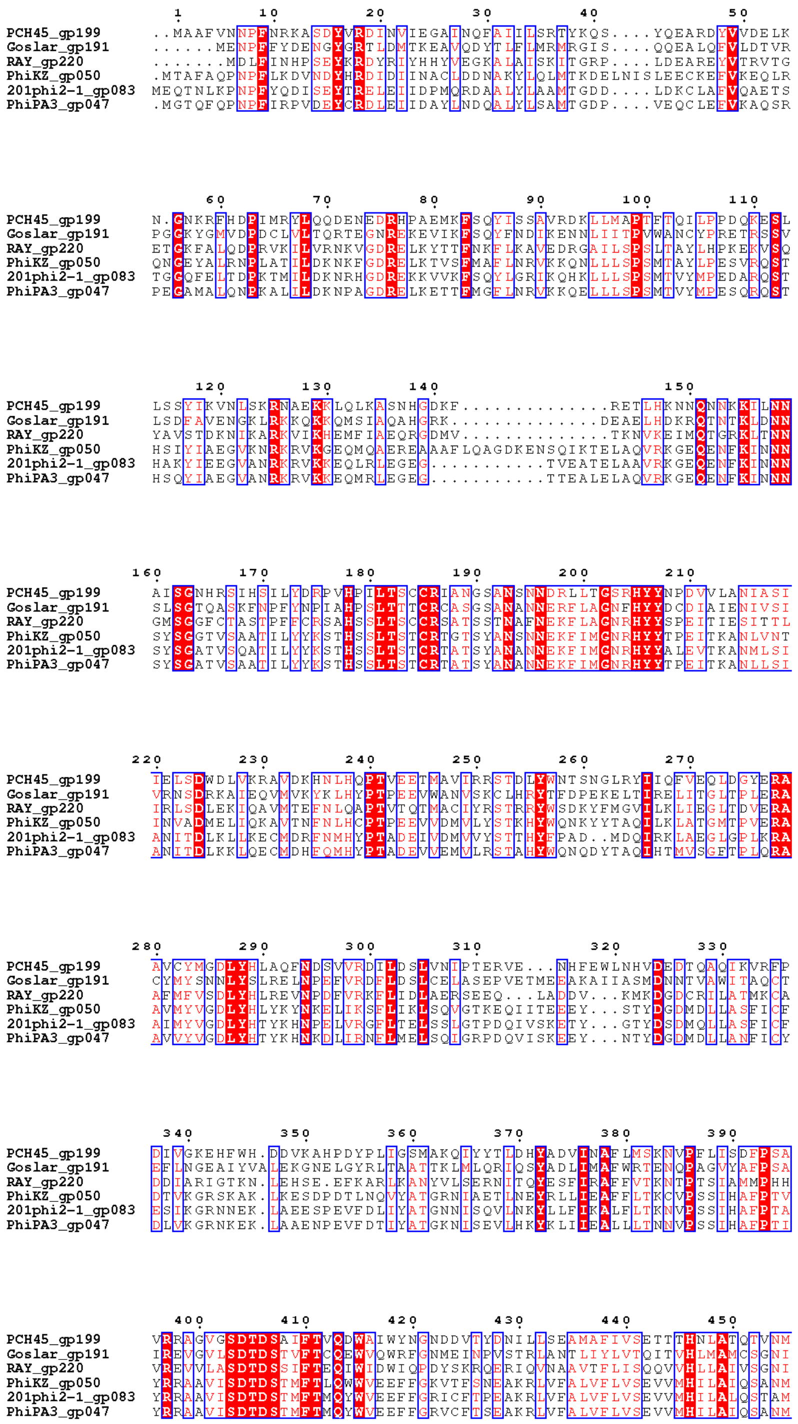
PCH45_gp199
Goslar_gp191
RAY_gp220
PhiKZ_gp050
201phi2-1_gp083
PhiPA3_gp047

```
                     460       470       480       490       500       510
PCH45_gp199         GVTDEKEVFRLSMKNEYLFPVFMLTSRAKHYAALQKVQEGIIFDKAKLEKKGVELHASNS
Goslar_gp191        GVAKEH.MYVMEMKNEYMFLILSLTSMGKNYYGYMTMREGVPINPPKLELKGPVMRDASA
RAY_gp220           GVADEH.LFRLAMKNEYYFETFVLTNMGKHYFATQAAREGNFFPKPKLELKGVHLRNSNV
PhiKZ_gp050         GVSKDK.LRLFAMKNEYYFAVLALTTRSKHYFASQDAQEGVMFNESRMEIKGVGLRDSKV
201phi2-1_gp083     GVAEKK.LRLLAMKNEYYFAVLSLTTRSKHYYASQDALEGVMFNKARMEVKGVGLRDSKV
PhiPA3_gp047        GVSKEK.LRLLAMKNEYYFAVLSLTTRSKHYYASQDAQEGVMFAKARMEVKGVGLRDSKV

                     520       530       540       550       560       570
PCH45_gp199         PEGIRKDLDKMIVDVLTKQERGEKISLHSMLVHVAEWEHRIFNSITAGETTYLKSARVKE
Goslar_gp191        PKMIVKRVERMMCGIMDKLLRNEKISIRLVLDDIISLENEIVESMKRGDPDFLTRVKVKP
RAY_gp220           PKEIRDRGNKLINDILDKSNVNEQFSVVDLLNQVGDIERFIINSITKGETIFLTKATIKD
PhiKZ_gp050         PKKINSRAKTLMEDIIKTVKTEEKIDLASILKEVGDIEREIIESVRSGKAEYLTSGQTKR
201phi2-1_gp083     PPRINKAAKKMMDHIITQIKAEQPLDLPALLKQIGDIERDIIESIFSGKAEYLTTGKVKQ
PhiPA3_gp047        PPKINKAAKKMMENIIKSVKEEIPLDLPAMLKEVADFERDIINSIFSGKSEYLTTGKCKK

                     580       590       600       610       620       630
PCH45_gp199         EDGYTKEAEKSRYMNFMLWSEVFADTYGASLPPPYSALAFSIIPDTKNKTKKWLDEWPNQ
Goslar_gp191        HVA.......VTKQSYDMWEEVFAQSYGHCQEPPYVGVRVPLATKNKTLLNDWIDSWEDK
RAY_gp220           KGSYAKPY.SSNYYHYCMWQEVFAEKYGQAPPLQYIGLKAKLGLSSQTAVNDWLDSLDDQ
PhiKZ_gp050         PESYKSED.NSTHKKGLVWKTVFAPSFGDAGDPPYSHVKISVTLNNKTAIKEWIDGIEDK
201phi2-1_gp083     LNAYKTES.NATYAKYKFWKEVFEPSFGFLEAPPYSFVKISVTINNRTRFEAWVDSIEDK
PhiPA3_gp047        LNAYKSED.NATYAKHRFWAEVFGPSYGGTEEPPYSFVKVSVTVDNRTRFNEWVESIEDK

                     640       650       660       670       680       690
PCH45_gp199         ERAERLRSFMARVKKDYLGEILLPEENVMSYGIPSEILDVIDVRKIVAKAVSPFYLFLES
Goslar_gp191        GLQQRFRDWIVRNDKKTISNLTIPLPVIEASGLPDIFIQGMDIRRLISTLVRSYYALMES
RAY_gp220           ALAERMRKFLVKYGKKSITQVVMPADIVMNMGVPPEIIAGIDLRNLIMENLSMVYSCLES
PhiKZ_gp050         KLAARIQAWVTEENKTSIGDFHIPSTVVETGRIPEVITRVADVRTIIGNTMGVFYLTLES
201phi2-1_gp083     GLAMRLKKWALDNNKKGITNFHVPMSVVENHGIPEVITRVANVRTVIANTMGAFYLLMES
PhiPA3_gp047        GLAMRLKKWAMENDKSGITTFHVPMTVVENHGIPSEITRVADVRTVISNTMGVFYLILES

                     700       710       720       730
PCH45_gp199         FEFYLQDKDQCKLIMDFISQEDVDNFKASKEESPSQKD
Goslar_gp191        YGIFISNKNNTLLAQDIAASYLPKQETAE.........
RAY_gp220           LGLYYVNDNATKLVSDEH....................
PhiKZ_gp050         LGIFLIDKNNDRLISDFY....................
201phi2-1_gp083     LGIMLIDKKNTRLISDFY....................
PhiPA3_gp047        LGIFLIDKDNSRLISDFY....................
```

C

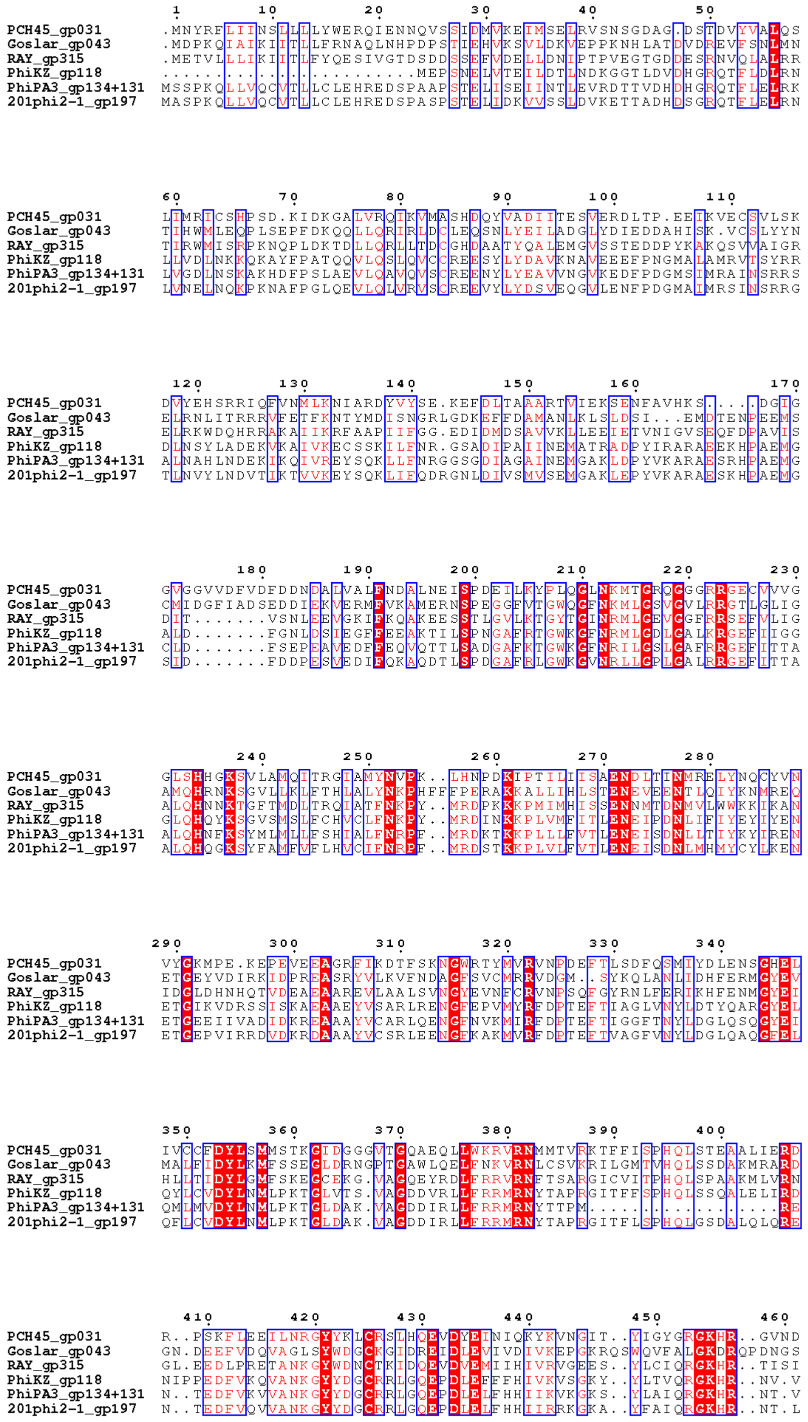
PCH45_gp031
Goslar_gp043
RAY_gp315
PhiKZ_gp118
PhiPA3_gp134+131
201phi2-1_gp197

```
                          470          480       490          500       510
PCH45_gp031         TPEQDKICWYQIGE..LGI.LDDINEPESRAMDKPGR.PVL..ADDDFWASTPTETYAMN
Goslar_gp043        TKPEDKHFAMPFQT..IGMLPDDYGKKAVYCRKVGGQ.PVS.EGGKGPWNKFDENAAPSN
RAY_gp315           TPERDKYCVYKFEPGV.GI.LDDVN.GKDKSRKHVAGETNSDG.GGAPWFG.........
PhiKZ_gp118         SDSKHHYFVMPFDPDRIGGIRWDIDKEENNYMDFVPSLSNMQATGGDDWGY.........
PhiPA3_gp134+131    TSEEDQYVLLPFSS..CGIIPWDIDKEEDYSLKIVPG.SIIGGMDDDAWSN.........
201phi2-1_gp197     TPEEDQYVIIPMSP..VGTMPWDIDKEEDYCIRVIPG.NNIGSDGDDAWD..........

PCH45_gp031         ........
Goslar_gp043        ELQFDLEF
RAY_gp315           ........
PhiKZ_gp118         ........
PhiPA3_gp134+131    ........
201phi2-1_gp197     ........
```

D

i

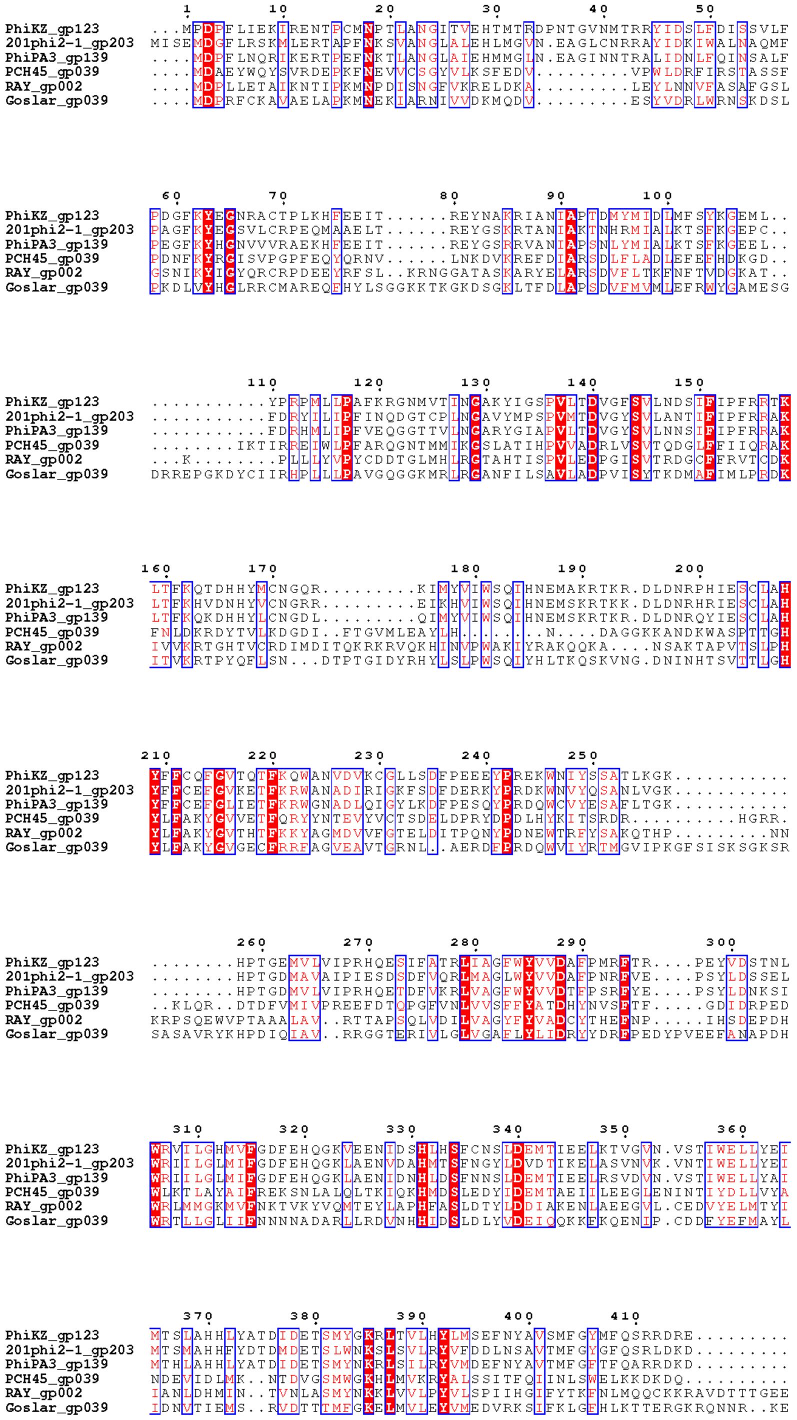
PhiKZ_gp123
201phi2-1_gp203
PhiPA3_gp139
PCH45_gp039
RAY_gp002
Goslar_gp039

```
                    420       430          440       450       460
                      .         .            .         .         .
PhiKZ_gp123      ...WTVQELNEGLKRSFKLQTAI...KRLTVDHGELDTMSNPNSSMLIKGTSILVTQDR.
201phi2-1_gp203  ...WTINEINDALKRSFKPNTAV...RRLSVDHGEFDTVSYPGDNKAIKLTSIVVPQDK.
PhiPA3_gp139     ...WTAQEINDALKRSFKLNTCI...RKLTSEHGEMETISMPGDNKAIKITSILVPQDR.
PCH45_gp039      ...LTYKKVKWLLGRYLHPNSFLGITKN....HGEKTNVQYPGDNMLFKHTLISVRQIDA
RAY_gp002        IMVFTEDTLFDVLGRNLKPEAIN...KVKGPDHGMISSVAAPGDNKMFKINNKITLQQNA
Goslar_gp039     ...MTNRDVQKALRNQVATEAILNIQSNRDKREKVATSIQSPGDCMLFKVSTHVVLQSQN

               470       480       490       500       510       520
                 .         .         .         .         .         .
PhiKZ_gp123      AKTAKAHNKSLINDSSRIIHASIAEVGQYKNQPKNNPDGRGRLNMYTKVGPTGLVERREE
201phi2-1_gp203  AKSKGSHNKSLIGDSSRLIHVSLADVGQYKNQPKNNPDGRGRLNLYVDVGPDGTIQRGKD
PhiPA3_gp139     AKTSKAHNKSLIGDSTRLIHASIAEVGQYKNQPKNNPDGRGRLNLFVGVRYDGMIERRDD
PCH45_gp039      VMTANGKSKINVDDPGYHLHPSIFDSGSMVNFPKPNPDGRTKLNPFITTSESGKILPNPK
RAY_gp002        TQSGGRRNESPMTDDSKGLDVSIADCASYLHITKPDPTGRSLFNPFKLLVRDK.LLPSVK
Goslar_gp039     AGG.....AALADESRLVSGSIPFCASYLNLPHSEPTGRQKFSMYAPIDERGRLIITPQ

               530       540
                 .         .
PhiKZ_gp123      VREIIDNAQLMFRAK.................
201phi2-1_gp203  DREFLDSVQARFNR..................
PhiPA3_gp139     DRELLDATQKRFAR..................
PCH45_gp039      YYRMLKDEVG...SEIG.FDN...........
RAY_gp002        YEKLFSVTQA..............IIYRRNI..
Goslar_gp039     IANDVAATNAGLKKDVGRFDEEIVEINDRDIID
```

**ii**

```
                       1        10        20        30        40        50
PCH45_gp216         ....MEKTRVASSVQEAVVSNKKRALENERLLTPELMGLSTNMVSMATGVSASRGQMEGG
201phi2-1_gp129     ............MIEQ.........KRVVRELKNRYVGN.GIIDPWLGTTSAARGAMLLG
PhiKZ_gp071+073     ..............MS.........QLGRREIDLTLLGH.TGLDPWYGTTSSARGAMFVT
PhiPA3_gp065+066    MPAIKGQFKMYEEHNL.........RRAVREIHAKLLGH.AALDPYYGTTSAARGAMFLS
RAY_gp248           ..............MSHIT........EASELSAELLGSVLCLNPTVHGDSSPRSAMFGG
Goslar_gp171        ..MHYKEKPLYESVKQAIA......EGKIIPIKQEYTGVGTGLNGLLHYNSAQRAAMFGG

                      60        70        80        90       100       110
PCH45_gp216         QAAQTLVLNNPDYPRTYTCVEQEMAKYTFAARARFHAEVIGVVNRFHTGM.TRDSFISNP
201phi2-1_gp129     QITQAPTIIGAEQRLFQTGGELRFGEHNFDVRIPEDCQILNVVRKYPTGM.GADAIRHNP
PhiKZ_gp071+073     HIGQAPEVNGNESRYFLTGAELEYAKYTHDVRFPEDCRVLHVLRKYPTGI.GKDSIRSNP
PhiPA3_gp065+066    HIGQAPVVEGNEPRRVMTGMEMRYAEYTFDVRLPTDCTILHKVRKYPTGQ.GYGAIQHNP
RAY_gp248           HAGQAVTIEGSTPRMLKTGIEYEYGQRTFKIEAPCQMLVIGVINRFVTNHVGTGGVKENP
Goslar_gp171        HLSQALVIKDAKPSRLTTGIEYQYGQNTWSVEFPVESRIIDIIPRFQPGHTA.NSIKTNT

                      120       130        140       150       160       170
PCH45_gp216         YSVAIVRNLEKRG..NHFDLIDIPSYYSYHNNFGYMTVKKPALSKIR..RPGRPHLNKGD
201phi2-1_gp129     ETTIVYENYYDEF..KTVGIVKVPEFMSFHQTFGFKLNKAKD...VWDNIHPGAMVSKDT
PhiKZ_gp071+073     VTTIIYENYFDKY..KTIGVLHVPEYMSHHQDFGYELVKNRE...VWETIAPNEMFSKDT
PhiPA3_gp065+066    VTTLIYENYYDEY..KTIGVLHVPEYMSFHQDFGYELVKNKE...VWESLQPDQMFAKDT
RAY_gp248           EKYVIYQNLSVNTPTPTFGILCIPTYHTRNHALGFKYVMDKKAINRLYSVDGKAYIEKGV
Goslar_gp171        MHYVLYENQEDGN.RHELRLLEVPEHHIMHQQYGFRFRPTN....LFHSLRRGQIVPRYS

                         180       190       200       210       220       230
PCH45_gp216         VLVDSP.GVGPEGFYRRGLLTKLCYLTSPYVTEDGFWASYEWCDRAAATGIGEIFVTVPK
201phi2-1_gp129     ILAESA.GKSKDGHFCAGMNVNACFMSHHATIEDGFWISDEILEQFAPHAYGTAIGSCGR
PhiKZ_gp071+073     VIAQSG.AVKKDGTLGMGVNANVVFLSAAGTIEDGFVANKNFLKRMMPTSYSTAVANAGR
PhiPA3_gp065+066    VIAQSS.TVKSNGLYGMGVNANVAFMSVPGTIEDGFVVSDEFLERMSPRTYTTAVCGAGK
RAY_gp248           IFARSP.NLTEGGDYKYGRETNVAFMSLPEVEQDGMVVTESFAQAMACTKIESRIAQWGD
Goslar_gp171        RLMESPAINQETREWGYGRDCKVCFGSFYQCIEDGGVARRGVLKHFTSTGIEKRTITSGK

                         240       250       260       270       280       290
PCH45_gp216         GHYLLPINGTPDNPKFLPSLGEPIRPDGMVLCTREADPILDLWNLTPEGMREVDLTFDEP
201phi2-1_gp129     KSFLLNAYGN....KPFPDIGDRIREDGVVFAMRDLSDDLAPAEMTKRALSDIDRTFDRV
PhiKZ_gp071+073     KAFFLNMYGDDKIYKPFPDIGDVIRPDGVIFAIRDHDDDLAPAEMTPRALRTLDRTFDRA
PhiPA3_gp065+066    KAFFLNMYGDDKIYKPFPDIGEKIREDGVIFAVRDLDDDLAPAEMTPRALRTLDRTFDRA
RAY_gp248           DQVLLNLYGDDENYKAFPDIGDKIREDGMLFAVRKIMPGTGIVNLTPKALRTYDPNFDYP
Goslar_gp171        SRYPINLYGDDEVYQAIPENGERIRKDGLLAATREIDPILSVVDMMTCNLQRVDYVYDDL

                         300       310           320       330       340
PCH45_gp216         KYIEGSCRNATVINIEVYLNEQEMSQ....VQPVMLDQYGQEIPNQLMQIWQEHKRYSAD
201phi2-1_gp129     VIGEP...GAIVKDIKIYRDERQNPS....FIPSGMEP.......QLVKYYDALCMYYRE
PhiKZ_gp071+073     VIGTP...GAKVIDIDIWRDERVNPS....PTPTGMDA.......QLVKYHTHLSSYYRE
PhiPA3_gp065+066    VIGDP...GATVKDIKVYWDERQNPS....FTPSGMDG.......QLRKYYDALCTYYRE
RAY_gp248           QYAEA...GATVVNVQVNSDRMRTGRTDVQFYD.KQAERYERAAREF...SKSLRKIYD.
Goslar_gp171        TYAEP...NARVTDIDVVSDRHRRSGRQARERAIMMEEKMRPPYRQFLKYENAIGLLYTR

                     350       360         370       380         390
PCH45_gp216         IVKIAEEIEAECPPN..RRPNYSGRLSEEIERALHIMA...YKNPKPIRY.....QNHGT
201phi2-1_gp129     ILRIYNDLKKKRKDRLRISDEFNQLIVEAL...........IYLPQAEGQRKLTRMYRLE
PhiKZ_gp071+073     LLKIYRGLLARRKDDLHITEEFERLIVTAQ...........MFLPQPDNVRKLSRFYRLD
PhiPA3_gp065+066    IIKIYRGLLARRKDKLRISEEFNQLLVEAM...........IYLPQAEGQRKLTRMYRLE
RAY_gp248           ..DLYRTYGHGMILEP.EL...NRMVTDAI.SDTGGMDRKMNNQGQIISVDKGTQVYNKV
Goslar_gp171        IQDAVRKYERERVGTP.IRHHQTVMWWLNLADAEHGIDIR.NPNGQ.LRHVLPTRDYRKE

                     400       410        420
PCH45_gp216         SIPTATIRITYKYDIIPEIGSKIAGDFGDK............................GV
201phi2-1_gp129     PLDEWRIEITYESLKQPGGAYKITDFFGGK............................GV
PhiKZ_gp071+073     PLDEWRVEVTYKAQKMPAGAFKMTDFHGGN..............................
PhiPA3_gp065+066    QLDEWRVELTYESIKVPGGAYKLTDFHGGSLMVCSHVKAWAGVIAHRDSVANYLDKKKGV
RAY_gp248           PIGDWRVKITFVKRIEAGVRFKITDTHGGK............................GV
Goslar_gp171        NLDDWRVTITYTYDVVPGIGFKFTGMAGDK............................FV
```

```
                  430        440        450        460        470        480
PCH45_gp216       LCRKTPGSHMPLDMYGTQAELVSHANATINRMISVRLDDNYLGAQCILREKEI.......
201phi2-1_gp129   VCKTSPRSEMPRDEFGNIADVVIFGGSTMRRSNYGRIYEHGFGATVRDLCQRLRVEAGFD
PhiKZ_gp071+073   .........MPIDENGNRADLIIFGGSTMRRSNYGRIYEHGFGAAARDLAQRLRVEAGLD
PhiPA3_gp065+066  VCEVRPKADMPVDEFGNVVDAIIFGGSTMRRSNYGRIYEHGFGAASRDLAQRLRVEAGLP
RAY_gp248         IVNVIPDADAPTDDYGNRADVIMDDVSITKRMNLGKPTEQYINGASVYAAKIA.......
Goslar_gp171      VTDIMEDDEMPVDQWGNVADFMFDDSTVIKRMSLSRVNAPYINGVGDQIMREL.......

                                    490        500        510        520
PCH45_gp216       .............RELYNAGRWEDAFSIISRFYEVATPRAFKEKF.IPYMTTPERRKRHV
201phi2-1_gp129   RHAELNNIDFAQSKAFNDPAWIEYAFNEFQELWWIIAPTMHEIMK......EHPNHKEYV
PhiKZ_gp071+073   RHAKPTQQQLN..SVMGNTQWVDYAFKELLGFYEIIAPTMHSKMM......EHPNPAEHV
PhiPA3_gp065+066  RHGVVPEQDLN..RVCSNREWVTYAFAELQEFYYIIAPTMHEILR......EHPSPAEYV
RAY_gp248         .............RQMAASGDLDGAWNHLMSYYHAAAYEQWEMMQSPTYLDNKPNRDHHV
Goslar_gp171      .............KPVMDAGDLETAWNTLMRYYYIVSPEFVEQVV.EKYCITDEDKWDHL

                   530        540        550        560        570        580
PCH45_gp216       ESVVNGHIMLELKHNDFPNH.IYLTEAL..EAEWPYEKGPVLFTDTRGVRRKTKVPVMIG
201phi2-1_gp129   KSALRQGTTVVYTPIDDTTHLPTAMNTIINT.KFRPNYTPVTYTDPGGRVVTTKNNVLIG
PhiKZ_gp071+073   KTVLMDGFPYIYAPVDDPVDLMAAVNKLINSDKYRPHYGKVSYRDQAGKWVTTKDNVLMG
PhiPA3_gp065+066  KTVLRDGFSYIYSPVDDPVDLMSSLNCIMNS.RFCPNHTRVTYRGQDGKMVTTKDKVLVG
RAY_gp248         RWVCDHGIE.LFAPTDRRYFGAEQVRRIMKE..HDFPVTPVTYRAPDGRMVRTRDPVVIA
Goslar_gp171      RYVQRHGTE.VYIPTDSIAAGSERMENLIRE..FPLKKGPVRYRGRSGQWRTTKNDIAIG

                     590        600        610        620        630        640
PCH45_gp216       TSYIRVLEKTGHDWGAVDSPSRQAHGIAAKISHRDRHARPYRKQPYRY.GEAEIRPLIAL
201phi2-1_gp129   PLYMMLLEKIGDDWSSVASVKVQQFGLPSKLNNFDRSSTPGRESAVRSFGESETRSYNCT
PhiKZ_gp071+073   PLYMMLLEKIGEDWSAAASVKTQPFGLPSKLNNADRASTPGRETAIRSFGESETRSYNCT
PhiPA3_gp065+066  PLYMMLLEKIGEDWSAVASVKVQQFGLPSKLNNSDRSSTPGRESAIRSFGESETRSYNCT
RAY_gp248         PIYIILLEKMGEYWASCAIPKLTHFGTLSSLTQADKFALPWRNTPTRF.GESELRLFLAA
Goslar_gp171      DTYIMMLEKTANDWSAVGIPKLQAHGLPSKLSNSDKYSSPGKEQPTRYCGESEHREVTAF

                      650        660        670        680        690        700
PCH45_gp216       AGEDFAADLLDRSNNPKASEEIFERIMEADRPSDMKFVLDRKKLPVGSSVTHQYLNNAIY
201phi2-1_gp129   VGPEPTMELVDQTNNPLAHVEVVEQFLTQEKPTRIDRAVDRKKIPFGNSRPVSLLNHMMQ
PhiKZ_gp071+073   VGPGPTAEILDQTNNPLAHAAVIESWLTAEKPSSVPVAVDREKIPFGGSRPVAMFDHLLE
PhiPA3_gp065+066  VGPEATVELLDQTNNPRAHLAVINSILTADKPSNIERAVDRTKVPFGSSRPVDLLEHLLE
RAY_gp248         CRGYYANRLQSMANTPAMQKEAALMFLRHDTPMNIPNVIDETKTPLGRARPLQMYKHNQG
Goslar_gp171      AGGWFASVFMDYANNPKQVDSITESIYATDKPSAIPIATNFEKYPIGGNRALRILYHQLN

                      710        720
PCH45_gp216       TAGARFNRIIADKKGGKR..................
201phi2-1_gp129   TRGIEFKYASSPSASH....................
PhiKZ_gp071+073   CSGIALEYAPDH........................
PhiPA3_gp065+066  CRGLKFEYATTDGVQPVHTAVPIRAQQKVKSEAIEE
RAY_gp248         CRGIEFETTVLNK.......................
Goslar_gp171      VAGGQIIDADDCES......................
```

**iii**

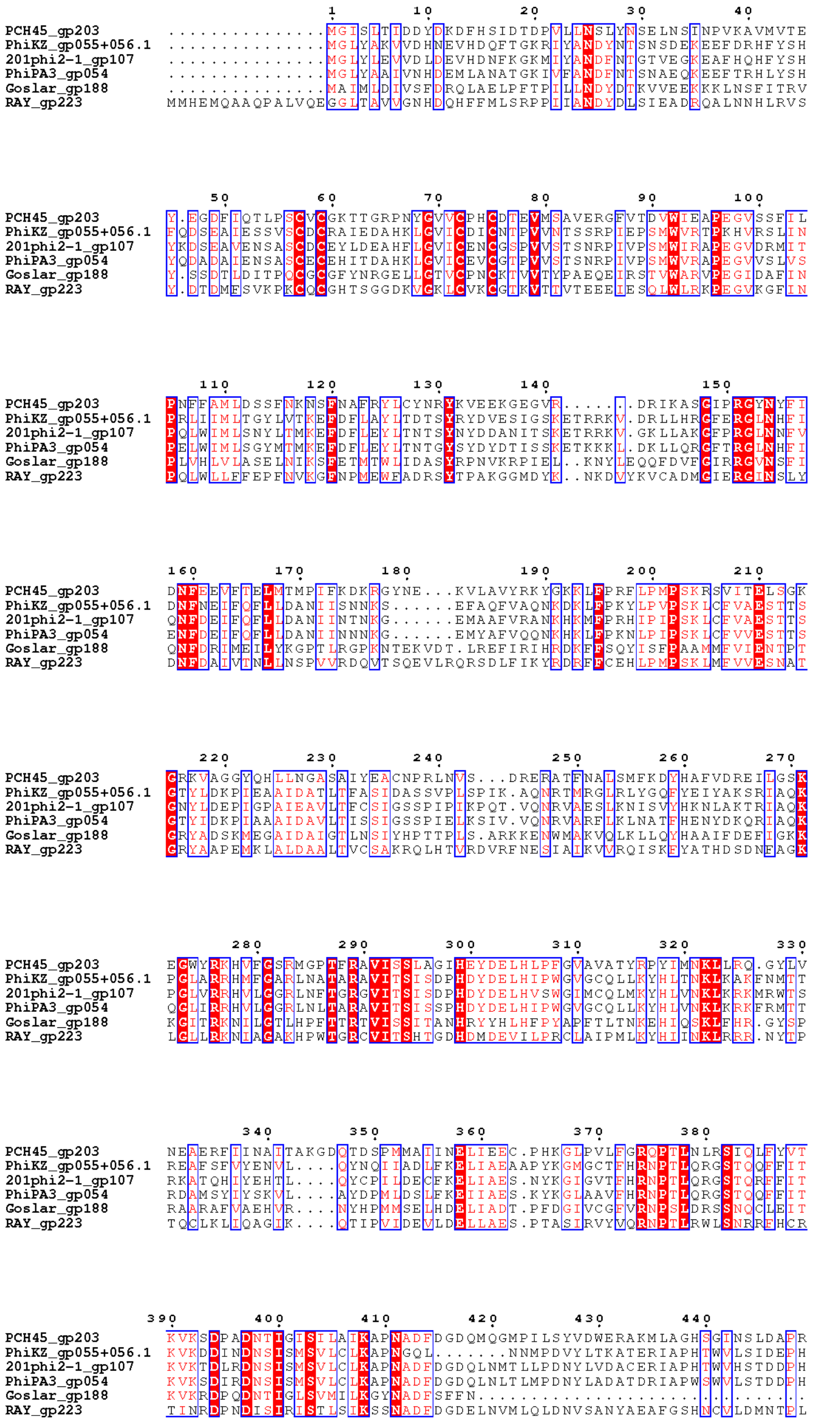
PCH45_gp203
PhiKZ_gp055+056.1
201phi2-1_gp107
PhiPA3_gp054
Goslar_gp188
RAY_gp223

```
                   450       460       470
                     .         .         .
PCH45_gp203        SLNRNLVMPDTDFSTLNNFMYGNSR...........
PhiKZ_gp055+056.1  EISGNLELQGPVVETIINWAHEKYLPPLEEWLKAK.
201phi2-1_gp107    QLSGNLELQGPVVETVVNYGHEDYLPPLEEWLAGH.
PhiPA3_gp054       EISGNLELQGPVVETIINFAHEDYLPPLEEWLKMAA
Goslar_gp188       ....................................
RAY_gp223          KISGDVGLPGTLISTMNRWMYSND............
```

iv

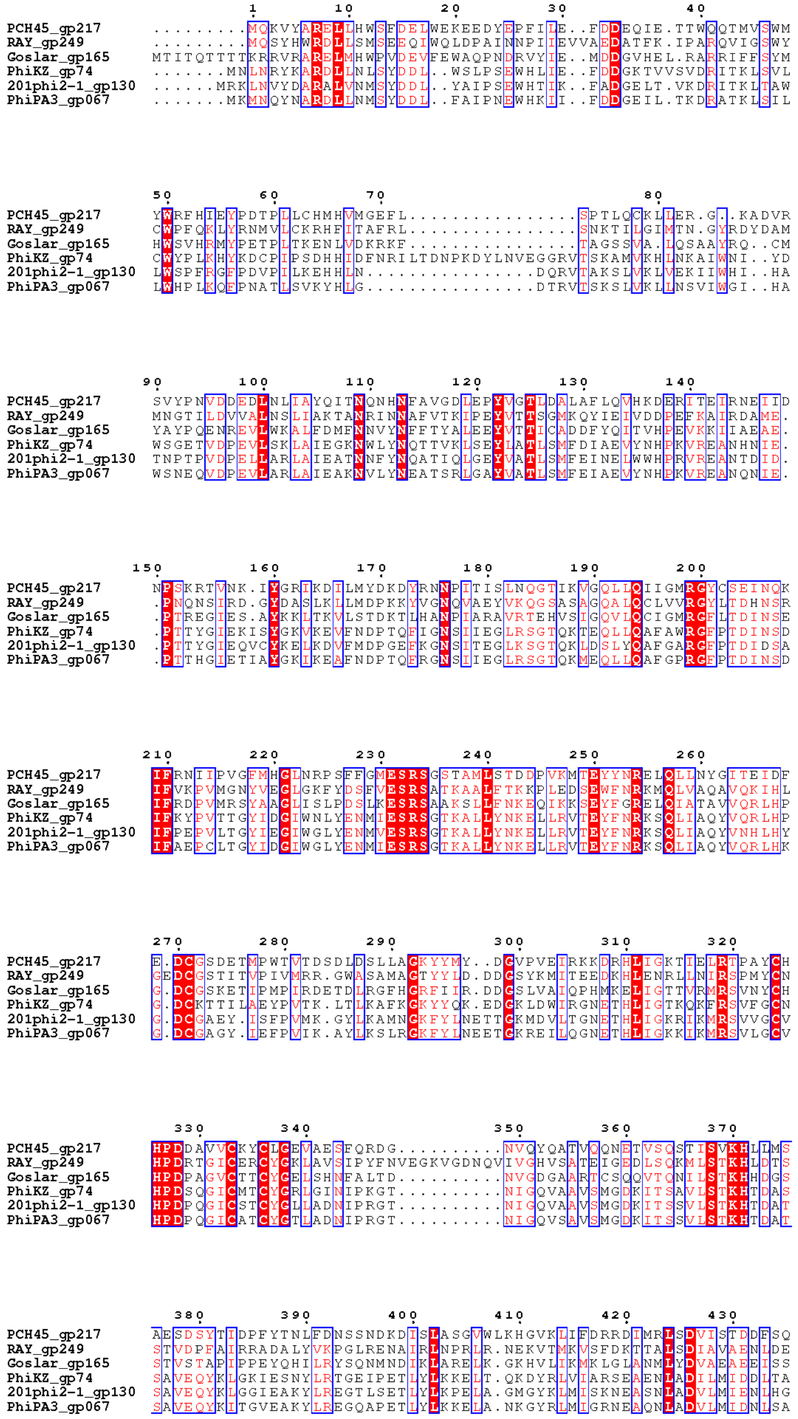
PCH45_gp217
RAY_gp249
Goslar_gp165
PhiKZ_gp74
201phi2-1_gp130
PhiPA3_gp067

```
                   440       450       460       470       480       490
PCH45_gp217        HDVSTFAKIRTVTLVYQDDHKKDPEVHSVPLNHGSYMPFLTAEFLAYIRDYGYDIV.EGN
RAY_gp249          VA.TRVSGFNEIVLEF.EREDGGKESIPINTTQGSRQGQFTVDFLRYLQRVSWTSADKDY
Goslar_gp165       LIPQRLFNMTLCDMEIYDRKDESYRKLKVNFDFCGRPVVPSKAFIRYLKRHSWEVTSDGI
PhiKZ_gp74         YPATSATELTSLALVY.DDEVNGECGDVLTVSLYNRRASLSIEMLKHIKMVRWELDQRDN
201phi2-1_gp130    YPPSSATEMTKIGLVR.QVD.GVDVGDVLTVSLYNRKASLSLEVLKHVKKVQWQPDDRGN
PhiPA3_gp067       YPPTSASELTRIGLVR.TVD.GIDEGDVLTVSLYNRKASLSIELLQHVKRVRWELDNRDN

                     500       510       520       530                540
PCH45_gp217        IEVSLAHWPEGEVIFRLPERRSTVLEAAIMLKKEIFAIGDEAK..........EARVRLRM
RAY_gp249          ISIRLDQFEYDCDVVELPLVHEDMMAYQKQIESYIRFSKESANWKNKFVTPDEV......
Goslar_gp165       VIFDLKHWDNSRTLFQMPLIHKNMMEYAKEIEREFKFGKGSFS..AGFLSEDEATPE...
PhiKZ_gp74         IVISLRGFDFNLPFLTLPNKHVNMYEVMKRFQSFLHSGSDSAE..AGKLSTEKVGYTSKT
201phi2-1_gp130    IVIDLDGFDHTQPFLTLPYKHVNMYEVMKRIQSFLHSGSDT.E..GSKLSSDKVGFTSKT
PhiPA3_gp067       IVIDLNGFDFSLPFLTLPYKHVNMYEVMKRIQSFLHSGSDT.E..GSKLSSDKVGFTSKT

                   550       560       570       580       590       600
PCH45_gp217        NLRDPMLMAKAIRDIAELTNARFHVSLPIIELVMLAMMARDPENGDFRLPRKGTGARFAP
RAY_gp249          ........GVVLDEFFSLLRQRLGVNIVHAQIMLYSVMTMDPAKGDYRLPRAYEPRQFSS
Goslar_gp165       ......EVANVLYYWHRLCAQRLRMNLSHLDVILYASMIRSPHTKDYRMPKQGTTRYFST
PhiKZ_gp74         YLKNYKSPIEALPVFATMANEKISLNISHCEILIYAMMIRSAQYRDYRLPKPGINGQFEK
201phi2-1_gp130    YLKNYENVIEGVVATASLINEKINLPLVHCEVLAYAMTIKSAQRKDYNLPKPGYLGQFEK
PhiPA3_gp067       YLKNYNDPIDAVAAFASLVNEKIQLPMPHCEVLVYAMMVRSTQQRDYRLPKPGISGQFEK

                   610       620       630       640       650       660
PCH45_gp217        QRAIMNGRSLGGKAPYERQYEMVVSPDSYLNEDRPDHPMDELLVPLGRRSSKWRPRAPIE
RAY_gp249          YHECIEYRSLSVQLVYQEQAAVMLKPSTFLNDKRQPHPMDEIFK................
Goslar_gp165       FKKNMNMRSLSMKFAHQEQLDAFQNPNSYIPTMKPDHPIDYMLLPRKEPKP....EDVIT
PhiKZ_gp74         YNRLMQCRSLGGAMAFEKQHEPLNNPGSFLNKMRNDHPYDLLVKGGKLR...........
201phi2-1_gp130    YNKLMHSRDLAGTMAFEKQHEPFANPASFLYTERNDHPYGLMVRGGQLN...........
PhiPA3_gp067       YNKLMQSRSLAGAMAFEKQHEPLNNPGSFLYTLRNDHPYDLAVKGGKLY...........

PCH45_gp217        KS
RAY_gp249          ..
Goslar_gp165       L.
PhiKZ_gp74         ..
201phi2-1_gp130    ..
PhiPA3_gp067       ..
```

E

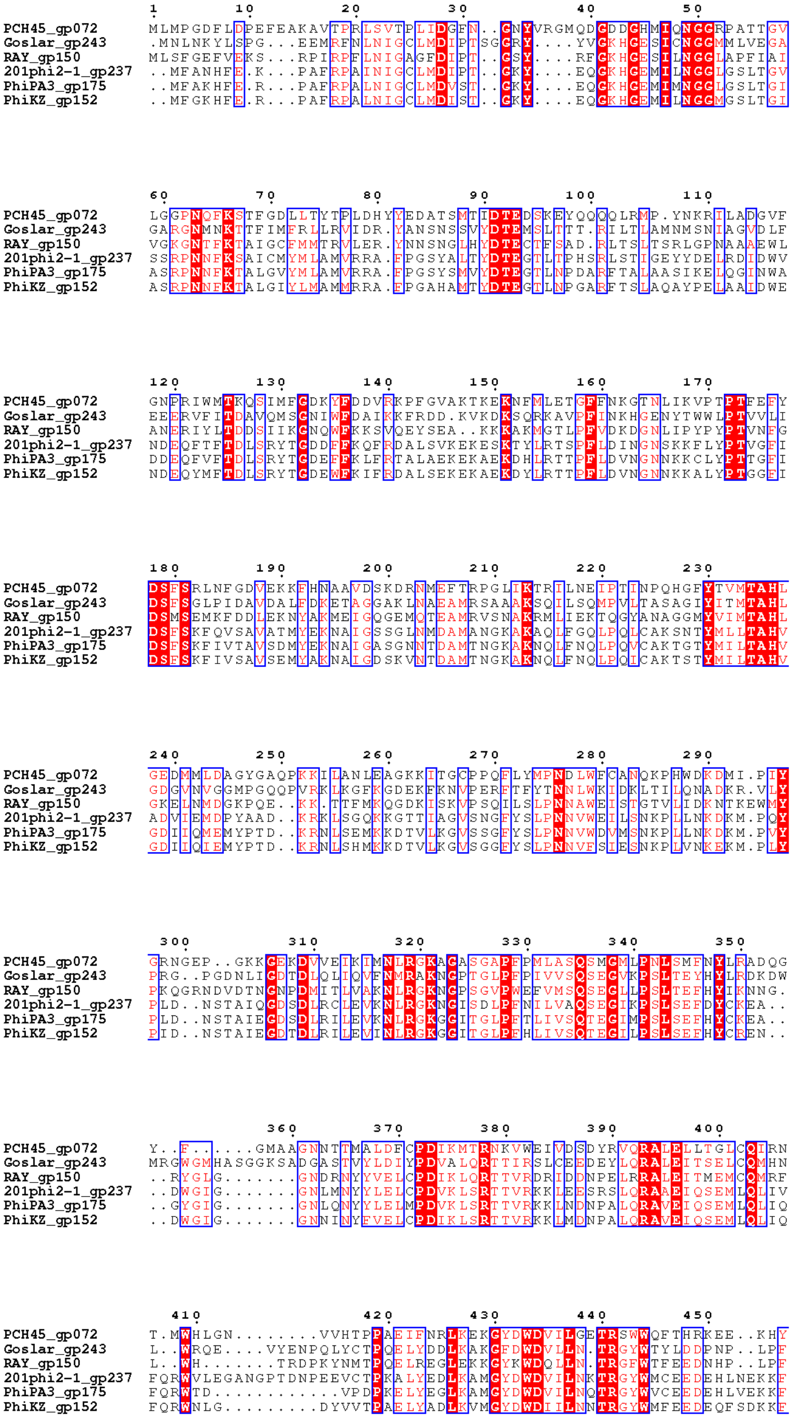
PCH45_gp072
Goslar_gp243
RAY_gp150
201phi2-1_gp237
PhiPA3_gp175
PhiKZ_gp152

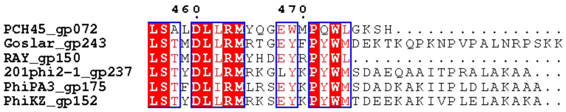
460 470
PCH45_gp072 LSALDLLRMYQGEWMPQWLGKSH...................
Goslar_gp243 LSTMDLLRMRTGEYFPYWMDEKTKQPKNPVPALNRPSKK
RAY_gp150 LSTMDLLRMYHDEYRPYWL....................
201phi2-1_gp237 LSTYDLLRMRKGLYKPYWMSDAEQAAITPRALAKAA...
PhiPA3_gp175 LSTFDLIRMLRSEYKPYWMSDADKAKIIPLDLAKAAA..
PhiKZ_gp152 LSTYDLLRMRKSEYKPYWMTDEEKAKIVPLELAKAKA..

F

```
Goslar_gp241  ............................................................
RAY_gp153     MSDNARVLPDPREDGKTHINVYSRGASWLGQQLSNMSYYDFAHPRYGVFASLEGFWYWLS
AH06_gp160    .MSDVRVLPDPREDGKSHMNVYSRGATWLGQQLSNMAYYNFAHPKYGVFASLEGFWYWLA

Goslar_gp241  ............................................................
RAY_gp153     TGKQHEELRKLAGVKAKMTGREFETIPNENFEEEFKEAMRLRLEQHPIIANALAESLLPL
AH06_gp160    TGKQHEELRNLAGVKAKMVGRDFEAIPLDTFEEEFKEAMRLRLEQHPIIANALAESILPL

Goslar_gp241  ............................................................
RAY_gp153     KHYYCYGGKVIDLYDRHKWQMDFYEEWRKANAPEDTTLVLLISGSRKEKDYDAFKHIVMT
AH06_gp160    EHYYCYGGKVIDLYERHKWQMEFYEQWRKENAPEDHSIVLLISGSRKEKDYDSFKNIVMT

                1       10        20        30        40        50
Goslar_gp241  ..MNQMLHNLPHDKVHLIAGGARSGADLHVRRYAKDYGFKYTTFPADWDGPYRKGAGFRR
RAY_gp153     YLQPYIDKIKDTYKITLLSGLAWEGPDDMAIRLCREEGFMLIGLPAKWKEQ.GKAAGMIR
AH06_gp160    YLQPYIERNKDTYKITLLSGLAWEGPDDMAIRLCREEGFMLVGLPAKWKEQ.GKAAGMIR

              60        70        80        90       100
Goslar_gp241  NEWMGDVITHLIAFWDFKSPGTKHMIDIANREDKNIIVQVFRVFPQQTRQ....
RAY_gp153     NGAMGRLCNKALVFWDGESPGTKGMIDYLKKNTIDHLVYLHGKHEPDWKAPETS
AH06_gp160    NGAMGRLCNKALVFWDGESPGTKGMIDYLKKNTIDHLVYMHGKHEPDWKAPEAA
```

G

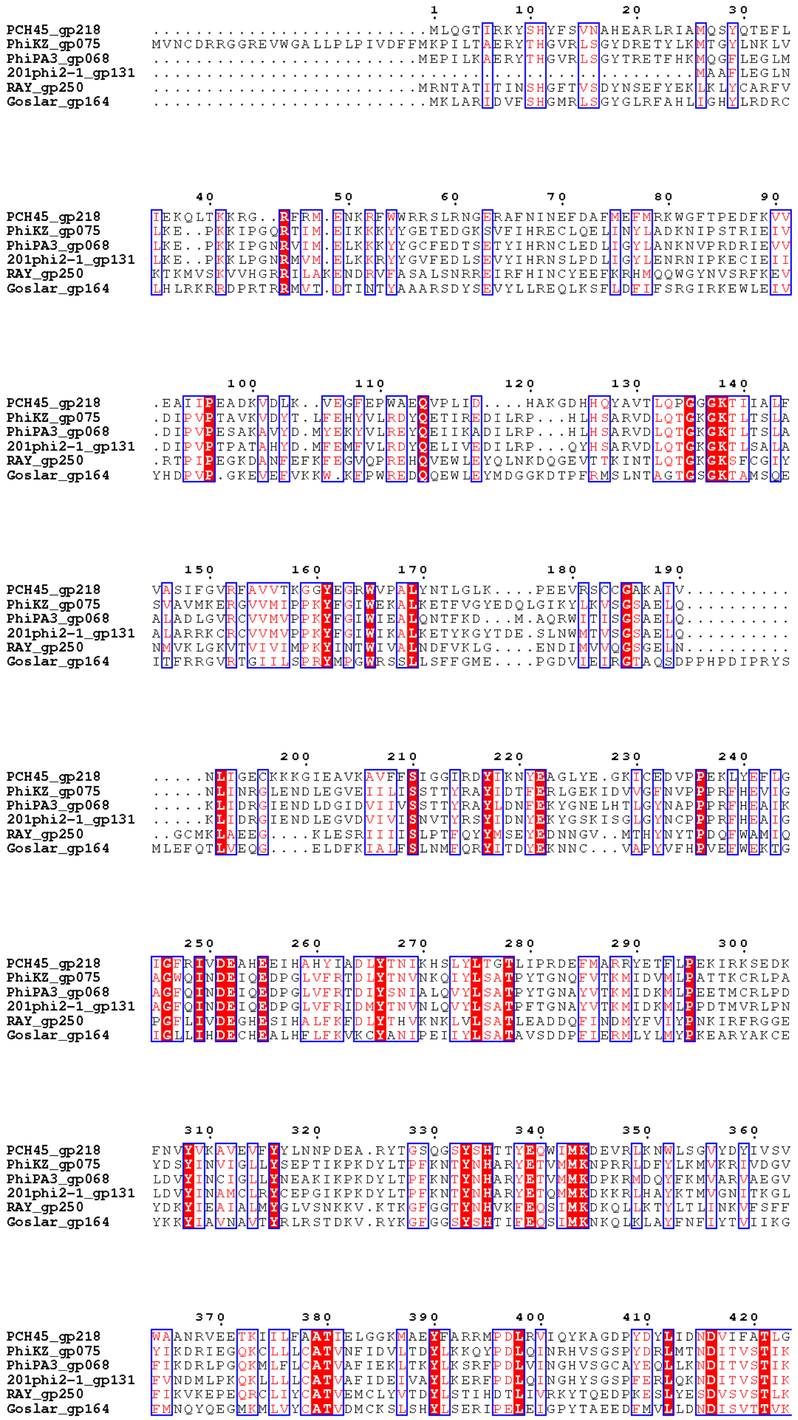
PCH45_gp218
PhiKZ_gp075
PhiPA3_gp068
201phi2-1_gp131
RAY_gp250
Goslar_gp164

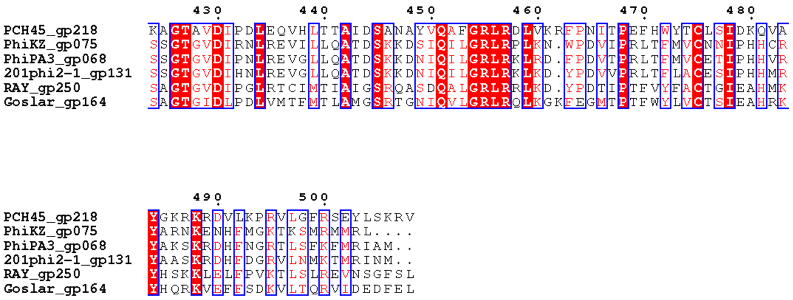
430 440 450 460 470 480
PCH45_gp218 KAGTAVDIPDLEQVHLTTAIDSANAYVQAFGRLRDLVKRFPNITPEFHWYTCLSIDKQVA
PhiKZ_gp075 SSGTGVDIRNLREVILLQATDSKKDSIQILGRLRPLKN.WPDVIPRLTFMVCNNIPHHCR
PhiPA3_gp068 SSGTGVDIPNLREVGLLQATDSKKDNIQILGRLRKLRD.FPDVTPRLTFMVCETIPHHVR
201phi2-1_gp131 SSGTGVDIHNLREVGLLQATDSKKDNIQILGRLRKLKD.YPDVVPRLTFLACESIPHHMR
RAY_gp250 SAGTGVDIPGLRTCIMTIAIGSRQASDQALGRLRRLKD.YPDTIPTFVYFACTGIEAHMK
Goslar_gp164 SAGTGIDLPDLVMTFMTLAMGSRTGNIQVLGRLRQLKGKFEGMTPTFWYLVCTSIEAHRK
490 500
PCH45_gp218 YGKRKRDVLKPRVLGFRSEYLSKRV
PhiKZ_gp075 YARNKENHFMGKTKSMRMMRL....
PhiPA3_gp068 YAKSKRDHFNGRTLSFKFMRIAM..
201phi2-1_gp131 YAASKRDHFDGRVLNMKTMRINM..
RAY_gp250 YHSKKLELFPVKTLSLREVNSGFSL
Goslar_gp164 YHQRKVEFFSDKVLTQRVIDEDFEL

**H**

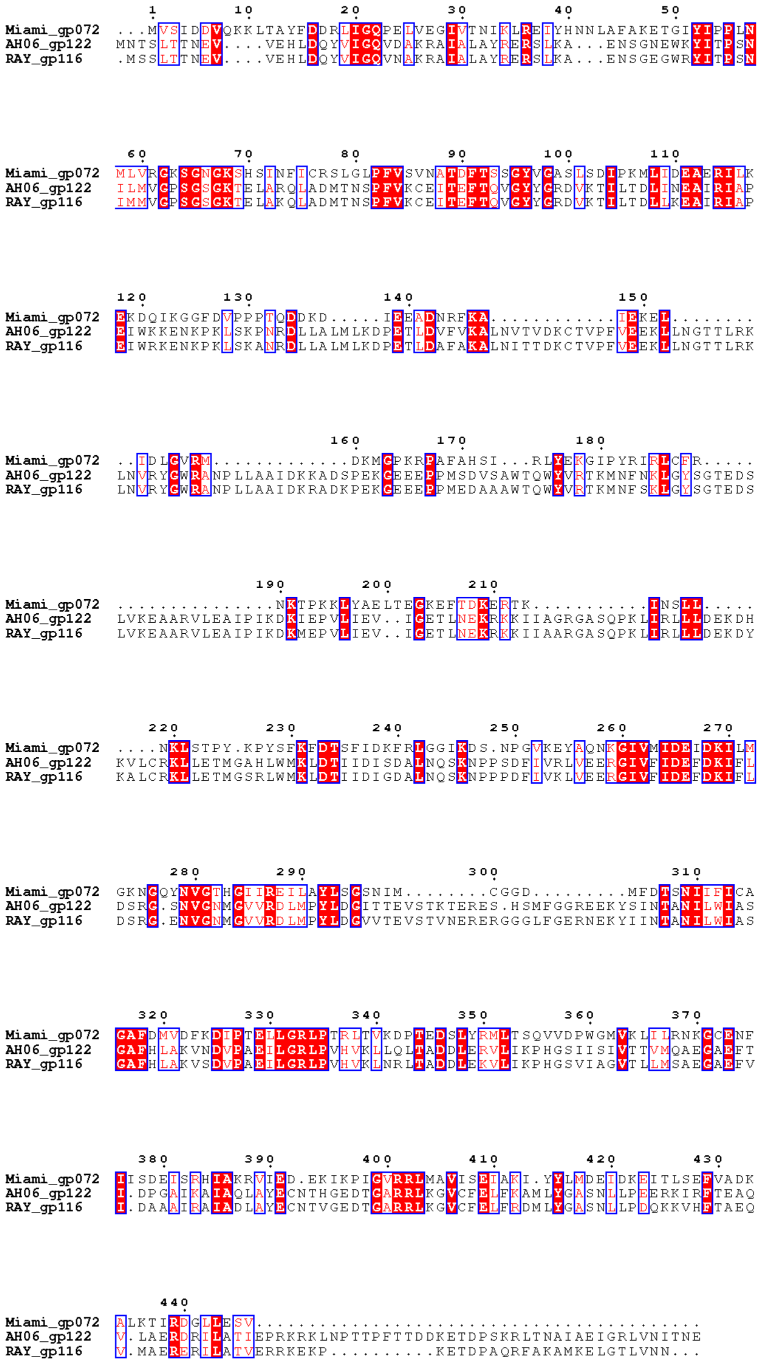
Miami_gp072 ...MVSIDDVQKKLTAYFDDRLIGQPELVEGIVTNIKLREIYHNNLAFAKETGIYIPPLN
AH06_gp122 MNTSLTTNEV....VEHLDQYVIGQVDAKRAIALAYRERSLKA...ENSGNEWKYITPSN
RAY_gp116 .MSSLTTNEV....VEHLDQYVIGQVNAKRAIALAYRERSLKA...ENSGEGWRYITPSN
Miami_gp072 MLVRCKSGNGKSHSINFICRSLGLPFVSVNATDFTSSGYVCASLSDIPKMLIDEAERILK
AH06_gp122 ILMVGPSGSGKTELARQLADMTNSPFVKCEITEFTQVGYYGRDVKTILTDLINEAIRIAP
RAY_gp116 IMMVGPSGSGKTELAKQLADMTNSPFVKCEITEFTQVGYYGRDVKTILTDLLKEAIRIAP
Miami_gp072 EKDQIKGGFDVPPPTQDDKD.....IEEADNRFKA............IEKEL........
AH06_gp122 EIWKKENKPKLSKPNRDLLALMLKDPETLDVFVKALNVTVDKCTVPFVEEKLLNGTTLRK
RAY_gp116 EIWRKENKPKLSKANRDLLALMLKDPETLDAFAKALNITTDKCTVPFVEEKLLNGTTLRK
Miami_gp072 ..IDLGVRM.............DKMGPKRPAFAHSI...RLYEKGIPYRIRLCFR.....
AH06_gp122 LNVRYGWRANPLLAAIDKKADSPEKGEEEPPMSDVSAWTQWYVRTKMNFNKLGYSGTEDS
RAY_gp116 LNVRYGWRANPLLAAIDKRADKPEKGEEEPPMEDAAAWTQWYVRTKMNFSKLGYSGTEDS
Miami_gp072 ...............NKTPKKLYAELTEGKEFTDKERTK...........INSLL.....
AH06_gp122 LVKEAARVLEAIPIKDKIEPVLIEV..IGETLNEKRKKIIAGRGASQPKLIRLLLDEKDH
RAY_gp116 LVKEAARVLEAIPIKDKMEPVLIEV..IGETLNEKRKKIIAARGASQPKLIRLLLDEKDY
Miami_gp072 ....NKLSTPY.KPYSFKFDTSFIDKFRLGGIKDS.NPGVKEYAQNKGIVMIDEIDKILM
AH06_gp122 KVLCRKLLETMGAHLWMKLDTIIDISDALNQSKNPPSDFIVRLVEERGIVFIDEFDKIFL
RAY_gp116 KALCRKLLETMGSRLWMKLDTIIDIGDALNQSKNPPPDFIVKLVEERGIVFIDEFDKIFL
Miami_gp072 GKNGQYNVGTHGIIREILAYISGSNIM........CGGD.........MFDTSNIIFICA
AH06_gp122 DSRG.SNVGNMGVVRDLMPYLDGITTEVSTKTERES.HSMFGGREEKYSINTANILWIAS
RAY_gp116 DSRG.ENVGNMGVVRDLMPYLDGVVTEVSTVNERERGGGLFGERNEKYIINTANILWIAS
Miami_gp072 GAFDMVDFKDIPTELLGRLPTRLTVKDPTEDSLYRMLTSQVVDPWGMVKLILRNKGCENF
AH06_gp122 GAFHLAKVNDVPAEHLGRLPVHVKLLQLTADDLERVLIKPHGSIISIVTTVMQAEGAEFT
RAY_gp116 GAFHLAKVSDVPAEHLGRLPVHVKLNRLTADDLEKVLIKPHGSVIAGVTLLMSAEGAEFV
Miami_gp072 IISDEISRHIAKRVIED.EKIKPIGVRRLMAVISEIAKI.YYLMDEIDKEITLSEFVADK
AH06_gp122 I.DPGAIKAIAQLAYECNTHGEDTGARRLKGVCFELFKAMLYGASNLLPEERKIRFTEAQ
RAY_gp116 I.DAAAIRAIADLAYECNTVGEDTGARRLKGVCFELFRDMLYGASNLLPDQKKVHFTAEQ
Miami_gp072 ALKTIRDGLLESV.......................................
AH06_gp122 V.LAERDRILATIEPRKRKLNPTTPFTTDDKETDPSKRLTNAIAEIGRLVNITNE
RAY_gp116 V.MAERERILATVERRKEKP..........KETDPAQRFAKAMKELGTLVNN...

**I**

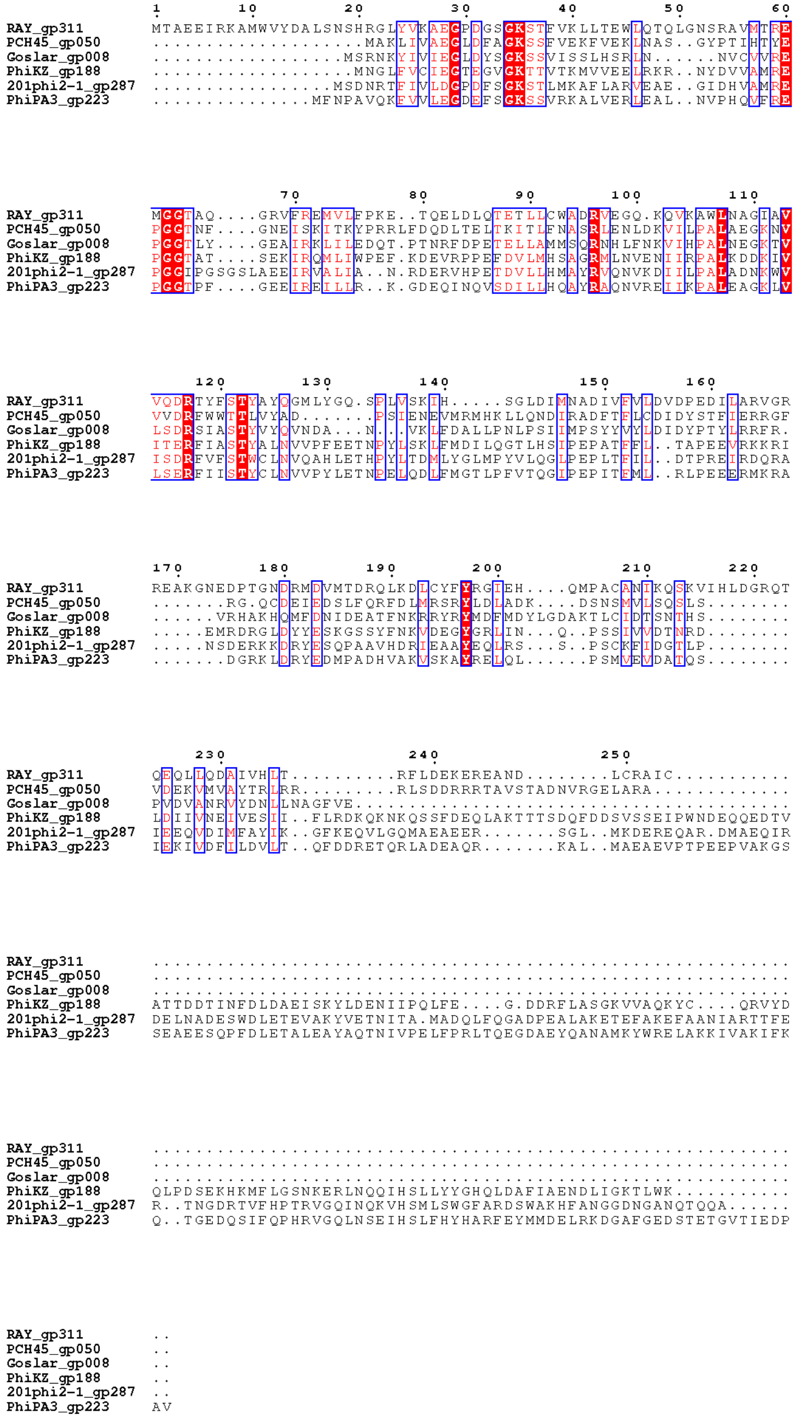
1 10 20 30 40 50 60
RAY_gp311 MTAEEIRKAMWVYDALSNSHRGLYVKAEGPDGSGKSTFVKLLTEWLQTQLGNSRAVMTRE
PCH45_gp050 ..................MAKLIVAEGLDFAGKSSFVEKFVEKLNAS..GYPTIHTYE
Goslar_gp008 ..................MSRNKYIVIEGLDYSGKSSVISSLHSRLN......NVCVVRE
PhiKZ_gp188 ..................MNGLFVCIEGTEGVGKTTVTKMVVEELRKR..NYDVVAMRE
201phi2-1_gp287 ................MSDNRTFIVLDGPDFSGKSTLMKAFLARVEAE..GIDHVAMRE
PhiPA3_gp223 ..............MFNPAVQKFVVLEGDEFSGKSSVRKALVERLEAL..NVPHQVFRE
70 80 90 100 110
RAY_gp311 MGGTAQ....GRVFREMVLFPKE..TQELDLQTETLLCWADRVEGQ.KQVKAWLNAGIAV
PCH45_gp050 PGGTNF....GNEISKITKYPRRLFDQDLTELTKITLFNASRLENLDKVILPALAEGKNV
Goslar_gp008 PGGTLY....GEAIRKLILEDQT.PTNRFDPETELLAMMSQRNHLFNKVIHPALNEGKTV
PhiKZ_gp188 PGGTAT....SEKIRQMLIWPEF.KDEVRPPEFDVLMHSAGRMLNVENIIRPALKDDKIV
201phi2-1_gp287 PGGIPGSGSLAEEIRVALIA..N.RDERVHPETDVLLHMAYRVQNVKDIILPALADNKWV
PhiPA3_gp223 PGGTPF....GEEIREILLR..K.GDEQINQVSDILLHQAYRAQNVREIIKPALEAGKLV
120 130 140 150 160
RAY_gp311 VQDRTYFSTYAYQGMLYGQ.SPLVSKIH.....SGLDIMNADIVFVLDVDPEDILARVGR
PCH45_gp050 VVDRFWWTTLVYAD......PSIENEVMRMHKLLQNDIRADFTFLCDIDYSTFIERRGF
Goslar_gp008 LSDRSIASTYVYQVNDA...N...VKLFDALLPNLPSIIMPSYYVYLDIDYPTYLRRFR.
PhiKZ_gp188 ITERFIASTYALNVVPFEETNPYLSKLFMDILQGTLHSIPEPATFFL..TAPEEVRKKRI
201phi2-1_gp287 ISDRFVFSTWCLNVQAHLETHPYLTDMLYGLMPYVLQGLPEPLTFIL..DTPREIRDQRA
PhiPA3_gp223 LSERFIISTYCLNVVPYLETNPELQDLFMGTLPFVTQGIPEPITEML..RLPEEERMKRA
170 180 190 200 210 220
RAY_gp311 REAKGNEDPTGNDRMDVMTDRQLKDLCYFYRGIEH....QMPACANIKQSKVIHLDGRQT
PCH45_gp050 .......RG.QCDEIEDSLFQRFDLMRSRYLDLADK....DSNSMVLSQSLS........
Goslar_gp008 ......VRHAKHQMFDNIDEATFNKRRYRYMDFMDYLGDAKTLCIDTSNTHS........
PhiKZ_gp188 .....EMRDRGLDYYESKGSSYFNKVDEGYGRLIN...Q..PSSIVVDTNRD........
201phi2-1_gp287 .....NSDERKKDRYESQPAAVHDRIEAAYEQLRS...S..PSCKFIDGTLP........
PhiPA3_gp223 .......DGRKLDRYEDMPADHVAKVSKAYRELQL......PSMVEVDATQS........
230 240 250
RAY_gp311 QEQLLQDAIVHLT..........RFLDEKEREAND........LCRAIC...........
PCH45_gp050 VDEKVMVAYTRLRR.........RLSDDRRRTAVSTADNVRGELARA.............
Goslar_gp008 PVDVANRVYDNLLNAGFVE.........................................
PhiKZ_gp188 LDIIVNEIVESII..FLRDKQKNKQSSFDEQLAKTTTSDQFDDSVSSEIPWNDEQQEDTV
201phi2-1_gp287 IEEQVDIMFAYIK..GFKEQVLGQMAEAEER.......SGL..MKDEREQAR.DMAEQIR
PhiPA3_gp223 IEKIVDFILDVLT..QFDDRETQRLADEAQR.......KAL..MAEAEVPTPEEPVAKGS
RAY_gp311 ............................................................
PCH45_gp050 ............................................................
Goslar_gp008 ............................................................
PhiKZ_gp188 ATTDDTINFDLDAEISKYLDENIIPQLFE....G.DDRFLASGKVVAQKYC....QRVYD
201phi2-1_gp287 DELNADESWDLETEVAKYVETNITA.MADQLFQGADPEALAKETEFAKEFAANIARTTFE
PhiPA3_gp223 SEAEESQPFDLETALEAYAQTNIVPELFPRLTQEGDAEYQANAMKYWRELAKKIVAKIFK
RAY_gp311 ............................................................
PCH45_gp050 ............................................................
Goslar_gp008 ............................................................
PhiKZ_gp188 QLPDSEKHKMFLGSNKERLNQQIHSLLYYGHQLDAFIAENDLIGKTLWK..........
201phi2-1_gp287 R..TNGDRTVFHPTRVGQINQKVHSMLSWGFARDSWAKHFANGGDNGANQTQQA......
PhiPA3_gp223 Q..TGEDQSIFQPHRVGQLNSEIHSLFHYHARFEYMMDELRKDGAFGEDSTETGVTIEDP
RAY_gp311 ..
PCH45_gp050 ..
Goslar_gp008 ..
PhiKZ_gp188 ..
201phi2-1_gp287 ..
PhiPA3_gp223 AV

**J**

```
                   1       10        20        30        40
201phi2-1_gp347  .......MSIQRITFEGVLNWLSTKQSDKWHMVFQASQFD..LNDF....VDSVSFGHLT
PhiKZ_gp232      MLKVKS..LLTGHILRATYDWFLENNFRDILIVAHT.QLVPDLSEVALKH..AKEDHTIT
PhiPA3_gp270     MITLKK..SLTQCTLDSWYTFMAENSLSRFDCLIDIGYLDEPLRKILEKHPMYRGDGTIL
AH06_gp038       MSENKKIIPLVHFQIDAFRNFLIANGFTPYAVFALPKGLDPVLDQ.....FIN..DGQII
RAY_gp039        MSEQKPLIPFLHFQIDAFRNYLVANDLTPYAVFTLPKGLDPVLDR.....YADPASGQLI

                 50        60        70        80        90        100
201phi2-1_gp347  LNLSPNATRDM.ALYDEHIYFKICKHGIPQEMMIPYTALMIIQDPDDPSGSMP..WPYFL
PhiKZ_gp232      FNIHP.RCAKNFYISDEYISFNVTVNGVGVSIKLPLYAVLGVVTPIDDNSNAFFEMPLVD
PhiPA3_gp270     LNLSVNACKHINYRGDAFTG.EIGLQGRPTSLYIPYHAFVSLQIPLSETATVEASFPIYD
AH06_gp038       LNVGLTACGHYEITDDGYMVMEQRFNGKPHRSFVPVKYLLAMYARE..DVKHAMMFPDLA
RAY_gp039        LNLSPNSCGHYEITDDGYMVLHQRFSGKAHEAFLPVQHLLAMYARE..RQDQAMVFADIE

                   110       120            130       140       150       160
201phi2-1_gp347  DHGEDYTPDEDEELDQGT...VVKKSNVIELPNSGVKLELRIPTLEDYNNLNFDNVLQFP
PhiKZ_gp232      RYLNA...DTIRATLEGTVNNVVDKNPVL.............TTVGNVTEVNFKNKADT.
PhiPA3_gp270     RLVFD...AQNPTFV.............................................
AH06_gp038       ELDV........................................................
RAY_gp039        HTCD........................................................

                      170       180       190            200       210
201phi2-1_gp347  KKDADGELPSAEEIA.ELNQLMSDNGMDINLVEFKRN.....DKGEFEVSLSLNDNPVVE
PhiKZ_gp232      ...TNGKLVVRNTSENEHDQMI.ESAIN.RVTEFSNPELDAHIRVAVTVIKQNAPELIMD
PhiPA3_gp270     ...................................HPYAEA....GQQSLLTIAPEAVVK
AH06_gp038       ........................................S....G..............
RAY_gp039        .............................................D..............

                  220              230
201phi2-1_gp347  WDGKDKHLKQLSDN........LPKG...............LVSD................
PhiKZ_gp232      WCERVIN..CTRN..................ENYQDNINVLMMELLSYPISNVVSESTVD
PhiPA3_gp270     WMHNEGHTECNVHNVGEFIKYVWPNGVPMEVQEYLVEETANMLRDHQMAVLQKQSAQAPV
AH06_gp038       ......................................ETEMLPDDQVVMQA.DVEAEAA
RAY_gp039        ......................................DVKFVEDETVPEV........D

                           240       250       260       270       280
201phi2-1_gp347  ...........DGIIDVVKLASKAQRLALAAPPTLQQRMAERGMSVIIGGAKPAEASMPF
PhiKZ_gp232      SGDNTSNSFDNDVNKMVEDFNNSSNSQVIINKP..SRPTGTPKLTVIKGGKK........
PhiPA3_gp270     PSNDDRIPFEEAEIKPLLEFPDLSANKVVLQQPVASKFKGKPTLTLIKGGKK........
AH06_gp038       PSNVTPL.RRG..............................PSLSIVK............
RAY_gp039        DTNVTALPFKK..............................PTLTVVK............

                 290       300       310       320       330       340
201phi2-1_gp347  IDEVYRAKRERREAIQKAADEALFKERALPAGTTLGEMLQSPGMTIRSDGSKGNSVFFPD
PhiKZ_gp232      ............................................................
PhiPA3_gp270     ............................................................
AH06_gp038       ............................................................
RAY_gp039        ............................................................

                 350       360       370
201phi2-1_gp347  LDVRKCYFHTRRIVRPEWLQVHEGGLK
PhiKZ_gp232      ...........................
PhiPA3_gp270     ...........................
AH06_gp038       ...........................
RAY_gp039        ...........................
```

K

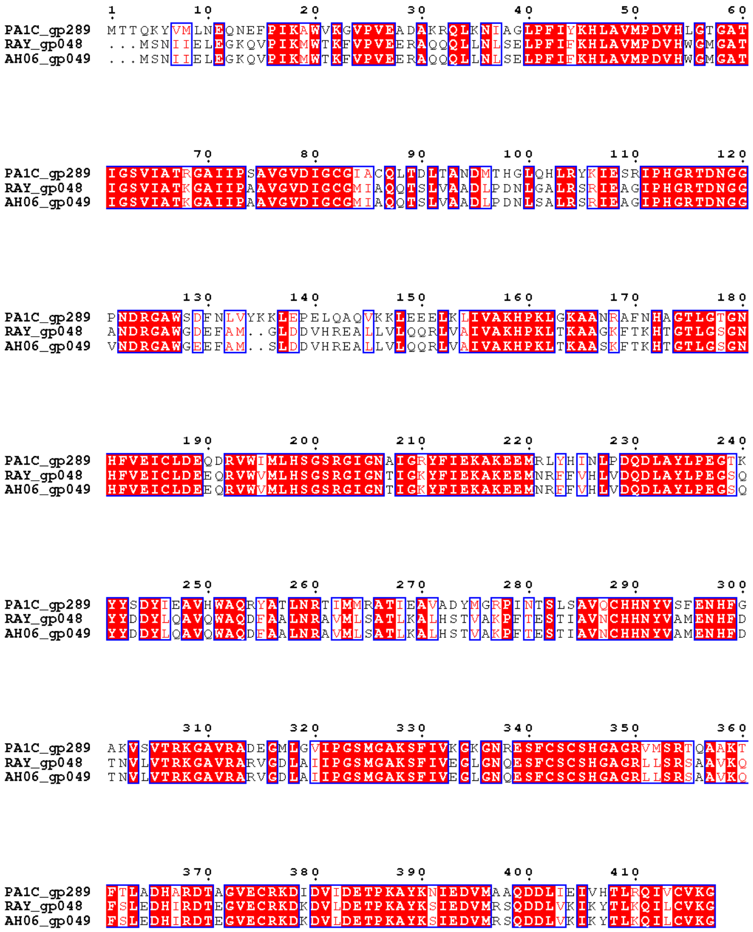

**L**

```
                1        10        20        30        40        50        60
Goslar_gp147    MALTTTIDNKELNPAETLMYAAIATLPNLDVEKLFKVHGDDLLNIIRDQIDPITPEEIKG
RAY_gp064       ............................................................
AH06_gp067      ............................................................

                         70        80        90       100       110       120
Goslar_gp147    NKELHEEIKRGEYYTYHGFMTLFQEDTSHLHEITLRHYLRCAIFQLMNHREETHKALAQS
RAY_gp064       ............................................................
AH06_gp067      ............................................................

                        130       140       150       160       170       180
Goslar_gp147    FQTHIFEAPYKPLDIVHLFYSISCCVNDQVMLLIQQNDRELIPLLSKLGIDLVAENITFS
RAY_gp064       ...........................................MITPLSLLY........
AH06_gp067      ............................................................

                        190       200       210       220       230       240
Goslar_gp147    QFIRETFLGAEPAPRYEGRFDLSTVEKPAFCLDVENLSLEKNGVLLEIGIVFFDLANPES
RAY_gp064       ..VKESYMI...................VVVVDTESTALDELAGILSLSAIRTNVADMSC
AH06_gp067      .......MI...................IGVVDAESTDLVDSAGLFTISGIIMNVTDLAD

                                   250       260       270       280
Goslar_gp147    I...........ANGPRIDIFPDVHGQIADGGRISESTVEWWQTKAPKEARDYCFQHSKV
RAY_gp064       IKHLIGVHQAGKTHDDIFHAVLNLNEQYVMGRTFLQSTQDWWMKKTTPAAKASVTGPTQ.
AH06_gp067      IEKQIADYKAGKRPEGCVHVVLNITEQYLLGRTFLASTQAWWVKKTTPAARAAILEQPT.

               290       300       310       320       330       340
Goslar_gp147    MDYKEAIAEVNEFINTIREVFTDYYGKDT.FYYLARGETDWPQLEHWFRKAGFKPVCRYN
RAY_gp064       .SAKDALTAFVAFIEKAIEATINERGEKAKVQLYYRGDFDAKAIASLAKAVGVELPYIFN
AH06_gp067      .PLKEAMELYAAFIQKLIDLSKEERGPNALVNLYYRGDFDGRAIASLAKATGVQLPSGYR

                350       360       370       380       390       400
Goslar_gp147    QVQDIRSMIAAYQDKPVSILGTKEYPDMGIPLIRHTAIGDAIMDAYDVAKARSMAASK..
RAY_gp064       QNKEIRTYIDAKLDTDIGYIPWLGYP...EGLGRHSSLDDVLIDGFEMAVAYRINHLEKF
AH06_gp067      SNKEIRTYIDAKLDTDIGYIPWLGYP...AGLERHNSLEDVLIDGFEMAVAWRINDMQKN

Goslar_gp147    ....LPK....................
RAY_gp064       DKKSVPGEIAKLKETYPVPEKTHAKSK
AH06_gp067      AAKAVEAEIAKLSKKYPPKENQHV...
```

**M**

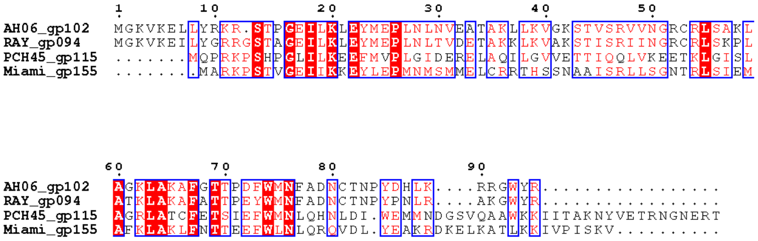
1 10 20 30 40 50
AH06_gp102 MGKVKELLYRKR.STPGEILKLEYMEPLNLNVEATAKLLKVGKSTVSRVVNGRCRLSAKL
RAY_gp094 MGKVKEILYGRRGSTAGEILKLEYMEPLNLTVDETAKKLKVAKSTISRIINGRCRLSKPL
PCH45_gp115 .......MQPRKPSHPGLILKEEFMVPLGIDERELAQILGVVETTIQQLVKEETKLGISL
Miami_gp155 ........MARKPSTVGEIIKKEYLEPMNMSMMELCRRTHSSNAAISRLLSGNTRLSIEM
60 70 80 90
AH06_gp102 AGKLAKAFGTTPDFWMNFADNCTNPYDHLK....RRGWYR.................
RAY_gp094 ATKLAKAFATTPEYWMNFADNCTNPYPNLR....AKGWYR.................
PCH45_gp115 AGRLATCFETSIEFWMNLQHNLDI.WEMMNDGSVQAAWKKIITAKNYVETRNGNERT
Miami_gp155 AFKLAKLFNTTEEFWLNLQRQVDL.YEAKRDKELKATLKKIVPISKV..........

**N**

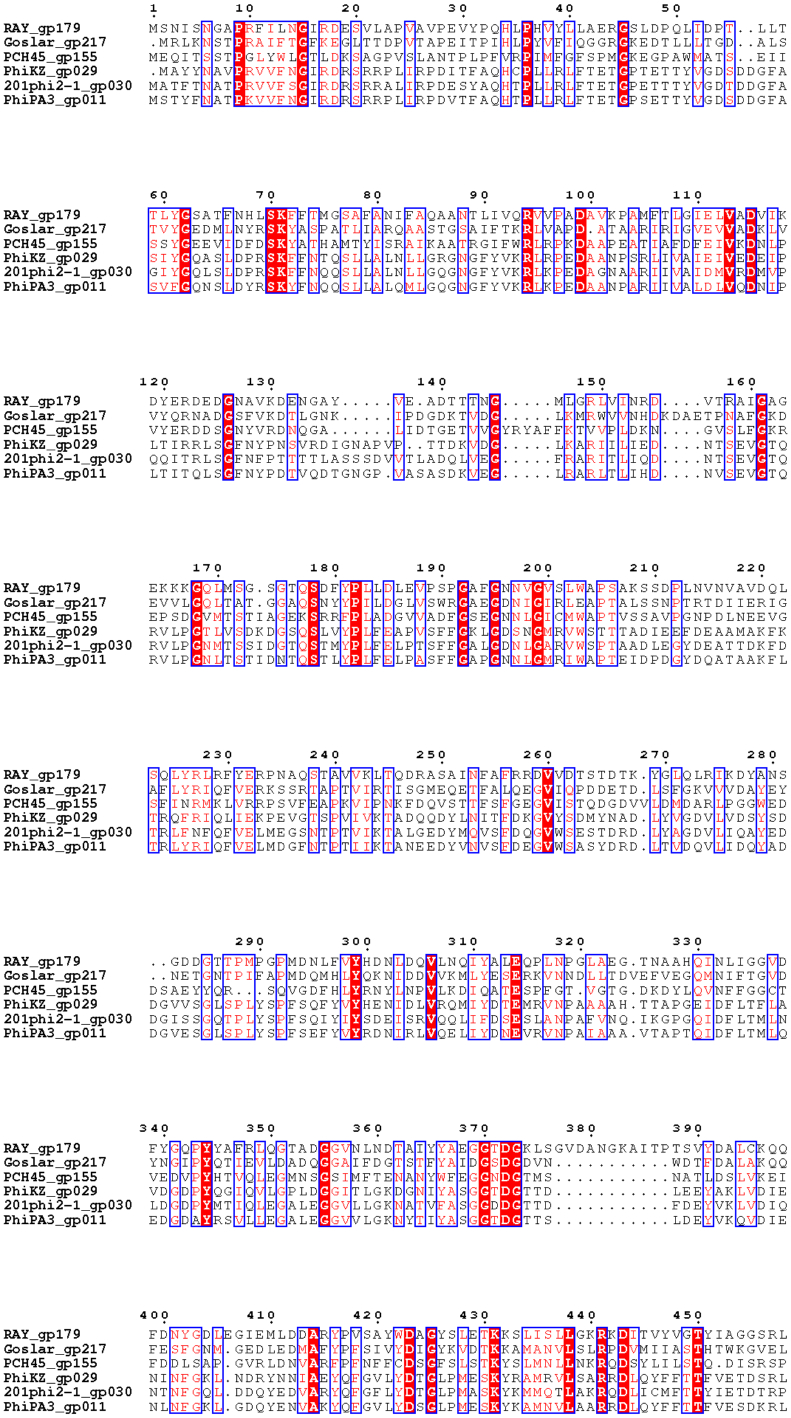

RAY_gp179
Goslar_gp217
PCH45_gp155
PhiKZ_gp029
201phi2-1_gp030
PhiPA3_gp011

```
                    460       470       480       490       500       510
RAY_gp179           SS.LEQRSMAASLRSYAMLFPESSLYGTSTCRAVFFMQSGQLASEDSDDFYPHLLDHAYK
Goslar_gp217        NV.DEESSMGAMLRNYYRLHPESVLYNTPACRAAVFMQYGESIEAPSLGKVPLTLEVANK
PCH45_gp155         NDEESEESIASSILTRLQMFPDSVEYATPPFRGALVGHVGQLAGTSRRVSAVLAIDLAYK
PhiKZ_gp029         PDEATELSRVQQIITRLKAFPESTLYGTGVCRAMIVMQSGKLMDGTYRKYVPQLLDVAMS
201phi2-1_gp030     LTTGDEVSRTIALMTRLKAFPESTLYGTPVARAQIVLQSGKLMAGGYSKRVPQLLDTAMK
PhiPA3_gp011        PTASEEVSRVQALMTRLKAFPESTLYGTPVCRAMIVMQSGRLVGGGYNKFVPQLLDVAVM

                     520       530       540       550       560       570
RAY_gp179           RARYAGSGDGVLKNAFAYDAAENN..KVQLMKNMDALWEPNEVRNTEWGLGLNYAQTYNR
Goslar_gp217        LATYMGASDGVIRGTRI.DNNPGN..VVTTMRKINKTYRNATVRDRDWENGLNYVQTYNR
PCH45_gp155         LMRYGALDSGILSPDRAPDTSDNGNYIVTEVKGLNVIDKTWRNKRRLWSQSVVYVEDYDH
PhiKZ_gp029         WARYAGAGTGNLVPGMEMDVSPNN..RVTFVKDLNVKFFDDRVRAQAWANGATWSQSYDH
201phi2-1_gp030     WAIYAGAGNGILRPGFEMDVSPNN..QVTFVKGLNVPFFNARTASNIWQNGGTYSSTYDK
PhiPA3_gp011        WAKYAGAGTGVLRPGLEMDVDPNN..RVTMVKNLNVPFFNARTSANLWANGATYSSSYDH

                        580       590       600       610       620       630
RAY_gp179           R.QAYVPHVQTVYDTESSVLRDEIALTICVDIEKQCQIAYRMLGTDTRRTTAQTLSELNR
Goslar_gp217        N.SNFFPAWGTIYNNDRSVWRSLINVIIGCDLTRVCYRLWAETTGTTGMDETLFLKLMGE
PCH45_gp155         NNRLFYPGMQSFYTDQTSVLNSAIVGLCVAQLARYAWETWRDLTGNQKLKDAQLIERSDE
PhiKZ_gp029         R.SSYYPCLRSVMLDDTSVLLSPITVNICCVLIRLIHKVHAQFSGNATLTPEQLVERCDE
201phi2-1_gp030     R.RQYYPCQRSVYLDDTSVLLSPLTVNICCVIMRLIHRVHAKFAGNATLSKEQLVARCDQ
PhiPA3_gp011        R.SSYYPCLRSVYLDDTSVLLSPVTVNICCVIMRLIHKIHAKFSGNAFLTKEQLVERCDQ

                        640       650       660       670       680       690
RAY_gp179           ILENLTKDRYDNRVVISVNSFRTIADQNSRVRYSCEVSAAFNKGMYIGSFTVVSRNQEDL
Goslar_gp217        RFLEYTTGRYNDHVTVKGRFYLDKEDGYNGFSYHGDISLIGDPGMTVGTFTITGLRRNTE
PCH45_gp155         SIKNKSAGAMDDRMDVIPHSQLTRLDQNRGYSWTCPIDIGANGMRTVMSAYTVAYRREDL
PhiKZ_gp029         YILDLVRDMFGTRVNIIPRTEITPIDANNGTSWTCNVTVEANNPRTTLNFNLETVRIETP
201phi2-1_gp030     EILDQTRDLFGARVDITPQTEITAADDQNGFSWTCTVTVAANNPRTVMDFKLETVRRETV
PhiPA3_gp011        EILDRTRDMFGGRVDIIPRTEITPIDENNGFSWTCTVTVAANNPRTVLDFRLETVRRENV

RAY_gp179           V.....
Goslar_gp217        AAEAA.
PCH45_gp155         NG....
PhiKZ_gp029         PAQQ..
201phi2-1_gp030     TAGTVQ
PhiPA3_gp011        TADQQ.
```

O

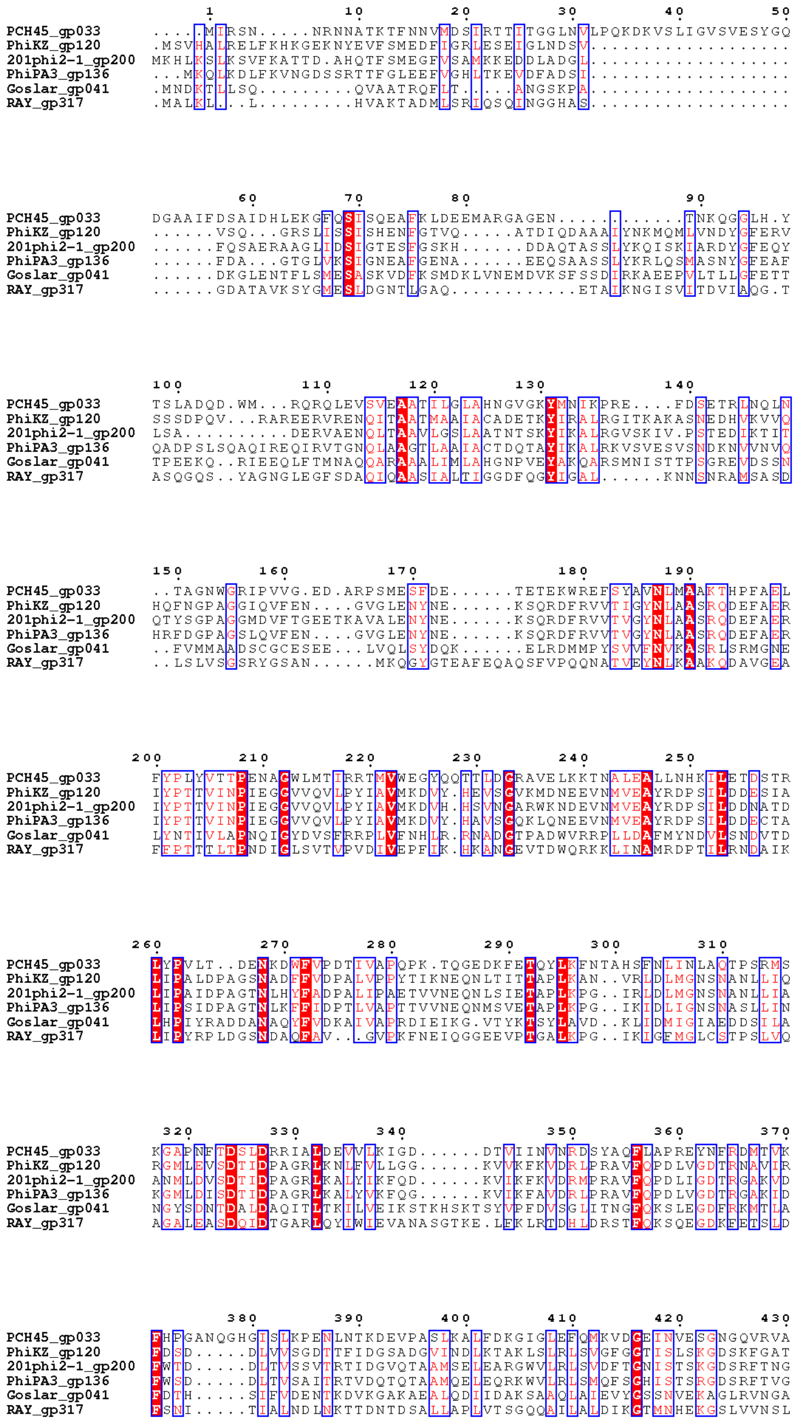

```
                           440       450         460       470       480
PCH45_gp033        NAQ.FTTAFRLDKPEEKIALDDAEVKALVP..AKFSAEGFSLEARLTNLNQLERGKLVDS
PhiKZ_gp120        DT.YVDKVLNEDGQVM..DNADPAVKAILDQLTDLAVIGFELDTRFTNTNRRQRGHLLQT
201phi2-1_gp200    DA.TIAKILNADRREI..DITAGDGKVLADTITDLEIVGFELQVRFTNTNRRQRGHLLQT
PhiPA3_gp136       EV.SVDRITDEDRRNV..SLETGAGKTLVDSITDLEIVGFDLDTRFTNTNRRQRGHLLQT
Goslar_gp041       NA.ELYRLVDQDN.IE.QPRKSEEFKQLAEDL.SFKLVGYELRIRRNNSNRRERGKHLDF
RAY_gp317          TNPSIIRVYDTVAKED.ANTKSGALRDAIDAL.TITFKGYFVRANLRNSNFRTREQRVDR

                   490       500       510        520       530       540
PCH45_gp033        DVQKEGFIIPTLSPICIVKPSQMEDEKVY.PKIESLQFVYRTMLRNDAVTSLLNRADTLE
PhiKZ_gp120        RALQFRHPIPMHAPVTLPMDTMTDEGPGE..VVKALTVNTNIRNSNNAVKRMLNYLAQLR
201phi2-1_gp200    RALQFRHPIPMHAPVTLPLSTMEDEGPGE..VVKALTVNTNIRNSNNAVKTLLNYLAQLR
PhiPA3_gp136       RALQFRHPIPMHAPVTLPLSTMDEEGPGE..VVKALTVNTNIRNSNNAVKRLLNYLAQLK
Goslar_gp041       NMKQERYAIGLLAPFSIPRP..LDWEENA.ADLDVLIQAANARFDNLMVEKLFSYKDALK
RAY_gp317          QTSVYKYMVQLGAPITAIAPVTDAWGPKESIAVETLSNATYARNSAMAISRLLDYVETIR

                      550       560       570       580       590       600
PCH45_gp033        SY...LGNGITHGLESNLGMEGLGQYYYRPHFERIKINVAQELNNLTSANKLGDIQGLIV
PhiKZ_gp120        EV...VHNGYN..RPKFGIIEGALSAVMRPTYRYKELDLEKVIDTIKSKDRWDDVCAAIL
201phi2-1_gp200    EL...TGNGFN..RPVINVYEGALAAVMRPTYHYDTMHLPDMIDTLKSKDRWDDVCSAIL
PhiPA3_gp136       EV...TANGFD..RPQFGAVEGALSAVMRPTYRYKELHLPDVIDTLKSQDRWEDVCSAIL
Goslar_gp041       AV...ATSRFR..QFGDIQIEGFGRYLIRPWFEEINLDLEKSVQGTTTAERRAEVSAAIM
RAY_gp317          DAGVTNNGLWQ..RYDENSIEGVGKAVVDPWYGEGSFDISKVVNGVKSSQTRDDVSEKIL

                       610       620       630       640       650       660
PCH45_gp033        SRMNEVIYRVNKVTGYSAALEEQFPGSTPKPHVAIGTDLYLPQFLMIQGDDRTVAIGFDH
PhiKZ_gp120        NCVKAELFPAHRDSNIEAAFRVISGNQDETPMYLFCSDKEIANYLMTKGDDRTLGAYLKY
201phi2-1_gp200    NKVKGILFPAYRNSNIEAAFRVISGNQDERPIFLFCTDKEIANYLMTKGDDRTLGAYLEY
PhiPA3_gp136       NAVKGELFPAYRESNIEAAFRVISGNQDERPMFLFCSDKEIANYLMTKGDDRTLGAYLEY
Goslar_gp041       NVIREMAYRMYSESGYQPALQLLSSNADEKPRCVVATDNYIPQFLQTDGDFRTVGVSFNF
RAY_gp317          NILRTYAYRMNAESGYNVALQMLYN..EVQPTLVIATDNIIAQYLMEVGDLRTAGISFKV

                      670       680       690       700       710         720
PCH45_gp033        TIASISDLRMSNKIVMQFTLPGEKEPHPMQSGVCGMIPEYLANFQMIRDQRI.NTEMRLT
PhiKZ_gp120        DIVSTNNQLFDGKLVVIPTRAVQQENDILSWGQFFYVSTVIADLPITRGGHQVTREIAAI
201phi2-1_gp200    DIVSTNNELFDGKLVVVPTRKTPVENDILSFGQFFYVSTIIADLPITRGGNQITREIAAV
PhiPA3_gp136       DLVSTNNELFDSKLVVIPTRKNPQENDILSFGQFFYVSTVIADLPITRGGQQVTREIAAI
Goslar_gp041       VVVDNPNVMLRDKIFVTLSRGIQGSPDPLTFGMFLYVPELVSVAQVTRNGQV.SKETMVQ
RAY_gp317          HVATTNNIEMRGKIIMTLSRMKSGGLDLLTFGMHLWVPEIVATVNVMRNGTY.IEETMVQ

                        730       740       750       760                 770
PCH45_gp033        PRYRYFNFTPVMIVIEIEGLEEAVAQRTAMNVNEKAVTAFPG.........EPVAPTPPV
PhiKZ_gp120        PFNLHVNNIPFALEFKITGFQKVMGETQFNGKLADLKP......................
201phi2-1_gp200    PFNLHINNVPFALEFDVTGFEQVMGHSMWNKPVVDALGA.....................
PhiPA3_gp136       PFNLHVNNIPFALEFKITGFQEVMGESQWNKTVASLKP......................
Goslar_gp041       PRCRFVNNCPLLGLINVVNLRKVYTENIKLPVQTN.........................
RAY_gp317          PRNLHVVVCPIVANITITGLEEAYTGKITVPVDIVSEPESAGAQDLGGLNTTPATTTNPA

PCH45_gp033        TDGE
PhiKZ_gp120        ....
201phi2-1_gp200    ....
PhiPA3_gp136       ....
Goslar_gp041       ....
RAY_gp317          A...
```

**P**

**i**

```
                        1        10         20         30         40         50         60
PCH45_gp086             MTELELETWLYRYSARTEGNLPNPKAFESDNWNLPKNVALHYLPIAAQDLGPEEDYFPTR
RAY_gp164               ..MILYNAFFRRTVVRKKEQIFGPRFLQLSQFELPRGSLLHYIPTDLTEQGIENNDLLIN
Goslar_gp228            MAKLKYLDYYRQFGVRRYQELLSPRLRLVQLLQLPRYAVYHYTATDPSLIGPPENEYMLS
PhiKZ_gp178             .MRLNITQFLKNYSVREYAKLQSPKLHALNKLDLPFESIYQFFDGNNAVMGPSQTDPLFS
201phi2-1_gp273/274     .MRLKIDQFLRSFGLRQAAELQSPRLHAIGKFDFPMETVYHFHADNQAVLGPSQVDPIIA
PhiPA3_gp211+209        .MRLKIAQFLKNYGLKQASELQKPRLHAVKKLELPLETVYQFFDDNYAVRGPSQSDPIFA

                                 70         80         90        100        110        120
PCH45_gp086             GFNRQHYVHHVEDMTQKDGNPRFIPAFQIKNMIRFYYQKNRKVKRVTKLPLALMDKRYPI
RAY_gp164               RYSDDIYIDHVPQIQTPLGNPQRKPISL.MPAIKRYHNTHRRFKLVRNINSVIRNKLYMI
Goslar_gp228            KESAQLWISFADDLATKQGAPRRDQRFQLPRAKLDYRASHRRFRLVTDLSTVEKNINAQI
PhiKZ_gp178             KHQGKVYIEHVTDMLTFEGNPRRTSNIP.ATMIQEFRRQNRFFKPLRSDTGFKLSNQNIL
201phi2-1_gp273/274     KLKGKVFLEQITELKSEVGNPKRTSVLP.PTLINDFRRQNRFFKPLRRDESVKLNQQNVA
PhiPA3_gp211+209        RLMGKSFIEHKLELKTLDGNARRTSVIA.TTLEQEFRRQHRFFKPLRKDEAVKLNLQNVA

                                130        140        150        160        170
PCH45_gp086             VFNYAMIPLRWRYLPTAWARIQRWNNLYGTLCDTVAAVAKE.STRNHLIFFNLPEVLPPR
RAY_gp164               VENYAIAQHQLVYRPAMYSNYYRWYNMQYTVMHNMTRLQGE.SDRNQFVYLPIPASLPQL
Goslar_gp228            VANYAHLNHMYIYRPTLTRWYDEWFNVRKTIWSKANEIAQQ.SDRQHFLLFRIPSTLPKI
PhiKZ_gp178             VMNYNLINPQWNYMASYKATWFRWSNDMRTFWSGVAAACERFPGWNQFIDVHLPELVPPP
201phi2-1_gp273/274     LFNYNLLNPLYRYIASYKANYYRWVNDTATFWDGVQDACTRFPTWNHFIELHVPESMPTM
PhiPA3_gp211+209        VFNYNMLNDLYKYQANYKAGWYRWVNNTATFWDGVVDAHKRF.GWNQFIEIHIPESIPLY

                      180       190        200        210        220
PCH45_gp086             NKFHAVEEYEQAAHAHGVDGTQRPEFFEGKLRADETRITSF..........RELFAPLAD
RAY_gp164               AQLKIYEERLSAGLDKVSNGTGLENFDDLSA...EARIGYAMEAMLEYQDTADLSQPMAR
Goslar_gp228            SLMRTTQQAP..................................................
PhiKZ_gp178             SSFNKLRGGL..................................................
201phi2-1_gp273/274     AQFKMFETSQ..................................................
PhiPA3_gp211+209        SEFVQFSKGQ..................................................

                      230       240        250        260        270
PCH45_gp086             NLDRILGDAAVKELNEGFESFDEATLGWSDDAEIDLEGCDCDLGF..............E
RAY_gp164               DQAMAFQTLVMNATNDTV.NLAQRIPGYYNGD....AGSVLRVGMEALGAYASKVMNFTP
Goslar_gp228            ............................................................
PhiKZ_gp178             ............................................................
201phi2-1_gp273/274     ............................................................
PhiPA3_gp211+209        ............................................................

                          280       290        300        310        320        330
PCH45_gp086             AWTRVTIGWFPDDDSLQLVDLWRWLGEKREDSKLNAIDPAHYEKVFFVFTVGGRYNVLSM
RAY_gp164               QLSMAVMNRLRTPADYWFIHFWMWLGNQREASLFSMLDHDKLDKIHLVLGNVGAYSVIRL
Goslar_gp228            ..TRNNLMNFATPEELSFFDFFRWLGVDRENSTMASLDRATLDKLNVVFTDGVYFFTLNL
PhiKZ_gp178             ..TQDLANQFNTPDILNIFDLVRWVSDDRESSFMNVIDAKYYGNINLLFRVQSSFFVINL
201phi2-1_gp273/274     ..TQNLLETFRTGELMDLFDLYRFLGPDRKTSFVSKVDKKYFPQINFFIRVQGSFFVINL
PhiPA3_gp211+209        ..TQALLEKFRTPAVLNIFDLYRFFGNSRETSFMSVLPREAYEKINFLIRAQGSFFVINL

                          340       350
PCH45_gp086             AKLNEWREKSGIQTMT............................................
RAY_gp164               DILDQWRTEILSKTSNPETAV.......................................
Goslar_gp228            GLLEEWRTGLVKEGEELDKTT......L................................
PhiKZ_gp178             GMLDQWRRDPEIKDDK............................................
201phi2-1_gp273/274     GKLDEWREQTEEEKEADKLKLLEDAVTFETYQDADGNYETYEEKRNLSDMGISERLTYGF
PhiPA3_gp211+209        GLLEEWRKSPEFEE.......................................EQLTVAQ

                                                        360           370        380
PCH45_gp086             ...................QRFHNYLSNMMAFRQ.....LDQRDVILETVPESEADKPET
RAY_gp164               ...............QNFRKHILKFLTRLFDV.......KNGNELVIEHLDDGADAHE..
Goslar_gp228            ...............ETFQRRWMMAVTRLWSQSANEEEQKQEEEQVLRQERDILADSP..
PhiKZ_gp178             ...........GYDTQQIARRLISLVSAMVEY.......NQGNTSLIKEDTPIFNEDI..
201phi2-1_gp273/274     ETYVDEFGMEAYFKPELIQRRFVSLMTTLVEY.......AAGNDQLIENDANVQANAM..
PhiPA3_gp211+209        ESYVDELGLEVVYTPDVMQRRILSLLTTLVEY.......NHGNDTLVEQDSSDVAISP..
```

```
                     390        400        410         420                  430
PCH45_gp086          IGMVTAGDSDASVVEDDTEAPEQGEERQTDRR..GAL.......GMVAPQFA....GG..
RAY_gp164            V.....GEE......GQEIAPEQGSVVPPT.ADTGLFGTELPTLDDPAPVVASSTKKGTS
Goslar_gp228         ..........DAEYDDRDVMLPNGEDL.ATVLDPDELEAELVGGSDEDDDAD.......A
PhiKZ_gp178          I.....VDE......ETPVSYEEGEAEEVEDVESNNVEEEVTEVD....VID.......D
201phi2-1_gp273/274  I.....EAEAEESDEPVIVVNEDEEEFS.EEEEPVEIKAKQVSVDLDIEEPE.......V
PhiPA3_gp211+209     ..........TALEEPTDVVSEDEDIEPTDDVEPQE.EAEDVDVGDVVD.........D

                          440       450                       460       470
PCH45_gp086          RQLALTDKVMDLNELDNQ............DITEV...TDEDVPELPPERSARVVNK...
RAY_gp164            KGKDIGSALNDEEPIDAVDLYE..TRTLADDIDDDEFNHDTES.................
Goslar_gp228         WNAIVERAFEDVREVPGVEVQPDFQPDEDQDITDDLFEVVTDDT............EEEK
PhiKZ_gp178          VQFDP.TNLTNLIDIGAI..EVTYTPPPESDLEVTKLIIEKDPLESSPSRQIKEEKELIV
201phi2-1_gp273/274  VTPPL.SKVYRSFDLDIL..QVTFEPPPELDLERTTLNIENDELSGVA.......AKTLK
PhiPA3_gp211+209     SSKDD.SSIVKTLDLGLM..EVTYNPPPESELEITTLVIEKDPLETAPLVKEVEAEKTLN

                          480       490       500       510       520       530
PCH45_gp086          ..PVKEDLPAVINVDTSNPLEAGVAVKSAELLRKGVISRQEHERHIRLSRKYKEIKVD..
RAY_gp164            .RRGEAAAY....TDTSTNPEDGVLLYLERLSDAGVLTVAEYKRFQQLAVAYKNIPNPVG
Goslar_gp228         VDVFDAATLPKTHGEYIQSLSDAVLVRASDLADAGQLSGVQYKAAMRNADAIHEIEAPDG
PhiKZ_gp178          TDVPEDELFINTEVDEEDKLIDAIRAKAYDMYRVNMISANTFEQAQEDSIMYRRLPDPFT
201phi2-1_gp273/274  VEQARPATIIDQEFTTGDKMLDGVAKRAFRLAKVGMISERTFEMAIDDAQRYEEMPDPFG
PhiPA3_gp211+209     VSDGKATKSALPRFETDDPLVDGVGAKAFELYQVGMISPRTFEQAVEDASSYKSLPDPFG

                               540       550       560       570       580
PCH45_gp086          ........GVTLEEAAKPPIQDIWNFKPAQIPDIPNVVDKGMLQSSLINFDTEYVEKVMP
RAY_gp164            E........GTLADLMIIDPSKVTDLGGLDAPDSISIIDKGLLKSSTKDFDKNYIKNLME
Goslar_gp228         S........GLTLAEYVEVKPKAAKLAPAKVMPEADVIVDKSLCKSTLAVWDKHYVEHVMQ
PhiKZ_gp178          DPNDESVEPMTIAEAMEYHPDDLKIPEDTTFEDKPTIVDKSMLGSKLKAIQRKYNKVLLK
201phi2-1_gp273/274  S.......GLTVKEAMQYAKEDFEVPAH.EFPDKTTIMDKSMLRSVHKSMMRKYIKTLLP
PhiPA3_gp211+209     S.......GKTIAEAMQYAAEDYAIPEV.KFADKTTILDKSMLGAKHKAMVRKYNKTLLP

                          590       600       610       620       630       640
PCH45_gp086          KDTANMVLAIQKAGIAVTGFEREEFSDALSKTIDYRVKVTPVHGKESTLSFQLPKIDENG
RAY_gp164            ADILNAVLSIQNSGVAIIDYQREEKKDARNSYVVYSVQVQPVGGKVTTLRFRVPKLNEDG
Goslar_gp228         KDIAAAVVAVQQAGYPVTGYKVEDVVDVMGDYQVITIKLAPVGGVPTTFPVMLPKVRKDG
PhiKZ_gp178          KDILNSVLSVQKQGVSVTNYKIETVRDSGNHYQIHKVTLKPIRGRSSQVMFRIPVIDNDG
201phi2-1_gp273/274  KDILQSIMAIQRQGVAVTDIKIQENEDAMNHTQTFTVTVKPVRGMASQLNFTIPVIDKDG
PhiPA3_gp211+209     KDIMNSVLAIQQQGIAVTDIKMEEVQDAMNHYQSFTVTVKPIRGRSSQLRFRIPVIDRDG

                          650       660       670       680       690       700
PCH45_gp086          KYMINGVRYYMRKLRADMPIRKINTHTVALTSYYGKLFVERSQKKVASFDNWILSALTSE
RAY_gp164            TSVVNGVKSRLRKQRVDIPIRKVSASKVSLTSYYGKLFMERSELSVHNYSEWLSRRIKAL
Goslar_gp228         IYVVNGIKYRMRKQRVDKPIRKVSSSEVALSTAYGKVFVKRSERVVNNAGRWLTRQLTAM
PhiKZ_gp178          RFMSNGVTYRQRLQRADIPIRKVNPRKVALTSYYNKTFVTRSERAENNFDNWLIRAITNR
201phi2-1_gp273/274  RFLSNGVTYRQRVQRADIPIRKVSPTRVALTSYTNKTFVMRSPRAEHAFDVWLTRQIDTK
PhiPA3_gp211+209     RFRSNGVTYRQRMQRADVPIRKVNPTRVALTSYYNKTFVSRSGLAVNNYDVWLVRQITAR

                           710        720       730       740       750       760
PCH45_gp086          ILDGSRV.KGVVYGNSYG.EQQPVPRIYSAIAQSYLEMQIGDIHFHFDYEGMEKNFPGVA
RAY_gp164            AIADTPEITRLVLGRSFD.PKVRVPHLYAILASQFKGFQYLDYTFNFNRREMVAVFGEEV
Goslar_gp228         YYDKSSPLTTLKRGNVFD.PAVKVPYYFAVLARNYTQIQFADWDIRLDIKTLPEFIGEDR
PhiKZ_gp178          ALDGSDMSVHSVTYAELDQSNYVLPRVYTTLGTAFKGFHNHRNVFYFNFKDRNEFFAK.Q
201phi2-1_gp273/274  ARDATNPIVTNQKYIELDLSEYHLPRVYSAMAGKFASIDNGANHLYFNYPNRAKYFKEKF
PhiPA3_gp211+209     ALDPNDQSITNVRYAELDQSAYVLPRVYSAMGGAFQAFDNGKNQLYFKYADRVDYFREKF

                                770        780         790          800       810
PCH45_gp086          ...LPKTKNQDWP..VGRKGNEIVYM.DRNGRLKVKG....QDIGTIPSLIDLDDNTAPA
RAY_gp164            ...IEKYAENRFVV.CGMIEDSPLLM.DDNGTLYQANNDVLSNLGDFETLIGLDVSKAPI
Goslar_gp228         .FN.E.QLAEGLLTFARNTSGEFLAIDERNQLYICQSGQTPTPVGTFPDFFGINAAKMPI
PhiKZ_gp178          GLHIEDYETDDLIM.VGFNGTDAILL.DKNSIFYMKTGNELEPIGTITDILGLDLTKAPL
201phi2-1_gp273/274  NIDPEFNERDGYVM.VGVREGHAILL.DKAGIFYLKEGNELEPMGTLLDMTGINAAKAPL
PhiPA3_gp211+209     HLDVTQFEKDGYVM.TGVREGQPIML.DQAGNFYLQDGNDLEPMGTLVDMVGLNLTKAPL
```

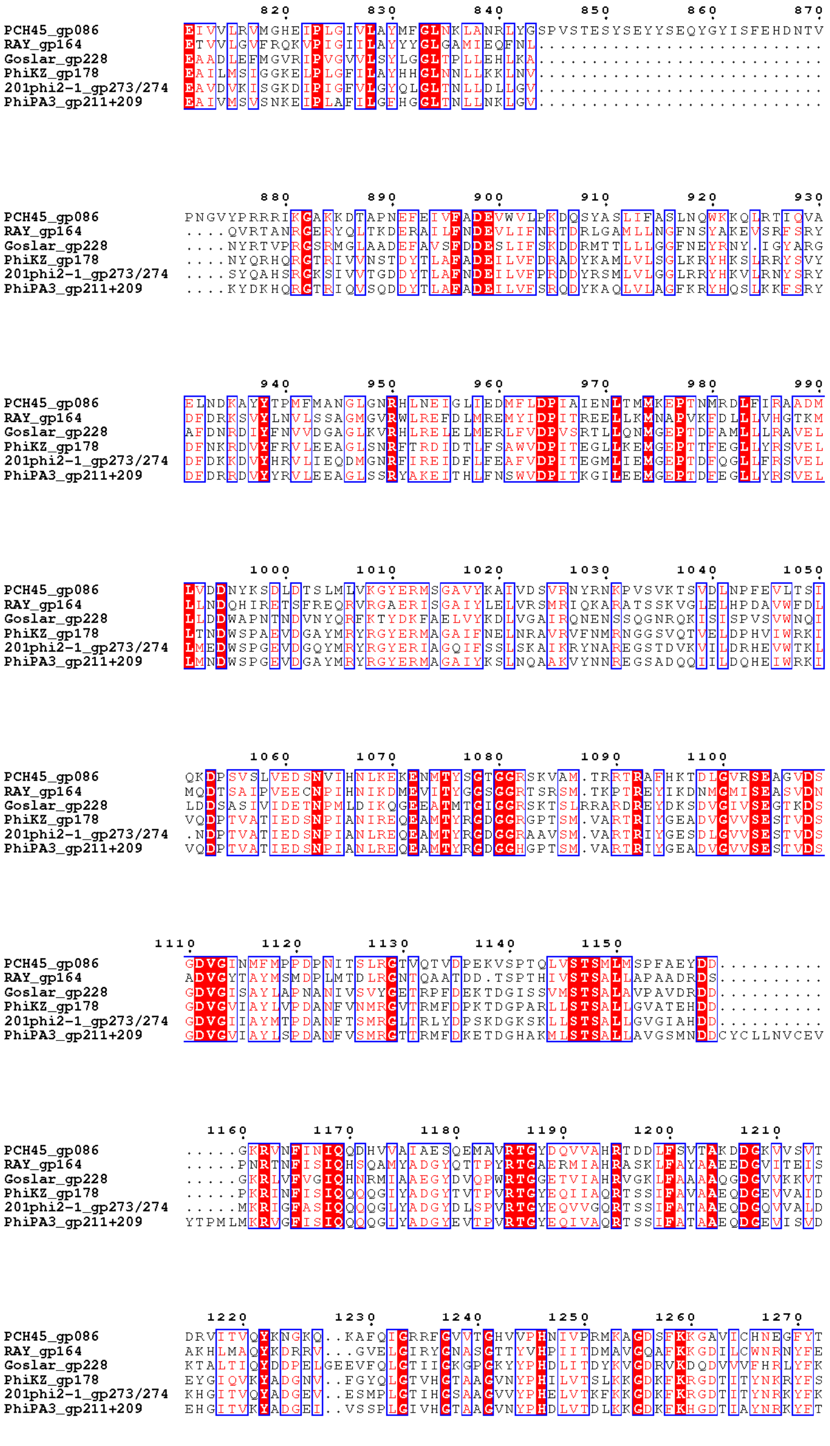
820 830 840 850 860 870
PCH45_gp086 EIVVLRVMGHEIPLGIVLAYMFGLNKLANRLYGSPVSTESYSEYYSEQYGYISFEHDNTV
RAY_gp164 ETVVLGVFRQKVPIGIILAYYGLGAMIEQFNL............................
Goslar_gp228 EAADLEFMGVRIPVGVVLSYLGGLTPLLEHLKA...........................
PhiKZ_gp178 EAILMSIGGKEIPLGFILAYHHGLNNLLKKLNV...........................
201phi2-1_gp273/274 EAVDVKISGKDIPIGFVLGYQLGLTNLLDLLGV...........................
PhiPA3_gp211+209 EAIVMSVSNKEIPLAFILGFHGGLTNLLNKLGV...........................
880 890 900 910 920 930
PCH45_gp086 PNGVYPRRRIKGAKKDTAPNEFEIVFADEVWVLPKDQSYASLIFASLNQWKKQLRTIQVA
RAY_gp164 ....QVRTANRGERYQLTKDERAILFNDEVLVIFNRTDRLGAMLLNGFNSYAKEVSRFSRY
Goslar_gp228 ....NYRTVPRGSRMGLAADEEAVSFDDESLIFSKDDRMTTLLLGGFNEYRNY.IGYARG
PhiKZ_gp178 ....NYQRHQRGTRIVVNSTDYTLAFADEILVFDRADYKAMLVLSGLKRYHKSLRRYSVY
201phi2-1_gp273/274 ....SYQAHSRGKSIVVTGDDYTLAFNDEILVFPRDDYRSMLVLGGLRRYHKVLRNYSRY
PhiPA3_gp211+209 ....KYDKHQRGTRIQVSQDDYTLAFADEILVFSRQDYKAQLVLAGFKRYHQSLKKFSRY
940 950 960 970 980 990
PCH45_gp086 ELNDKAYYTPMFMANGIGNRHLNEIGLIEDMFLDPIAIENLTMMKEPTNMRDLFIRAADM
RAY_gp164 DFDRKSVYLNVLSSAGMGVRWLREFDLMREMYIDPITREELLKMNAPVKFDLLLVHGTKM
Goslar_gp228 AFDNRDIYFNVVDGAGLKVRHLRELELMERLFVDPVSRTLLQNMGEPTDFAMLLLRAVEL
PhiKZ_gp178 DFNKRDVYFRVLEEAGLSNRFTRDIDTLFSAWVDPITEGLLKEMGEPTTFEGLLYRSVEL
201phi2-1_gp273/274 DFDKKDVYHRVLIEQDMGNRFIREIDFLFEAFVDPITEGMLIEMGEPTDFQGLLFRSVEL
PhiPA3_gp211+209 DFDRRDVYYRVLEEAGLSSRYAKEITHLFNSWVDPITKGILEEMGEPTDFEGLLYRSVEL
1000 1010 1020 1030 1040 1050
PCH45_gp086 LVDDNYKSDLDTSLMLVKGYERMSGAVYKAIVDSVRNYRNKPVSVKTSVDLNPFEVLTSI
RAY_gp164 LLNDQHIRETSFREQRVRGAERISGAIYLELVRSMRIQKARATSSKVGLELHPDAVWFDL
Goslar_gp228 LLDDWAPNTNDVNYQRFKTYDKFAELVYKDLVGAIRQNENSSQGNRQKISISPVSVWNQI
PhiKZ_gp178 LTNDWSPAEVDGAYMRYRGYERMAGAIFNELNRAVRVFNMRNGGSVQTVELDPHVIWRKI
201phi2-1_gp273/274 LMEDWSPGEVDGQYMRYRGYERIAGQIFSSLSKAIKRYNAREGSTDVKVILDRHEVWTKL
PhiPA3_gp211+209 LMNDWSPGEVDGAYMRYRGYERMAGAIYKSLNQAAKVYNNREGSADQQIILDQHEIWRKI
1060 1070 1080 1090 1100
PCH45_gp086 QKDPSVSLVEDSNVIHNLKEKENMTYSGTGGRSKVAM.TRRTRAFHKTDLGVRSEAGVDS
RAY_gp164 MQDTSAIPVEECNPIHNIKDMEVITYGGSGGRTSRSM.TKPTREYIKDNMGMISEASVDN
Goslar_gp228 LDDSASIVIDETNPMLDIKQGEEATMTGIGGRSKTSLRRARDREYDKSDVGIVSEGTKDS
PhiKZ_gp178 VQDPTVATIEDSNPIANIREQEAMTYRGDGGRGPTSM.VARTRIYGEADVGVVSESTVDS
201phi2-1_gp273/274 .NDPTVATIEDSNPIANLREQEAMTYRGDGGRAAVSM.VARTRIYGESDLGVVSESTVDS
PhiPA3_gp211+209 VQDPTVATIEDSNPIANLREQEAMTYRGDGGHGPTSM.VARTRIYGEADVGVVSESTVDS
1110 1120 1130 1140 1150
PCH45_gp086 GDVGINMFMPPDPNITSLRGTVQTVDPEKVSPTQLVSTSMLMSPFAEYDD..........
RAY_gp164 ADVGYTAYMSMDPLMTDLRGNTQAATDD.TSPTHIVSTSALLAPAADRDS..........
Goslar_gp228 GDVGISAYLAPNANIVSVYGETRPFDEKTDGISSVMSTSALAVPAVDRDD..........
PhiKZ_gp178 GDVGVIAYLVPDANFVNMRGVTRMFDPKTDGPARLLSTSALLGVATEHDD..........
201phi2-1_gp273/274 GDVGIIAYMTPDANFTSMRGLTRLYDPSKDGKSKLLSTSALLGVGIAHDD..........
PhiPA3_gp211+209 GDVGVIAYLSPDANFVSMRGTTRMFDKETDGHAKMLSTSALLAVGSMNDDCYCLLNVCEV
1160 1170 1180 1190 1200 1210
PCH45_gp086 .....GKRVNFINIQQDHVVAIAESQEMAVRTGYDQVVAHRTDDLFSVTAKDDGKVVSVT
RAY_gp164 .....PNRTNFISIQHSQAMYADGYQTTPYRTGAERMIAHRASKLFAYAAEEDGVITEIS
Goslar_gp228 .....GKRILVFVGIQHNRMIAAEGYDVQPWRTGGETVIAHRVGKLFAAAAQGDGVVKKVT
PhiKZ_gp178 .....PKRINFISIQQQQGIYADGYTVTPVRTGYEQIIAQRTSSIFAVAAEQDGEVVAID
201phi2-1_gp273/274 .....MKRIGFASIQQQQGLYADGYDLSPVRTGYEQVVGQRTSSIFATAAEQDGQVVALD
PhiPA3_gp211+209 YTPMLMKRVGFISIQQQQGIYADGYEVTPVRTGYEQIVAQRTSSIFATAAEQDGEVISVD
1220 1230 1240 1250 1260 1270
PCH45_gp086 DRVITVQYKNGKQ..KAFQICRRFGVVTGHVVPHNIVPRMKAGDSFKKGAVICHNEGFYT
RAY_gp164 AKHLMAQYKDRRV...GVELGIRYGNASGTTYVHPIIITDMAVGQAFKKGDILCWNRNYFE
Goslar_gp228 KTALTIQYDDPELGEEVFQLGTIIGKGPGKYYPHDLITDYKVGDRVKDQDVVVFHRLYFK
PhiKZ_gp178 EYGIQVKYADGNV..FGYQLGTVHGTAAGVNYPHILVTSLKKGDKFKRGDTITYNRKYFS
201phi2-1_gp273/274 KHGITVQYADGEV..ESMPLGTIHGSAAGVVYPHELVTKFKKGDKFKRGDTITYNRKYFK
PhiPA3_gp211+209 EHGITVKYADGEI..VSSPLGIVHGTAAGVNYPHDLVTDLKKGDKFKHGDTIAYNRKYFT

```
1280 1290 1300 1310 1320 1330
PCH45_gp086            PDRRDPTSSALKQGCYPYTVLIEKNETWEDSSLMSPELAYKMRANVTHIRTLFVRFDNAI
RAY_gp164              RDFMEPMQCSWKAGAMVRVALMEEEFTFEDSSVISQTTAKRLGTRTAKPIAITVDFTQEV
Goslar_gp228           RDWWNPGQVVWRAGVLSLVGILDIPETHEDSSYIFDNLADRLVTPSIKSRTLIVDAHLNI
PhiKZ_gp178            EDRYTPGQVLWKAGCMAVVAFDDNLDTLEDGSVISEDLAKRLNTQTTAIKNIDVRFDQTI
201phi2-1_gp273/274    EDRYNPDQVSLMPGVIGVVAFDDNLDTLEDGSVVSESIAKKLNTQTTDVKNVIVRFDQHV
PhiPA3_gp211+209       ADRYAPGQVLWKAGCMATVAFCDNLDTLEDGSTISKELAVRLQTQTTDIKNITVRFDQHV

1340 1350 1360 1370 1380
PCH45_gp086            HNLVSVGEEVDIDTVLAVIEDKVSAGVG...SDNLDALNLLSRPTPRANNSGKVEEIQVI
RAY_gp164              RNLLPVGTQVDPETILCTLEDPVTANLGQFDDESFDSLRILANKNPKAKVTGEIARIEVT
Goslar_gp228           RGLVKIGDHVEPRTILATLEEPIATDLSDESQEAVEALRRMSQYNPRADVHGEVVQIDVL
PhiKZ_gp178            RDMVKVGDHVDLNSILCIIEDPETAAHSLYDEASIETLRKLSAYSPRAKLVGTVSKIECF
201phi2-1_gp273/274    SELVQPGDHLDLSSILCIIEDPETAAGSLFDDASIETLKRLSSSTPTAKVVGTVSKIECF
PhiPA3_gp211+209       SELVKRGDHVDLNSILCIIEDPETAENTLFGDAAIETLRRLAAHTPRAKVVGTVSKIECY

1390 1400 1410 1420 1430 1440
PCH45_gp086            YFGDKDDMSPSVRKVADKYDRVRGQLARELNNGDA.TTGEITTPARVDGISVDIDMMAIR
RAY_gp164              YRGSVDDMSESLGLIANRSDRERTKLNRQLGNNEA.NTGESLTPIRVQGTPLEANQAIIE
Goslar_gp228           YRAQIEDMSETMAALVGDADKARAKRVKELKLNEATTGRINNSNVRIDGNPLGENQIAIR
PhiKZ_gp178            YHGEIDDMTPSLQALANTSDKQRAERAKSLKEPT..FTGQVDANYRVKGRALEYDHMIIR
201phi2-1_gp273/274    YHGDIEDMSDNLRELAEASDKARAEIAQQTGRNA..YSGEVDSSFRIKGQILEPDSMVIR
PhiPA3_gp211+209       YHGDIEDMSENLQAIARASDKQRADIAKSLNQPV..FTGEVDSSFRVKGQILEPDHMVIR

1450 1460 1470 1480 1490 1500
PCH45_gp086            IFITEERIMSGGDKLVLSNQLKSVSAGEQRGPTYTASELWPGSGVRKIDLEFSYRAEEAR
RAY_gp164              VYVITPMPTLSGDKGVFANQMKSTFGSIMPDGITTA.......SGKRLDAKFSNKSIANR
Goslar_gp228           IFMRYDLGAGMGDKGTFAAQAKSVVSSVGHGVNKTE.......DGHTLDAIFSGTSIANR
PhiKZ_gp178            VYIDHDIPCGVGDKGVVANQMKTVFSRVMTGRNETE.......DGRNIDLLFGNTSVEER
201phi2-1_gp273/274    IYIDHDIPFGTGDKAVLCNQMKTVISRVMTGTNTLE.......DGTPIDIIFGNTSVEER
PhiPA3_gp211+209       IYIDHDISCGVGDKGVLGNQMKTVISRVMEGRNETE.......DGTPIDLIFGNTSVEER

1510 1520 1530
PCH45_gp086            IVHSSIMAGMSIICQEFIGFSMVQAYDS.......
RAY_gp164              MVTSPYVMGTTNVLLDTITRKACAMYKRLKG....
Goslar_gp228           IIGSPEVVGTVNLGLLYATERAVDIYFNDAK....
PhiKZ_gp178            MVLSPKIIATTTGILWAAGKHLVAVYRGNTNAKAK
201phi2-1_gp273/274    MVMSPKLIATTSILLKALSKRVAGVYRGTVNAKAK
PhiPA3_gp211+209       MVMSPKLISTTSILLRALSKHIAGVYRGTTNAKAK
```

**ii**

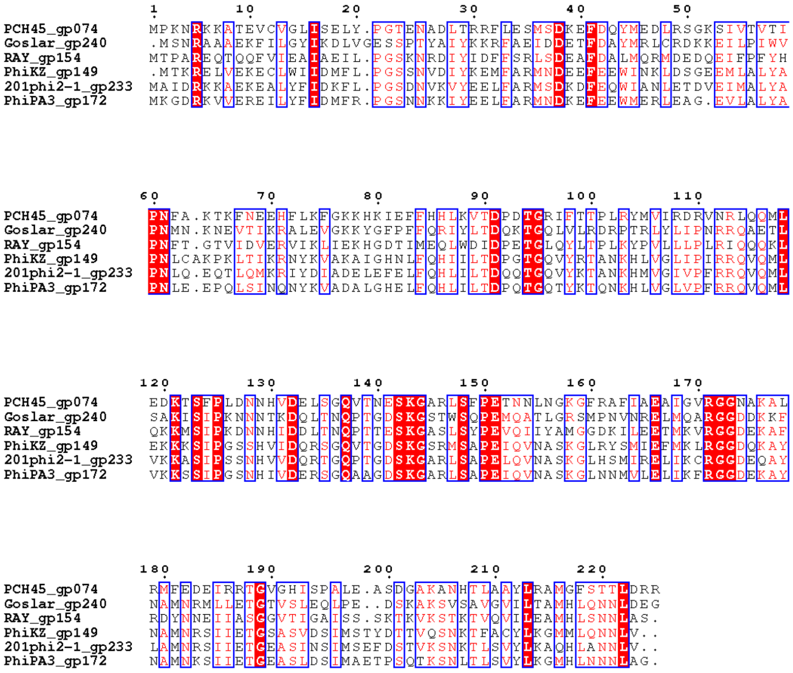
1 10 20 30 40 50
PCH45_gp074 MPKNRKKATEVCVGLIISELY.PGTENADLTRRFLESMSDKEFDQYMEDLRSGKSIVTVTI
Goslar_gp240 .MSNRAAAEKFILGYIKDLVGESSPTYAIYKKRFAEIDDETFDAYMRLCRDKKEILPIWV
RAY_gp154 MTPAREQTQQFVIEAIAEIL.PGSKNRDIYIDFFSRLSDEAFDALMQRMDEDQEIFPFYH
PhiKZ_gp149 .MTKRELVEKECLWIIDMFL.PGSSNVDIYKEMFARMNDEEFEEWINKLDSGEEMLALYA
201phi2-1_gp233 MAIDRKKAEKEALYFIDKFL.PGSDNVKVYEELFARMSDKDFEQWIANLETDVEIMALYA
PhiPA3_gp172 MKGDRKVVEREILYFIDMFR.PGSNNKKIYEELFARMNDKEFEEWMERLEAG.EVLALYA
60 70 80 90 100 110
PCH45_gp074 PNFA.KTKFNEEHFLKFGKKHKIEFFHHLKVTDPDTGRIFTTPLRYMVIRDRVNRLQQML
Goslar_gp240 PNMN.KNEVTIKRALEVGKKYGFPFFQRIYLTDQKTGQLVLRDRPTRLYLIPNRRQAETL
RAY_gp154 PNFT.GTVIDVERVIKLIEKHGDTIMEQLWDIDPETGLQYLTPLKYPVLLLPLRIQQQKL
PhiKZ_gp149 PNLCAKPKLTIKRNYKVAKAIGHNLFQHIILTDPGTGQVYRTANKHLVGLIPIRRQVQML
201phi2-1_gp233 PNLQ.EQTLQMKRIYDIADELEFELFQHLILTDQQTGQVYKTANKHMVGIVPFRRQVQML
PhiPA3_gp172 PNLE.EPQLSINQNYKVADALGHELFQHLILTDPQTGQTYKTQNKHLVGLVPFRRQVQML
120 130 140 150 160 170
PCH45_gp074 EDKTSFPLDNNHVDELSGQVTNESKGARLSFPETNNLNGKGFRAFIAEAIGVRGGNAKAL
Goslar_gp240 SAKISIPKNNNTKDQLTNQPTGDSKGSTWSQPEMQATLGRSMPNVNRELMQARGGDDKKF
RAY_gp154 QKKMSIPKDNNHIDDLTNQPTTESKGASLSYPEVQIIYAMGGDKILEETMKVRGGDEKAF
PhiKZ_gp149 EKKKSIPGSSHVIDQRSGQVTGDSKGSRMSAPEIQVNASKGLRYSMIEFMKLRGGDQKAY
201phi2-1_gp233 VKKASIPSSNHVVDQRTGQPTGDSKGARLSAPELQVNASKGLHSMIRELIKCRGGDEQAY
PhiPA3_gp172 VKKSSIPGSNHIVDERSGQAAGDSKGARLSAPEIQVNASKGLNNMVLELIKFRGGDEKAY
180 190 200 210 220
PCH45_gp074 RMFEDEIRRTGVGHISPALE.ASDGAKANHTLAAYLRAMGFSTTLDRR
Goslar_gp240 NAMNRMLLETGTVSLEQLPE..DSKAKSVSAVGVILTAMHLQNNLDEG
RAY_gp154 RDYNNEIIASGGVTIGAISS.SKTKVKSTKTVQVILEAMHLSNNLAS.
PhiKZ_gp149 NAMNRSIIETGSASVDSIMSTYDTTVQSNKTFACYLKGMMLQNNLV..
201phi2-1_gp233 LAMNRSIIETGEASINSIMSEFDSTVKSNKTLSVYLKAQHLANNLV..
PhiPA3_gp172 NAMNKSIIETGEASLDSIMAETPSQTKSNLTLSVYLKGMHLNNNLAG.

**iii**

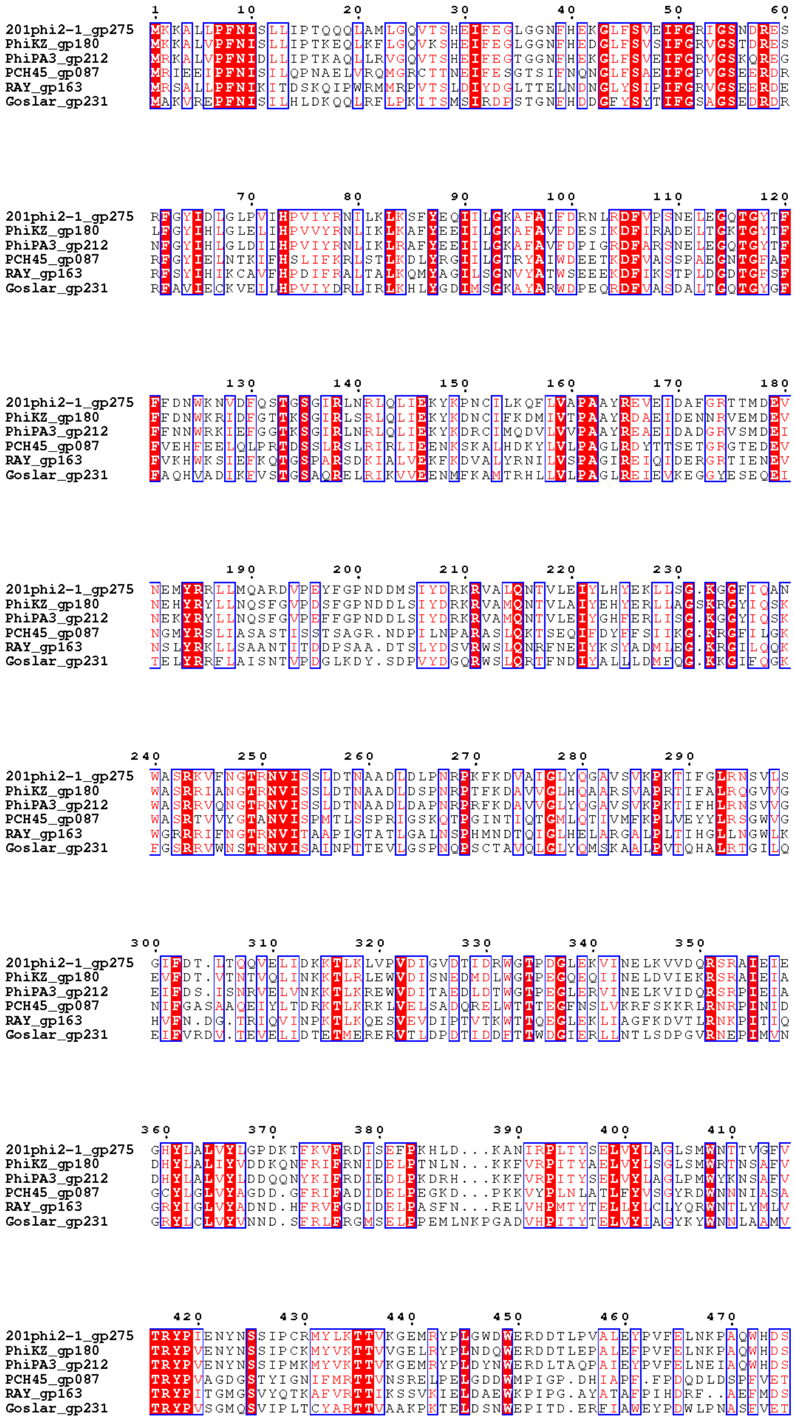

```
                   480       490         500        510        520        530
201phi2-1_gp275  ISLPPATLAPLTADFDGDTT.SWIACYSEESIAECNKFFKSRLAYIRAGGGIAFSLNIHT
PhiKZ_gp180      VSISPSILAPLGAD..............................................
PhiPA3_gp212     TSVSPAVLAPLTADFDGDTV.SFNAVYSKEAIEESDKFFKSRLAYIKAGGGLAFSINIHT
PCH45_gp087      MSPHTARIKAQGGDFDGDRESC.IGAMSKEARDEYQRLKSKRDWYVGADNKLVLSAANDN
RAY_gp163        LSVHPSRLGLLGGDYDGD.TMSATILLGDDVLRECAMLLSRRESYITGRGEFLIDVTNET
Goslar_gp231     TIVPAPYLAGLGGDQGKANKMPVFWQI.................................

                    540        550
201phi2-1_gp275  LNLTLRYMTGDPIPRK
PhiKZ_gp180      ................
PhiPA3_gp212     LNLTLRYMTGDPVARD
PCH45_gp087      IELLLLQITGGPTK..
RAY_gp163        LDFTLKALTS......
Goslar_gp231     ................
```

**iv**

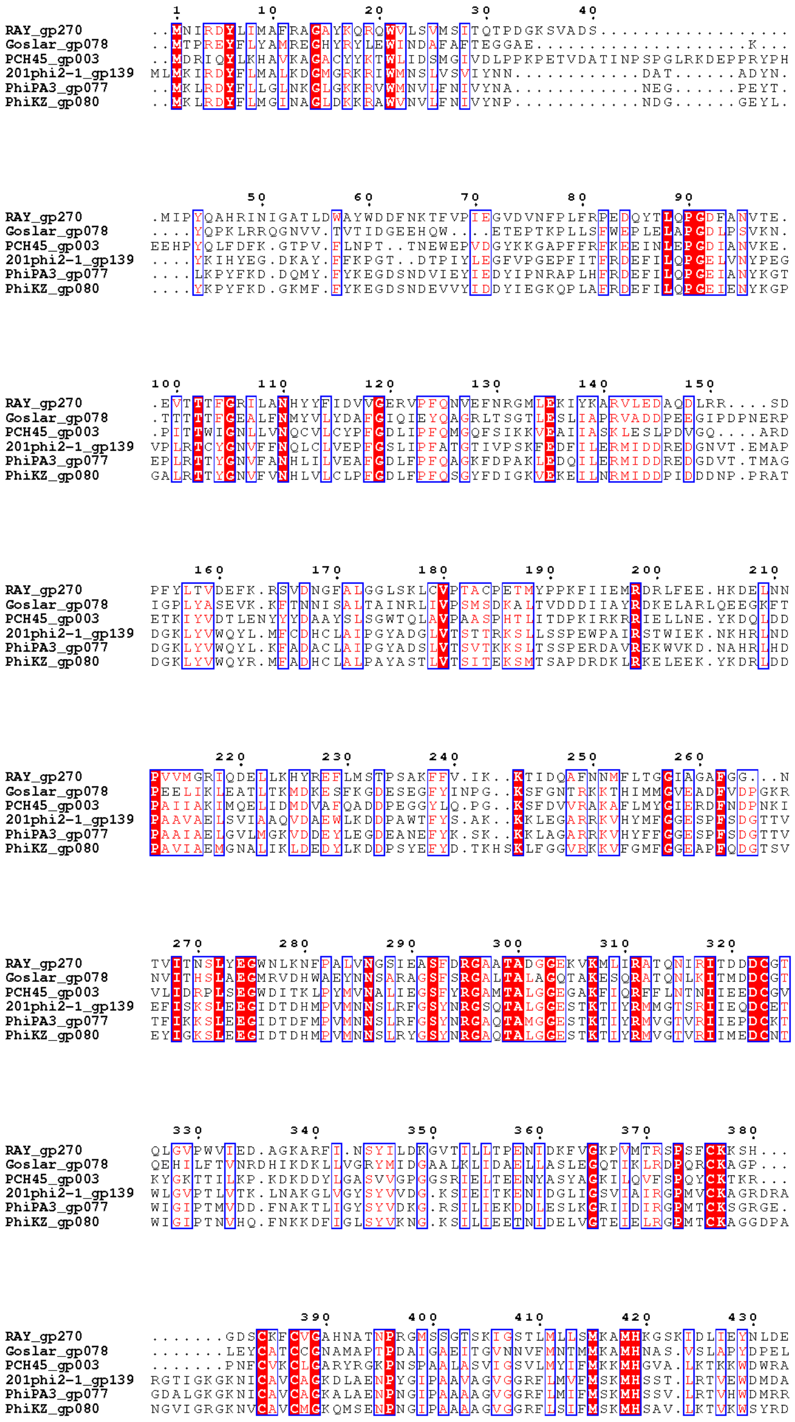
RAY_gp270
Goslar_gp078
PCH45_gp003
201phi2-1_gp139
PhiPA3_gp077
PhiKZ_gp080

| | |
|---|---|
| **RAY_gp270** | VFN |
| **Goslar_gp078** | YIS |
| **PCH45_gp003** | TAE |
| **201phi2-1_gp139** | RLT |
| **PhiPA3_gp077** | RIT |
| **PhiKZ_gp080** | RIS |

**Q**

```
                 1       10        20        30        40        50
Miami_gp214      MLN....YIFGPDVKQG.PTITEINHVNFRNFNDDIQTAYGTSTPGRYMFRRVGSNKFVT
Goslar_gp160     MLDRINRLMNRITVKNQSNNSVHIGGIDAWRLSDDIQRIWGTSRFMKHMFRNFSSSGLSL
RAY_gp131        MFDYLRRALGFINVEEK.NDIITITGFNAPLATRDILKVWKTSKIAGYLFREVTQNKISF
AH06_gp136       MFDYLRRALGFINVEEK.NDVITITGFNAPLATRDILKVWKTSKVAGYLFREVTQNKISF
PhiKZ_gp203      MIDSFRKLMGSLSITET.DQEIIISGFDGAAFIRDINKYWRTTKLATQLFNTVSRRSISF
201phi2-1_gp300  MLDQFRNVFGGVTVKET.NTEIVVSGIRAKDIVRDMDKHWKTTRITQNIFNTVSGNSFSF
PhiPA3_gp233     MFDTFRTLFSGLDVKET.DTEIIISGVAANEIIRDMDKFWKTTKLTGNMFNNVSRRSMSF

                    60        70        80        90        100        110
Miami_gp214      DNFFLLELDHLITKILQSRNTWSSRKNLNELQIKLRTDTWIKDTVTPTAYPIDK...ASF
Goslar_gp160     HNFYLLDFVYILETIVEAKNTRSNKRMLKHLIEVLLQETWLQNTTIEQPAILDK...ARM
RAY_gp131        NSFFAIEVEYILKQLYEHDKTWSDRRGLGKILELLRKNTWMRNLEEQ....YEDIIDLGQ
AH06_gp136       NSFFAIEVEYIFKQLLDHDKTWTDRRGIGKILDLLRKNTWMRNLDEQ....YEDIIDLSQ
PhiKZ_gp203      YKFFAPEIYYMLEAVKNYRSRYISIKTVNAIREAMLQYTWLKNTRPVDTNSVPGRLNFKM
201phi2-1_gp300  YKFFAPEIAYILDGIKKYRHRWTSIRAINGIIEGLNEHTWLKGTIPPDTTTIKGRLDFRK
PhiPA3_gp233     YKFFAPEVMYMLENIKHYRNRWTSIKAINAIREALMEHTWLKGTIPVDPNTVPGRLDLRK

                       120       130       140       150       160
Miami_gp214      LKQFKIKPYDTQWGFLSTYKMVKKSHHLKGLLLDAKAGSGKTFTSLMWTRL.........
Goslar_gp160     NKMLTLSLFDYQDEFIDIYNRNVPKYRLKGYVCAMGAGAGKTITALA.............
RAY_gp131        LKYVKKTPLPEQRNWLNYYNTVIPRYGLRGALLSAAPGAGKTLCSIM.............
AH06_gp136       LKLVKKSPLPEQRNWLNYYNTAVPRYSLRGALLSAAPGAGKTLCSIM.............
PhiKZ_gp203      LDKLTFTPDESQKAYFENYNYRIDQYGLRGDIVAGKPGTGKTF.................
201phi2-1_gp300  LKNLHFTPKPYQMEYFKNYSYRLDQYNLKGDLIAAAAGTGKTYSM...............
PhiPA3_gp233     LQNLHFDAMTYQMEYFQNYSYRLDQYGLKGDLIAAAAGTGKAQPLTSMVKVPGGWKAMGN

Miami_gp214      ............................................................
Goslar_gp160     ............................................................
RAY_gp131        ............................................................
AH06_gp136       ............................................................
PhiKZ_gp203      ............................................................
201phi2-1_gp300  ............................................................
PhiPA3_gp233     IQVGDVVTAWDGTPTKVTGVYPQGKKQTFTVTFKDGRTTKACDEHLWNVYCQDWTRYGGT

Miami_gp214      ............................................................
Goslar_gp160     ............................................................
RAY_gp131        ............................................................
AH06_gp136       ............................................................
PhiKZ_gp203      ............................................................
201phi2-1_gp300  ............................................................
PhiPA3_gp233     GWRVINTLELFGRIQSGKQRLYVQLCKSEEGIDVELPIDPYNLGVILGDGCISSNCVSVT

Miami_gp214      ............................................................
Goslar_gp160     ............................................................
RAY_gp131        ............................................................
AH06_gp136       ............................................................
PhiKZ_gp203      ............................................................
201phi2-1_gp300  ............................................................
PhiPA3_gp233     SGDPQLFTEFAKALPENLELITRDDITMGVINKKGERNPYTSALREMGLLGENSLTKFIP

Miami_gp214      ............................................................
Goslar_gp160     ............................................................
RAY_gp131        ............................................................
AH06_gp136       ............................................................
PhiKZ_gp203      ............................................................
201phi2-1_gp300  ............................................................
PhiPA3_gp233     QNYLMASTAQRLALVQGLMDTDGTVDVNSSLSFSTSSYMLAKQFQYLIRSLGGIAKISFK
```

```
Miami_gp214         ............................................................
Goslar_gp160        ............................................................
RAY_gp131           ............................................................
AH06_gp136          ............................................................
PhiKZ_gp203         ............................................................
201phi2-1_gp300     ............................................................
PhiPA3_gp233        EPTYTYNGVKQYGNMSYRVLVRFDVPSALFRLDRKLARCNDNHQYTENLRLQIKHVNVSD

                                                         170       180
Miami_gp214         ....................................TGNKKHIIICPDAGINTVWKLHME
Goslar_gp160        ................................LSAAMNVDVFVVCCPKNTMRSAWQSDVN
RAY_gp131           ................................TMLCRKKDYVIVIAPKKATRDVWERTIT
AH06_gp136          ................................TMLCRKKDFVIVIAPKKATRDVWERTIT
PhiKZ_gp203         ............................MTMAIAEMVESDIIIVVCEKKSIDLIWKPSII
201phi2-1_gp300     ..............................SALAEMLGAELIVVFCPKAVLESVWVESVN
PhiPA3_gp233        RVECQCIQVEHQDHLYVTDDFIVTHNTYMTSAIAEMRSAELIVVICPKQALETVWLESIP

                    190        200        210        220        230        240
Miami_gp214         EKV..FVDPPKYWSTRQNK.PFDPSCEYFLIHIDYTRNPDFFKILDQVFEAAGKGGVSLV
Goslar_gp160        KAIVDLATETAFWSDTMPPSQLQKSDKYYFLHYES..MGLLNDVMIKH..KRTAKRVLIV
RAY_gp131           TE...LTTEESVWVAEYDQ.PYRKGTKWIVAHYER..LDEVVKMVKELR.L...PNVGII
AH06_gp136          TE...LTTEQDVWVAEYGQ.PYRKGTKWIVAHYEK..LDEIVKLVKEHR.L...PNVGII
PhiKZ_gp203         EM...YKERQKVWSTID.D.KAYNGQRILISHYQA..QDKIIDLLRS.G.IFKGKNITVI
201phi2-1_gp300     EM...FKSPQSIWHSGE.P.MAYEGQRWIMCHYDA..MSKLQELFQDPS.VYQGKKIVTI
PhiPA3_gp233        DM...FKAKQSVWSSANKH.APYKGERWIVCHYDA..MDRLIELLRDQR.VYKGKRVVTI

                       250        260              270        280        290
Miami_gp214         VDESHNFNELSSKQTQN.....IIKA..ADHYPFNDALPMSGTPLKAMAREAYSIFALID
Goslar_gp160        VDESHNFNDK..NSQRSQRLVDLVQTMRQTMNADVHVLFMSGTPVKQMGSEMIPCLGCID
RAY_gp131           LDESHNLNQKTKESIRTNLFVELCQA.....SGSQDIVWASGTALTAMGTEAVPLFRTLI
AH06_gp136          LDESHNLNQKTKESTRTGLFVELCQV.....SGAQDIVWASGTALTAMGTEAVPLFRTLI
PhiKZ_gp203         LDESHNMNDP..NSAQSLKFQQICFM.....SNSNNRLLASGTPVKALGSELVSALRVLD
201phi2-1_gp300     LDESHNMNDP..NSARSMLYQSLVKL.....LDSENNIQGSGTPVKALGAEIITLLRVVD
PhiPA3_gp233        LDESHNLNDP..NSARSLYYQTICKI.....LGSEDNLFASGTPVKALGSELIGLLRVVD

                    300        310        320        330         340        350
Miami_gp214         PFFKGNARERFMKSYGLSRDKLNTLLAHRLGRTRYVIDSL.FDMGE.EPPVEMVPVTVPN
Goslar_gp160        PMFQGQVVDSFKAIYGLTSSRANDILRHRLGFMMHVVPKEAY..RKTRPEVQDVYVKLPN
RAY_gp131           PGFTNDVELAMRKIWGKTATKANDILANRLGIVSFSVPKSKF..MTTKPIEMTVKIKIKN
AH06_gp136          PGFTADVELAMRKIWGKTATKANDILANRLGIVSFSVPKSKF..MSTKPIEATVKVKIKN
PhiKZ_gp203         DLFTNEVEERFKKAFGKETQKGLDIVQHRFGLIAYVIEKKD..TEVLPPRIKAYRIKIPN
201phi2-1_gp300     PLFTPAVEAKFKKMYGKEASKGLDIIRHRLGLVSYKVEKSEADESLLPPIMRPYPIKVPD
PhiPA3_gp233        DMFTPAVEERFRKMYGKEASKGLDIIRHRLGLMSFKVEKKE..TGLQPPIMKPYPIKIPN

                      360        370        380        390        400        410
Miami_gp214         GERFTLKAIRLEMLSYIQNRVKFYEQNMPMFLAFFNHVLDDYESYVKDDNGKLGELVKYK
Goslar_gp160        GSDYTISSIRDDMAIFVRDRAKYYKDNWGHYRKIFDDALDYYRKTIRTDG.ERKELSRYE
RAY_gp131           GAHYTLDNIQRIMMAFIQERFKFYTDNKPAYQKIYDDALGWYEKTLHSPK.EKEDFKLYN
AH06_gp136          GAHYTLDNIQRIMMAFIQERFKFYTENKATYQKIYDDALAWYEKTLHSPK.EKEDFKKYN
PhiKZ_gp203         GSQYTLNAIKRDCEAFIRERVKFYAARRPEDEKYWAKLMAIHENSLKTQA.QKDGFARYK
201phi2-1_gp300     GERFTLPAIRKMMEAFIKERTAYYQKRRDTDMRKWENFIKIARSKLKDRQ.ALIQFDEYL
PhiPA3_gp233        GELFTLPMIKKDMEKFINERWAYYKSRRPNDEKLWNQMLDIHRKSLKTRD.QVKAFEDYL

                      420        430        440        450        460        470
Miami_gp214         QIVNRFRTHGYNNFTDSADSQYAKNVEI.DIEARLRGEQLKQFRNIKSAVKYVGLKIRGE
Goslar_gp160        SIVA.RFQKGYDPVRDKEDAMFCNVFEKRAIMPVLPSDLRVAFKDAKSVIKYVGLKIQGE
RAY_gp131           QYVNTFVTQGFDPYSMAYMSQYCNEYELKRIIPVIPDHMRKTFKDARSVIKYVDLKIKGE
AH06_gp136          DYVEMFIKQGYQAEWMGPYSQYCNDYELKRIIPVIPDHMRKPFKDARSVIKYVDLKIKGE
PhiKZ_gp203         NLIK.IIQKNQDPRYIGEEIKESNQYEKMFIEPTLPRNEIAAFRDIKSVIKYVMLKIQGE
201phi2-1_gp300     HTVH.LISRTPDPRYLGDEMKRANAYEKDIFEKLLPQTMVKDFRDIKSVVKYVNLKIQGE
PhiPA3_gp233        RLVE.LVKRTPDPRYIGEEIKATNVYEKRIFEPTLPRTMIHQFRDIKSVIKYTNLKIQGE
```

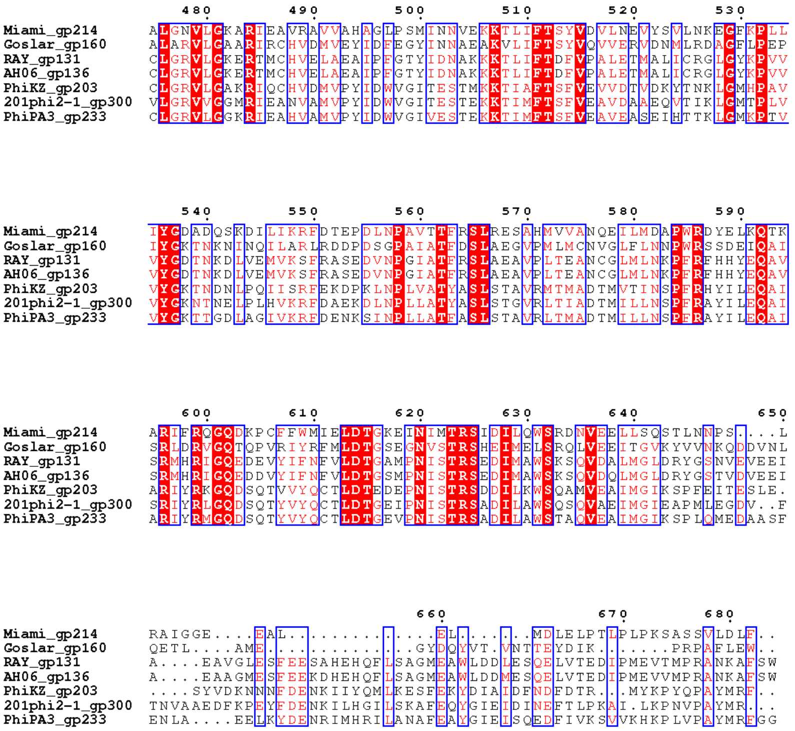
480 490 500 510 520 530
Miami_gp214 ALGNVLGKARIEAVRAVVAHAGLPSMINNVEKKTLIFTSYVDVLNEVYSVLNKEGFKPLL
Goslar_gp160 ALARVLGAARIRCHVDMVEYIDFEGYINNAEAKVLIFTSYVQVVERVDNMLRDAGFLPEP
RAY_gp131 CLGRVLGKERTMCHVELAEAIPFGTYIDNAKKKTLIFTDFVPALETMALICRGLGYKPVV
AH06_gp136 CLGRVLGKERTMCHVELAEAIPFGTYIDNAKKKTLIFTDFVPALETMALICRGLGYKPVV
PhiKZ_gp203 CLGRVLGAKRIQCHVDMVPYIDWVGITESTMKKTIAFTSFVEVVDTVDKYTNKLGMHPAV
201phi2-1_gp300 VLGRVVGGMRIEANVAMVPYIDWVGITESTEKKTIMFTSFVEAVDAAEQVTIKLGMTPLV
PhiPA3_gp233 CLGRVLGGKRIEAHVAMVPYIDWVGIVESTEKKTIMFTSFVEAVEASEIHTTKLGMKPTV
540 550 560 570 580 590
Miami_gp214 IYGDADQSKDILIKRFDTEPDLNPAVTTFRSLRESAHMVVANQEILMDAPWRDYELKQTK
Goslar_gp160 IYGKTNKNINQILARLRDDPDSGPAIATFDSLAEGVPMLMCNVGLFLNNPWRSSDEIQAI
RAY_gp131 VYGDTNKDLVEMVKSFRASEDVNPGIATFRSLAEAVPLTEANCGLMLNKPFRFHHYEQAV
AH06_gp136 VYGDTNKDLVEMVKSFRASEDVNPGIATFRSLAEAVPLTEANCGLMLNKPFRFHHYEQAV
PhiKZ_gp203 VYGKTNDNLPQIISRFEKDPKLNPLVATYASLSTAVRMTMADTMVTINSPFRHYILEQAI
201phi2-1_gp300 VYGKNTNELPLHVKRFDAEKDLNPLLATYASLSTGVRLTIADTMILLNSPFRAYILEQAI
PhiPA3_gp233 VYGKTTGDLAGIVKRFDENKSINPLLATFASLSTAVRLTMADTMILLNSPFRAYILEQAI
600 610 620 630 640 650
Miami_gp214 ARIFRQGQDKPCFFWMIELDTGKEINIMTRSIDILQWSRDNVEELLSQSTLNNPS....L
Goslar_gp160 SRLDRVGQTQPVRIYRFMLDTGSEGNVSTRSHEIMELSRQLVEEITGVKYVVNKQDDVNL
RAY_gp131 SRMHRIGQEDEVYIFNFVLDTGAMPNISTRSEDIMAWSKSQVDALMGLDRYGSNVEVEEI
AH06_gp136 SRMHRIGQEDDVYIFNFVLDTGSMPNISTRSEDIMAWSKSQVDQLMGLDRYGSTVDVEEI
PhiKZ_gp203 ARIYRKGQDSQTVVYQCTLDTEDEPNISTRSDDILKWSQAMVEAIMGIKSPFEITESLE.
201phi2-1_gp300 SRIYRLGQDSQTYVYQCTLDTGEIPNISTRSADILAWSQSQVAEIMGIEAPMLEGDV..F
PhiPA3_gp233 ARIYRMGQDSQTYVYQCTLDTGEVPNISTRSADILAWSTAQVEAIMGIKSPLQMEDAASF
660 670 680
Miami_gp214 RAIGGE....EAL..............EL.......MDLELPTLPLPKSASSVLDLF..
Goslar_gp160 QETL....AME..............GYDQYVT.VNTTEYDIK.......PRPAFLEW..
RAY_gp131 A....EAVGLESFEESAHEHQFLSAGMEAWLDDLESQELVTEDIPMEVTMPRANKAFSW
AH06_gp136 A....EAAGMESFEEKDHEHQFLSAGMEAWLDDMESQELVTEDIPMEVVMPRANKAFSW
PhiKZ_gp203 ....SYVDKNNNFDENKIIYQMLKESFEKYDIAIDFNDFDTR...MYKPYQPAYMRF..
201phi2-1_gp300 TNVAAEDFKPEYFDENKILHGILSKAFEQYGIEIDINEFTLPKAI.LKPNVPAYMR...
PhiPA3_gp233 ENLA....EELKYDENRIMHRILANAFEAYGIEISQEDFIVKSVVKHKPLVPAYMRFGG

**R**

```
              1        10         20        30        40        50        60
AH06_gp315    MTTMSELGPAHTTLLAEEKDVQKDAATDATETALAVLKKTSKSKPEKDDEDEPTISFGAE
RAY_gp299     MSTMTELGPSQGTLLADEKAVHDDAATEATEVALHVLKKIGKKKEKDDEDEEVVLSFGSE
Miami_gp025   ....................................................MSTPYTQK

                       70                 80        90        100       110
AH06_gp315    GYTMLTSMSFDLTE.NTDLA......IQELLDKISKIVIEANGNLTDKDLEAIKN.VGNE
RAY_gp299     GYAMLTSMSFDLTE.NSDLA......VQELLDKITTIVIEANGNLTQKDLEKIKS.VGNE
Miami_gp025   GMSMSLAEEYEGDKKKLDRYIEGTPDNVERYKKVAAVIGKEPGHVTDDK.DTIDNTAGTE

                                 120       130       140       150
AH06_gp315    .............ALLDAGNEDYKEVMNLVMRKIMQFIRWAINQGKEGIKRISDRLSRLS
RAY_gp299     .............SLLGDGNEDYKEVVTMVMKRIMQFIRWAINQGKEGIKRVADRLSRLS
Miami_gp025   EFDIDEAVLNFPEDENVAGMEDFVEFAKKAIVRLMRMIKNAAIFIYELVFNKVSRIKHIA

            160       170       180       190       200       210
AH06_gp315    VKAMYVERKIDVSADNSLPNDKFVLPRTYPLLMMANKPPANAMEVINVLNRTKYLFTTLH
RAY_gp299     VKTMYVERKIDISTDNSLPTDKFVLPRSYPMLMMVNKPPANAMEVINAVNRTKYLFTTLH
Miami_gp025   RKAEYQLRT.....QSLIPT.LVPYPKGTRKIFIGPALQTNANWTLKSIQQVQLFYTRLM

            220       230       240       250       260       270
AH06_gp315    NDYQNFQNLFKSAVATGSRADTLEMINSYLTSLASKLSARPNPLFDNRLSF.........
RAY_gp299     NDYQGFQALFKAAVATGSRADTLEMINNYLNGLISKLSARPNPLFDNRNTF.........
Miami_gp025   KAHGN...LLDAA.KSG..................NWRNNPNFIFETAATFANEICQNHH

                                280       290       300       310       320
AH06_gp315    ......NQLPGG..YRLVFSQGQAFADCSATLTRTSERYDMAPAVQRPDKASLMRLVAEV
RAY_gp299     ......NQLPGG..YRLVFSEGQSFADCGATILRTPEKYEAAPAVQRPDKASLVRLVSEI
Miami_gp025   SDYSETQVLPGGKIYRVNYSKGSGKNRPGVSLSQKL.SVQLKDKSFKPDRAIADDLMTAL

                    330          340       350        360       370
AH06_gp315    KTYLRIINEVY...GRVSGRLEADFRAIVKSAEREVKS.FDSTADIRTASTTIEWFTEQQ
RAY_gp299     KTYLRVINEVY...GKVSSRLEVDFRNIVKAAEKEVKG.FDSTADIRTASTTIEWFTEQQ
Miami_gp025   QGFADKIGNDHRSQTSTSRKMEAAIR........DLERYYVDPEAKRHAAAYFTWLIDFQ

             380       390       400       410       420       430
AH06_gp315    SKLYTRSMMLSCTVLNASLDYCLSAIGAKPAAGTESFDITETSSISYAIESLDEQLERLD
RAY_gp299     SRLYTRSMMLSCTVLNAALDYCLGAIGAKPAVGTESFDILDTSSMGYAIESLGEQMERLD
Miami_gp025   R...........TVLSTGLQYVFGTLFGYAEFISANVK......................

             440       450       460       470       480       490
AH06_gp315    AGLCSLEIDSRSMQSIADVEELVDVDNDQVINLLMNQTPASYYNPLYGISFAGLQGVLEE
RAY_gp299     AGLCELEIDARTMQSITDVKEMVDVDNDHVIQLLMQQRPSSYFNPPDGLSEYSLGDAFNG
Miami_gp025   ............................................................

             500       510       520       530       540                550
AH06_gp315    KKGAEYIASRLGGIVDITKQLDNTAEVIKQIMTDLPDGEVGAVKPGLK......PTVADV
RAY_gp299     SATARYIGRRLTSIVNLTNQLSGTTDLMKGLLAEMPTGEIK...PGSDADVIERNSLLDR
Miami_gp025   ............................................................

                    560       570       580       590       600       610
AH06_gp315    EFTSDHPWCAFLHRVDRQSIYASDVVRYVGDTYEKLRGVNNTLSVLAEQMTKTVVDGEMN
RAY_gp299     EMFEGHPLCAFLHRVDREGIYATDVIRYVTDTYEKLRQVRTTLAVQAEQMTLTVADGEMN
Miami_gp025   ............................................................
```

```
                      620        630        640        650        660        670
AH06_gp315     VSLLKEYMFNAPTEPFTASSLCGGFTISPVAEKLGKLVVRGYVLESPPPLKPMVTIHRPS
RAY_gp299      VSLLKEFLFNAPQEPFTDSVLCGGFRLQPVAEKLGELVIHGAVMDSPPPLKPMTTIHRPS
Miami_gp025    ............................................................

                      680        690        700        710        720        730
AH06_gp315     YDEYNEINSLAARFNAEQARLAEITRLLQIGTGHLRYITASVADNLTADGLKGGGDGWFS
RAY_gp299      YDEYNGVNQLASLFGNELRELAKIAERLQIGTGYLRYITTSVADNLNEDGLKGGGDGWFN
Miami_gp025    ............................................................

                      740        750        760        770        780
AH06_gp315     TALEYLAISARQYRWMYRLTVQLAIYQRTMISALRQYNNEGGWDGDKQ
RAY_gp299      TALEYLAVSARQYRWMYRLTVQLAVYEHTMINALRHYNNSGGWHD...
Miami_gp025    ................................................
```

**Figure S4. Multiple sequence alignments, Related to Figures 3, 4, and 5.** (A) Multiple sequence alignment of RAY gp222 with chimallin from previously published nucleus-forming phages. (B) Multiple sequence alignment of RAY gp220 and DNA polymerases from previously published nucleus-forming phages. (C) Multiple sequence alignment of RAY gp315 with DNA helicases from previously published nucleus-forming phages. (D) Multiple sequence alignments of RAY gp002 (i), gp248 (ii), gp223 (iii), and gp249 (iv) with nvRNAP subunits from previously published nucleus-forming phages. These RAY proteins are homologs of known phage msRNAP subunits ΦKZ gp123, gp71-73, gp55-56.1, and gp74, respectively [88]. (E) Multiple sequence alignment of RAY gp150 with UvsX from previously published nucleus-forming phages. UvsX is a RecA homolog. (F) Multiple sequence alignment of RAY gp153 with DprA from previously published nucleus-forming phage Goslar and RAY close relative AH06. DprA is a RecA-associated protein involved in recombination. Since it is not part of the core genome, it was not present in most of the previously-studied nucleus-forming phages used for other MSA analysis. (G) Multiple sequence alignment of RAY gp250 with SF2 helicase homologs from previously published nucleus-forming phages. RAY gp250 localized in the phage nucleus (Fig. 4B) as opposed to RAY gp131, another SF2 helicase homolog which localized similarly to capsids (Fig. 5B), leading us to call gp250 and its homologs the "non-virion" SF2 helicase. (H) Multiple sequence alignment of RAY gp116 with HslUV-like protease homologs from RAY's close relative AH06 and its distant relative Miami. HslUV is not part of the core genome and was not present in any of the previously-studied nucleus-forming phages used for other MSA analysis. (I) Multiple sequence alignment of RAY gp311 with TMK from previously published nucleus-forming phages. (J) Multiple sequence alignment of RAY gp039 with SspB from previously published nucleus-forming phages and RAY close relative AH06. Since SspB is not part of the core genome, it was not present in some of the previously-studied nucleus-forming phages used for other MSA analysis. (K) Multiple sequence alignment of RAY gp048 with RtcB from previously RAY close relative AH06 and ΦPA3 close relative *Pseudomonas* phage PA1C. RtcB is not part of the core genome and was not present in any of the previously-studied nucleus-forming phages used for other MSA analysis. However, despite its uncommon occurrence in Chimalliviridae accessory genomes, its amino acid sequence conservation is remarkably high, even between distantly-related phages like RAY and PA1C. (L) Multiple sequence alignment of RAY gp064 with related exonucleases from previously published nucleus-forming phage Goslar and RAY close relative AH06. Since this exonuclease is not part of the core genome, it was not present in most of the previously-studied nucleus-forming

phages used for other MSA analysis. (M) Multiple sequence alignment of RAY gp094 with XRE superfamily transcriptional repressors from previously published nucleus-forming phage PCH45, RAY close relative AH06, and RAY distant relative Miami. Since this transcriptional regulator is not part of the core genome, it was not present in most of the previously-studied nucleus-forming phages used for other MSA analysis. (N) Multiple sequence alignment of RAY gp179 with tail sheath proteins from previously published nucleus-forming phages. (O) Multiple sequence alignment of RAY gp317 with MCP from previously published nucleus-forming phages. (P) Multiple sequence alignments of RAY gp164 (i), gp154 (ii), gp163 (iii), and gp270 (iv) with vRNAP subunits from previously published nucleus-forming phages. These RAY proteins are homologs of known phage msRNAP subunits ΦKZ gp178, gp149, gp180, and gp80, respectively [17]. (Q) Multiple sequence alignment of RAY gp131 with SF2 helicase homologs from previously published nucleus-forming phages, as well as RAY close relative AH06 and RAY distant relative Miami. RAY gp131 localized similarly to capsids (Fig. 5B) and was seen in virion mass spec experiments [16] as opposed to RAY gp250, another SF2 helicase homolog which localized in the phage nucleus (Fig. 4B), leading us to call gp131 and its homologs the “virion” SF2 helicase. While this virion SF2 helicase is not part of the core genome (and not found in PCH45), it is widely conserved in a majority of Chimalliviridae. (R) Multiple sequence alignment of RAY gp299 with homologs from RAY’s close relative AH06 and its distant relative Miami. This protein is found in capsids [16] but is not part of the core genome and was not present in any of the previously-studied nucleus-forming phages used for other MSA analysis. As there is very little information about this protein, we are not able to make any hypotheses concerning its potential function, and it remains an area of future study.

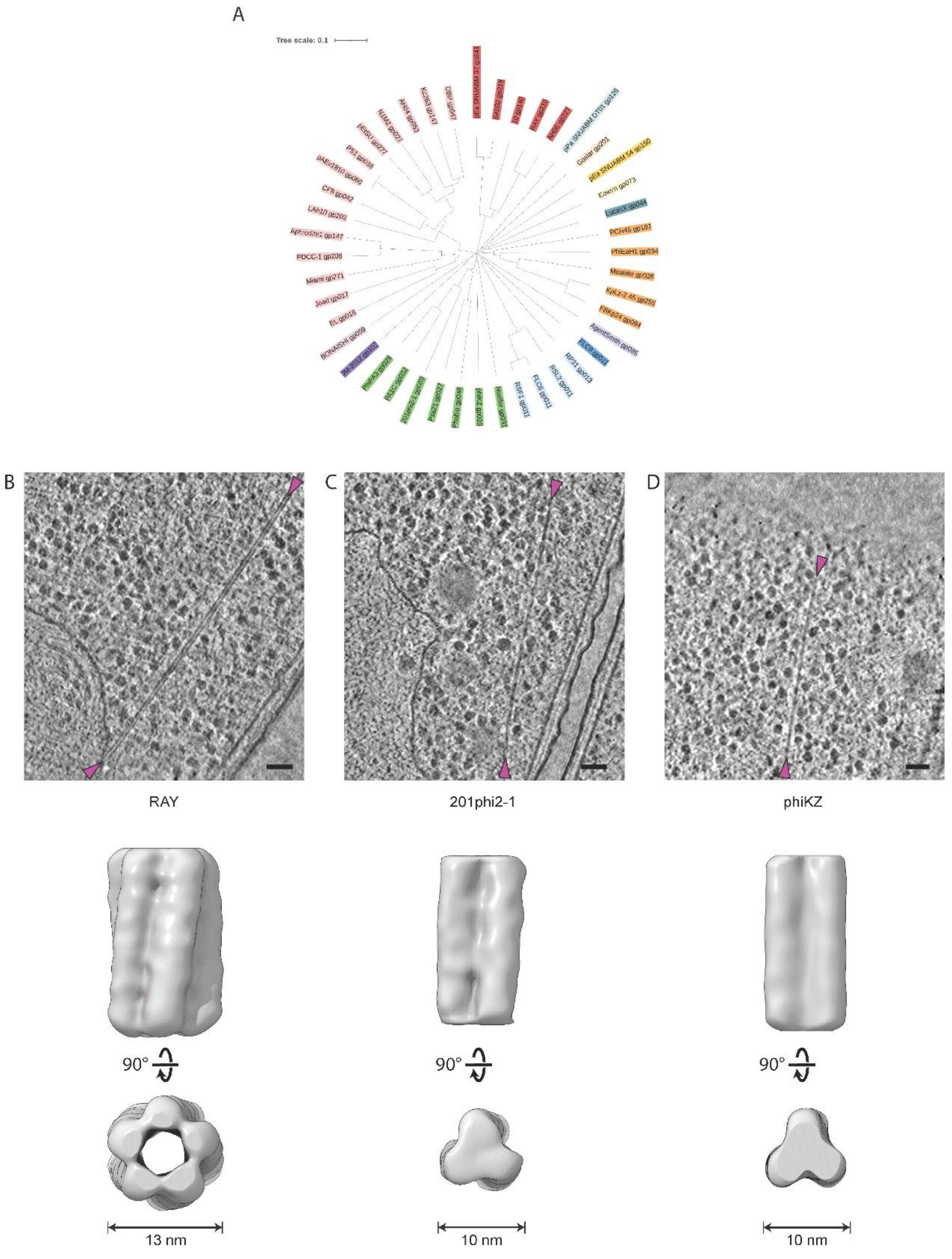


**Figure S5. PhuZ protein phylogeny and filament structural comparison, Related to Figure 6.** (A) A protein tree of PhuZ homologs from Chimalliviridae. While PhuZ is not a member of the core

genome, it is present in a majority of Chimalliviridae. Chimalliviridae are color-coded by predicted genus as in Figure 1. (B,C,D) Top, tomographic slices from jumbo phage infected host cells with the ends of putative PhuZ filaments marked with magenta arrows. Bottom, orthogonal views of subtomogram averages from the corresponding tomographic datasets for (B) RAY, (C) 201ϕ2-1 (EMPIAR-10859), and (D) ΦKZ. Scale bars: 50 nm.

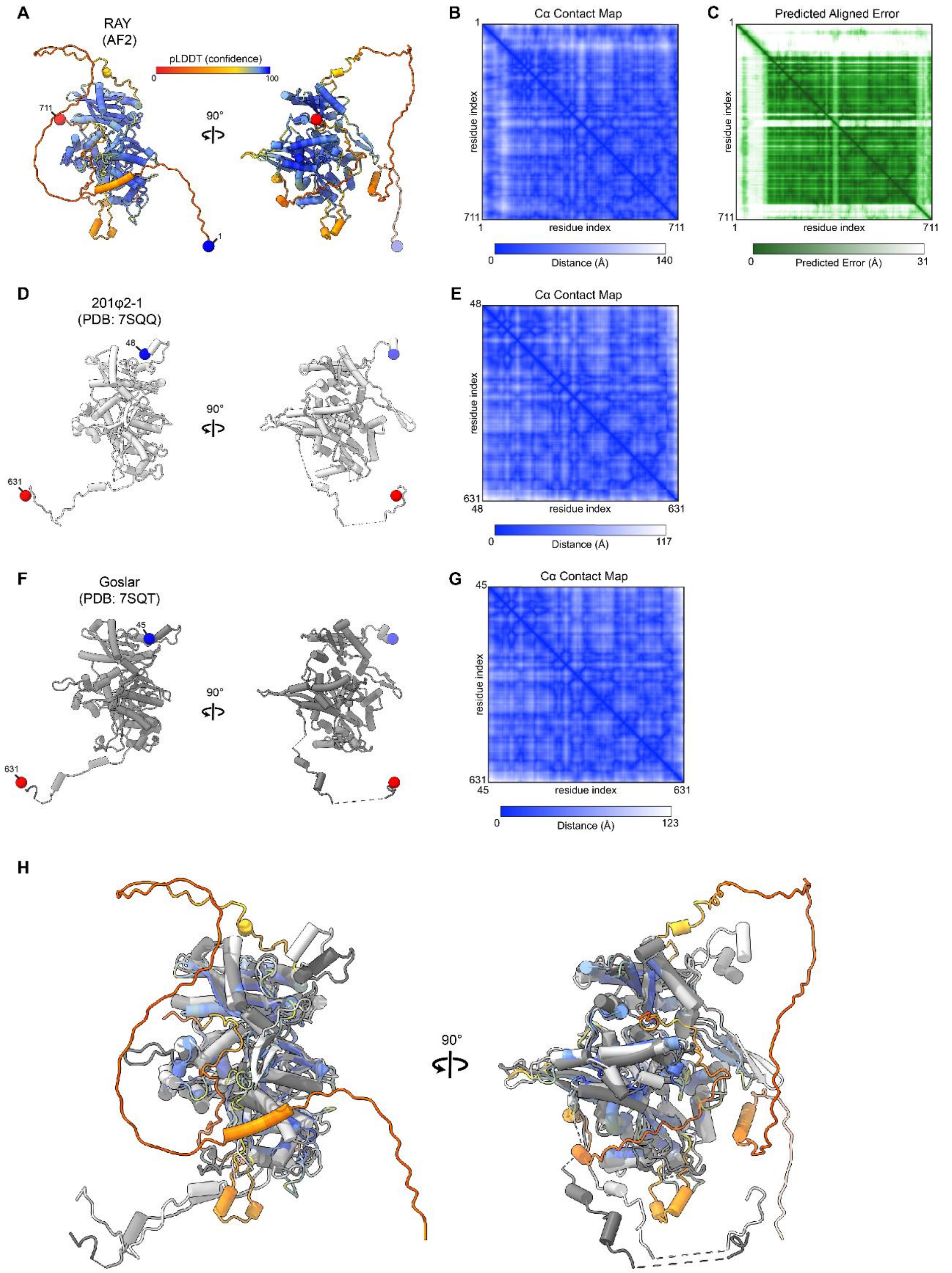


**Figure S6. Structural comparison of RAY, 201ϕ2-1, and Goslar chimallin protomers, Related to Figure 7.** (A) Orthogonal views of the AF2 predicted RAY chimallin protomer model colored by pLDDT. N- and C-termini are labeled and shown as blue and red spheres, respectively. (B) Pairwise C-alpha distance map and (C) predicted aligned error plot for the RAY chimallin protomer model. (D)

Orthogonal views of the 201ϕ2-1 chimallin protomer model (PDB ID: 7SQQ) colored white with termini indicated as in A and (E) corresponding pairwise C-alpha distance map. (F) Orthogonal views of the Goslar chimallin protomer model (PDB ID: 7SQT) colored gray with termini indicated as in A and (G) corresponding pairwise C-alpha distance map. (H) Orthogonal views of superimposed chimallin protomer models from A,D, and F.

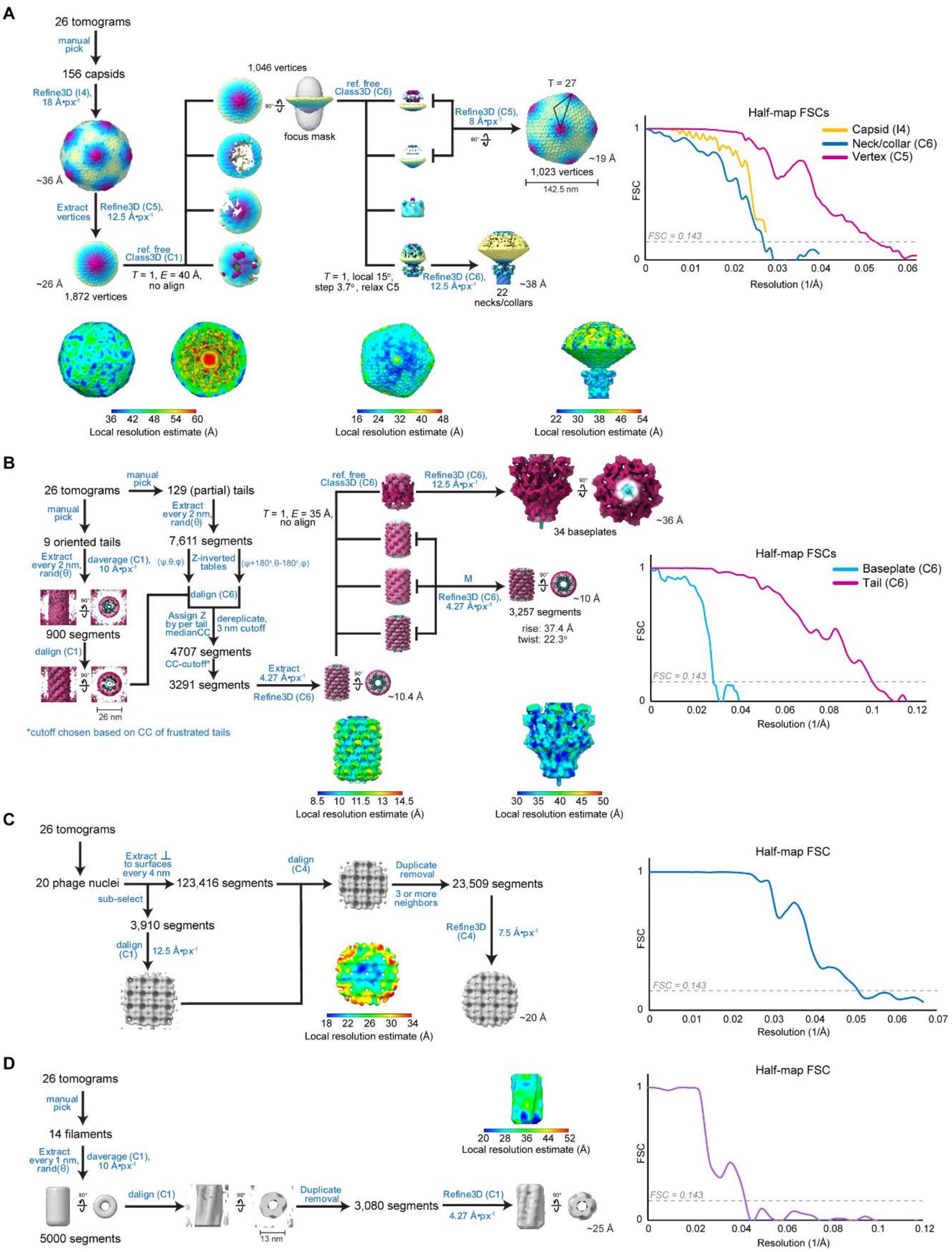
A
26 tomograms
manual pick
156 capsids
Refine3D (I4), 18 Å•px⁻¹
~36 Å
Extract vertices
Refine3D (C5), 12.5 Å•px⁻¹
~26 Å
1,872 vertices
ref. free Class3D (C1)
T = 1, E = 40 Å, no align
1,046 vertices
focus mask
ref. free Class3D (C6)
T = 1, local 15°, step 3.7°, relax C5
Refine3D (C5), 8 Å•px⁻¹
T = 27
~19 Å
1,023 vertices
142.5 nm
Refine3D (C6), 12.5 Å•px⁻¹
~38 Å
22 necks/collars
Half-map FSCs
Capsid (I4)
Neck/collar (C6)
Vertex (C5)
FSC = 0.143
FSC
Resolution (1/Å)
Local resolution estimate (Å)
B
26 tomograms
manual pick
129 (partial) tails
Extract every 2 nm, rand(θ)
7,611 segments
9 oriented tails
daverage (C1), 10 Å•px⁻¹
900 segments
dalign (C1)
26 nm
Z-inverted tables
dalign (C6)
Assign Z by per tail medianCC
dereplicate, 3 nm cutoff
4707 segments
CC-cutoff*
3291 segments
Extract 4.27 Å•px⁻¹
Refine3D (C6)
~10.4 Å
T = 1, E = 35 Å, no align
ref. free Class3D (C6)
Refine3D (C6), 12.5 Å•px⁻¹
~36 Å
34 baseplates
M
Refine3D (C6), 4.27 Å•px⁻¹
~10 Å
3,257 segments
rise: 37.4 Å
twist: 22.3°
*cutoff chosen based on CC of frustrated tails
Half-map FSCs
Baseplate (C6)
Tail (C6)
FSC = 0.143
Resolution (1/Å)
Local resolution estimate (Å)
C
26 tomograms
20 phage nuclei
Extract ⊥ to surfaces every 4 nm
123,416 segments
sub-select
3,910 segments
dalign (C1)
12.5 Å•px⁻¹
dalign (C4)
Duplicate removal
3 or more neighbors
23,509 segments
Refine3D (C4)
7.5 Å•px⁻¹
~20 Å
Local resolution estimate (Å)
Half-map FSC
FSC = 0.143
Resolution (1/Å)
D
26 tomograms
manual pick
14 filaments
Extract every 1 nm, rand(θ)
daverage (C1), 10 Å•px⁻¹
5000 segments
dalign (C1)
13 nm
Duplicate removal
3,080 segments
Refine3D (C1)
4.27 Å•px⁻¹
~25 Å
Local resolution estimate (Å)
Half-map FSC
FSC = 0.143
Resolution (1/Å)

**Figure S7. Subtomogram analysis workflows of RAY components, Related to Figure 7.** Workflow schematics, local resolution estimates, and half-map Fourier shell correlation curves for the RAY (A) capsid and collar, (B) tail sheath and baseplate, (C) chimallin, (D) putative PhuZ filament.

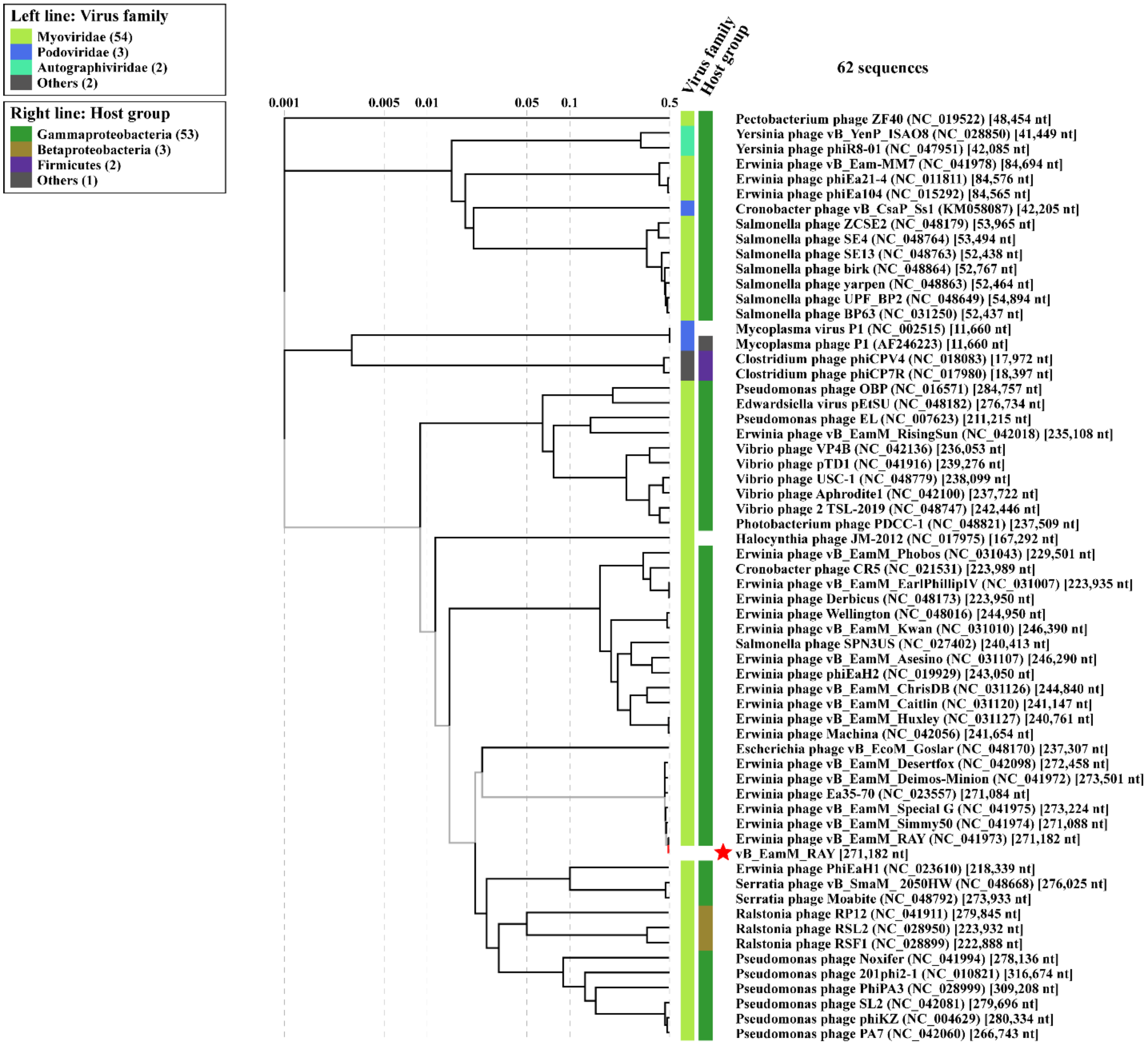


**Figure S8. ViPTree-predicted relatives of RAY, related to STAR Methods.** Relatives of RAY were attempted to be found using ViPTree to identify similar genomes. This whole genome tree represents possible RAY relatives found by ViPTree[64].
