# Supplementary material for: Identifying the core genome of the nucleus-forming bacteriophage family and characterization of Erwinia phage RAY
Source: Cell Rep. Author manuscript; Available in PMC 2023 Jun 27. (PMC10299810; doi:10.1016/j.celrep.2023.112432)
Supplement: 6 [file NIHMS1905355-supplement-6.pdf]

Cell Reports

Article

Identifying the core genome of the nucleus-forming bacteriophage family and characterization of *Erwinia* phage RAY

Graphical abstract

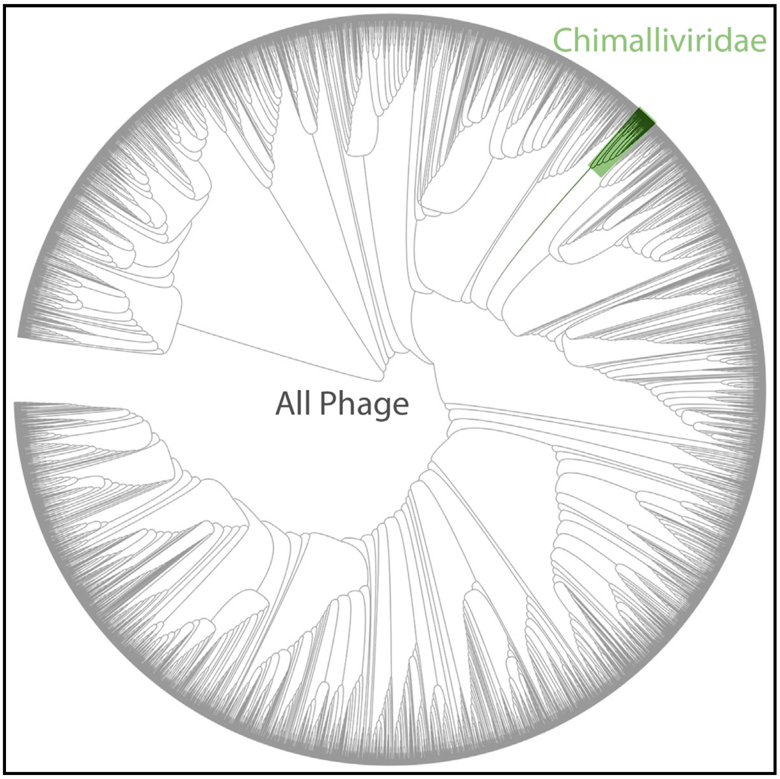

Highlights

- *Erwinia* phage RAY is a nucleus-forming phage
- Nucleus-forming phages cluster phylogenetically and share a core genome, including ChmA
- We propose that all phage encoding ChmA form a family of nucleus-forming phages

Authors

Amy Prichard, Jina Lee,
Thomas G. Laughlin, ..., Elizabeth Villa,
Rachel Dutton, Joe Pogliano

Correspondence

jpogliano@ucsd.edu

In brief

Some bacteriophages have been shown to make a nucleus-like structure (the phage nucleus) during their infection cycle. These phages are widespread and infect numerous hosts. Prichard et al. find that these phages cluster together in phylogenetic trees, suggesting a common ancestry of all known nucleus-forming phages.

Prichard et al., 2023, Cell Reports *42*, 112432
May 30, 2023 © 2023 The Authors.
https://doi.org/10.1016/j.celrep.2023.112432

CellPress

Cell Reports

CellPress
OPEN ACCESS

Article

Identifying the core genome of the nucleus-forming bacteriophage family and characterization of *Erwinia* phage RAY

Amy Prichard,[1] Jina Lee,[1] Thomas G. Laughlin,[1] Amber Lee,[1] Kyle P. Thomas,[1] Annika E. Sy,[1] Tara Spencer,[1] Aileen Asavavimol,[1] Allison Cafferata,[1] Mia Cameron,[1] Nicholas Chiu,[1] Demyan Davydov,[1] Isha Desai,[1] Gabriel Diaz,[1] Melissa Guereca,[1] Kiley Hearst,[1] Leyi Huang,[1] Emily Jacobs,[1] Annika Johnson,[1] Samuel Kahn,[1] Ryan Koch,[1] Adamari Martinez,[1] Meliné Norquist,[1] Tyler Pau,[1] Gino Prasad,[1] Katrina Saam,[1] Milan Sandhu,[1] Angel Jose Sarabia,[1] Siena Schumaker,[1] Aaron Sonin,[1] Ariya Uyeno,[1] Alison Zhao,[1] Kevin D. Corbett,[2] Kit Pogliano,[1] Justin Meyer,[1] Julianne H. Grose,[3] Elizabeth Villa,[1,4] Rachel Dutton,[1] and Joe Pogliano[1,5,*]

[1]School of Biological Sciences, University of California San Diego, La Jolla, CA 92093, USA
[2]Department of Cellular and Molecular Medicine, University of California San Diego, La Jolla, CA 92093, USA
[3]Department of Microbiology and Molecular Biology, Brigham Young University, Provo, UT 84602, USA
[4]Howard Hughes Medical Institute, University of California San Diego, La Jolla, CA 92093, USA
[5]Lead contact
*Correspondence: jpogliano@ucsd.edu
https://doi.org/10.1016/j.celrep.2023.112432

SUMMARY

We recently discovered that some bacteriophages establish a nucleus-like replication compartment (phage nucleus), but the core genes that define nucleus-based phage replication and their phylogenetic distribution were still to be determined. Here, we show that phages encoding the major phage nucleus protein chimallin share 72 conserved genes encoded within seven gene blocks. Of these, 21 core genes are unique to nucleus-forming phage, and all but one of these genes encode proteins of unknown function. We propose that these phages comprise a novel viral family we term Chimalliviridae. Fluorescence microscopy and cryoelectron tomography studies of *Erwinia* phage vB_EamM_RAY confirm that many of the key steps of nucleus-based replication are conserved among diverse chimalliviruses and reveal variations on this replication mechanism. This work expands our understanding of phage nucleus and PhuZ spindle diversity and function, providing a roadmap for identifying key mechanisms underlying nucleus-based phage replication.

INTRODUCTION

The ability to establish and maintain subcellular organization is fundamental to cellular function. Even many viruses remodel their host cells, setting up their own complex compartments to suit their unique needs for viral replication.[1–6] We recently discovered that some bacteriophages replicate by creating a nucleus-like proteinaceous structure (the phage nucleus) that compartmentalizes the bacterial host cell during phage infection much in the same way a membranous nucleus compartmentalizes a eukaryotic cell.[6–10] Although the phage nucleus is structurally different from the eukaryotic nucleus, it performs many similar functions. The phage nucleus, which is made of a protein called chimallin (ChmA), separates transcription from translation, exports mRNA, selectively imports proteins, and shields the phage DNA from cytoplasmic nucleases.[7–13]

Nucleus-forming phages belong to a larger group of phages that encode rifampicin-resistant multi-subunit RNA polymerases (msRNAP).[14–22] Nucleus-forming phages, typified by *Pseudomonas aeruginosa* phage ΦKZ, encode two msRNAPs composed of 4–5 subunits. One of these msRNAPs is the virion RNAP (vRNAP), which is packaged within the capsid and is likely injected into the cell along with the DNA upon infection.[15–17] The non-vRNAP (nvRNAP) is expressed by the vRNAP during infection.[17,19,20] While all nucleus-forming phages studied to date encode these unique msRNAPs, this feature is also observed in several jumbo phages that do not form phage nuclei.[14]

Another feature shared by currently characterized nucleus-forming phages is a phage-encoded tubulin homolog called PhuZ. Members of the PhuZ family studied thus far share a common filament structure and assembly mechanism, producing a dynamic three-stranded filament.[23,24] In *Pseudomonas* phages 201φ2-1, ΦKZ, and ΦPA3, the phage nucleus is centered at the host midcell and rotated by a bipolar PhuZ spindle,[7,8,25–27] and in *Escherichia coli* phage Goslar, the PhuZ filaments form a vortex that rotates the nucleus without positioning it at midcell.[10] Capsids assemble on the membrane and traffic along PhuZ filaments to reach the nucleus in the *Pseudomonas* phages.[7,8,27] Capsids then dock to initiate DNA packaging at the surface of the phage nucleus.[7,8,10,27] Filled capsids assemble with tails, forming cytoplasmic bouquet structures at rates varying from phage to phage prior to cell lysis.[10,28]

These recent discoveries prompt intriguing questions: how widespread is nucleus formation among phages that infect

This is an open access article under the CC BY license (http://creativecommons.org/licenses/by/4.0/).

different hosts? Do all nucleus-forming phages share a common set of core genes? If so, which viral genes are part of the core genome versus the accessory genome? To answer these questions, we began by studying *Erwinia* phage vB_EamM_RAY (hereafter called RAY), which encodes a chimallin homolog. RAY infects *Erwinia amylovora*, an important agricultural pathogen and the causative agent of fire blight.[29,30] Many chimallin-encoding *Erwinia* phages are distantly related to each other and to known nucleus-forming phages, making *Erwinia amylovora* an enticing host for studying possible nucleus formation in diverse phages.[29,31,32]

Core genomes of phage and bacteria define their conserved components, while accessory genomes provide genes that are specific to the individual species, strains, or variants.[33–37] While genes required for making viral particles such as virion structural components, polymerases, and chimallin can be identified bioinformatically by comparison to sequence and structural homologs, the genes that comprise the nucleus-forming phage core genome have not been previously identified. Studying the core genome will allow us to identify and understand the genes required for the nucleus-based replication mechanism, including nuclear shell assembly, PhuZ spindle formation, mRNA export, selective protein import, capsid docking, and bouquet formation. While previous work identified some core genome components common of chimallin-encoding *Pseudomonas* phages 201φ2-1, ΦKZ, ΦPA3, EL, and OBP and *Vibrio* phage JM-2012, this work predates the discovery of the phage nucleus replication mechanism, and only 6 phage genomes were included at the time.[38] Additionally, core genomes of large groups of jumbo phages have been analyzed but did not focus on viral replication mechanisms to identify the core genomes of phage that replicate by forming a nucleus.[39]

Here, we investigated the conservation of nucleus-based phage replication by focusing on *Erwinia* phage RAY and comparing it with previously studied nucleus-forming phages. We analyzed its phylogenetic relationships with other phages, identified the core and accessory genes, and used fluorescence microscopy and bioinformatics to characterize the putative functions of 15 core and 9 accessory RAY proteins. We show that RAY is a nucleus-forming phage by fluorescence microscopy and cryoelectron tomography and describe how its replication mechanism varies compared with previously characterized nucleus-forming phages.

RESULTS

Defining the core genome of chimallin-encoding phages

To identify the key, conserved genes required to replicate via the phage nucleus pathway, we first identified all phages in the NCBI database that encode a homolog of the major nuclear shell protein chimallin (ChmA). We found 66 unique phages encoding chimallin homologs and made whole genome trees to compare them (Figures 1A and 1B), showing that all 66 phages form a monophyletic group (Figure 1A) when compared with phages that encode msRNAPs and other related phages (see STAR Methods). Members of this clade infect a wide range of Gram-negative bacteria and one Gram-positive *Bacillus* species, and they vary greatly in genome size, from 167 to 322 kb. This suggests that the chimallin-encoding group of phages arose only once from a common ancestor, has a wide host range, and is not restricted only to "jumbo" phages larger than 200 kb. We created phylogenetic trees based on 6 conserved proteins. The whole genome tree (Figure 1B) is generally congruent with all of the protein-based trees, including chimallin (Figure 1C), major capsid protein (Figure 1D), terminase large subunit (Figure S1A), DNA polymerase (Figure S1B), replicative helicase (Figure S1C), and an RNAP subunit (Figure S1D). Each of these trees contained 16 groups of related phage species composed of the same individuals (color coded in Figures 1, S1, and S2). The phylogeny of the groups closely matched the whole genome tree (Figure S2). The similarity across different phylogenetic trees suggests that these proteins have been co-evolving, with little evidence of horizontal gene transfer between divergent phages for these proteins. This supports previous findings that the phage nucleus may help to limit recombination between nucleus-forming phages.[40] We then determined the set of core genes that are conserved among chimallin-encoding phages and whether this core genome is conserved in other msRNAP-encoding phages.

We classified a gene as core if it is present in more than 90% of the chimallin-encoding phages to accommodate potential sequencing errors and variability due to sampling[42] and found 72 conserved genes (Figures 2A–2C) (see STAR Methods for details). The 72 core genes included the major capsid protein, terminase large subunit, and msRNAP subunits, but surprisingly, the majority of the highly conserved core genes (53, 73.6%) had no predicted function. A PhuZ homolog was present in only 66.2% of chimallin-encoding phages, which is notable given its well-characterized role in nucleus-based phage replication yet consistent with PhuZ function being dispensable for replication.[10,25,27,40,43] The core genes identified here likely contain many of the key proteins required for nucleus-forming phages to replicate.

The core genes occur in seven distinct blocks (of three or more genes) whose general order is conserved across *Pseudomonas aeruginosa* phage ΦKZ, *Escherichia coli* phage vB_EcoM_Goslar (Goslar), and *Erwinia amylovora* phage RAY (Figure 2C). These three phages were chosen for our analysis because they represent a diverse group of chimallin-encoding phages and are well studied (ΦKZ), infect highly tractable hosts (Goslar), or formed the basis of our bioinformatic studies (RAY). The core genome blocks are often rich with certain types of genes, such as block 7, which contains the terminase and a handful of virion structural genes, and block 5, which contains nineteen genes, including seven structural genes, the major capsid protein, one helicase, and an RNAP β subunit (Table S3). Gene order within the blocks is also conserved across chimallin-encoding phages. Upon mapping the level of conservation across the RAY, ΦKZ, and Goslar genomes, we found that there is a conserved region that is dense with core genome blocks and a variable region with putative accessory genes specific to each phage (Figures 2A and 2B). The majority of RAY's 317 genes are either part of this core conserved genome (23%, 72 genes) or are accessory genes only detectable in 10% or fewer of the phages we used in our analysis (178 genes, 56%) (Figure 2C). This is a similar organization to other previously reported phage core genomes.[35,36]

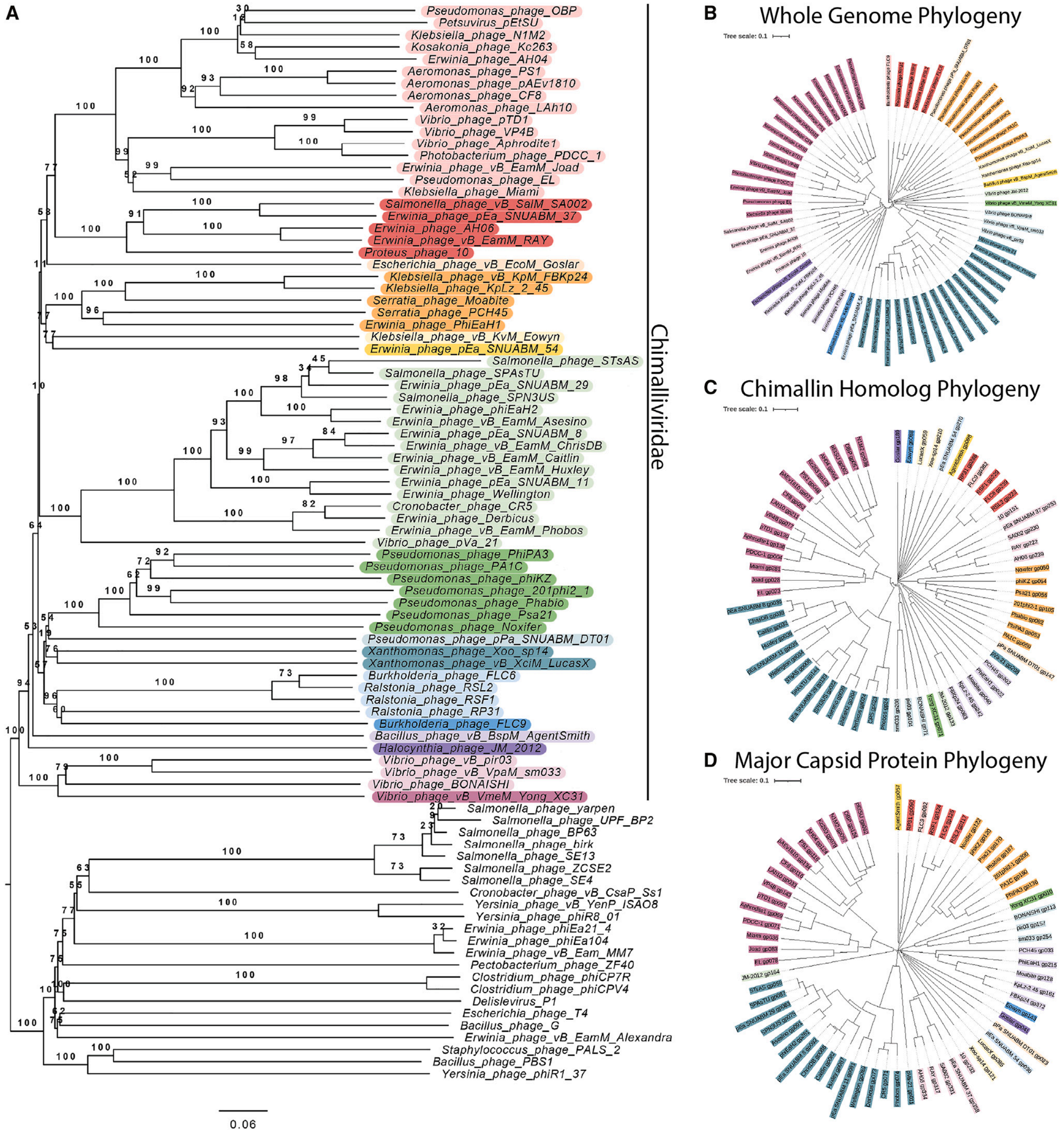

Figure 1. Phylogenetic comparison of Chimalliviridae

(A) Phylogenetic tree of the Chimalliviridae and related phages. The Chimalliviridae appear to form one clade, separate from msRNAP-encoding phages, ViPTree-predicted relatives, and other phages with large genomes color coded by predicted genus (Table S2) to improve readability.

(B) Phylogenetic tree based on whole-genome comparison of 66 representative chimallin-encoding phages (Table S1).

(C and D) Phylogenetic tree based on the protein sequences of the (C) chimallin homologs and (D) major capsid proteins from the 66 phages used in our analysis. Trees were colored in iTOL[41] by predicted genus (Table S2). Some of these predicted genera are not fully consistent with current ICTV classification, but for maximum readability and consistency of the colors, only the genera predicted by VICTOR are color coded.

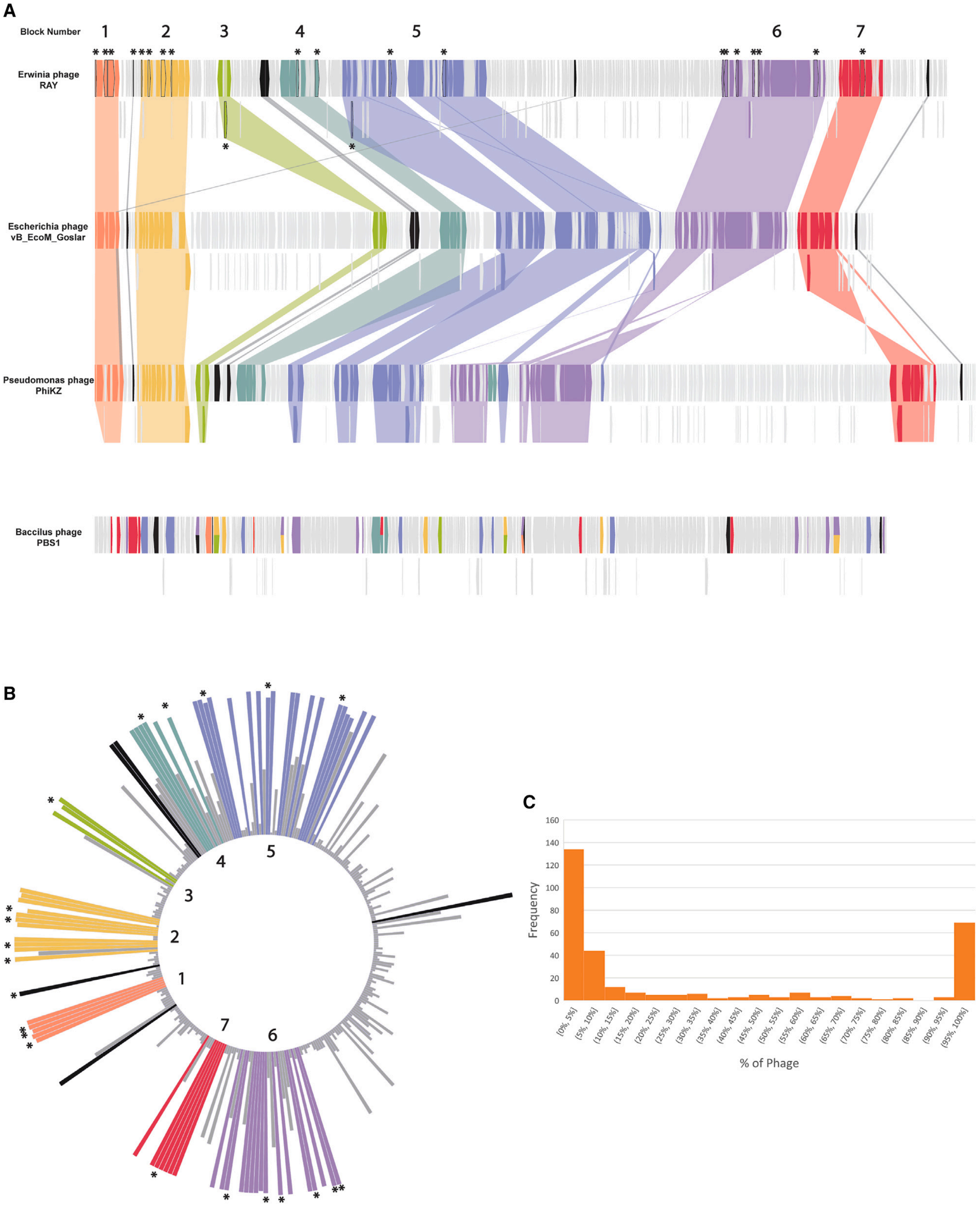

Figure 2. Core genome determination

(A) A genome alignment of *Erwinia* phage RAY, *Escherichia* phage Goslar, *Pseudomonas* phage ΦKZ, and *Bacillus* phage PBS1. PBS1 was included for comparison since it encodes the unique msRNAP but does not encode chimallin. The highly conserved regions with more than 3 genes are annotated in colors as

(legend continued on next page)

We next identified which of the core genes are specific to chimallin-encoding phages and likely represent the key genes required to replicate via the phage nucleus pathway versus core genes that are broadly conserved among phage in general. We found that 51 out of the 72 genes are also present in other phages lacking chimallin homologs (Table S1). These broadly conserved proteins include DNA polymerases, RNAP subunits, the major capsid protein, and other virion structural proteins. The remaining 21 genes include chimallin and 20 hypothetical proteins with no predicted structure or function. This unique set of 21 genes identified by our bioinformatic screen likely encodes the proteins specifically required to form and maintain a phage nucleus, but further study on each proposed core gene will be required to determine if it has a conserved role across different nucleus-forming phages. We propose that all phages encoding chimallin and the associated core genome belong to one viral family, Chimalliviridae, named after the shared protein that makes up the phage nucleus lattice.

Additionally, we searched metagenomic databases, and we were able to identify many more phages that encode chimallin and cluster within the Chimalliviridae. These phages were found in numerous environments from multiple locations across North America (Data S1). We also looked for phage metagenomes on NCBI, and when analyzing their whole-genome similarity, compared with known phages using VIPTree, we found that they also cluster within the Chimalliviridae clade (Figure S3). Taken together with the whole-phage genome sequence alignments, our results suggest that chimalliviruses are likely common and found throughout the world.

Phage nucleus formation by *Erwinia* phage RAY

Since RAY is a member of the Chimalliviridae and shares the core genome, we predicted that it replicates by forming a phage nucleus. We tested this prediction by imaging infected *Erwinia* cells and observed a bright zone of DAPI fluorescence that was often positioned at midcell, similar to *Pseudomonas* nucleus-forming phages (Figure 3A). We created an N-terminal fusion of GFPmut1 to gp222 (RAY chimallin homolog [ChmA$_{RAY}$]) (Figure S4A) and examined its localization in the absence and presence of phage infection. Fluorescence from GFP-ChmA$_{RAY}$ was uniformly distributed throughout uninfected cells but formed a ring in the center of the cell enclosing DNA during RAY infections (Figure 3B). Zones of DAPI fluorescence consistent with host chromosomal DNA were also present outside of the nucleus in every RAY infection, unlike previously characterized nucleus-forming phages, which degrade the host DNA,[7,8,10] but similar to *Serratia* phage PCH45.[11] To determine whether the extranuclear DAPI staining was due to host DNA, we tagged the *Erwinia amylovora* H-NS protein with GFP, which coats DNA, and used the H-NS-GFP fusion as a marker to visualize host DNA during phage infection, as H-NS is not imported into the phage nucleus. When we expressed H-NS-GFP in *Erwinia* cells and infected them with RAY, the H-NS-GFP fluorescence co-localized with the DAPI outside of the phage nucleus (Figure 3C). In time-lapse microscopy, the bacterial DNA was coated with H-NS before infection and was then pushed to the poles and compacted upon infection as the phage nucleus formed (Figure 3D; Video S1). These results suggest that RAY forms a nucleus-like structure without detectably degrading the host DNA.

Identification of RAY nuclear and cytoplasmic proteins

To gain further insight into the subcellular organization of RAY's lytic cycle, we used fluorescence microscopy to observe the localization of 24 RAY core and accessory proteins fused to GFP. To gain insights into both the conserved Chimalliviridae replication mechanism as well as RAY-specific variations, we tagged 15 proteins that were part of the Chimalliviridae core genome and 9 that were accessory components. We were able to tentatively assign potential protein families, to which most of these proteins belong based on *in silico* predictions; however, outside of the RNAP subunits, few of these phage proteins have been studied in detail, and their functions in the phage life cycle are unknown.

We identified 10 proteins that co-localize with the phage DNA, likely inside the phage nucleus (Figures 4A and 4B). Eight of these nucleus-associated RAY proteins (gp002, gp150, gp220, gp223, gp248, gp249, gp250, gp315) are part of the core genome, and two are not (gp116, gp153). Most of these proteins are likely involved in the core functions of DNA replication (gp220, gp315) (Figures 4B and S4C), transcription (gp002, gp248, gp223, gp249) (Figure S4D), or recombination (gp150, gp153) (Figures S4E and S4F) based on sequence and structural homology to previously characterized or annotated phage proteins. Two other proteins could be assigned to a protein family, but their functions are less clear (core gene gp250, SWI/SNF helicase family [Figure S4G] and accessory gene gp116, HslUV protease family [Figure S4H]).

We identified 4 proteins (gp039, gp048, gp064, and gp311) among our set of 24 that display diffuse localization in the cytoplasm outside of the phage nucleus and one protein (gp094) that localized on the host chromosome (Figure 4C). Of these, only RAY gp311, a thymidylate kinase homolog (Figure S4I), is part of the core genome. Homologs of gp311 have also been shown to localize in the cytoplasm during replication of other phages,[7,10] where they likely participate in nucleotide metabolism. The remaining 4 proteins included a putative SspB-like ClpXP adaptor protein (gp039; Figure S4J), a putative tRNA ligase (gp048; Figure S4K), a putative exonuclease (gp064; Figure S4L), and a putative XRE superfamily transcriptional regulator (gp094; Figure S4M) whose

different blocks. Core genes that are not part of blocks are colored black. While in chimalliviruses the core genes are conserved in seven blocks, in PBS1, some of these genes are present but are dispersed across its genome rather than being encoded in blocks. Goslar gp188 and ΦKZ gp055 are homologous to each other and to two RAY genes, gp223 (orange) and gp070 (black). The full list of genes and blocks can be found in Table S3.

(B) A circular bar plot of RAY's genome with heights of bars corresponding to the percentage of phage that contain genes homologous to each RAY gene. Certain regions are more variable (right of circle), while other regions are highly conserved.

Colors denote blocks and asterisks denote unique genes in (A) and (B).

(C) Histogram showing the frequency of conservation of each RAY protein among chimalliviruses. The left peak indicates RAY-specific proteins (conserved in less than 10% of chimalliviruses), and the right peak indicates core proteins (conserved in 90% or more chimalliviruses).

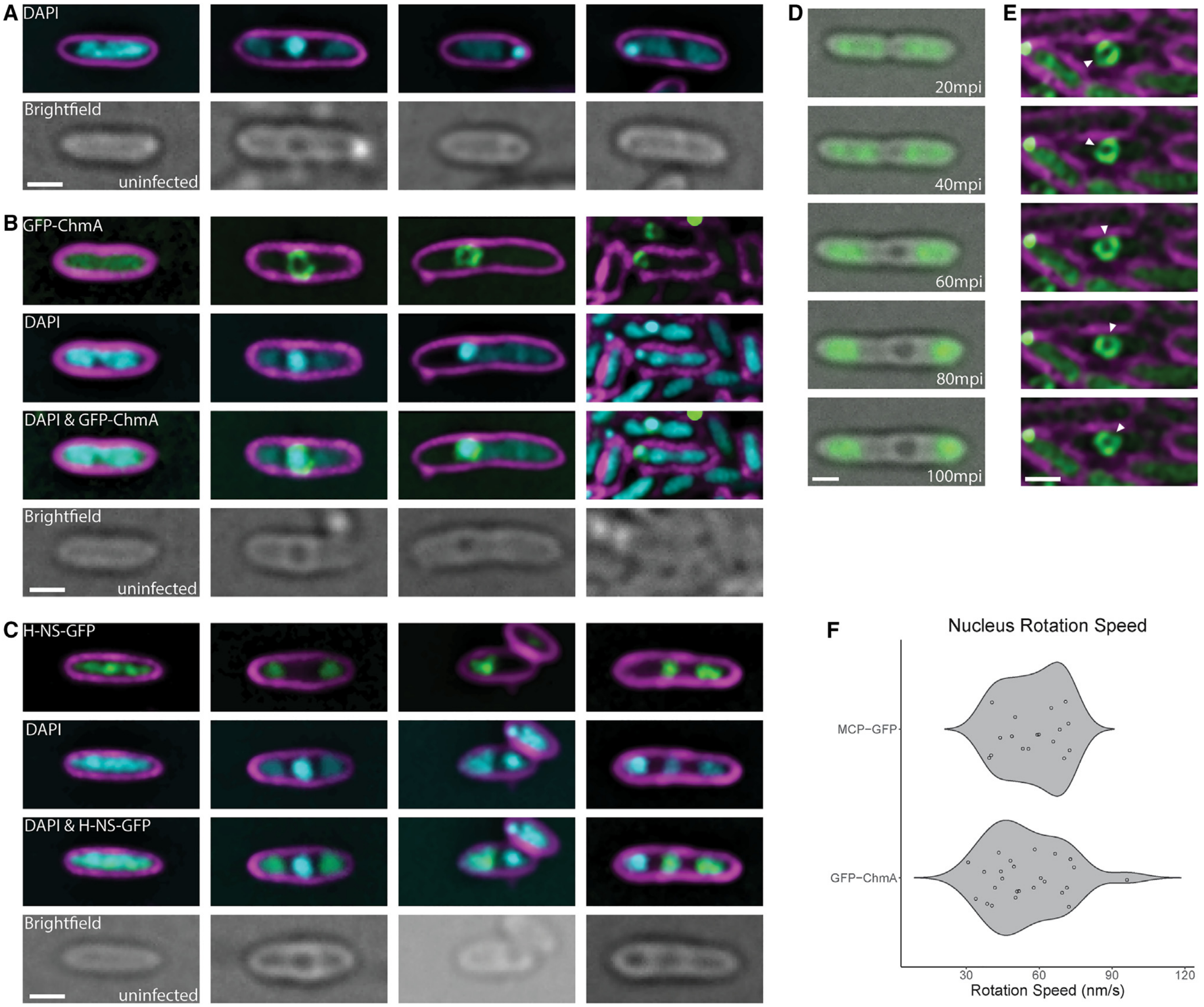

Figure 3. RAY phage nucleus formation

(A) DAPI localization showing the presence of a bright staining consistent with phage nucleus formation. Cells were imaged during midinfection between 60 and 75 minutes post infection (mpi) .

(B) GFP-ChmA$_{RAY}$ surrounds the bright DAPI zones, consistent with the morphology of a phage nucleus.

(C) H-NS-GFP localizes to the DNA outside of the phage nucleus.

(D) H-NS-GFP moves away from midcell during the course of infection (Video S1).

(E) The phage nucleus rotates during infection (Video S2). Time-lapse images were taken 4 s apart. The white arrow shows a segment of the nuclear shell that was tracked to determine rotation speed.

(F) Violin plot showing distribution of rotation speeds of individual measured RAY nuclei using either a chimallin (ChmA) (n = 25) or major capsid protein (MCP) (n = 17) GFP tag to track rotation. Scale bar is 1 μm; magenta is membrane stain FM4-64, cyan is DNA stain DAPI, green is GFP, and grayscale is brightfield. Cells were imaged between 60 and 75 mpi (midinfection) unless labeled otherwise.

localization mimics that of bacterial H-NS by binding to host DNA (Figures 3C and 4C). Taken together, these studies suggest that RAY forms a proteinaceous shell that encloses phage DNA and compartmentalizes proteins according to their functions.

Virion-associated RAY proteins

To follow the assembly process of RAY virions, we created GFP fusions to RAY's tail sheath (gp179; Figure S4N) and major capsid (gp317; Figure S4O) proteins. Fluorescence microscopy showed that the major capsid protein localized around the nucleus (Figure 4D), suggesting that capsids dock on the phage nucleus to initiate DNA packaging as occurs in ΦKZ-like phages and Goslar. The tail protein localizes in the cytoplasm, often forming foci near the phage nucleus (Figure 4D); however, we did not observe bouquet-like clusters of virions. This suggests that capsids dock on the phage nucleus to be packaged with DNA, but after assembling

CellPress
OPEN ACCESS

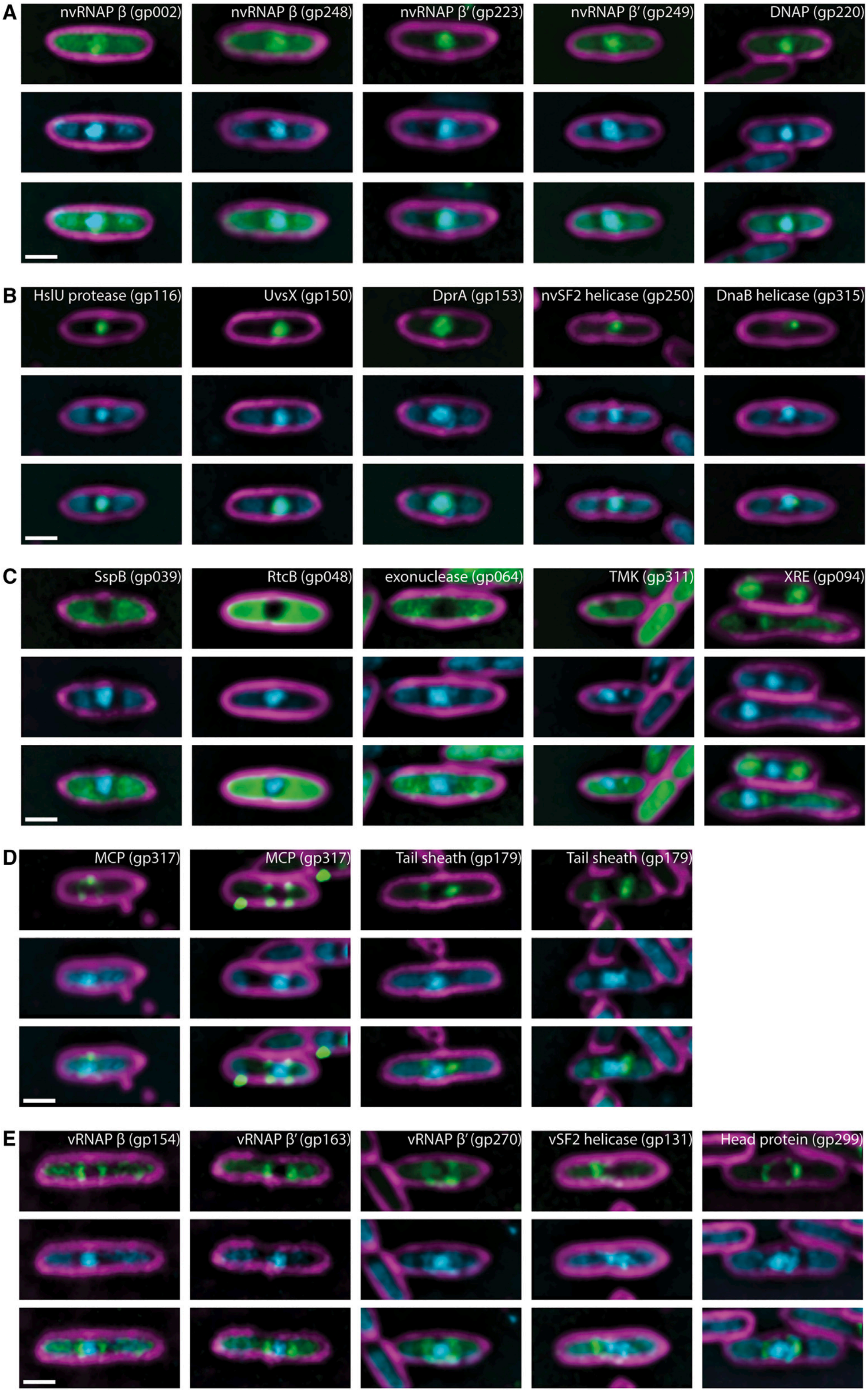

(legend on next page)

virions, bouquet-like structures are not formed like those observed in other nucleus-forming phages.[10,28]

We identified 5 proteins in our set of 24 that localized at the periphery of the phage nucleus during part of the lytic cycle, similar to the capsids (Figures 4D and 4E). Three of these (gp154, gp163, and gp270; Figure S4P) are vRNAP subunits. The other two are an SWI/SNF family helicase (gp131; Figure S4Q) and a putative head protein with unknown function (gp299; Figure S4R). Our localization of vRNAP and these other proteins during RAY infection is consistent with previous mass spectrometry results, which showed that RNAP subunits are packaged within the viral capsid.[16,29] These results also suggest that GFP fusions and fluorescence microscopy can be used as an additional approach to identify proteins that accumulate within capsids.

Phage tubulin PhuZ

RAY encodes a PhuZ homolog (PhuZ$_{RAY}$; gp210) (Figure S5A) that contains conserved domains of the tubulin superfamily, including the tubulin T4 signature motif (Figure 5A). In ΦKZ-like phages, PhuZ forms filaments that use dynamic instability and treadmilling to center and rotate the phage nucleus,[7,8,25–27] and in Goslar, PhuZ forms a vortex that rotates the phage nucleus without centering it.[10] To determine whether PhuZ$_{RAY}$ also forms filaments, we created a GFP-PhuZ$_{RAY}$ fusion and assessed its ability to form polymers at different expression levels in both infected and uninfected cells. In uninfected cells, at low levels of arabinose ($\leq$0.01%), GFP-PhuZ$_{RAY}$ is uniformly distributed throughout the cell, but it spontaneously forms filaments at $\geq$0.05% arabinose (Figures 5B and 5C). During infections, we studied GFP-PhuZ$_{RAY}$ during infections under conditions where it did not spontaneously assemble filaments (0% arabinose) and found visible filaments in all infected cells (Figures 5D and 5E), suggesting that the preexpressed GFP fusion in the host cell is able to co-assemble with phage-expressed native, untagged PhuZ.

To determine whether PhuZ centers the nucleus in RAY infections, we expressed a GTP-hydrolysis-deficient mutant, PhuZ$_{RAY}$D198A, as a dominant negative to inhibit the dynamic properties of the endogenous filaments and test whether the lack of filament dynamics interferes with nucleus positioning.[8,25,26] In uninfected and infected cells, GFP-PhuZ$_{RAY}$ D198A formed filaments at all arabinose concentrations, likely due to its inability to depolymerize (Figures 5B, 5C, and 5E). We measured the distance from the phage nucleus to the cell pole when GFP-PhuZ$_{RAY}$ and GFP-PhuZ$_{RAY}$D198A were expressed, taking into account whether the presence and location of bacterial nucleoids contributed to phage nucleus positioning. When a single bacterial nucleoid was present, the positioning of the RAY nucleus had a broad distribution with a slight bias toward midcell (Figure 5F, blue bars). The single nucleoid in these cells frequently occluded the center of the cell, thereby preventing the nucleus from being centered (Figure 5D, cell iii, white arrow). When two bacterial nucleoids were present, the RAY nucleus was usually (97%, n = 103) positioned between them near the cell midpoint (Figures 3A, 6D, cells i, ii, and iv, and 6G). When PhuZ$_{RAY}$D198A was expressed, the positioning of the RAY nucleus was less biased toward midcell when two nucleoids were present and had no specific localization when there was one nucleoid (Figures 5F and 5G). This is the first time that phage nucleus positioning was measured in the presence of the bacterial genome, and these results suggest that while the PhuZ filaments likely play a role in positioning the nucleus in phages that do not degrade host DNA, the phage nucleus must also compete for space with the bacterial DNA when it is not degraded.

In addition to centering the phage nucleus within the host cell, PhuZ also rotates the phage nucleus in both the ΦKZ-like phages and Goslar. We therefore performed time-lapse microscopy and measured nucleus rotation in RAY-infected cells expressing GFP-ChmA$_{RAY}$ (Figure 3E; Video S2). We observed very few of the phage nuclei actively rotating (3.7%, n = 858) compared with Goslar (97%)[10] and 201φ2-1 (46%).[27] Those that did rotated for 27.7 ± 5.8 s with an average linear rotation speed of 54.8 ± 15.6 nm/s and an angular velocity of 10.1° ± 3.2°/s (n = 25) (Figure 3F). We also measured nucleus rotations using GFP-tagged major capsid protein gp317 (MCP$_{RAY}$-GFP) docked on the surface of the nucleus as a fiduciary mark. The nuclei with docked capsids displayed a similar linear rotation speed of 57.5 ± 11.9 nm/s (n = 17). This rotation speed is only marginally faster when compared with Goslar (49.7 ± 12.5 nm/s) and 201φ2-1 (43.6 ± 7.6 nm/s) nucleus rotation speeds,[10,27] suggesting that this rotation rate is conserved, possibly to allow the even spacing of capsids docking on the phage nucleus surface for efficient DNA encapsidation.

Figure 4. Localization of proteins encoded by RAY

(A) Putative RNA (nvRNAP β subunit 1 gp002, nvRNAP β subunit 2 gp248, nvRNAP β′ subunit 1 gp223, and nvRNAP β′ subunit 2 gp249) and DNA (DNA polymerase gp220) polymerases co-localize with phage DNA in the phage nucleus.

(B) RAY gp116 is a homolog of HslUV superfamily heat shock proteases, but other nuclear-localized phage proteins are homologs of known DNA-associated proteins such as proteins involved in recombination (UvsX gp150 and DprA gp153) and helicases (non-virion superfamily 2 helicase gp250 and DnaB-like replicative helicase gp315).

(C) Cytoplasmic proteins are not predicted to be involved with DNA replication, recombination, or transcription. These include an SspB homolog gp039, RtcB homolog gp048, putative exonuclease gp064, thymidylate kinase gp311, and putative XRE superfamily transcriptional regulator gp094.

(D) The MCP gp317 and tail sheath gp179 are structural components of the RAY virion that localize near the periphery of the phage nucleus. Puncta can be seen near the membrane in the MCP fusion, possibly because capsids are assembled there or, alternatively, as part of an aggregate formed due to overexpression of gp317-GFP.

(E) Virion proteins (vRNAP β subunit 2 gp154, vRNAP β′ subunit 1 gp163, vRNAP β′ subunit 2 gp270, virion-associated superfamily 2 helicase gp131, and head protein of unknown function gp299) can be seen with similar localizations as the MCP and sometimes (in the case of gp270 and gp299) have visible DAPI co-localized with them.

For all images, scale bar is 1 μm; magenta is membrane stain FM4-64, cyan is DNA stain DAPI, and green is GFP.

CellPress
OPEN ACCESS

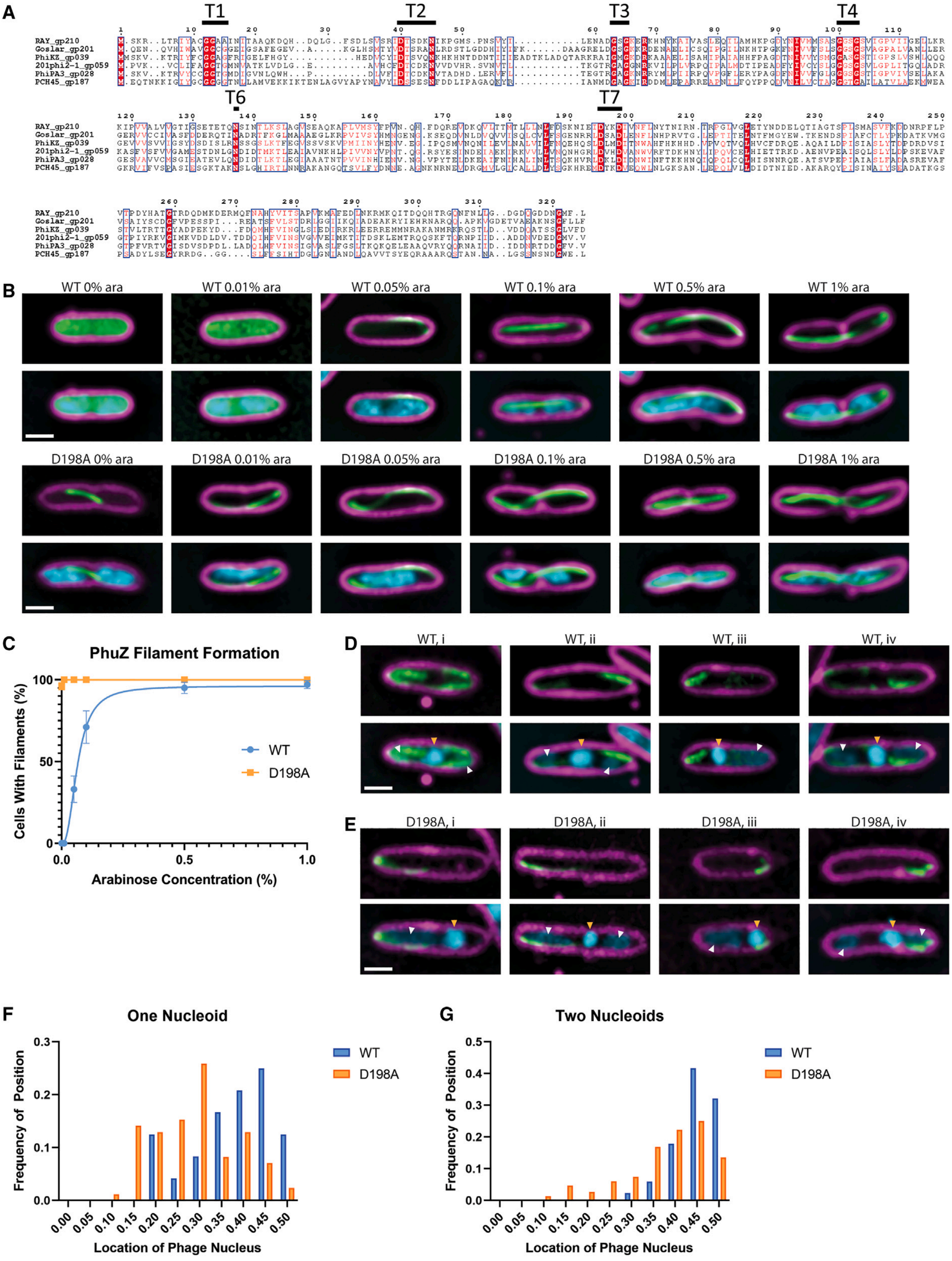

(legend on next page)

In situ structural analysis of RAY phage replication in *Erwinia amylovora*

In order to investigate the structural conservation of chimallivirus replication components, we performed cryo-focused ion beam milling and electron tomography (cryo-FIB-ET) of *Erwinia amylovora* cells infected with RAY (Figure 6). The tomograms revealed the presence of the RAY phage nuclear shell as an irregularly shaped, protein-based compartment enclosing nucleic acid and excluding host ribosomes (Figures 6A and 6B). Viral capsids were observed at different stages of maturation, docked on the phage nucleus where they package DNA, or in the cytoplasm with or without tails attached. We did not observe virion particles assembled into structured bouquets, in agreement with fluorescence microscopy (Figures 6A and 6B).

To further assess the components of RAY replication, we performed subtomogram analysis of virion components, the phage nucleus shell, and PhuZ filaments. Through averaging of assembled and isolated virion components, we obtained reconstructions for the RAY capsid (~19 Å), collar (~38 Å), tail sheath (~10 Å), and baseplate (~36 Å). Dimensions of assembled virions measured from the tomograms and overlapping segments of the component maps allowed reconstruction of a composite RAY virion (Figure 6C). The virion is largely similar to the composite structure of the isolated ΦKZ virion.[44] The RAY capsid exhibits a triangulation number of 27 (T = 27) and is approximately 142.5 nm in diameter, which is similar to the ΦKZ capsid. However, the RAY baseplate reconstruction appears in a retracted state, as opposed to the wider expanded state observed for the ΦKZ virion.[44]

Subtomogram analysis of the phage nuclear shell resulted in a ~20 Å reconstruction which revealed that the RAY nuclear shell is principally composed of a square chimallin lattice with ~11.5 nm repeat distance, similar to that assembled by the 201φ2-1 and Goslar chimallin (Figure 6D).[9] The conserved higher-order structure of the RAY chimallin lattice indicates a conserved underlying protomer structure. Indeed, the AlphaFold 2.0-predicted structure of RAY chimallin is largely consistent with the experimental chimallin structures determined for 201φ2-1 (root-mean-square deviation [RMSD]: 2.2 Å) and Goslar (RMSD: 2.2 Å).[9] The $ChmA_{RAY}$ protomer prediction predominantly differs from experimental structures in the low-confidence placement of the extended N- and C-terminal segments, which mediate interactions across ChmA protomers in higher-order assemblies (Figure S6).

Finally, we performed subtomogram analysis (StA) of long, hollow, filamentous structures present in RAY-infected *Erwinia* cells assumed to be the phage-encoded tubulin PhuZ (Figures 6A, 6B, and 6E). The resulting StA map achieved an estimated resolution of ~25 Å and revealed a hollow, five-stranded filament of approximately 13 nm in diameter (Figure 6F), which is in stark contrast to the three-stranded filaments made by $PhuZ_{201φ2-1}$ obtained *in vitro*.[23,25] We did not observe other filamentous structures in our tomogram set, thus the five-stranded structures are tentatively assigned as RAY PhuZ. StA of PhuZ filaments observed in our previously published 201φ2-1 and ΦKZ cryo-FIB-ET datasets revealed that these PhuZ variants form three-stranded filaments *in situ* (Figures S5B–S5D). The structure of the $PhuZ_{RAY}$ protomer as predicted by AlphaFold is highly similar (RMSD = 1.069 Å) to the experimentally determined crystal structure of $PhuZ_{201φ2-1}$ (Figure 5H),[23,25] thus indicating that subtle differences are likely responsible for differences in higher-order assembly among PhuZ variants. Of note, residues R217:D305 and E225:R290 form essential salt bridges for self-assembly of $PhuZ_{201φ2-1}$ and are conserved in $PhuZ_{ΦKZ}$ (R230:D316 and D238:R301) but not in $PhuZ_{RAY}$ (D223:D315 and L235:D299). Further work will be necessary to unambiguously determine whether the five-stranded filaments are indeed $PhuZ_{RAY}$ and the molecular bases of their higher-order assembly.

Taken together, the structures seen in the cryo-FIB-ET corroborate the fluorescence microscopy results and confirm that RAY is a nucleus-forming phage that has unique and intriguing differences when compared with previously published Chimalliviridae Goslar, PCH45, and ΦKZ-like phages.

DISCUSSION

The phage nucleus is a remarkable and provocative cell biological structure, but the viral genes required for phage to replicate via this pathway are unknown. Here, we bioinformatically identified the core genome that defines the nucleus-forming Chimalliviridae family. The Chimalliviridae core genome consists of a set of 72 genes encoded within seven distinct blocks. The order of the core blocks and genes within the blocks was generally conserved among divergent members of the family, as was their phylogeny. This suggests that these phages evolved from a common ancestor and that their genes have been co-evolving without significant horizontal gene transfer. We propose that

Figure 5. RAY PhuZ homolog

(A) $PhuZ_{RAY}$ has conserved tubulin motifs. T5 is a structural motif with a poorly conserved sequence and is not included.

(B) Wild-type $PhuZ_{RAY}$ does not polymerize spontaneously until it reaches a critical concentration, but the D198A mutant polymerizes at all levels of arabinose induction tested.

(C) The average percentage of uninfected cells with PhuZ filaments plotted versus arabinose concentration. Data are represented as mean ± SEM.

(D) Wild-type PhuZ filaments form with 0% arabinose during RAY infections. The nucleus is normally positioned near midcell (white arrows show bacterial DNA, and gold arrows show phage nuclear DNA).

(E) When the D198A mutant PhuZ is expressed, midcell localization of the phage nucleus during infection is not as common as with wild-type PhuZ (white arrows show bacterial DNA, and gold arrows show phage nuclear DNA).

(F) When only one bacterial nucleoid is present, the phage nucleus positioning histogram has a wide distribution for wild-type PhuZ (blue), and the D198A mutant PhuZ (orange) does not appear to strongly affect positioning.

(G) When two bacterial nucleoids are present, the wild-type PhuZ has a strong nucleus positioning bias toward midcell (blue), and the D198A mutant PhuZ has a weaker positioning bias toward midcell (orange).

For all microscopy images (B, D, and E), scale bar is 1 μm; magenta is membrane stain FM4-64, cyan is DNA stain DAPI, and green is GFP.

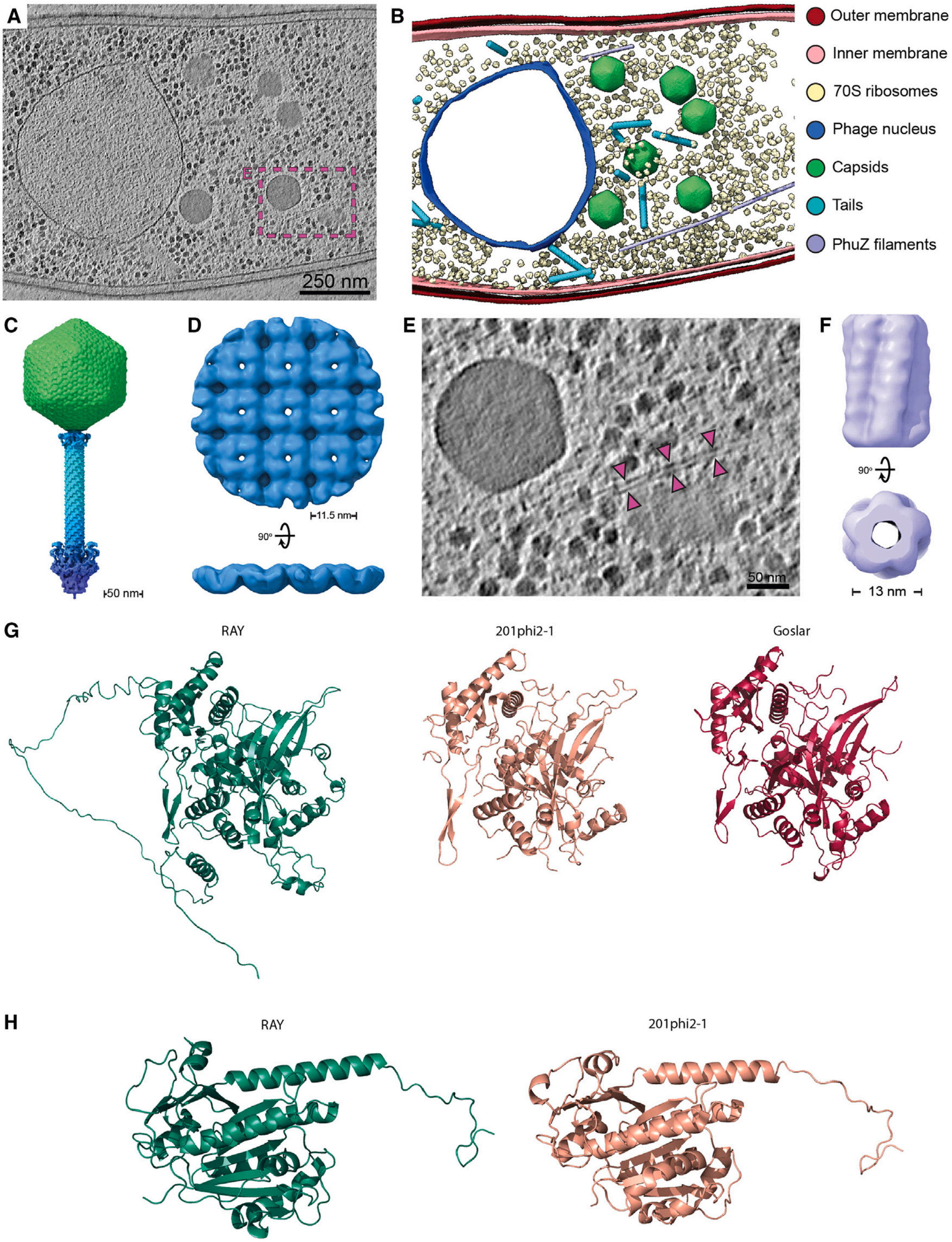

Figure 6. Cryoelectron tomography and structural analysis

(A) Slice through a cryoelectron tomogram of a RAY-infected *Erwinia amylovora* cell at approximately 105–110 mpi. Scale bar is 250 nm.

(B) Segmentation of the tomogram in (A).

(C) Composite RAY virion reconstruction.

(D) Orthogonal views of the RAY chimallin lattice reconstruction.

(E) Enlarged view of region boxed in (A) with magenta arrows pointing to a putative RAY PhuZ filament. Scale bar is 50 nm.

(F) Reconstruction of the putative RAY PhuZ filaments showing the five-stranded structure and hollow lumen.

(legend continued on next page)

the core genome identified here includes many of the key genes required for nucleus-based phage replication and that the less conserved accessory genes may be important for different subfamilies of phage or allow specialization for infecting different hosts. The core genes encode proteins that participate in many key processes such as genome replication, gene expression, and formation of virion particles. However, 74% of the core genes (53/72) are hypothetical proteins of unknown function and origin; moreover, 29% (21/72) make up the core genes that are unique to the Chimalliviridae, and of these, only chimallin has a known function.[7,9] This analysis agrees well with previous studies that identified a core gene set shared by different groups of jumbo phages.[38,39] This core gene set likely encodes numerous unknown components required for phage nucleus formation, standing in stark contrast to the core genome of the phage T4 family (Tequatroviridae), where the function of the majority of the core genes are known and genes of unknown function are primarily accessory.[35] Thus, the Chimalliviridae core genome is rich with potential for discovery of novel biological functions due to its numerous uncharacterized genes.

Phage can be classified into families based on genetic data.[45] Our phylogenetic analysis suggests that all phages encoding this core genome form a single family, the Chimalliviridae, that only arose once and developed a unique nucleus-based replication mechanism. Within this family are clades of phages with specific accessory genomes, suggesting that different Chimalliviridae clades have adapted the phage nucleus replication mechanism in a variety of diverse ways. The core genome of nucleus-forming phages described here can be used to study the basic requirements for nucleus-based replication, discover new members of the Chimalliviridae, and guide future studies in synthetic biology aimed at building a phage encoding only the minimal components required for phage nucleus-based replication.

Chimalliviruses infect a broad range of bacteria and have been found in many locations throughout the world. By searching metagenomic databases, we identified many more Chimalliviridae members from a variety of sources (rhizosphere, brine, fracking water, peat, uranium contaminated flood plain, bog, agave, subway wood, and deep shale) and many distant locations (Michigan, Oregon, Alaska, Oklahoma, Iowa, New York, Ohio, Alaska, Nebraska, and Wyoming, USA, and Guanajuato, Mexico) (Data S1). Indeed, phages closely related to ΦKZ have been isolated from South Korea, Germany, Poland, Denmark, the USA, Australia, Japan, Thailand, Iran, India, and Kazakhstan.[46–52] We also found that phage metagenomes on NCBI cluster within the clade of chimalliviruses as part of this same family (Figure S3). We conclude that Chimalliviridae form a single clade of phages that are widespread and found in habitats throughout the world.

We performed a detailed investigation into RAY to determine how conserved the phage nucleus replication mechanism is in a divergent Chimalliviridae family member. By examining the intracellular localization of 15 core and 9 non-core RAY proteins via GFP tagging and fluorescence microscopy, we discovered that these proteins have conserved subcellular localizations across Chimalliviridae, with DNA processing proteins being found inside the phage nucleus, virion proteins being found around the periphery of the phage nucleus, and other proteins being found in the cytoplasm (Figures 3, 4, 5, and 6).[7,8,10,11,13,27] We show that chimallin surrounds the phage DNA, and cryo-FIB-ET revealed it assembles an enclosed square lattice, forming a 6-nm-thick shell that separates phage DNA from ribosomes and virion structural proteins in the cytoplasm, similar to 201φ2-1, ΦKZ, ΦPA3, and Goslar.[7,8,10,13,27] The putative functions of these proteins have been updated in the NCBI annotation (NC_041973.1).

We also found several additional surprises (unconserved processes) that add to our knowledge of the diversity of chimallivirus replication. First, the host chromosome is not degraded and is excluded from the phage nucleus. This has also previously been observed with phage PCH45[11] and suggests that the host chromosome is not a barrier to building a phage nucleus. Second, the PhuZ spindle and host chromosomes both likely impact phage nucleus position. Third, unlike previously studied PhuZ proteins from 201φ2-1, ΦKZ, and ΦPA3, which form three-stranded filaments,[23] $PhuZ_{RAY}$ appears to form five-stranded filaments *in vivo*, which make $PhuZ_{RAY}$ the first phage-encoded tubulin-based structure that possesses a lumen (Figures 6F and S5). This is not only distinct from other PhuZ homologs but also from most bacterial tubulins, which assemble either single filaments (FtsZ)[53–55] or two- or four-stranded filaments (TubZ).[56–58] However, it is similar to *Prosthecobacter* tubulins BtubA/BtubB, which assemble five-stranded tubules *in vivo*, and a variety of single, double, and polymeric structures *in vitro* and are more similar to eukaryotic tubulins than bacterial tubulins.[59–61] Future studies will be needed to confirm that PhuZ has these assembly properties *in vitro*. Fourth, unlike *Pseudomonas* and *Escherichia coli* nucleus-forming phages, whose nuclei are more consistently rotated by the PhuZ spindle, we rarely observed nucleus rotation with RAY. Finally, unlike *Pseudomonas* and *Escherichia coli* phages,[10,28] RAY viral particles do not form bouquets.

We described the characterization of ten proteins that had not previously been studied in any chimallivirus. For example, we discovered one core and one accessory protein that both contain a domain belonging to the SWI/SNF helicase family, but their functions are unknown. Surprisingly, we found one of these proteins (gp250) localized inside the nucleus, while the other one (gp131) localized inside the capsids. Another example is gp094, an accessory protein predicted to be an XRE family transcriptional repressor. gp094 binds to bacterial DNA, prompting us to speculate that it might regulate host gene expression. Other proteins described here include a HslUV-like protease (gp116), a ClpXP protease adapter protein (gp039), and a tRNA ligase (gp048), which could potentially be involved in phage replication or overcoming host defense systems.

(G) AlphaFold v.2.1.0 structure of RAY chimallin compared with experimentally determined 201φ2-1 and Goslar chimallin.[9]
(H) AlphaFold v.2.1.0 structure of RAY PhuZ compared with experimentally determined 201φ2-1 PhuZ.[23,25]
Accession numbers: (C) EMD-28003 (additional map), (D) EMD-28007, and (F) EMD-28008. See Figure S7 for subtomogram analysis workflows of RAY components.

CellPress
OPEN ACCESS

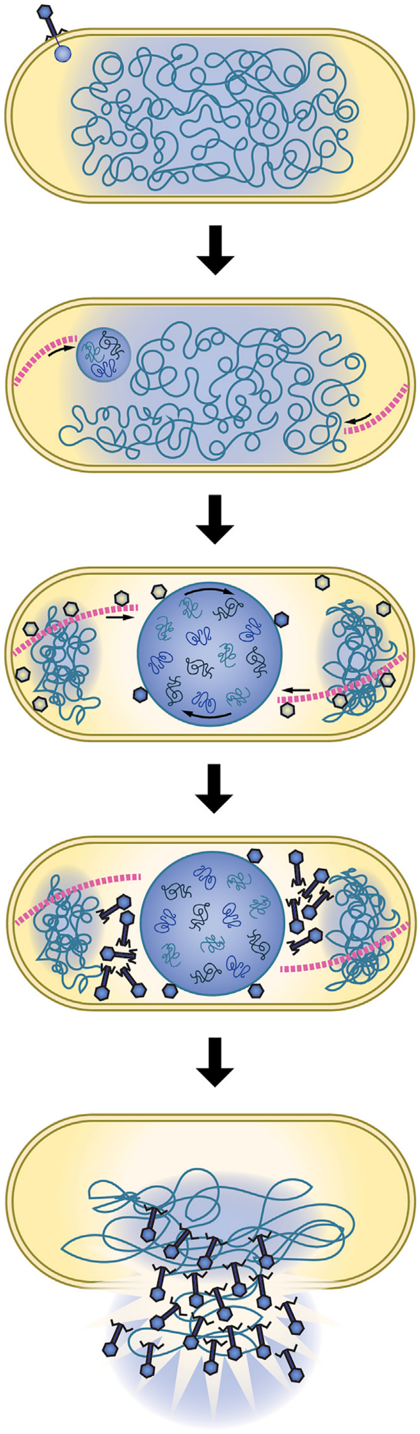

Figure 7. RAY infection model
See text for details.

Our results suggest a model for RAY replication that is distinct from other previously characterized nucleus-forming phages (Figure 7). Upon infection, RAY injects its DNA and a suite of proteins for viral replication, including four vRNAP proteins, as well as proteins of unknown function (such as putative SF2 helicase gp131 and internal head protein gp299). The injected RNAP expresses chimallin,[17] which forms a proteinaceous shell surrounding the phage DNA (Figure 3B). Unlike with 201φ2-1, ΦKZ, ΦPA3, and Goslar, the host chromosome is not degraded, similar to PCH45 infection,[11] and the cell does not undergo substantial swelling. The growing phage nucleus therefore must compete for limited space with the chromosomal DNA. If the cell contains two well-separated nucleoids, the nucleus usually localizes between them (Figures 3B, 6D, and 6E; Video S1). Capsids dock on the phage nucleus (Figures 4D, 4E, 7A, and 7B), package DNA, and assemble mature viral particles (Figure 7). $PhuZ_{RAY}$ forms filaments that appear to contribute to RAY nucleus positioning (Figures 5F and 5G), but whether they contribute to capsid trafficking as speculated in Figure 7 is unknown. It is also unclear if they contribute to nuclear rotation: since the RAY nucleus was only observed to rotate in ~3.7% of infected cells, it was not possible to measure a significant difference between expressing live versus catalytically dead PhuZ. After assembly of mature phage particles, RAY does not form bouquets (Figures 6A and 6B). Given RAY's inability to degrade the chromosome or cause the cell to bulge, there may be no room for such structures to assemble.

The phage-encoded tubulin homolog PhuZ is absent from one-third of known Chimalliviridae yet displays conserved function in phage nucleus rotation in 201φ2-1, ΦKZ, ΦPA3, and Goslar[10,27] and in nuclear positioning in 201φ2-1, ΦKZ, ΦPA3, and RAY[7,8,25,26] (Figures 5F and 5G). PhuZ is an example of an accessory protein that has likely adapted to make different types of cytoskeletal structures for different phages. Among the subset of Chimalliviridae we have examined thus far, we have observed PhuZ to form a bipolar spindle or a vortex and either three- or five-stranded tubes reminiscent of Verrucomicrobial BtubA/BtubB.[59–61] Additionally, prior studies have reported only a 50% decrease in phage titers when expressing dominant negative PhuZ mutants despite severe phage nucleus positioning defects.[8,25,26] In agreement with these studies, the PhuZ gene can be completely deleted from ΦKZ, and while phage nucleus position is again strongly affected, the phage is still viable.[43] Taken together, these studies suggest that PhuZ is an accessory protein that may confer improved infection efficiency in some nucleus-forming phages, but it is not essential for this viral replication pathway.

Many phages package proteins within their capsids that they are thought to inject into the cell along with their DNA.[14,16,62–64] These proteins have previously been discovered by performing mass spectrometry on purified viral particles.[16] Using GFP fusions, we have shown localizations consistent with virion packaging of these proteins, corroborating previous mass spectroscopy data and showing that visualization of internal capsid proteins is possible using fluorescence microscopy, as found in another a recent study.[43] For example, the vRNAP subunits localized around the phage nucleus where the capsids accumulated, in agreement with the previous mass spectrometry data that these vRNAPs are encapsidated.[16]

This work highlights how, despite the unique features of each nucleus-forming phage studied to date, many of the basic components are conserved across the entire diverse family of Chimilliviridae. Here, we document differences in phage tubulin assembly, nucleus positioning, nucleus rotation, chromosome

degradation, and cell bulging that likely represent just a few of the many phenotypic consequences that result from continual phage-host evolutionary conflicts. Many of these differences are likely due to the continually evolving accessory genome. Now that the core and accessory genomes of this unique phage family have been identified, future studies can be better directed toward understanding their functions in order to elucidate the intricacies of the nucleus-based phage replication mechanism.

Limitations of the study

The core genome determination is based on genes identified using BLAST. Proteins with similar structures and common ancestry can be so highly divergent as to appear unrelated solely based on sequence identity. To counter this, we performed four iterations of PSI-BLAST. Another limitation is the arbitrary 90% conservation cutoff we used to define the core genome. We used this number to avoid excluding some genes from the core genome due to sequencing errors. Thus, the list of core proteins identified here is based on currently available phage sequence space but does not represent all genes important for nucleus-based replication. Finally, GFP tagging can alter protein localization. To address this possibility, we tagged proteins at both the N and C termini.

STAR★METHODS

Detailed methods are provided in the online version of this paper and include the following:

- KEY RESOURCES TABLE
- RESOURCE AVAILABILITY
  - Lead contact
  - Materials availability
  - Data and code availability
- EXPERIMENTAL MODEL AND SUBJECT DETAILS
  - Bacterial growth and phage infection conditions
  - Expression of phage proteins
- METHOD DETAILS
  - Genome alignments and phylogenetic analysis
  - Core genome determination
  - Phage protein characterization
  - Fluorescence microscopy
  - Cryo-electron microscopy of RAY-infected cells
  - Tomogram segmentation
  - Structural predictions
- QUANTIFICATION AND STATISTICAL ANALYSIS
  - Nucleus rotation analysis
  - PhuZ filamentation and nucleus positioning analysis
  - Subtomogram analysis

SUPPLEMENTAL INFORMATION

Supplemental information can be found online at https://doi.org/10.1016/j.celrep.2023.112432.

ACKNOWLEDGMENTS

We thank B. Dennis and K. Smith of UCSD Physics Computing for computational support. Electron microscopy data were collected at the UCSD Cryo-Electron Microscopy Facility, which was built and equipped with funds from the UCSD and an initial gift from the Agouron Institute. The authors acknowledge funding from the National Institutes of Health grant R01 GM129245 (to J.P. and E.V.), the National Science Foundation grant DBI 1920374 (to E.V.), and the Howard Hughes Medical Institute Emerging Pathogens Initiative grant (to E.V., J.P., K.P., J.M., and K.D.C.). T.G.L. is a Simons Foundation Awardee of the Life Sciences Research Foundation. E.V. is a Howard Hughes Medical Institute Investigator.

AUTHOR CONTRIBUTIONS

Conceptualization, A.P., T.S., J.P., and R.D.; data curation, A.P., J.L., and T.G.L.; formal analysis, A.P., J.L., T.G.L., and A.L.; funding acquisition, J.P. and E.V.; investigation, A.P., J.L., T.G.L., A.L., K.P.T., and A.E.S.; methodology, A.P., J.L., and R.D.; project administration, J.P. and R.D.; resources, J.H.G., E.V., J.P., and R.D.; software, J.L.; supervision, J.P. and R.D.; validation, A.P. and J.L.; visualization, A.P., J.L., T.G.L., A.L., and K.P.T.; writing – original draft, A.P., J.L., T.G.L., A.L., K.P.T., A.E.S., T.S., A.A., A.C., M.C., N.C., D.D., I.D., G.D., M.G., K.H., L.H., E.J., A.J., S.K., R.K., A.M., M.N., T.P., G.P., K.S., M.S., A.J.S., S.S., A.S., A.U., A.Z., R.D., and J.P.; writing – review & editing, A.P., J.L., T.G.L., K.D.C., K.P., J.M., J.H.G., E.V., R.D., and J.P.

DECLARATION OF INTERESTS

K.P. and J.P. have an equity interest in Linnaeus Bioscience Incorporated and receive income. The terms of this arrangement have been reviewed and approved by the University of California, San Diego, in accordance with its conflict-of-interest policies.

INCLUSION AND DIVERSITY

We support inclusive, diverse, and equitable conduct of research.

Received: December 14, 2022
Revised: February 28, 2023
Accepted: April 8, 2023
Published: April 28, 2023

REFERENCES

1. Paszkowski, P., Noyce, R.S., and Evans, D.H. (2016). Live-cell imaging of vaccinia virus recombination. PLoS Pathog. *12*, e1005824.
2. Tomer, E., Cohen, E.M., Drayman, N., Afriat, A., Weitzman, M.D., Zaritsky, A., and Kobiler, O. (2019). Coalescing replication compartments provide the opportunity for recombination between coinfecting herpesviruses. FASEB J *33*, 9388–9403.
3. Kieser, Q., Noyce, R.S., Shenouda, M., Lin, Y.-C.J., and Evans, D.H. (2020). Cytoplasmic factories, virus assembly, and DNA replication kinetics collectively constrain the formation of poxvirus recombinants. PLoS One *15*, e0228028.
4. Trinh, J.T., Shao, Q., Guan, J., and Zeng, L. (2020). Emerging heterogeneous compartments by viruses in single bacterial cells. Nat. Commun. *11*, 3813.
5. Labarde, A., Jakutyte, L., Billaudeau, C., Fauler, B., López-Sanz, M., Ponien, P., Jacquet, E., Mielke, T., Ayora, S., Carballido-López, R., and Tavares, P. (2021). Temporal compartmentalization of viral infection in bacterial cells. Proc. Natl. Acad. Sci. USA *118*, e2018297118. https://doi.org/10.1073/pnas.2018297118.
6. Knipe, D.M., Prichard, A., Sharma, S., and Pogliano, J. (2022). Replication compartments of eukaryotic and bacterial DNA viruses: common themes between different domains of host cells. Annu. Rev. Virol. *9*, 307–327.
7. Chaikeeratisak, V., Nguyen, K., Khanna, K., Brilot, A.F., Erb, M.L., Coker, J.K.C., Vavilina, A., Newton, G.L., Buschauer, R., Pogliano, K., et al. (2017). Assembly of a nucleus-like structure during viral replication in bacteria. Science *355*, 194–197.

8. Chaikeeratisak, V., Nguyen, K., Egan, M.E., Erb, M.L., Vavilina, A., and Pogliano, J. (2017). The phage nucleus and tubulin spindle are conserved among large Pseudomonas phages. Cell Rep. *20*, 1563–1571.

9. Laughlin, T.G., Deep, A., Prichard, A.M., Seitz, C., Gu, Y., Enustun, E., Suslov, S., Khanna, K., Birkholz, E.A., Armbruster, E., et al. (2022). Architecture and self-assembly of the jumbo bacteriophage nuclear shell. Nature *608*, 429–435.

10. Birkholz, E.A., Laughlin, T.G., Armbruster, E., Suslov, S., Lee, J., Wittmann, J., Corbett, K.D., Villa, E., and Pogliano, J. (2022). A cytoskeletal vortex drives phage nucleus rotation during jumbo phage replication in E. coli. Cell Rep. *40*, 111179.

11. Malone, L.M., Warring, S.L., Jackson, S.A., Warnecke, C., Gardner, P.P., Gumy, L.F., and Fineran, P.C. (2020). A jumbo phage that forms a nucleus-like structure evades CRISPR-Cas DNA targeting but is vulnerable to type III RNA-based immunity. Nat. Microbiol. *5*, 48–55.

12. Mendoza, S.D., Nieweglowska, E.S., Govindarajan, S., Leon, L.M., Berry, J.D., Tiwari, A., Chaikeeratisak, V., Pogliano, J., Agard, D.A., and Bondy-Denomy, J. (2020). A bacteriophage nucleus-like compartment shields DNA from CRISPR nucleases. Nature *577*, 244–248.

13. Nguyen, K.T., Sugie, J., Khanna, K., Egan, M.E., Birkholz, E.A., Lee, J., Beierschmitt, C., Villa, E., and Pogliano, J. (2021). Selective transport of fluorescent proteins into the phage nucleus. PLoS One *16*, e0251429.

14. Sokolova, M.L., Misovetc, I., and Severinov, K.,V. (2020). Multisubunit RNA polymerases of jumbo bacteriophages. Viruses *12*, 1064. https://doi.org/10.3390/v12101064.

15. Thomas, J.A., Rolando, M.R., Carroll, C.A., Shen, P.S., Belnap, D.M., Weintraub, S.T., Serwer, P., and Hardies, S.C. (2008). Characterization of Pseudomonas chlororaphis myovirus 201varphi2-1 via genomic sequencing, mass spectrometry, and electron microscopy. Virology *376*, 330–338.

16. Thomas, J.A., Weintraub, S.T., Wu, W., Winkler, D.C., Cheng, N., Steven, A.C., and Black, L.W. (2012). Extensive proteolysis of head and inner body proteins by a morphogenetic protease in the giant Pseudomonas aeruginosa phage φKZ. Mol. Microbiol. *84*, 324–339.

17. Ceyssens, P.-J., Minakhin, L., Van den Bossche, A., Yakunina, M., Klimuk, E., Blasdel, B., De Smet, J., Noben, J.-P., Bläsi, U., Severinov, K., and Lavigne, R. (2014). Development of giant bacteriophage φKZ is independent of the host transcription apparatus. J. Virol. *88*, 10501–10510.

18. Matsui, T., Yoshikawa, G., Mihara, T., Chatchawankanphanich, O., Kawasaki, T., Nakano, M., Fujie, M., Ogata, H., and Yamada, T. (2017). Replications of two closely related groups of jumbo phages show different level of dependence on host-encoded RNA polymerase. Front. Microbiol. *8*, 1010.

19. Yakunina, M., Artamonova, T., Borukhov, S., Makarova, K.S., Severinov, K., and Minakhin, L. (2015). A non-canonical multisubunit RNA polymerase encoded by a giant bacteriophage. Nucleic Acids Res. *43*, 10411–10420.

20. Orekhova, M., Koreshova, A., Artamonova, T., Khodorkovskii, M., and Yakunina, M. (2019). The study of the phiKZ phage non-canonical non-virion RNA polymerase. Biochem. Biophys. Res. Commun. *511*, 759–764.

21. Krylov, V., Bourkaltseva, M., Pleteneva, E., Shaburova, O., Krylov, S., Karaulov, A., Zhavoronok, S., Svitich, O., and Zverev, V. (2021). Phage phiKZ-the first of giants. Viruses *13*, 149. https://doi.org/10.3390/v13020149.

22. de Martín Garrido, N., Orekhova, M., Lai Wan Loong, Y.T.E., Litvinova, A., Ramlaul, K., Artamonova, T., Melnikov, A.S., Serdobintsev, P., Aylett, C.H.S., and Yakunina, M. (2021). Structure of the bacteriophage PhiKZ non-virion RNA polymerase. Nucleic Acids Res. *49*, 7732–7739.

23. Zehr, E.A., Kraemer, J.A., Erb, M.L., Coker, J.K.C., Montabana, E.A., Pogliano, J., and Agard, D.A. (2014). The structure and assembly mechanism of a novel three-stranded tubulin filament that centers phage DNA. Structure *22*, 539–548.

24. Aylett, C.H.S., Izoré, T., Amos, L.A., and Löwe, J. (2013). Structure of the tubulin/FtsZ-like protein TubZ from Pseudomonas bacteriophage ΦKZ. J. Mol. Biol. *425*, 2164–2173.

25. Kraemer, J.A., Erb, M.L., Waddling, C.A., Montabana, E.A., Zehr, E.A., Wang, H., Nguyen, K., Pham, D.S.L., Agard, D.A., and Pogliano, J. (2012). A phage tubulin assembles dynamic filaments by an atypical mechanism to center viral DNA within the host cell. Cell *149*, 1488–1499.

26. Erb, M.L., Kraemer, J.A., Coker, J.K.C., Chaikeeratisak, V., Nonejuie, P., Agard, D.A., and Pogliano, J. (2014). A bacteriophage tubulin harnesses dynamic instability to center DNA in infected cells. Elife *3*, e03197. https://doi.org/10.7554/eLife.03197.

27. Chaikeeratisak, V., Khanna, K., Nguyen, K.T., Sugie, J., Egan, M.E., Erb, M.L., Vavilina, A., Nonejuie, P., Nieweglowska, E., Pogliano, K., et al. (2019). Viral capsid trafficking along treadmilling tubulin filaments in bacteria. Cell *177*, 1771–1780.e12.

28. Chaikeeratisak, V., Khanna, K., Nguyen, K.T., Egan, M.E., Enustun, E., Armbruster, E., Lee, J., Pogliano, K., Villa, E., and Pogliano, J. (2022). Subcellular organization of viral particles during maturation of nucleus-forming jumbo phage. Sci. Adv. *8*, eabj9670.

29. Sharma, R., Pielstick, B.A., Bell, K.A., Nieman, T.B., Stubbs, O.A., Yeates, E.L., Baltrus, D.A., and Grose, J.H. (2019). A novel, highly related jumbo family of bacteriophages that were isolated against Erwinia. Front. Microbiol. *10*, 1533.

30. Vanneste, J.L. (2000). Fire Blight: The Disease and its Causative Agent, Erwinia Amylovora (CABI).

31. Arens, D.K., Brady, T.S., Carter, J.L., Pape, J.A., Robinson, D.M., Russell, K.A., Staley, L.A., Stettler, J.M., Tateoka, O.B., Townsend, M.H., et al. (2018). Characterization of two related Erwinia myoviruses that are distant relatives of the PhiKZ-like Jumbo phages. PLoS One *13*, e0200202.

32. Esplin, I.N.D., Berg, J.A., Sharma, R., Allen, R.C., Arens, D.K., Ashcroft, C.R., Bairett, S.R., Beatty, N.J., Bickmore, M., Bloomfield, T.J., et al. (2017). Genome sequences of 19 novel Erwinia amylovora bacteriophages. Genome Announc. *5*, e00931-17. https://doi.org/10.1128/genomeA.00931-17.

33. Konstantinidis, K.T., and Tiedje, J.M. (2005). Genomic insights that advance the species definition for prokaryotes. Proc. Natl. Acad. Sci. USA *102*, 2567–2572.

34. Park, S.-C., Lee, K., Kim, Y.O., Won, S., and Chun, J. (2019). Large-scale genomics reveals the genetic characteristics of seven species and importance of phylogenetic distance for estimating Pan-genome size. Front. Microbiol. *10*, 834.

35. Comeau, A.M., Bertrand, C., Letarov, A., Tétart, F., and Krisch, H.M. (2007). Modular architecture of the T4 phage superfamily: a conserved core genome and a plastic periphery. Virology *362*, 384–396.

36. Cazares, A., Mendoza-Hernández, G., and Guarneros, G. (2014). Core and accessory genome architecture in a group of Pseudomonas aeruginosa Mu-like phages. BMC Genom. *15*, 1146.

37. Mathee, K., Narasimhan, G., Valdes, C., Qiu, X., Matewish, J.M., Koehrsen, M., Rokas, A., Yandava, C.N., Engels, R., Zeng, E., et al. (2008). Dynamics of Pseudomonas aeruginosa genome evolution. Proc. Natl. Acad. Sci. USA *105*, 3100–3105.

38. Jang, H.B., Fagutao, F.F., Nho, S.W., Park, S.B., Cha, I.S., Yu, J.E., Lee, J.S., Im, S.P., Aoki, T., and Jung, T.S. (2013). Phylogenomic network and comparative genomics reveal a diverged member of the ΦKZ-related group, marine vibrio phage ΦJM-2012. J. Virol. *87*, 12866–12878.

39. Iyer, L.M., Anantharaman, V., Krishnan, A., Burroughs, A.M., and Aravind, L. (2021). Jumbo phages: a comparative genomic overview of core functions and adaptions for biological conflicts. Viruses *13*, 63. https://doi.org/10.3390/v13010063.

40. Chaikeeratisak, V., Birkholz, E.A., Prichard, A.M., Egan, M.E., Mylvara, A., Nonejuie, P., Nguyen, K.T., Sugie, J., Meyer, J.R., and Pogliano, J. (2021). Viral speciation through subcellular genetic isolation and virogenesis incompatibility. Nat. Commun. *12*, 342.

41. Letunic, I., and Bork, P. (2021). Interactive Tree of Life (iTOL) v5: an online tool for phylogenetic tree display and annotation. Nucleic Acids Res. *49*, W293–W296.

42. van Tonder, A.J., Mistry, S., Bray, J.E., Hill, D.M.C., Cody, A.J., Farmer, C.L., Klugman, K.P., von Gottberg, A., Bentley, S.D., Parkhill, J., et al. (2014). Defining the estimated core genome of bacterial populations using a Bayesian decision model. PLoS Comput. Biol. *10*, e1003788.

43. Guan, J., Oromí-Bosch, A., Mendoza, S.D., Karambelkar, S., Berry, J., and Bondy-Denomy, J. (2022). RNA targeting with CRISPR-Cas13a facilitates bacteriophage genome engineering. Preprint at bioRxiv. https://doi.org/10.1101/2022.02.14.480438.

44. Fokine, A., Battisti, A.J., Bowman, V.D., Efimov, A.V., Kurochkina, L.P., Chipman, P.R., Mesyanzhinov, V.V., and Rossmann, M.G. (2007). Cryo-EM study of the Pseudomonas bacteriophage phiKZ. Structure *15*, 1099–1104.

45. Barylski, J., Enault, F., Dutilh, B.E., Schuller, M.B., Edwards, R.A., Gillis, A., Klumpp, J., Knezevic, P., Krupovic, M., Kuhn, J.H., et al. (2020). Analysis of spounaviruses as a case study for the overdue reclassification of tailed phages. Syst. Biol. *69*, 110–123.

46. Krylov, V.N., and Zhazykov, I.Z. (1978). Pseudomonas bacteriophage phiKZ–possible model for studying the genetic control of morphogenesis. Genetika *14*, 678–685.

47. Blazanin, M., Lam, W.T., Vasen, E., Chan, B.K., and Turner, P.E. (2022). Decay and damage of therapeutic phage OMKO1 by environmental stressors. PLoS One *17*, e0263887.

48. Latz, S., Krüttgen, A., Häfner, H., Buhl, E.M., Ritter, K., and Horz, H.-P. (2017). Differential effect of newly isolated phages belonging to PB1-like, phiKZ-like and LUZ24-like viruses against multi-drug resistant Pseudomonas aeruginosa under varying growth conditions. Viruses *9*, 315. https://doi.org/10.3390/v9110315.

49. Danis-Wlodarczyk, K., Vandenheuvel, D., Jang, H.B., Briers, Y., Olszak, T., Arabski, M., Wasik, S., Drabik, M., Higgins, G., Tyrrell, J., et al. (2016). A proposed integrated approach for the preclinical evaluation of phage therapy in Pseudomonas infections. Sci. Rep. *6*, 28115.

50. Olsen, N.S., Hendriksen, N.B., Hansen, L.H., and Kot, W. (2020). A new high-throughput screening method for phages: enabling crude isolation and fast identification of diverse phages with therapeutic potential. Phage (New Rochelle) *1*, 137–148.

51. Vaitekenas, A., Tai, A.S., Ramsay, J.P., Stick, S.M., Agudelo-Romero, P., and Kicic, A.WAERP; AREST CF (2022). Complete genome sequences of four Pseudomonas aeruginosa bacteriophages: Kara-mokiny 8, Kara-mokiny 13, Kara-mokiny 16, and Boorn-mokiny 1. Microbiol. Resour. Announc. *11*, e0096022.

52. Aghaee, B.L., Khan Mirzaei, M., Alikhani, M.Y., Mojtahedi, A., and Maurice, C.F. (2021). Improving the inhibitory effect of phages against Pseudomonas aeruginosa isolated from a burn patient using a combination of phages and antibiotics. Viruses *13*, 334. https://doi.org/10.3390/v13020334.

53. Erickson, H.P., Anderson, D.E., and Osawa, M. (2010). FtsZ in bacterial cytokinesis: cytoskeleton and force generator all in one. Microbiol. Mol. Biol. Rev. *74*, 504–528.

54. Du, S., and Lutkenhaus, J. (2019). At the heart of bacterial cytokinesis: the Z ring. Trends Microbiol. *27*, 781–791.

55. Haeusser, D.P., and Margolin, W. (2016). Splitsville: structural and functional insights into the dynamic bacterial Z ring. Nat. Rev. Microbiol. *14*, 305–319.

56. Larsen, R.A., Cusumano, C., Fujioka, A., Lim-Fong, G., Patterson, P., and Pogliano, J. (2007). Treadmilling of a prokaryotic tubulin-like protein, TubZ, required for plasmid stability in Bacillus thuringiensis. Genes Dev. *21*, 1340–1352.

57. Aylett, C.H.S., Wang, Q., Michie, K.A., Amos, L.A., and Löwe, J. (2010). Filament structure of bacterial tubulin homologue TubZ. Proc. Natl. Acad. Sci. USA *107*, 19766–19771.

58. Montabana, E.A., and Agard, D.A. (2014). Bacterial tubulin TubZ-Bt transitions between a two-stranded intermediate and a four-stranded filament upon GTP hydrolysis. Proc. Natl. Acad. Sci. USA *111*, 3407–3412.

59. Schlieper, D., Oliva, M.A., Andreu, J.M., and Löwe, J. (2005). Structure of bacterial tubulin BtubA/B: evidence for horizontal gene transfer. Proc. Natl. Acad. Sci. USA *102*, 9170–9175.

60. Yee, B., Lafi, F.F., Oakley, B., Staley, J.T., and Fuerst, J.A. (2007). A canonical FtsZ protein in Verrucomicrobium spinosum, a member of the Bacterial phylum Verrucomicrobia that also includes tubulin-producing Prosthecobacter species. BMC Evol. Biol. *7*, 37.

61. Díaz-Celis, C., Risca, V.I., Hurtado, F., Polka, J.K., Hansen, S.D., Maturana, D., Lagos, R., Mullins, R.D., and Monasterio, O. (2017). Bacterial tubulins A and B exhibit polarized growth, mixed-polarity bundling, and destabilization by GTP hydrolysis. J. Bacteriol. *199*, e00211-17. https://doi.org/10.1128/JB.00211-17.

62. Falco, S.C., Zehring, W., and Rothman-Denes, L.B. (1980). DNA-dependent RNA polymerase from bacteriophage N4 virions. Purification and characterization. J. Biol. Chem. *255*, 4339–4347.

63. Molineux, I.J., and Panja, D. (2013). Popping the cork: mechanisms of phage genome ejection. Nat. Rev. Microbiol. *11*, 194–204.

64. Jin, Y., Sdao, S.M., Dover, J.A., Porcek, N.B., Knobler, C.M., Gelbart, W.M., and Parent, K.N. (2015). Bacteriophage P22 ejects all of its internal proteins before its genome. Virology *485*, 128–134.

65. Nishimura, Y., Yoshida, T., Kuronishi, M., Uehara, H., Ogata, H., and Goto, S. (2017). ViPTree: the viral proteomic tree server. Bioinformatics *33*, 2379–2380.

66. Meier-Kolthoff, J.P., and Göker, M. (2017). VICTOR: genome-based phylogeny and classification of prokaryotic viruses. Bioinformatics *33*, 3396–3404.

67. Altschul, S.F., Madden, T.L., Schäffer, A.A., Zhang, J., Zhang, Z., Miller, W., and Lipman, D.J. (1997). Gapped BLAST and PSI-BLAST: a new generation of protein database search programs. Nucleic Acids Res. *25*, 3389–3402.

68. Kelley, L.A., Mezulis, S., Yates, C.M., Wass, M.N., and Sternberg, M.J.E. (2015). The Phyre2 web portal for protein modeling, prediction and analysis. Nat. Protoc. *10*, 845–858.

69. Madeira, F., Pearce, M., Tivey, A.R.N., Basutkar, P., Lee, J., Edbali, O., Madhusoodanan, N., Kolesnikov, A., and Lopez, R. (2022). Search and sequence analysis tools services from EMBL-EBI in 2022. Nucleic Acids Res. *50*, W276–W279. https://doi.org/10.1093/nar/gkac240.

70. Robert, X., and Gouet, P. (2014). Deciphering key features in protein structures with the new ENDscript server. Nucleic Acids Res. *42*, W320–W324.

71. Lam, V., and Villa, E. (2021). Practical approaches for cryo-FIB milling and applications for cellular cryo-electron tomography. Methods Mol. Biol. *2215*, 49–82.

72. Mastronarde, D.N. (2005). Automated electron microscope tomography using robust prediction of specimen movements. J. Struct. Biol. *152*, 36–51.

73. Liu, Y.-T., Zhang, H., Wang, H., Tao, C.-L., Bi, G.-Q., and Zhou, Z.H. (2022). Isotropic reconstruction for electron tomography with deep learning. Nat. Commun. *13*, 6482.

74. Martinez-Sanchez, A., Garcia, I., Asano, S., Lucic, V., and Fernandez, J.-J. (2014). Robust membrane detection based on tensor voting for electron tomography. J. Struct. Biol. *186*, 49–61. https://doi.org/10.1016/j.jsb.2014.02.015.

75. Goddard, T.D., Huang, C.C., Meng, E.C., Pettersen, E.F., Couch, G.S., Morris, J.H., and Ferrin, T.E. (2018). UCSF ChimeraX: meeting modern challenges in visualization and analysis. Protein Sci. *27*, 14–25.

76. Jumper, J., Evans, R., Pritzel, A., Green, T., Figurnov, M., Ronneberger, O., Tunyasuvunakool, K., Bates, R., Žídek, A., Potapenko, A., et al. (2021). Highly accurate protein structure prediction with AlphaFold. Nature *596*, 583–589.

77. Schrödinger, L.L.C. (2021). The PyMOL Molecular Graphics System, Version 2.5.2.

78. Tegunov, D., and Cramer, P. (2019). Real-time cryo-electron microscopy data preprocessing with Warp. Nat. Methods *16*, 1146–1152.

CellPress
OPEN ACCESS

79. Kremer, J.R., Mastronarde, D.N., and McIntosh, J.R. (1996). Computer visualization of three-dimensional image data using IMOD. J. Struct. Biol. *116*, 71–76.

80. Scheres, S.H.W. (2012). RELION: implementation of a Bayesian approach to cryo-EM structure determination. J. Struct. Biol. *180*, 519–530.

81. Bharat, T.A.M., Russo, C.J., Löwe, J., Passmore, L.A., and Scheres, S.H.W. (2015). Advances in single-particle electron cryomicroscopy structure determination applied to sub-tomogram averaging. Structure *23*, 1743–1753. https://doi.org/10.1016/j.str.2015.06.026.

82. Tegunov, D., Xue, L., Dienemann, C., Cramer, P., and Mahamid, J. (2021). Multi-particle cryo-EM refinement with M visualizes ribosome-antibiotic complex at 3.5 Å in cells. Nat. Methods *18*, 186–193.

83. Navarro, P., Scaramuzza, S., Stahlberg, H., and Castaño-Díez, D. (2020). The dynamo software package for cryo-electron tomography and subtomogram averaging. Microsc. Microanal. *26*, 3142–3145. https://doi.org/10.1017/s1431927620023958.

84. Burt, A., Gaifas, L., Dendooven, T., and Gutsche, I. (2021). A flexible framework for multi-particle refinement in cryo-electron tomography. PLoS Biol. *19*, e3001319.

85. Pyle, E., Hutchings, J., and Zanetti, G. (2022). Strategies for picking membrane-associated particles within subtomogram averaging workflows. Faraday Discuss *240*, 101–113. https://doi.org/10.1039/d2fd00022a.

86. Kovtun, O., Leneva, N., Bykov, Y.S., Ariotti, N., Teasdale, R.D., Schaffer, M., Engel, B.D., Owen, D.J., Briggs, J.A.G., and Collins, B.M. (2018). Structure of the membrane-assembled retromer coat determined by cryo-electron tomography. Nature *561*, 561–564. https://doi.org/10.1038/s41586-018-0526-z.

87. Chen, S., McMullan, G., Faruqi, A.R., Murshudov, G.N., Short, J.M., Scheres, S.H.W., and Henderson, R. (2013). High-resolution noise substitution to measure overfitting and validate resolution in 3D structure determination by single particle electron cryomicroscopy. Ultramicroscopy *135*, 24–35. https://doi.org/10.1016/j.ultramic.2013.06.004.

88. Goddard, T.D., Huang, C.C., and Ferrin, T.E. (2007). Visualizing density maps with UCSF Chimera. J. Struct. Biol. *157*, 281–287.

STAR★METHODS

KEY RESOURCES TABLE

REAGENT or RESOURCE	SOURCE	IDENTIFIER
Bacterial and virus strains		
Erwinia amylovora	American Type Culture Collection	ATCC 29780
Erwinia phage RAY	Julie Grose	vB_EamM_RAY
Chemicals, peptides, and recombinant proteins		
DAPI (4',6-diamidino-2-phenylindole dihydrochloride)	Life Technologies	Cat#D21490
FM4-64	Thermo Scientific	Cat#T13320
Gentamicin	MP Biomedicals, LLC	Cat#190057
Arabinose	Sigma Aldrich	Cat#A91906
Deposited data		
Fluorescence microscopy	This study	https://doi.org/10.17632/jd4yj84463.1
Cryo-ET tilt series	This study	EMPIAR-11198
Set of proteins encoded in the genomes of all tailed phages [taxid: 28883]	NCBI	https://www.ncbi.nlm.nih.gov/protein/?term=txid28883[organism:exp]
Recombinant DNA		
pHERD-30T plasmid	Qiu D, Damron FH, Mima T, Schweizer HP, Yu HD.	https://novoprolabs.com/vector/Vg4ydcni
Table S4	This study	N/A
Software and algorithms		
Core genome identification script	This study	https://doi.org/10.5281/zenodo.7787482
DeltaVision softWoRx 6.5.2	GE HealthCare	http://incelldownload.gehealthcare.com/bin/download_data/SoftWoRx/7.0.0/SoftWoRx.htm
FIJI (ImageJ 1.52p)	Wayne Rasband, National Institutes of Health, USA	http://imagej.nih.gov/ij
GraphPad Prism version 9.4.1	GraphPad Software, San Diego, California, USA	www.graphpad.com
Benchling	Benchling	benchling.com
Microsoft Excel	Microsoft Office	http://www.microsoft.com/en-us/microsoft-365/excel
Microsoft PowerPoint	Microsoft Office	http://www.microsoft.com/en-us/microsoft-365/powerpoint
Microsoft Word	Microsoft Office	http://www.microsoft.com/en-us/microsoft-365/word
Adobe Illustrator	Adobe	http://www.adobe.com/products/illustrator.html
Adobe Photoshop	Adobe	http://www.adobe.com/products/photoshop.html
R-v1.2.1335	CRAN	https://cran.r-project.org/bin/windows/base
Python-v3.9.11	Python	https://www.python.org
PSI-BLAST-v2.12.0	Bioconda	https://anaconda.org/bioconda/blast
IMG/VR	JGI	https://img.jgi.doe.gov/cgi-bin/vr/main.cgi
SerialEM-v3.8.7	PMID: 16182563	bio3d.colorado.edu/SerialEM/
IsoNet-v0.1	https://doi.org/10.1101/2021.07.17.452128	https://zenodo.org/record/7016051#.Y_fE8x_MKUk
Warp-v1.09	PMID: 31591575	www.warpem.com/warp/
TomoSegMemTV	https://doi.org/10.1016/j.jsb.2014.02.015	http://sites.google.com/site/3demimageprocessing/tomosegmemtv
Amira3D-v2021.2	Thermo Fisher Scientific	http://www.thermofisher.com/us/en/home/electron-microscopy/products/software-em-3d-vis/amira-software.html

(*Continued on next page*)

Continued

REAGENT or RESOURCE	SOURCE	IDENTIFIER
Dynamo-v1.1514	https://doi.org/10.1017/s1431927620023958	https://wiki.dynamo.biozentrum.unibas.ch/w/index.php/Main_Page
ChimeraX-v1.3 or v1.4	PMID: 28710774	http://www.rbvi.ucsf.edu/chimerax/
AlphaFold v2.1.0	DeepMind	https://colab.research.google.com/github/deepmind/alphafold/blob/main/notebooks/AlphaFold.ipynb
PyMOL version 2.5.2	Schrödinger	https://pymol.org/2/
IMOD-v4.10.28	PMID: 8742726	http://bio3d.colorado.edu/imod/
RELION-v3.13	https://doi.org/10.1016/j.jsb.2012.09.006 and https://doi.org/10.1016/j.str.2015.06.026	http://relion.readthedocs.io/en/release-3.1/
M-v1.09	PMID: 33542511	https://warpem.com/warp/#
dynamo2m-v0.2.2	PMID: 34437530	http://github.com/alisterburt/dynamo2m
UCSF-Chimera-v1.15	PMID: 16963278	https://www.cgl.ucsf.edu/chimera/download.html

RESOURCE AVAILABILITY

Lead contact

Further information and requests for resources and reagents should be directed to and will be fulfilled by the lead contact, Joe Pogliano (jpogliano@ucsd.edu).

Materials availability

This study did not generate new unique reagents.

Data and code availability

- All raw fluorescence microscopy images used in this paper are deposited in a Mendeley dataset (Mendeley Data: https://doi.org/10.17632/jd4yj84463.1). Tilt-series frames and alignment metadata for the RAY-infected *E. amylovora* cells are deposited with the Electron Microscopy Public Image Archive with accession number EMPIAR-11198. All subtomogram averaging maps from this study are deposited with the Electron Microscopy Data Bank with the following accession numbers: *E. amylovora* 70S (EMD-27973), *E. amylovora* 50S (EMD-27993), RAY capsid vertex (EMD-28003), RAY collar (EMD-28004), RAY tail sheath (EMD-28005), RAY baseplate (EMD-28006), RAY chimallin (EMD-28007), putative RAY PhuZ (EMD-28008), 201φ2-1 PhuZ (28009), and ΦKZ PhuZ (28010). The composite RAY virion map is deposited as an additional map with the RAY capsid vertex (EMD-28003).
- All original code used for core genome determination PSI-BLAST has been deposited on GitHub (GitHub: https://github.com/jina-leemon/core-genome-proj). Raw data of the PSI-BLAST results used for core genome determination can be found in Data S2.
- Any additional information required to reanalyze the data reported in this paper is available from the Lead Contact upon request.

EXPERIMENTAL MODEL AND SUBJECT DETAILS

Bacterial growth and phage infection conditions

Erwinia amylovora ATCC 29780 was grown on LB plates at room temperature or in LB liquid cultures at 30°C. To collect RAY lysates, 0.5 mL of dense *E. amylovora* culture grown in liquid LB media at 30°C overnight was incubated with 10 μL of RAY serial dilutions, incubated for 15 minutes at room temperature, and mixed with 4.5 mL molten (approximately 55°C) LB 0.35% top agar. This mixture was quickly poured over standard LB plates and incubated overnight at room temperature. The following day, 5 mL phage buffer was poured over plates showing web lysis and incubated at room temperature for 5 hours. The phage lysate was collected by aspiration and centrifuged for 10 min at 3220 rcf to pellet cell debris. The resulting clarified phage lysate was filtered through a 0.45 μm filter to remove any residual bacterial contamination and stored at 4°C.

Expression of phage proteins

To visualize phage proteins in *Erwinia amylovora*, the proteins were fused to GFPmut1 and expressed from the pHERD-30T plasmid under the inducible control of the AraBAD promoter. GFP fusions were designed using Benchling with the GFP tag attached at either the N- or C-terminus depending on the protein. The plasmids were then synthesized by GenScript. Plasmids were transformed into

electrocompetent *Erwinia amylovora* cells via electroporation. The cells were plated on LB plates with 15 μg/mL gentamicin sulfate as a selectable marker for transformants.

METHOD DETAILS

Genome alignments and phylogenetic analysis

To find close and distant relatives of RAY, we used ViPTree[65] to find phage genomes related to RAY (Figure S8) and Position-Specific Interative Basic Local Alignment Search Tool (PSI-BLAST) to find phage encoding chimallin homologs (Table S1) and msRNAP homologs since all chimallin-encoding phages also encode msRNAPs[14,38,39] PSI-BLAST iterations were performed until no new sequences were found above the 0.005 E-value threshold (for chimallin, the highest E-value in the results was 1e-08). These results were checked for validity by multiple sequence alignments with previously identified homologs to ensure they were not false positives. *Bacillus* phage PBS1, *Staphylococcus* phage PALS_2, and *Yersinia* phage ΦR1-37 were used as the representative phages that encode msRNAPs but not chimallin homologs, and well-studied *Escherichia* phage T4, giant *Bacillus* phage G, and *Erwinia* phage vB_EamM_Alexandra were also included in our analysis for comparison. VICTOR[66] predicted the species assignments, which were used to determine which phage could be discluded to keep the trees readable; genus assignments, which were used to color-code the trees; and subfamily and family assignments, which were used along with core genome analysis to define the family Chimalliviridae (Table S2). Phylogenetic trees were made using VICTOR (Figure 1A) and colored using Photoshop (Adobe) or Clustal Omega (Figures 1B–1D, S1, S2, and S5) and colored using iTOL.[41] Data on metagenomes was found by BLASTing $ChmA_{RAY}$ in IMG/VR and collecting the high confidence results.

Core genome determination

Bulk PSI-BLAST in the standalone BLAST+ Suite[67] was performed on the tailed phages taxon (taxid: 28883) with RAY proteins as the query, a max iteration of 4, and an E-value cutoff of 0.05. Then two scripts were run on the result to search for proteins from the phages of interest as well as output the ones present in more than 95% of the phages through a text mining approach. Scripts are available on GitHub (https://github.com/jina-leemon/core-genome-proj). Blocks were defined as 3 or more conserved genes, tolerating a maximum of 3 non-conserved genes between. The core genome numbers (Table S3) were assigned depending on the homologs that showed up through PSI-BLAST in ΦKZ, Goslar, and RAY.

Phage protein characterization

PSI-BLAST was used in order to identify potential homologs of each unknown RAY protein.[67] The PSI-BLASTs were performed with the non-redundant protein database, limited to tailed phages (taxid: 28883), excluding uncultured and environmental sample sequences, and with a maximum of 5000 sequences. Iterations were run excluding results with E-values lower than the default PSI-BLAST threshold, stopping when results converged. For each protein studied, $Phyre^2$ was used in order to predict secondary structure and to identify known proteins with similar structures.[68] The amino acid sequences of each protein were uploaded to $Phyre^2$ with the normal modeling mode. $Phyre^2$ results with confidence higher than 70% were compared to PSI-BLAST results as an independent method for predicting the putative functions of unannotated RAY proteins. Using the potential homologs of each unknown RAY protein identified by PSI-BLAST, multiple sequence alignments were created using Clustal Omega[69] to align homologs from previously studied nucleus-forming phages 201φ2-1, ΦKZ, ΦPA3, PCH45, and Goslar. If one or more of these phages were missing a homolog of the query protein (as was the case with some of the non-core genome proteins), a close relative of RAY (*Erwinia* phage AH06) and/or a distant relative of RAY (*Klebsiella* phage Miami) were supplemented to fill out the alignment when possible. The resulting multiple sequence alignments were uploaded to ESPript for visualization using the default settings and downloaded as PDF files.[70]

Fluorescence microscopy

E. amylovora cells were inoculated onto imaging pads in welled microscopy slides. Pads were made up of 1% agarose, 25% LB, 2 μg/mL FM4-64, and 0.1 μg/mL DAPI. Between 0-1% arabinose was used to induce expression from the pHERD-30T plasmid, depending on the construct. The slides were incubated at 30°C for 3 hours, then moved to room temperature for infection with 10 μL undiluted RAY lysate. Slides were imaged using the DeltaVision Elite deconvolution microscope (Applied Precision) and deconvolved using the aggressive algorithm in the DeltaVision softWoRx program (Applied Precision). Image analysis was performed on images prior to deconvolution.

Cryo-electron microscopy of RAY-infected cells

E. amylovora cells were infected on agarose pads as previously described[7] with a few changes. Briefly, cells were grown on agarose pads as described above for fluorescence microscopy and infected at room temperature with 10 μL undiluted RAY lysate. 60 minutes post infections, 25 μL 25% LB was added to each pad and cells were gently scraped off with the bottom of a 1.7 mL eppendorf tube. A droplet containing cells was collected from each pad and an aliquot was saved as an unconcentrated sample. The remainder was centrifuged at 8000 rcf for 30 sec, and resuspended in a portion of the supernatant to concentrate the cells.

Infected cell suspension was mixed 9:1 with 50% (w/v) trehalose solution to mitigate crystalline ice formation and 4 μL of this mixture immediately applied to a R2/1 Cu 200 grid (Quantifoil), which had been glow-discharged for 1 min at 0.19 mbar and 20 mA in a PELCO

easiGlow device shortly before use. The grid was then mounted in a custom-built manual plugning device (MPI-Biochemistry, Martinsreid, Germany) and excess media wicked away with filter paper (Whatman #1) from the backside of the grid for 4-7 seconds prior to plunging the grid into a 50:50 ethane:propane mixture (Airgas) cooled by liquid nitrogen. The data presented in this manuscript were collected from two independent preparations of RAY-infected *E. amylovora* at approximately 100-110 mpi.

Frozen grids of infected cells were mounted into notched Autogrids (TFS) compatible with cryo-focused ion beam milling. Samples were loaded into an Aquilos 2 cryo-focused ion beam/scanning electron microscope (TFS) with a Gallium ion source and milled to generate lamellae using progressively lower milling currents from 0.5 nA to 10 pA as previously described.[71]

For tilt-series collection, samples were transferred to a Titan Krios G3 transmission electron microscope (TFS) operated at 300 kV, configured for fringe-free illumination, and equipped with a K2 direct electron detector (Gatan) mounted post a Quantum 968 LS imaging filter (Gatan). The microscope was operated in EFTEM mode with a slit-width of 20 eV and using a 70 μm objective aperture. Automated data acquisition was performed using SerialEM-v3.8.7[72] and all images were acquired using the K2 in counting mode. Tilt-series were acquired at 4.27 Å/pixel following a dose-symmetric scheme to span a nominal range of either +/− 57° (dataset-1) or +/− 64° (dataset-2) in 2° steps. The exposure was uniformly distributed across tilt-images, achieving totals of approximately 150 e/Å^{-2} (dataset-1) or 170 e/Å^{-2} (dataset-2). Target defoci for tilt-series were between 4 and 6 μm. A total of 32 tilt-series were acquired, of which 26 (13 from each session) were deemed suitable for subsequent processing.

Tomogram segmentation

To aid in segmentation and for display purposes, tomograms were missing-wedge corrected using IsoNet-v0.1[73] on tomograms reconstructed at 20 Å/px and devolved with Warp-v1.09. Membranes were initially segmented using TomoSegMemTV[74] and patched manually using Amira3D-v2021.2 (TFS). Subtomogram averages were placed at refined particle positions using Dynamo-v1.1514. The segmentation was rendered using ChimeraX-v1.3.[75]

Structural predictions

AlphaFold v2.1.0[76] was used in order to predict the tertiary structure of unsolved RAY proteins. We ran ChmA$_{RAY}$ (gp222) and PhuZ$_{RAY}$ (gp210) through AlphaFold v2.1.0 to obtain the predicted structure of the proteins. These predictions were then imported into PyMOL Version 2.5.2[77] in order to visualize the structures. Structural alignments between previously published protein structures and RAY protein structural predictions were made using PyMOL's align function, and the RMSD values of these alignments were found using the same method.

QUANTIFICATION AND STATISTICAL ANALYSIS

Nucleus rotation analysis

Time lapses (2 minutes long with 4-second intervals) were obtained, generally between 60-90 mpi. The time lapses that included actively rotating nuclei were analyzed using FIJI. The segmented line tool was used to measure all distances. To determine the nucleus rotation speed, a distinct point on the surface of the nucleus was tracked frame by frame to measure the total distance traveled. The number of frames used and the time lapse interval size were used to determine the rotation time. The rotation speed was then calculated by dividing the total distance traveled by the rotation time. The total number of cells with nuclei and the number of nuclei that were actively rotating were manually quantified to determine the percentage of rotating nuclei. The angular velocity was determined by dividing the total distance traveled by the length of the nucleus' radius (half of the nucleus' diameter).

PhuZ filamentation and nucleus positioning analysis

For the filamentation analysis, the number of cells with filaments versus without filaments was manually quantified from microscopy images, and the percentage of cells with filaments at different arabinose concentrations was recorded. A filament was defined as a visible line that was at least double the level of background GFP fluorescence. The data were graphed using Prism (GraphPad), and a nonlinear [Agonist] vs. response curve was fit to represent the [Arabinose] vs. filamentation data.

For the nucleus positioning analysis, the length of the cell and the distance between the center of the phage nucleus and the cell pole was measured using FIJI. The nucleus position was calculated as the ratio of the distance from the phage nucleus to the pole over the entire cell length. The data were graphed as a relative frequency histogram using Prism (GraphPad).

Subtomogram analysis

All pre-processing steps were performed using Warp-v1.09[78] unless otherwise specified. Tilt-movies were corrected for whole-frame motion, their defocus values estimated, and stacked into tilt-series. Tilt-series were aligned via patch-tracking with Etomo (IMOD-v4.10.28).[79] Tomograms were reconstructed with the default deconvolution filter settings for visualization and without for template-matching and subsequent processing, when necessary.

First, an *ab initio* host 70S ribosome reference was generated from the data by manually picking particles and aligning them in RELION-v3.1.3.[80,81] A low-pass filtered (50 Å) reference was used for template-matching across the entire dataset and the hits curated by Figure-Of-Merit score to remove false-positives. The 51,512 template-matching hits were classified in RELION-v3.1.3 without a starting reference to further remove false positives. Alignment of the 28,032 selected particles resulted in a 18 Å map of

the 70S ribosome. Further refinement of particle poses and tilt-series parameters in M-v1.09[82] improved the map to 10.3 Å. This map was low-pass filtered and used for a second round of template-matching against the tomograms, which resulted in 74,713 hits after curation. The new particle set was coarsely aligned with RELION-v3.1.3 and followed by M-v1.0.9, which resulted in a 9.5 Å map. Further alignment with RELION-v3.1.3 improved the map to 9.2 Å. Reference-free classification without alignment in identified 45,708 70S particles which refined to 8.9 Å and 2,551 50S particles which refined to 13.4 Å.

For virion capsids, 156 particle positions were manually picked and aligned while enforcing icosahedral symmetry in RELION-v3.1.3. The vertices were subsequently extracted to create 1,872 sub-particles, which aligned with C5 symmetry and then classified in C1 without a starting reference nor additional alignment. The 1,056 selected particles were further classified without a starting reference using an ellipsoid mask, C6 symmetry with relaxed C5 symmetry, and local-searches to separate pentameric vertices from the portal vertices. The 1,023 pentameric vertices were further aligned with C5 symmetry to yield a 19 Å map. The 22 portal vertices were aligned with C6 symmetry to yield a 38 Å map.

For virion tails, the start and endpoints of nine tails exhibiting clear polarity in the tomograms were picked and filament cropping models generated using Dynamo-v1.1514.[83] Subtomograms were extracted every 2 nm to yield 900 segments and the azimuth angles randomized. An initial reference was generated by reconstruction of the segments and smoothing of the resulting map using a Gaussian filter of 3 pixel width. The smoothed map was used to align all segments without enforced symmetry and restricted angular search range. The resulting map exhibited clear polar and C6 symmetry. Next, the start and endpoints of all tails were picked, filament models generated, and subtomograms extracted every 2 nm with randomized azimuth angles. A copy of the metadata table was generated in which the polarity of each filament was flipped. Both tables were aligned for a single iteration against the previously generated reference with C6 symmetry enforced while limiting the alignment to 40 Å. Duplicate particles which had converged on the same position due to the initial oversampling and subsequent alignment step were removed. In order to determine the polarity of segments, the cross-correlation (CC) values for analogous tails between the two tables were compared. For each tail, the orientation leading to the higher median CC across all its segments was chosen for subsequent steps. For tails exhibiting similar, low median CC values in both orientations were discarded. A CC threshold was selected for the entire dataset to remove these ambiguous tails and other low CC segments. This was typical of tails only partially contained within the lamellae, for which a majority was either FIB-ablated or extended outside of the field-of-view. The resulting 3,291 segments were converted for processing in RELION-v3.1.3 using dynamo2m-v0.2.2[84] and halfsets were assigned on a per-tail basis. Refinement in RELION-v3.1.3 while enforcing C6 symmetry resulted in a 10.4 Å map. Reference-free classification with C6 symmetry and without alignment separated tail segments from baseplates. Alignment of the selected 3,257 tails segments with C6 symmetry resulted in a 10 Å map which exhibits a rise of 37.4 Å and twist of 22.3°. The 34 baseplates were aligned with C6 symmetry to yield 36 Å map.

For ChmA, the surfaces of the twenty phage nuclei were coarsely contoured to generate surface cropping models with Dynamo-v1.1514. Subtomograms were extracted every 4 nm and oriented normal to the surface models. An initial reference was generated by reconstruction of the segments and smoothing of the resulting map using a Gaussian filter of 3 pixel width. A subset of 3,910 segments from two tomograms were aligned against the smoothed map with a restricted angular search range to prevent flipping of sidedness and without enforced symmetry. The resulting map exhibited apparent p442 lattice symmetry with an approximate 11.5 nm spacing, which was corroborated by inspection of 'neighbor plots'.[85,86] This new reference was C4 symmeterized and used to align the entire dataset of 123,416 oversampled segments for a single iteration while limiting alignment to 40 Å. Duplicate particles which had converged on the same position due to the initial oversampling and subsequent alignment step were removed to leave 52,646 segments. Misaligned and edge segments were then removed by selecting only segments with at least three neighboring segments with 10–13 nm, which retained 23,509 segments. The metadata was converted for processing in RELION-v3.1.3 using dynamo2m-v0.2.2. Further alignment in RELION-v3.1.3 enforcing C4 symmetry yielded a 20 Å map.

For PhuZ, the start and endpoints of 14 filaments were manually picked from the tomograms and cropping models generated with Dynamo-v1.1514. The polarity of the filaments was uniformly assigned from the cell pole to the mid-cell. Segments were extracted every 1 nm along the filaments and the azimuth angles were randomized, which resulted in 5,000 subtomograms. Sub-tomograms were aligned to the unaligned average reconstructed from the initial cropping points with Dynamo-v1.1514 while limiting the alignment resolution to 40 Å and preventing subtomograms from flipping polarity. Duplicate particles which had converged on the same position due to the initial oversampling and subsequent alignment step were removed, which resulted in 3,080 remaining subtomograms. The metadata was converted for processing in RELION-v3.1.3 using dynamo2m-v0.2.2 and halfsets were assigned on a per-filament basis. Alignment in RELION-v3.1.3 resulted in a 25 Å map. Individual protomers are not resolved in this map and attempts at estimating reliable helical parameters were unsuccessful.

All resolution estimates are based on the 0.143-threshold of the Fourier shell correlation between masked half-maps using high-resolution noise-substitution to mitigate masking artifacts.[87] Local-resolution estimates were commuted using RELION. The virion 'frakenmap' was generated by the *vop maximum* command in UCSF-Chimera-v1.15[88] to combine the various components for display purposes. Maps were rendered using ChimeraX-v1.3 or v1.4.[75]

Cell Reports, Volume *42*

Supplemental information

Identifying the core genome of the nucleus-forming bacteriophage family and characterization of *Erwinia* phage RAY

Amy Prichard, Jina Lee, Thomas G. Laughlin, Amber Lee, Kyle P. Thomas, Annika E. Sy, Tara Spencer, Aileen Asavavimol, Allison Cafferata, Mia Cameron, Nicholas Chiu, Demyan Davydov, Isha Desai, Gabriel Diaz, Melissa Guereca, Kiley Hearst, Leyi Huang, Emily Jacobs, Annika Johnson, Samuel Kahn, Ryan Koch, Adamari Martinez, Meliné Norquist, Tyler Pau, Gino Prasad, Katrina Saam, Milan Sandhu, Angel Jose Sarabia, Siena Schumaker, Aaron Sonin, Ariya Uyeno, Alison Zhao, Kevin D. Corbett, Kit Pogliano, Justin Meyer, Julianne H. Grose, Elizabeth Villa, Rachel Dutton, and Joe Pogliano

Table S1. List of chimallin-encoding phage used in our analyses, Related to Figures 1 and 2. These phage were used for our determination of the Chimalliviridae core genome.

Host	Phage Name	Host	Phage Name
Aeromonas	PS1	Erwinia	pEa_SNUABM_37
Aeromonas	pAEv1810	Erwinia	AH06
Aeromonas	CF8	Erwinia	vB_EamM_RAY
Aeromonas	LAh10	Erwinia	vB_EamM_Joad
Bacillus	vB_BspM_AgentSmith	Erwinia	AH04
Burkholderia	FLC6	Escherichia	vB_EcoM_Goslar
Burkholderia	FLC9	Klebsiella	vB_KvM-Eowyn
Cronobacter	CR5	Klebsiella	vB_KpM_FBKp24
Edwardsiella	pEt-SU	Klebsiella	KpLz-2_45
Erwinia	pEa_SNUABM_29	Klebsiella	Miami
Erwinia	pEa_SNUABM_11	Klebsiella	N1M2
Erwinia	vB_EamM_Asesino	Kosakonia	Kc263
Erwinia	vB_EamM_Huxley	Photobacterium	PDCC-1
Erwinia	pEa_SNUABM_8	Proteus	10
Erwinia	vB_EamM_ChrisDB	Pseudomonas	pPa_SNUABM_DT01
Erwinia	vB_EamM_Caitlin	Pseudomonas	201phi2-1
Erwinia	phiEaH2	Pseudomonas	PhiPA3
Erwinia	Wellington	Pseudomonas	phiKZ
Erwinia	Derbicus	Pseudomonas	PA1C
Erwinia	vB_EamM_Phobos	Pseudomonas	Psa21
Erwinia	pEa_SNUABM_54	Pseudomonas	Phabio
Erwinia	PhiEaH1	Serratia	Moabite
Pseudomonas	Noxifer	Vibrio	JM-2012
Pseudomonas	EL	Vibrio	vB_VmeM-Yong MS32
Pseudomonas	OBP	Vibrio	BONAISHI
Ralstonia	RSL2	Vibrio	vB_pir03
Ralstonia	RSF1	Vibrio	vB_VpaM_sm033
Ralstonia	RP31	Vibrio	pVa-21
Salmonella	STsAS	Vibrio	Aphrodite1
Salmonella	SPAsTU	Vibrio	VP4B
Salmonella	SPN3US	Vibrio	pTD1
Salmonella	vB_SalM_SA002	Xanthomonas	Xoo-sp14
Serratia	PCH45	Xanthomonas	vB_XciM_LucasX

Table S2. VICTOR predictions of taxonomic groups, Related to Figure 1. Predicted family, subfamily, genus, and species categories from VICTOR were used to guide our inclusion of phages in our analyses.

Genomes	species	genus	subfamily	family
Mycoplasma phage P1 (NC_002515)	41	11	3	1
Clostridium phage phiCP7R (NC_017980)	46	14	4	2
Clostridium phage phiCPV4 (NC_018083)	46	14	4	2
Escherichia phage T4 (NC_000866)	40	10	2	3
Bacillus phage G (NC_023719)	51	16	6	3
Erwinia phage vB_EamM_Alexandra (NC_047995)	69	23	10	3
Cronobacter phage vB_CsaP_Ss1 (KM058087)	5	1	1	4
Pectobacterium phage ZF40 (NC_019522)	47	15	5	4
Erwinia phage phiEa21-4 (NC_011811)	63	19	7	4
Erwinia phage phiEa104 (NC_015292)	63	19	7	4
Erwinia phage vB_Eam-MM7 (NC_041978)	63	19	7	4
Yersinia phage vB_YenP_ISAO8 (NC_028850)	53	22	9	4
Yersinia phage phiR8-01 (NC_047951)	68	22	9	4
Salmonella phage ZCSE2 (NC_048179)	74	26	11	4
Salmonella phage SE4 (NC_048764)	74	26	11	4
Salmonella phage BP63 (NC_031250)	77	26	11	4
Salmonella phage UPF_BP2 (NC_048649)	77	26	11	4
Salmonella phage SE13 (NC_048763)	77	26	11	4
Salmonella phage yarpen (NC_048863)	77	26	11	4
Salmonella phage birk (NC_048864)	77	26	11	4
Staphylococcus phage PALS_2 (MN091626)	21	5	8	5
Yersinia phage phiR1-37 (NC_016163)	44	12	8	5
Bacillus phage PBS1 (NC_043027)	67	21	8	5
Burkholderia phage FLC9 (LC667451)	8	2	12	5
Klebsiella phage vB_KvM-Eowyn (LR881104)	9	3	12	5
Vibrio phage vB_VmeM-Yong XC31 (MK308674)	15	4	12	5
Pseudomonas phage pPa_SNUABM_DT01 (MW735835)	30	6	12	5
Bacillus phage vB_BspM_AgentSmith (MW749006)	31	7	12	5
Xanthomonas phage Xoo-sp14 (MT939492)	28	8	12	5
Xanthomonas phage vB_XciM_LucasX (MW825358)	33	8	12	5
Erwinia phage pEa_SNUABM_54 (MW879341)	36	9	12	5
Vibrio phage JM-2012 (NC_017975)	45	13	12	5
Ralstonia phage RP31 (AP017925)	1	17	12	5
Burkholderia phage FLC6 (LC592711)	7	17	12	5
Ralstonia phage RSF1 (NC_028899)	54	17	12	5
Ralstonia phage RSL2 (NC_028950)	55	17	12	5
Salmonella phage vB_SalM_SA002 (MN445183)	23	18	12	5
Proteus phage 10 (MT661596)	25	18	12	5
Erwinia phage pEa_SNUABM_37 (MW845760)	35	18	12	5
Erwinia phage AH06 (MZ501268)	39	18	12	5
Erwinia phage vB_EamM_RAY (NC_041973)	62	18	12	5
Pseudomonas phage 201phi2-1 (EU197055)	2	20	12	5

Pseudomonas phage PhiPA3 (HQ630627)	3	20	12	5
Pseudomonas phage Phabio (MF042360)	10	20	12	5
Pseudomonas phage Psa21 (MK552327)	16	20	12	5
Pseudomonas phage PA1C (MK599315)	17	20	12	5
Pseudomonas phage phiKZ (NC_004629)	42	20	12	5
Pseudomonas phage Noxifer (NC_041994)	64	20	12	5
Escherichia phage vB_EcoM_Goslar (NC_048170)	71	24	12	5
Vibrio phage pVa-21 (KY499642)	6	25	12	5
Salmonella phage STsAS (MH221128)	12	25	12	5
Salmonella phage SPAsTU (MH221129)	13	25	12	5
Erwinia phage pEa_SNUABM_29 (MW812339)	32	25	12	5
Erwinia phage pEa_SNUABM_11 (MW845758)	34	25	12	5
Erwinia phage phiEaH2 (NC_019929)	48	25	12	5
Cronobacter phage CR5 (NC_021531)	49	25	12	5
Salmonella phage SPN3US (NC_027402)	52	25	12	5
Erwinia phage vB_EamM_Phobos (NC_031043)	56	25	12	5
Erwinia phage vB_EamM_Asesino (NC_031107)	57	25	12	5
Erwinia phage vB_EamM_Caitlin (NC_031120)	58	25	12	5
Erwinia phage pEa_SNUABM_8 (MW760841)	59	25	12	5
Erwinia phage vB_EamM_ChrisDB (NC_031126)	59	25	12	5
Erwinia phage vB_EamM_Huxley (NC_031127)	60	25	12	5
Erwinia phage Wellington (NC_048016)	70	25	12	5
Erwinia phage Derbicus (NC_048173)	72	25	12	5
Serratia phage PCH45 (MN334766)	22	27	12	5
Klebsiella phage vB_KpM_FBKp24 (MW394391)	29	27	12	5
Erwinia phage PhiEaH1 (NC_023610)	50	27	12	5
Serratia phage Moabite (NC_048792)	75	27	12	5
Klebsiella phage KpLz-2_45 (NC_061418)	78	27	12	5
Pseudomonas phage OBP (JN627160)	4	28	12	5
Erwinia phage vB_EamM_Joad (MF459647)	11	28	12	5
Aeromonas phage CF8 (MK774614)	18	28	12	5
Aeromonas phage LAh10 (MK838116)	19	28	12	5
Aeromonas phage PS1 (MN032614)	20	28	12	5
Klebsiella phage N1M2 (MN642089)	24	28	12	5
Klebsiella phage Miami (MT701590)	26	28	12	5
Kosakonia phage Kc263 (MZ348422)	37	28	12	5
Erwinia phage AH04 (MZ501267)	38	28	12	5
Pseudomonas phage EL (NC_007623)	43	28	12	5
Vibrio phage pTD1 (NC_041916)	61	28	12	5
Vibrio phage Aphrodite1 (NC_042100)	65	28	12	5
Vibrio phage VP4B (NC_042136)	66	28	12	5
Edwardsiella phage pEtSU (NC_048182)	73	28	12	5
Photobacterium phage PDCC-1 (NC_048821)	76	28	12	5
Aeromonas phage pAEv1810 (OL964756)	79	28	12	5
Vibrio phage BONAISHI (MH595538)	14	29	12	5
Vibrio phage vB_pir03 (MT811961)	27	29	12	5

Vibrio phage vB_VpaM_sm033 (OV032902)	80	29	12	5

Table S3. Core genome blocks by function, Related to Figure 2. The core genome numbers were determined with homologies within and across the genomes of ΦKZ, Goslar, and RAY. Each block is color coded and marked by number, and the last column indicates putative functions from PSI-BLAST hits, many of which are hypothetical. If a core gene has multiple homologs in one phage (for instance, cg42), they will be lettered by numerical order (for instance, RAY gp017 = cg42A, RAY gp018 = cg42B, and RAY gp019 = cg42C). Genes unique to chimallin-encoding phage are marked with an asterisk.

Block Number	Core Genome Number	Goslar	ΦKZ	RAY	Putative Function
1	cg1	gp192	gp049	gp219*	hypothetical protein
	cg2	gp191	gp050	gp220	virion DNAP
	cg3	gp190	gp052	gp221*	hypothetical protein
	cg4	gp189	gp054	gp222*	Nuclear Shell Protein
	cg5	gp188	gp055	gp070, gp223	putative DNA-directed RNA polymerase beta subunit
	cg6	gp184	gp059	gp229*	hypothetical protein
2	cg7	gp180	gp062	gp236*	hypothetical protein
	cg8	gp178	gp065	gp238	putative nuclease
	cg9	gp177	gp066	gp239*	hypothetical protein
	cg10	gp176	gp067	gp240	hypothetical protein
	cg11	gp175	gp068	gp243	putative nvRNAP (non-virion RNAP) sigma factor
	cg12	gp174	gp069	gp244*	hypothetical protein
	cg13	gp173	gp070	gp245	hypothetical protein
	cg14	gp172	n/a	gp246*	hypothetical protein
	cg15	gp171	gp071, gp073	gp248	putative DNA directed RNA polymerase beta subunit
	cg16	gp165	gp074	gp249	putative DNA directed RNA polymerase beta subunit
	cg17	gp164	gp075	gp250	putative RAD2/SF2 helicase
3	cg18	gp081	gp077	gp267	hypothetical protein
	cg19	gp079	gp079	gp269*	hypothetical protein

	cg20	gp078	gp080	gp270	putative DNA-directed RNA polymerase beta prime subunit
	cg21	gp068	gp082	gp285	putative DNA polymerase
	cg22	gp067	gp084	gp286	putative virion structural protein
4	cg23	gp063	gp087	gp290	putative virion structural protein
	cg24	gp062	gp088	gp291	putative virion structural protein
	cg25	gp061	gp089	gp292	hypothetical protein
	cg26	gp060	gp090	gp293*	putative virion structural protein
	cg27	gp058	gp093, gp162, gp163	gp295, gp298*	virion structural protein/internal head
5	cg28	gp051	gp098	gp304	hypothetical protein
	cg29	gp050	gp099	gp305	putative virion structural protein
	cg30	gp049	gp100	gp306*	hypothetical protein
	cg31	gp048	gp101	gp307	putative virion structural protein
	cg32	gp008	gp188	gp311	putative thymidylate kinase
	cg33	gp043	gp118	gp315	putative DnaB helicase
	cg34	gp041	gp120	gp317	major capsid protein
	cg35	gp040	gp122	gp001*	hypothetical
	cg36	gp039	gp123	gp002	putative RNA polymerase beta subunit
	cg37	gp036	gp129	gp006	putative virion structural protein
	cg38	gp035	gp128	gp007	putative virion structural protein
	cg39	gp032	gp139	gp010	virion structural protein
	cg40	gp010	gp140	gp012	hypothetical
	cg41	gp030	gp130	gp016*	virion structural protein
	cg42	gp025, gp028, gp029	gp131, gp132, gp134, gp135	gp017, gp018, gp019	virion structural protein/tail fiber

	cg43	gp013	gp164	gp021	structural protein
	cg44	gp012	gp165	gp023	putative SbcC-like protein
6	cg45	gp249	gp161	gp144*	hypothetical
	cg46	gp248	gp157	gp145*	virion structural protein
	cg47	gp246	gp155	gp147	putative ribonuclease HI
	cg48	gp244	gp153	gp149*	hypothetical
	cg49	gp243	gp152	gp150	putative UvsX protein
	cg50	gp240	gp149	gp154	virion structural protein
	cg51	gp238	gp147	gp156*	hypothetical
	cg52	gp235	gp171	gp159*	hypothetical
	cg53	gp234	gp174	gp160	hypothetical
	cg54	gp233	gp182	gp161	virion structural protein
	cg55	gp232	gp181	gp162	putative lysozyme domain protein
	cg56	gp231	gp180	gp163	putative DNA-direct RNA polymerase beta subunit 2
	cg57	gp228	gp178	gp164	putative RNA polymerase beta subunit
	cg58	gp226	gp177	gp167*	hypothetical protein
	cg59	gp225	gp176	gp168	hypothetical protein
	cg60	gp223	gp175	gp170	putative virion structural protein
7	cg61	gp218	gp030	gp178	putative major virion structural protein
	cg62	gp217	gp029	gp179	putative tail sheath protein
	cg63	gp216	gp028	gp180	putative virion structural protein
	cg64	gp215	gp027	gp181	putative virion structural protein
	cg65	gp214	gp026	gp182*	putative structural protein
	cg66	gp213	gp025	gp183	putative terminase large subunit function
	cg67	gp211	gp032	gp187	hypothetical protein
	cg68	gp202	gp042	gp209	hypothetical protein

Table S4. GFP fusion proteins used in this study, Related to STAR Methods.

Bacterial strain	Plasmid backbone	Fusion protein	Known or putative function
E. amylovora ATCC 29780	pHERD30T	GFP-RAYgp222	Major nuclear shell protein (chimallin/ChmA)
E. amylovora ATCC 29780	pHERD30T	HNS-GFP	Histone-like nucleoid structuring protein (H-NS)
E. amylovora ATCC 29780	pHERD30T	GFP-RAYgp002	Non-virion RNA polymerase subunit β 1
E. amylovora ATCC 29780	pHERD30T	RAYgp248-GFP	Non-virion RNA polymerase subunit β 2
E. amylovora ATCC 29780	pHERD30T	GFP-RAYgp223	Non-virion RNA polymerase subunit β' 1
E. amylovora ATCC 29780	pHERD30T	RAYgp249-GFP	Non-virion RNA polymerase subunit β' 2
E. amylovora ATCC 29780	pHERD30T	RAYgp220-GFP	DNA polymerase
E. amylovora ATCC 29780	pHERD30T	RAYgp116-GFP	HslUV-like protease
E. amylovora ATCC 29780	pHERD30T	GFP-RAYgp150	UvsX/RecA
E. amylovora ATCC 29780	pHERD30T	RAYgp153-GFP	DNA processing (DprA)
E. amylovora ATCC 29780	pHERD30T	RAYgp250-GFP	Non-virion SF2 helicase
E. amylovora ATCC 29780	pHERD30T	RAYgp315-GFP	Replicative helicase (DnaB)
E. amylovora ATCC 29780	pHERD30T	RAYgp039-GFP	Stringent starvation (SspB)
E. amylovora ATCC 29780	pHERD30T	RAYgp049-GFP	tRNA ligase (RtcB)
E. amylovora ATCC 29780	pHERD30T	RAYgp064-GFP	exonuclease
E. amylovora ATCC 29780	pHERD30T	RAYgp311-GFP	Thymidylate kinase (TMK)
E. amylovora ATCC 29780	pHERD30T	RAYgp094-GFP	XRE family transcriptional regulator/repressor
E. amylovora ATCC 29780	pHERD30T	RAYgp317-GFP	Major capsid protein (MCP)
E. amylovora ATCC 29780	pHERD30T	RAYgp179-GFP	Tail sheath
E. amylovora ATCC 29780	pHERD30T	RAYgp154-GFP	Virion RNA polymerase subunit β 2
E. amylovora ATCC 29780	pHERD30T	GFP-RAYgp163	Virion RNA polymerase subunit β' 1
E. amylovora ATCC 29780	pHERD30T	RAYgp270-GFP	Virion RNA polymerase subunit β' 2
E. amylovora ATCC 29780	pHERD30T	GFP-RAYgp131	Virion SF2 helicase
E. amylovora ATCC 29780	pHERD30T	RAYgp299-GFP	Head protein
E. amylovora ATCC 29780	pHERD30T	GFP-RAYgp210	Phage tubulin (PhuZ)
E. amylovora ATCC 29780	pHERD30T	GFP-RAYgp210D198A	Catalytically dead phage tubulin (dPhuZ)

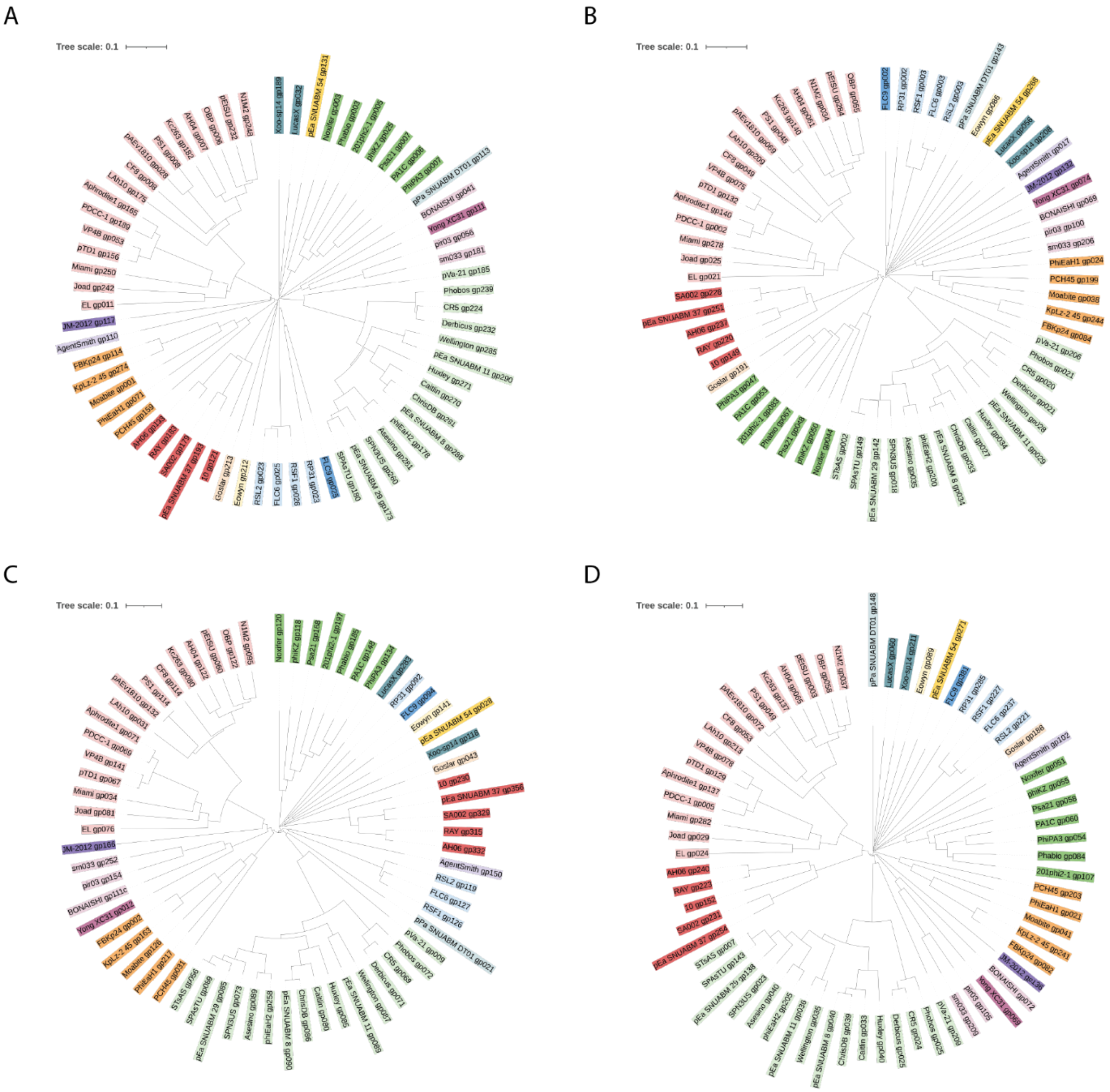

Figure S1. Phylogenetic trees including Chimalliviridae members based on well-conserved phage proteins, Related to Figure 1. (A) A protein tree of terminase large subunit homologs from Chimalliviridae. (B) A protein tree of DNA polymerase homologs from Chimalliviridae. (C) A protein tree of replicative (DnaB-like) helicases from Chimalliviridae. (D) A protein tree of non-virion RNA polymerase β′ subunit 1 homologs from Chimalliviridae. All homologs were found by the top PSI-BLAST hit. Chimalliviridae are color-coded by predicted genus as in Figure 1.

A

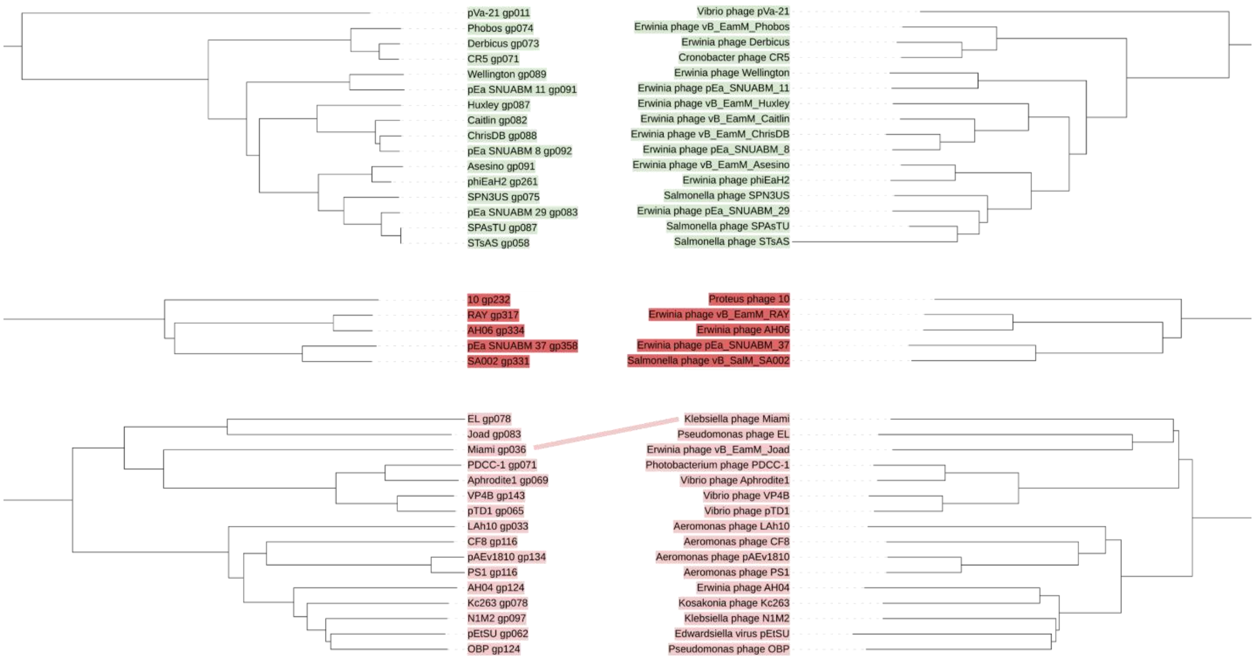

Major Capsid Protein vs. Whole Genome Tree

B

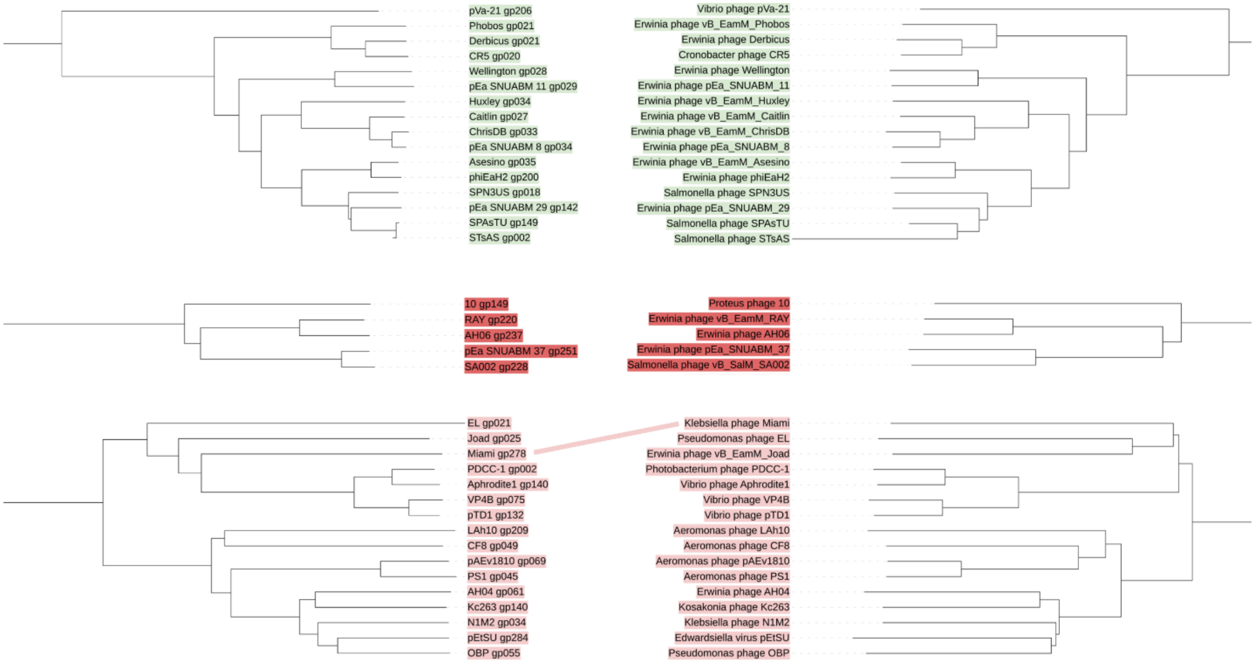

DNA Polymerase vs. Whole Genome Tree

C

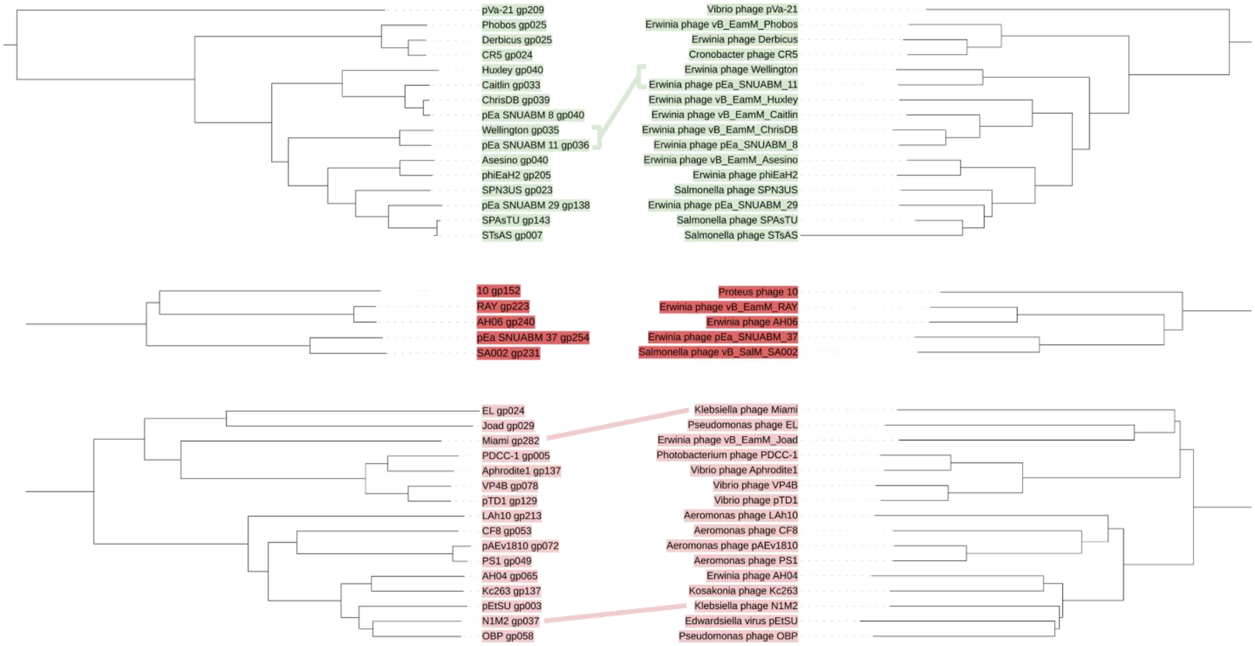

nvRNAP β' Subunit 1 vs. Whole Genome Tree

D

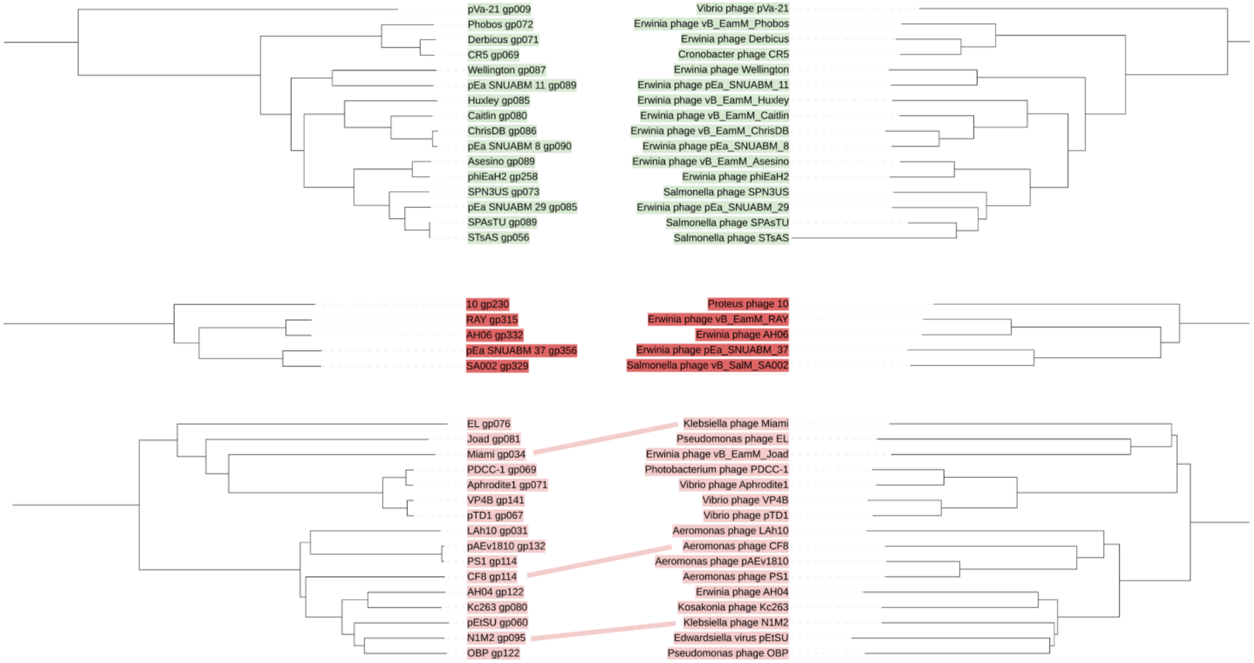

DnaB-like Helicase vs. Whole Genome Tree

E

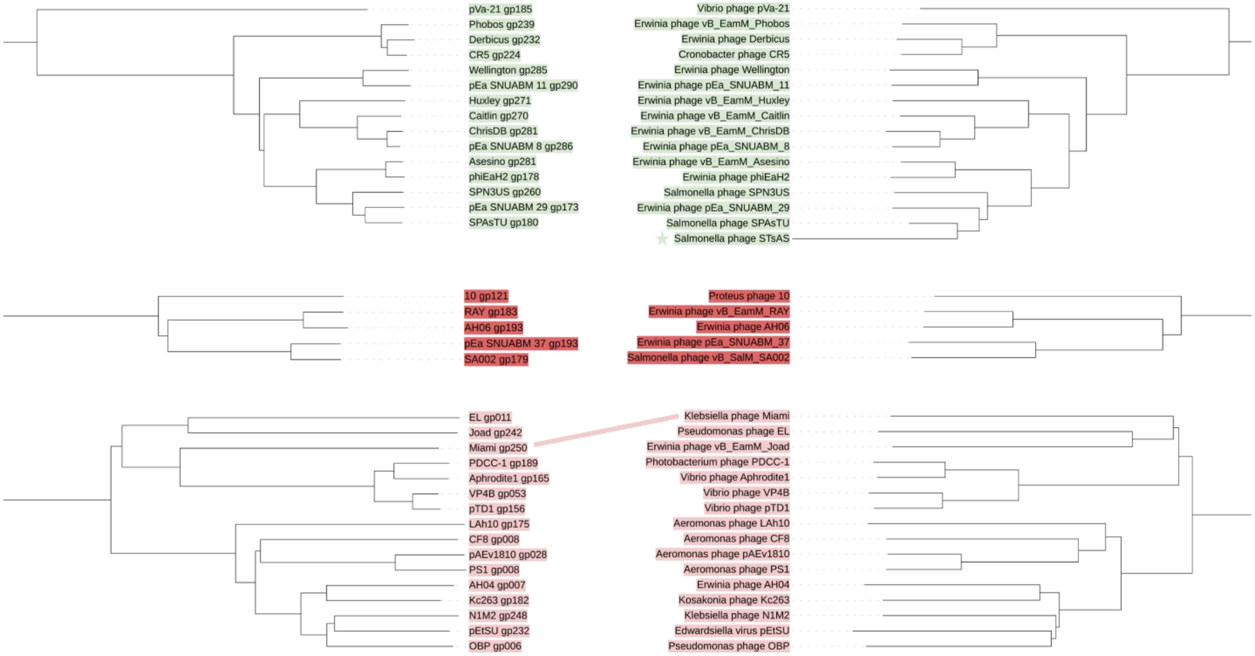

Terminase Large Subunit vs. Whole Genome Tree

Figure S2. Congruence between protein and whole genome based phylogenetics trees, Related to Figure 1. Mirrored trees comparing the whole genome phylogeny (right) with the phylogeny of individual proteins (left) were made for (A) the major capsid protein, (B) the DNA polymerase, (C) an RNA polymerase subunit, (D) the replicative helicase, and (E) the terminase large subunit. Minor discrepancies are pointed out with lines connecting the phage that have different branching patterns. In the case of the terminase, one phage was missing a homolog and is marked with a star. Clades are color-coded by predicted genus as in Figure 1. The light green, red, and light red clades were chosen for comparison because they were the largest clades and had the greatest potential to show how congruent the trees were (light green and light red) or were the clade containing RAY (red).

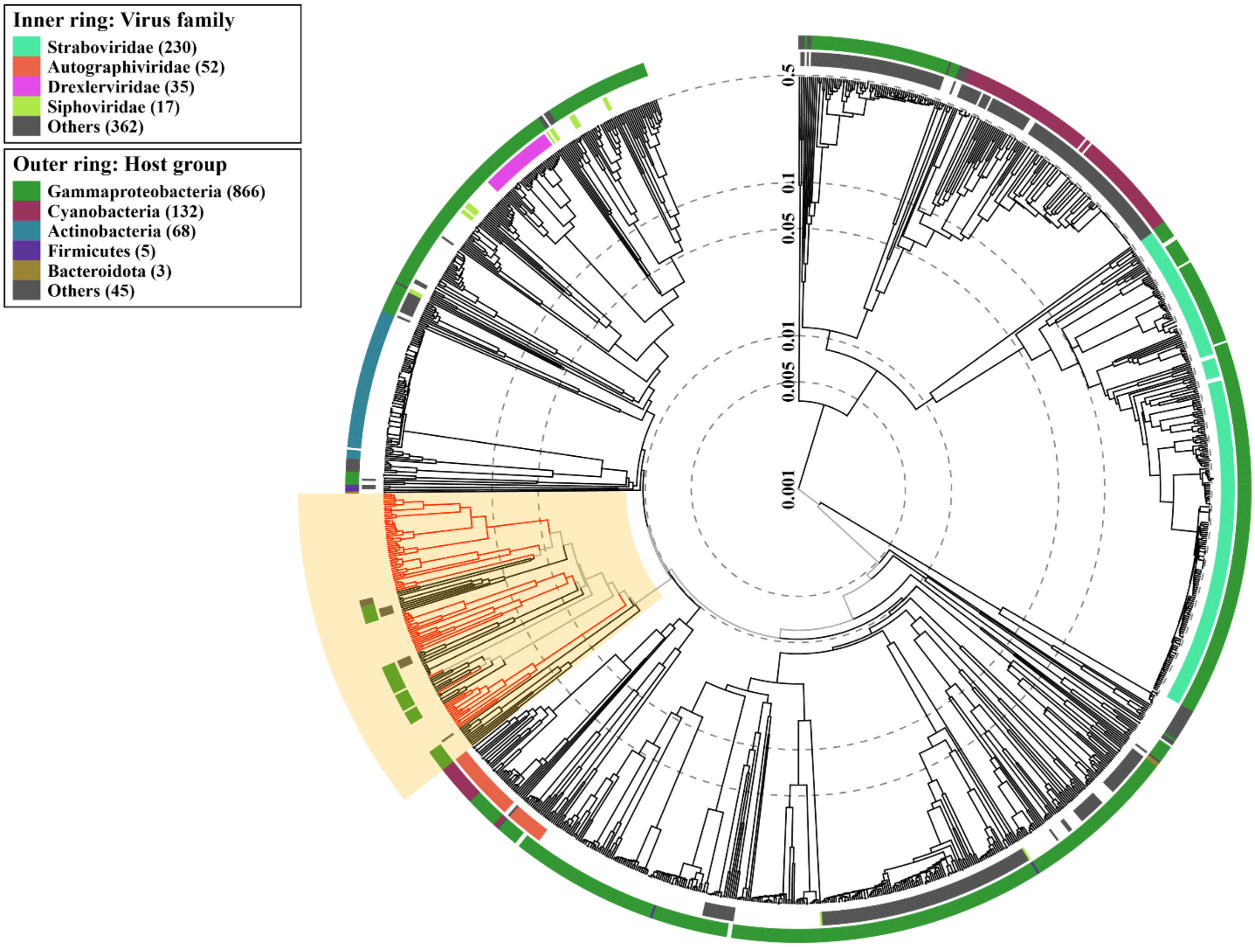

Figure S3. ViPTree data showing clustering of metagenomes, Related to Figure 1 and Supplemental File 1. Metagenome whole genome (complete or partial) sequences that encode chimallin homologs from the NCBI database (highlighted with red lines) cluster within the monophyletic clade of Chimalliviridae (highlighted in beige).

A

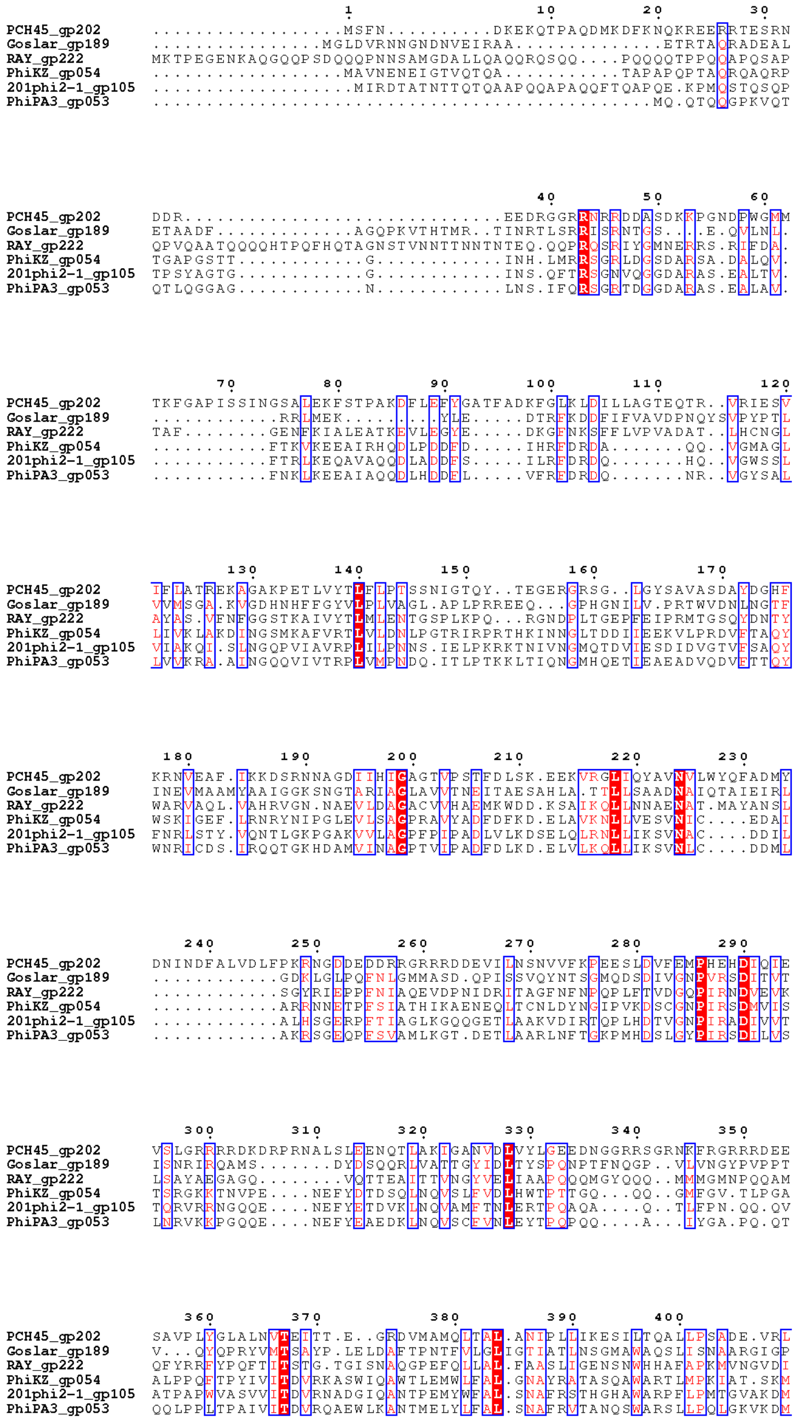
PCH45_gp202
Goslar_gp189
RAY_gp222
PhiKZ_gp054
201phi2-1_gp105
PhiPA3_gp053

```
                  410              420        430            440        450
PCH45_gp202       RDPRALALE......QPESFPADLIAEH....PSEEEWANLMEAVLHEDSTYIFLHAPRT
Goslar_gp189      HNPGALAMVLDPEVTAPLDLS...TQ......TNEQIYKFLQQVLYP.SLLISIDVPEE
RAY_gp222         NDIGAINYELKMGLESPDDRPKKIITKDHSF..TTQALHQLLYTACHE.SMSIAIDIEET
PhiKZ_gp054       RDIGALGYYSELGK........AIETRTEAFMADDSNFVMLMNKMVNQ.NPAFQIDIDPM
201phi2-1_gp105   KDIGALGWMSALRN........RIDTKAANF..DDAQFGQLMLSQVQP.NPVFQIDLNRM
PhiPA3_gp053      RDIGAIGYLSRLAA........RVETKTETF..TDQNFAELLYNMVRP.SPVFMSDLNRF

                  460        470                480        490        500           510
PCH45_gp202       GVHSQLLTYLVDACDEESD.......TSDDSYEIVTRVLNALTNNEYRQLGGED..MEFG
Goslar_gp189      GEYSWLLRMIPAAEKIYTGKVEGEVREISEGYKALYRAFDDVTLGCFSKKYQYG..LPLV
RAY_gp222         GTRTWVNSMLLLAGGQPNGNAAALSPQGQQAHKAIIQAANNLTNGEFSKHFTDQ.NQLIA
PhiKZ_gp054       GDNSAIEQVILDAAGGVNQ.........ARAVSLIFQALTNLYGTDFRQFYNLAEAGPII
201phi2-1_gp105   GETAQMDSLQLDAAGGPNA.........QKAAATIIRQINNLGGGGFERFFDHT.TQPIL
PhiPA3_gp053      GDNAAIENVFIDALGGVNQ.........QRAVAAIIAGVNNLIGGGFEKFFDHN.TMPII

                       520        530        540        550           560
PCH45_gp202       TLEIRQSFAGYWNDDRSGERRDLAPIGYFNMLTRFGQQHP....EYLDIYTRCFDNDSDT
Goslar_gp189      YATGNRIPLGHYNHQDG.HRHDIRDMDDLYMMNITN...PDTVEAWEDSFDRT...DMTM
RAY_gp222         VRDGTRIQGGFYVSKDNHQKMDIRNVDLLAVLNFVGETDPRIVEEWKIITSSTSGMSTP.
PhiKZ_gp054       FDNHHEVDLGHYTDEHG.ELSDRRDLDVLGAMNMSEGND....QEWKTWYATQVGND.HI
201phi2-1_gp105   ERTGQVIDLGNWFDGD..EKRDRRDLDNLAALNAAEGNE....NEFWGFYGAQLNPNLHP
PhiPA3_gp053      QPYGTDIQLGYYLDGEG.EKQDRRDLDVLGALNASDGNI....QEWMSWYGTQCNVAVHP

                     570        580        590        600        610        620
PCH45_gp202       DDINARNEELISAYTGKGYTIVDRNDVVRLNPAWLATIADALRDSGVSIDAEGIHTERGR
Goslar_gp189      SQRVVARHEIIDRVLSGSWEQTGWAMRYDFDPLALQALIEAAADAGFTIRPENIQHLAGT
RAY_gp222         .KRVALRQQKLQQLLGESFVLKAYYERVVINWAFMDALRKAITAAGLIVRPENTNMQYNV
PhiKZ_gp054       VRRMNNSKGFDKMYLGN.VTYTGRARRLTFNPKFITAMDAAAAAGVTVTMENLITNFGQ
201phi2-1_gp105   DLRNRQSRNYDRQYLGSTVTYTGKAERCTYNAKFIEALDRYLAEAGLQITMDNTSVLNSG
PhiPA3_gp053      ELRARQSKNFDRQYLGNSVTYTTRAHRGIWNPKFIEALDKAIASVGLTVAMDNVAQVFGA

                     630        640         650
PCH45_gp202       RRPMSNQ...YATGDMKS......GLFRRGRGRD...........:...RGGRGGRW..
Goslar_gp189      AVRGNMAARARGLGNISG.....NIYARSDRPNV...GVNNMGGAFNLF.........
RAY_gp222         QSYGTPMAQLYGMPTNIGSSLQQGAYATDNQGRVVNMAAFRTGGAFGTFNG........
PhiKZ_gp054       QRFAGYTG........MG......NMAVSGSAQV.G.MAGSMGQTYGAPSYNAPGTWY.
201phi2-1_gp105   QRFMGNSV........IG......NNMVSGQAQV.H.SAYAGTQGFNTQYQTGPSSFY.
PhiPA3_gp053      QRFSGNLA........IA......DYAVTGTAQV.S.SGLVSNGGYNPQFGVGQGSGFY
```

B

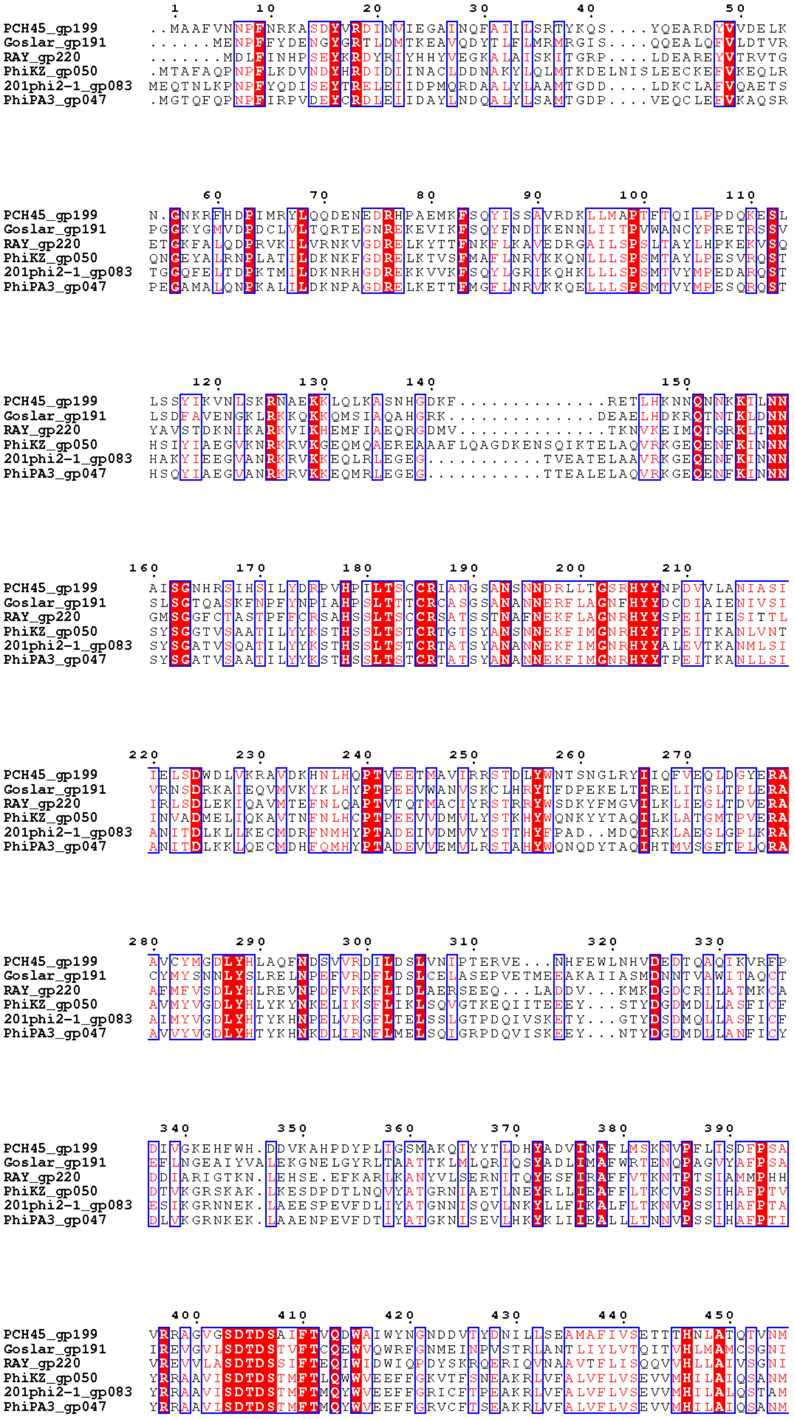
1 10 20 30 40 50
PCH45_gp199 ..MAAFVNNPFNRKASDYVRDINVIEGAINQFAIILSRTYKQS....YQEARDYVVDELK
Goslar_gp191 ......MENPFFYDENGYGRTLDMTKEAVQDYTLFLMRMRGIS....QQEALQFVLDTVR
RAY_gp220 .......MDLFINHPSEYKRDYRIYHHYVEGKALAISKITGRP....LDEAREYVTRVTG
PhiKZ_gp050 .MTAFAQPNPFLKDVNDYHRDIDIINACLDDNAKYLQLMTKDELNISLEECKEFVKEQLR
201phi2-1_gp083 MEQTNLKPNPFYQDISEYTRELEIIDPMQRDAALYLAAMTGDD....LDKCLAFVQAETS
PhiPA3_gp047 .MGTQFQPNPFIRPVDEYCRDLEIIDAYLNDQALYLSAMTGDP....VEQCLEFVKAQSR
60 70 80 90 100 110
PCH45_gp199 N.GNKRFHDPIMRYLQQDENEDRHPAEMKFSQYISSAVRDKLLMAPTFTQILPPDQKESL
Goslar_gp191 PGGKYGMVDPDCLVLTQRTEGNREKEVIKFSQYFNDIKENNLIITPVWANCYPRETRSSV
RAY_gp220 ETCKFALQDPRVKILVRNKVGDRELKYTTFNKFLKAVEDRGAILSPSLTAYLHPKEKVSQ
PhiKZ_gp050 QNGEYALRNPLATILDKNKFGDRELKTVSFMAFLNRVKKQNLLLSPSMTAYLPESVRQST
201phi2-1_gp083 TGGQFELTDPKTMILDKNRHGDREKKVVKFSQYLGRIKQHKLLLSPSMTVYMPEDARQST
PhiPA3_gp047 PEGAMALQNPKALILDKNPAGDRELKETTFMGFLNRVKKQELLLSPSMTVYMPESQRQST
120 130 140 150
PCH45_gp199 LSSYIKVNLSKRNAEKKLQLKASNHGDKF..............RETLHKNNQNNKKILNN
Goslar_gp191 LSDFAVENGKLRKKQKKQMSIAQAHGRK.............DEAELHDKRQTNTKKLDNN
RAY_gp220 YAVSTDKNIKARKVIKHEMFIAEQRGDMV..............TKNVKEIMQTGRKLTNN
PhiKZ_gp050 HSIYIAEGVKNRKRVKKGEQMQAEREAAAFLQAGDKENSQIKTELAQVRKGEQENFKINNN
201phi2-1_gp083 HAKYIEEGVANRKRVKKEQLRLEGEG...........TVEATELAAVRKGEQENFKINNN
PhiPA3_gp047 HSQYIAEGVANRKRVKKEQMRLEGEG...........TTEALELAQVRKGEQENFKINNN
160 170 180 190 200 210
PCH45_gp199 AISCNHRSIHSILYDRPVHPILTSCCRIANGSANSNNDRLLTGSRHYYNPDVVLANIASI
Goslar_gp191 SLSGTQASKFNPFYNPIAHPSLTTTCRCASGSANANNERFLAGNFHYYDCDIAIENIVSI
RAY_gp220 GMSGGFCTASTPFFCRSAHSSLTSCCRSATSSTNAFNEKFLAGNRHYYSPEITIESITTL
PhiKZ_gp050 SYSGGTVSAATILYYKSTHSSLTSTCRTGTSYANSNNEKFIMGNRHYYTPEITKANLVNT
201phi2-1_gp083 SYSGATVSQATILYYKSTHSSLTSTCRTATSYANANNEKFIMGNRHYYALEVTKANMLSI
PhiPA3_gp047 SYSGATVSAATILYYKSTHSSLTSTCRTATSYANANNEKFIMGNRHYYTPEITKANLLSI
220 230 240 250 260 270
PCH45_gp199 IELSDWDLVKRAVDKHNLHQPTVEETMAVTRRSTDLYWNTSNGLRYIIQFVEQLDGYERA
Goslar_gp191 VRNSDRKAIEQVMVKYKLHYPTPEEVWANVSKCLHRYTFDPEKELTIRELITGLTPLERA
RAY_gp220 IRLSDLEKIQAVMTEFNLQAPTVTQTMACIYRSTRRYWSDKYFMGVILKLIEGLTDVERA
PhiKZ_gp050 INVADMELIQKAVTNFNLHCPTPEEVVDMVLYSTKHYWQNKYYTAQILKLATGMTPVERA
201phi2-1_gp083 ANITDLKLLKECMDRFNMHYPTADEIVDMVVYSTTHYFPAD..MDQIRKLAEGLGPLKRA
PhiPA3_gp047 ANITDLKKLQECMDHFQMHYPTADEVVEMVLRSTAHYWQNQDYTAQIHTMVSGFTPLQRA
280 290 300 310 320 330
PCH45_gp199 AVCYMGDLYHLAQFNDSVVRDILDSLVNIPTERVE...NHFEWLNHVDEDTQAQIKVRFP
Goslar_gp191 CYMYSNNLYSLRELNPEFVRDFLDSLCELASEPVETMEEAKAIIASMDNNTVAWITAQCT
RAY_gp220 AFMFVSDLYHLREVNPDFVRKFLIDLAERSEEQ...LADDV...KMKDGDCRILATMKCA
PhiKZ_gp050 AVMYVGDLYHLYKYNKELIKSFLIKLSQVGTKEQIITEEEY...STYDGDMDLLASFICF
201phi2-1_gp083 AIMYVGDLYHTYKHNPELVRGFLTELSSLGTPDQIVSKETY...GTYDSDMQLLASFICF
PhiPA3_gp047 AVVYVGDLYHTYKHNKDLIRNELMELSQIGRPDQVISKEEY...NTYDGDMDLLANFICY
340 350 360 370 380 390
PCH45_gp199 DIVGKEHFWH.DDVKAHPDYPLIGSMAKQIYYTLDHYADVINAFLMSKNVPFLTSDFPSA
Goslar_gp191 EFLNGEAIYVALEKGNELGYRLTATTKLMLQRIQSYADLIMAFWRTENQPAGVYAFPSA
RAY_gp220 DDIARIGTKN.LEHSE.EFKARLKANYVLSERNITQYESFIRAFFVTKNTPTSIAMMPHH
PhiKZ_gp050 DTVKGRSKAK.LKESDPDTLNQVYATGRNIAETLNEYRLLIEAFFLTKCVPSSIHAFPTV
201phi2-1_gp083 ESIKGRNNEK.LAEESPEVFDLIYATGNNISQVLNKYLLFIKALFLTKNVPSSIHAFPTA
PhiPA3_gp047 DLVKGRNKEK.LAAENPEVFDTIYATGKNISEVLHKYKLIIEALLLTNNVPSSIHAFPTI
400 410 420 430 440 450
PCH45_gp199 VRRAGVGSDTDSAIFTVQDWAIWYNGNDDVTYDNILLSEAMAFIVSETTTHNLATQTVNM
Goslar_gp191 IREVGVISDTDSTVFTCQEWVQWRFGNMEINPVSTRLANTLIYLVTQITVHLMAMCSGNI
RAY_gp220 VREVVLASDTDSSIFTEQIWIDWIQPDYSKRQERIQVNAAVTFLISQQVVHLLAIVSGNI
PhiKZ_gp050 YRRAAVISDTDSTMFTLQWWVEEFFGKVTFSNEAKRLVFALVFLVSEVVMHILAIQSANM
201phi2-1_gp083 YRRAAVISDTDSTMFTMQYWVEEFFGRICFTPEAKRLVFALVFLVSEVVMHILALQSTAM
PhiPA3_gp047 YRRAAVISDTDSTMFTMQYWVEEFFGRVCFTSEAKRLVFALVFLVSEVVMHILAIQSANM

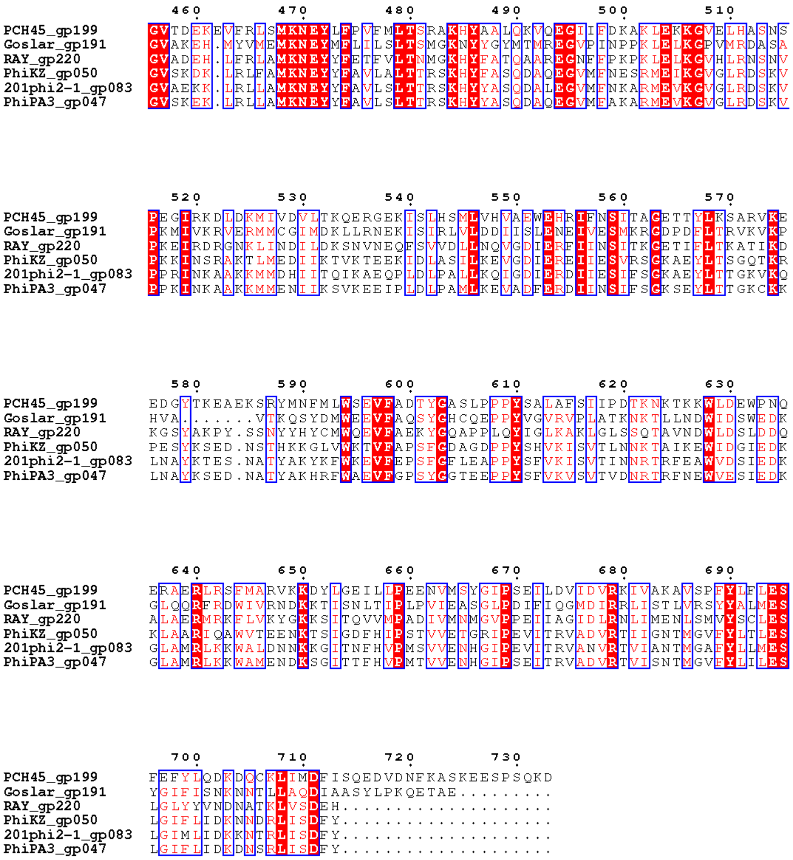

460 470 480 490 500 510
PCH45_gp199 GVTDEKEVFRLSMKNEYLFPVFMLTSRAKHYAALQKVQEGIIFDKAKLEKKGVELHASNS
Goslar_gp191 GVAKEH.MYVMEMKNEYMFLILSLTSMGKNYYGYMTMREGVPINPPKLELKGPVMRDASA
RAY_gp220 GVADEH.LFRLAMKNEYYFETFVLTNMGKHYFATQAAREGNFFPKPKLELKGVHLRNSNV
PhiKZ_gp050 GVSKDK.LRLFAMKNEYYFAVLALTTRSKHYFASQDAQEGVMFNESRMEIKGVGLRDSKV
201phi2-1_gp083 GVAEKK.LRLLAMKNEYYFAVLSLTTRSKHYYASQDALEGVMFNKARMEVKGVGLRDSKV
PhiPA3_gp047 GVSKEK.LRLLAMKNEYYFAVLSLTTRSKHYYASQDAQEGVMFAKARMEVKGVGLRDSKV
520 530 540 550 560 570
PCH45_gp199 PEGIRKDLDKMIVDVLTKQERGEKISLHSMLVHVAEWEHRIFNSITAGETTYLKSARVKE
Goslar_gp191 PKMIVKRVERMMCGIMDKLLRNEKISIRLVLDDIISLENEIVESMKRGDPDFLTRVKVKP
RAY_gp220 PKEIRDRGNKLINDILDKSNVNEQFSVVDLLNQVGDIERFIINSITKGETIFLTKATIKD
PhiKZ_gp050 PKKINSRAKTLMEDIIKTVKTEEKIDLASILKEVGDIEREIIESVRSGKAEYLTSGQTKR
201phi2-1_gp083 PPRINKAAKKMMDHIITQIKAEQPLDLPALLKQIGDIERDIIESIFSGKAEYLTTGKVKQ
PhiPA3_gp047 PPKINKAAKKMMENIIKSVKEEIPLDLPAMLKEVADFERDIINSIFSGKSEYLTTGKCKK
580 590 600 610 620 630
PCH45_gp199 EDGYTKEAEKSRYMNFMLWSEVFADTYGASLPPPYSALAFSIIPDTKNKTKKWLDEWPNQ
Goslar_gp191 HVA.......VTKQSYDMWEEVFAQSYGHCQEPPYVGVRVPLATKNKTLLNDWIDSWEDK
RAY_gp220 KGSYAKPY.SSNYYHYCMWQEVFAEKYGQAPPLQYIGLKAKLGLSSQTAVNDWLDSLDDQ
PhiKZ_gp050 PESYKSED.NSTHKKGLVWKTVFAPSFGDAGDPPYSHVKISVTLNNKTAIKEWIDGIEDK
201phi2-1_gp083 LNAYKTES.NATYAKYKFWKEVFEPSFGFLEAPPYSFVKISVTINNRTRFEAWVDSIEDK
PhiPA3_gp047 LNAYKSED.NATYAKHRFWAEVFGPSYGGTEEPPYSFVKVSVTVDNRTRFNEWVESIEDK
640 650 660 670 680 690
PCH45_gp199 ERAERLRSFMARVKKDYLGEILLPEENVMSYGIPSEILDVIDVRKIVAKAVSPFYLFLES
Goslar_gp191 GLQQRFRDWIVRNDKKTISNLTIPLPVIEASGLPDIFIQGMDIRRLISTLVRSYYALMES
RAY_gp220 ALAERMRKFLVKYGKKSITQVVMPADIVMNMGVPPEIIAGIDLRNLIMENLSMVYSCLES
PhiKZ_gp050 KLAARIQAWVTEENKTSIGDFHIPSTVVETGRIPEVITRVADVRTIIGNTMGVFYLTLES
201phi2-1_gp083 GLAMRLKKWALDNNKKGITNFHVPMSVVENHGIPEVITRVANVRTVIANTMGAFYLLMES
PhiPA3_gp047 GLAMRLKKWAMENDKSGITTFHVPMTVVENHGIPSEITRVADVRTVISNTMGVFYLILES
700 710 720 730
PCH45_gp199 FEFYLQDKDQCKLIMDFISQEDVDNFKASKEESPSQKD
Goslar_gp191 YGIFISNKNNTLLAQDIAASYLPKQETAE.........
RAY_gp220 LGLYYVNDNATKLVSDEH....................
PhiKZ_gp050 LGIFLIDKNNDRLISDFY....................
201phi2-1_gp083 LGIMLIDKKNTRLISDFY....................
PhiPA3_gp047 LGIFLIDKDNSRLISDFY....................

C

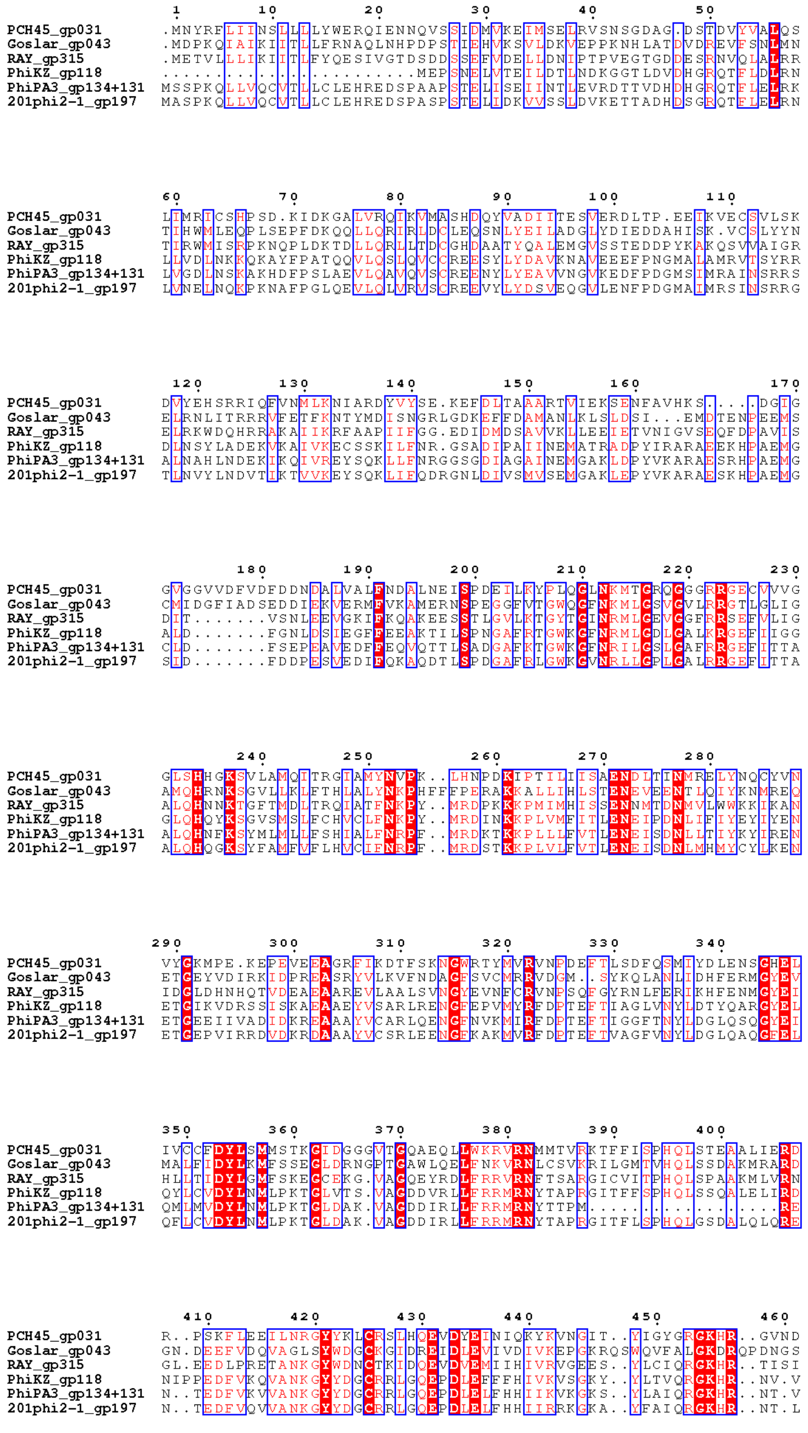
1 10 20 30 40 50
PCH45_gp031 .MNYRFLIINSLLLLYWERQIENNQVSSIDMVKEIMSELRVSNSGDAG.DSTDVYVALQS
Goslar_gp043 .MDPKQIAIKIITLLFRNAQLNHPDPSTIEHVKSVLDKVEPPKNHLATDVDREVFSNLMN
RAY_gp315 .METVLLLIKIITLFYQESIVGTDSDDSSEFVDELLDNIPTPVEGTGDDESRNVQLALRR
PhiKZ_gp118 ........................MEPSNELVTEILDTLNDKGGTLDVDHGRQTFLDLRN
PhiPA3_gp134+131 MSSPKQLLVQCVTLLCLEHREDSPAAPSTELISEIINTLEVRDTTVDHDHGRQTFLELRK
201phi2-1_gp197 MASPKQLLVQCVTLLCLEHREDSPASPSTELIDKVVSSLDVKETTADHDSGRQTFLELRN
60 70 80 90 100 110
PCH45_gp031 LTMRICSHPSD.KIDKGALVRQIKVMASHDQYVADIITESVERDLTP.EEIKVECSVLSK
Goslar_gp043 TIHWMLEQPLSEPFDKQQLLQRIRLDCLEQSNLYEILADGLYDIEDDAHISK.VCSLYYN
RAY_gp315 TIRWMISRPKNQPLDKTDLLQRLLTDCGHDAATYQALEMGVSSTEDDPYKAKQSVVAIGR
PhiKZ_gp118 LLVDLNKKQKAYFPATQQVLQSLQVCCREESYLYDAVKNAVEEEFPNGMALAMRVTSYRR
PhiPA3_gp134+131 LVGDLNSKAKHDFPSLAEVLQAVQVSCREENYLYEAVVNGVKEDFPDGMSIMRAINSRRS
201phi2-1_gp197 LVNELNQKPKNAFPGLQEVLQLVRVSCREEVYLYDSVEQGVLENFPDGMAIMRSINSRRG
120 130 140 150 160 170
PCH45_gp031 DVYEHSRRIQFVNMLKNIARDYVYSE.KEFDITAAARTVIEKSENFAVHKS.....DGIG
Goslar_gp043 ELRNLITRRRVFETFKNTYMDISNGRLGDKEFFDAMANLKLSLDSI...EMDTENPEEMS
RAY_gp315 ELRKWDQHRRAKAIIKRFAAPIIFGG.EDIDMDSAVVKLLEEIETVNIGVSEQFDPAVIS
PhiKZ_gp118 DLNSYLADEKVKAIVKECSSKILFNR.GSADIPAIINEMATRADPYIRARAEEKHPAEMG
PhiPA3_gp134+131 ALNAHLNDEKIKQIVREYSQKLLFNRGGSGDIAGAINEMGAKLDPYVKARAESRHPAEMG
201phi2-1_gp197 TLNVYLNDVTLKTVVKEYSQKLIFQDRGNLDIVSMVSEMGAKLEPYVKARAESKHPAEMG
180 190 200 210 220 230
PCH45_gp031 GVGGVVDFVDFDDNDALVALFNDALNEISPDEILKYPLQGLNKMTGRQGGGRRGECVVVG
Goslar_gp043 CMIDGFIADSEDDIEKVERMFVKAMERNSPEGGFVTGWQGFNKMLGSVGVLRRGTLGLIG
RAY_gp315 DIT.......VSNLEEVGKIFKQAKEESSTLGVLKTGYTGINRMLGEVGGFRRSEFVLIG
PhiKZ_gp118 ALD.......FGNLDSIEGFFEEAKTILSPNGAFRTGWKGFNRMLGDLGALKRGEFIIGG
PhiPA3_gp134+131 CLD.......FSEPEAVEDFFEQVQTTLSADGAFKTGWKGFNRIIGSLGAFRRGEFITTA
201phi2-1_gp197 SID.......FDDPESVEDIFQKAQDTLSPDGAFRLGWKGVNRLLGPLGALRRGEFITTA
240 250 260 270 280
PCH45_gp031 GLSHHGKSVLAMQITRGIAMYNVPK..LHNPDKIPTILIISAENDLTINMRELYNQCYVN
Goslar_gp043 AMQHRNKSGVLLKLFTHLALYNKPHFFFPERAKKALLIHLSTENEVEENTLQIYKNMREQ
RAY_gp315 ALQHNNKTGFTMDLTRQIATFNKPY..MRDPKKKPMIMHISSENNMTDNMVLWWKKIKAN
PhiKZ_gp118 GLQHQYKSGVSMSLFCHVCLFNKPY..MRDINKKPLVMFITLENEIPDNLIFIYEYIYEN
PhiPA3_gp134+131 ALQHNFKSYMLMLLFSHIALFNRPF..MRDKTKKPLLLFVTLENEISDNLLTIYKYIREN
201phi2-1_gp197 ALQHQGKSYFAMFVFLHVCIFNRPF..MRDSTKKPLVLFVTLENEISDNLMHMYCYLKEN
290 300 310 320 330 340
PCH45_gp031 VYGKMPE.KEPEVEEAGRFIKDTFSKNGWRTYMVRVNPDEFTLSDFQSMIYDLENSGHEL
Goslar_gp043 ETCEYVDIRKIDPREASRYVLKVFNDAGFSVCMRRVDGM..SYKQLANLIDHFERMGYEV
RAY_gp315 IDGLDHNHQTVDEAEAAREVLAALSVNGYEVNFCRVNPSQFGYRNLFERIKHFENMGYEI
PhiKZ_gp118 ETGIKVDRSSISKAEAAEYVSARLRENGFEPVMYRFDPTEFTIAGLVNYLDTYQARGYEL
PhiPA3_gp134+131 ETGEEIIVADIDKREAAAYVCARLQENGFNVKMIRFDPTEFTIGGFTNYLDGLQSQGYEI
201phi2-1_gp197 ETGEPVIRRDVDKRDAAAYVCSRLEENGFKAKMVRFDPTEFTVAGFVNYLDGLQAQGFEL
350 360 370 380 390 400
PCH45_gp031 IVCCFDYLSMMSTKGIDGGGVTGQAEQLLWKRVRNMMTVRKTFFISPHQLSTEAALIERD
Goslar_gp043 MALFIDYLKMFSSEGLDRNGPTGAWLQELFNKVRNLCSVKRILGMTVHQLSSDAKMRARD
RAY_gp315 HLLTIDYLGMFSKEGCEKG.VACQEYRDLFRRVRNFTSARGICVITPHQLSPAAKMLVRN
PhiKZ_gp118 QYLCVDYLNMLPKTGLVTS.VACDDVRLLFRRMRNYTAPRGITFFSPHQLSSQALELIRD
PhiPA3_gp134+131 QMLMVDYLNMLPKTGLDAK.VAGDDIRLLFRRMRNYTTPM..................RE
201phi2-1_gp197 QFLCVDYLNMLPKTGLDAK.VACDDIRLLFRRMRNYTAPRGITFLSPHQLGSDALQLQRE
410 420 430 440 450 460
PCH45_gp031 R..PSKFLEEILNRGYYKLCRSIHQEVDYEINIQKYKVNGIT..YIGYGRGKHR..GVND
Goslar_gp043 GN.DEEFVDQVAGLSYWDGCKGIDREIDLEVIVDIVKEPGKRQSWQVFALCKDRQPDNGS
RAY_gp315 GL.EEDLPRETANKGYWDNCTKIDQEVDVEMIIHIVRVGEES..YLCIQRGKHR..TISI
PhiKZ_gp118 NIPPEDFVKQVANKGYYDGCRRLGQEPDLEFFFHIVKVSGKY..YLTVQRGKHR..NV.V
PhiPA3_gp134+131 N..TEDFVKVVANKGYYDGCRRLGQEPDLELFHHIIKVKGKS..YLAIQRGKHR..NT.V
201phi2-1_gp197 N..TEDFVQVVANKGYYDGCRRLGQEPDLELFHHIIRRKGKA..YFAIQRGKHR..NT.L

```
                          470          480       490          500       510
PCH45_gp031        TPEQDKICWYQIGE..LGI.LDDINEPESRAMDKPGR.PVL..ADDDFWASTPTETYAMN
Goslar_gp043       TKPEDKHFAMPFQT..IGMLPDDYGKKAVYCRKVGGQ.PVS.EGGKGPWNKFDENAAPSN
RAY_gp315          TPERDKYCVYKFEPGV.GI.LDDVN.GKDKSRKHVAGETNSDG.GGAPWFG.........
PhiKZ_gp118        SDSKHHYFVMPFDPDRIGGIRWDIDKEENNYMDFVPSLSNMQATGGDDWGY.........
PhiPA3_gp134+131   TSEEDQYVLLPFSS..CGIIPWDIDKEEDYSLKIVPG.SIIGGMDDDAWSN.........
201phi2-1_gp197    TPEEDQYVIIPMSP..VGTMPWDIDKEEDYCIRVIPG.NNIGSDGDDAWD..........


PCH45_gp031        ........
Goslar_gp043       ELQFDLEF
RAY_gp315          ........
PhiKZ_gp118        ........
PhiPA3_gp134+131   ........
201phi2-1_gp197    ........
```

D

i

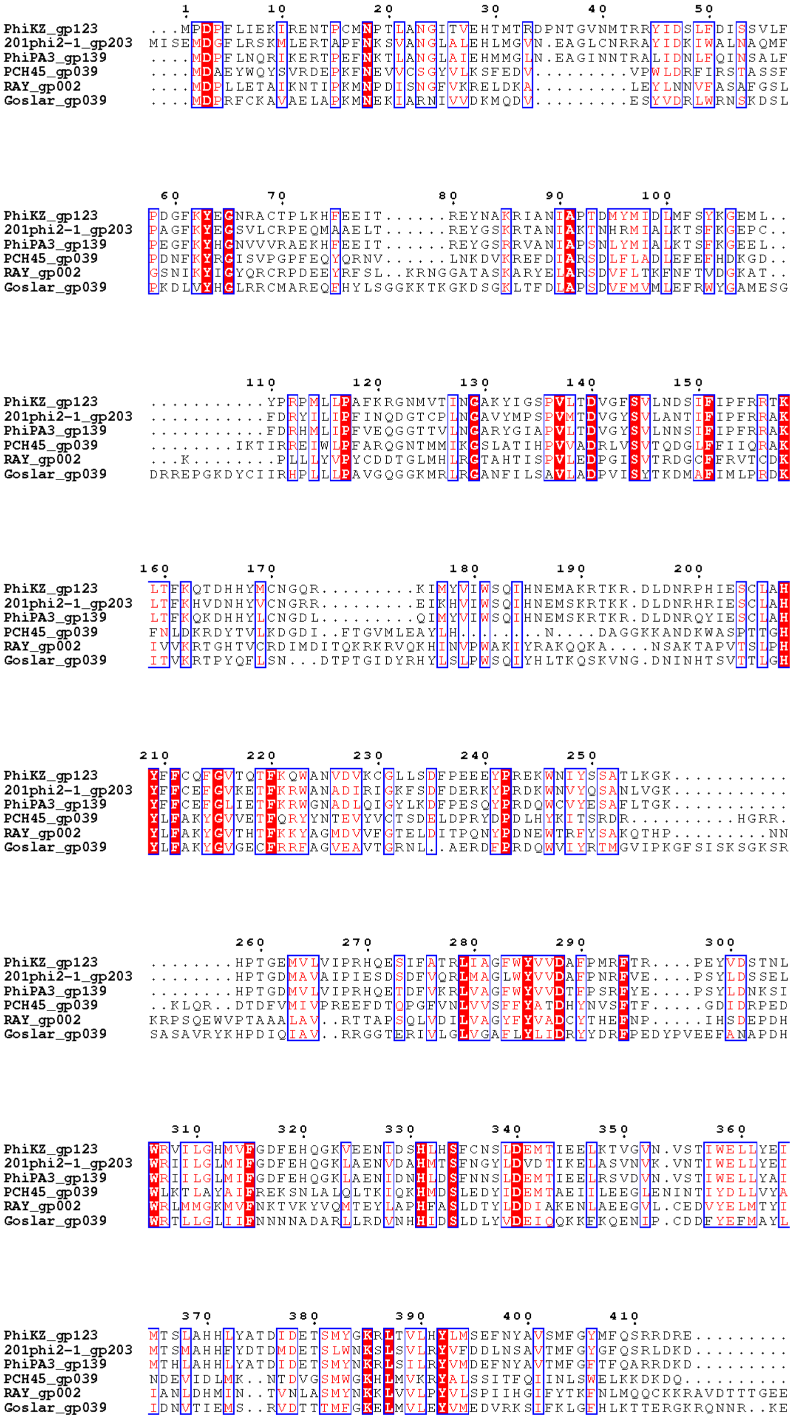
PhiKZ_gp123
201phi2-1_gp203
PhiPA3_gp139
PCH45_gp039
RAY_gp002
Goslar_gp039

```
                     420       430           440        450       460
PhiKZ_gp123      ...WTVQELNEGLKRSFKLQTAI...KRLTVDHGELDTMSNPNSSMLIKGTSILVTQDR.
201phi2-1_gp203  ...WTINEINDALKRSFKPNTAV...RRLSVDHGEFDTVSYPGDNKAIKLTSIVVPQDK.
PhiPA3_gp139     ...WTAQEINDALKRSFKLNTCI...RKLTSEHGEMETISMPGDNKAIKITSILVPQDR.
PCH45_gp039      ...LTYKKVKWLLGRYLHPNSFLGITKN....HGEKTNVQYPGDNMLFKHTLISVRQIDA
RAY_gp002        IMVFTEDTLFDVLGRNLKPEAIN...KVKGPDHGMISSVAAPGDNKMFKINNKITLQQNA
Goslar_gp039     ...MTNRDVQKALRNQVATEAILNIQSNRDKREKVATSIQSPGDCMLFKVSTHVVLQSQN

                 470       480       490       500       510       520
PhiKZ_gp123      AKTAKAHNKSLINDSSRIIHASIAEVGQYKNQPKNNPDGRGRLNMYTKVGPTGLVERREE
201phi2-1_gp203  AKSKGSHNKSLIGDSSRLIHVSLADVGQYKNQPKNNPDGRGRLNLYVDVGPDGTIQRGKD
PhiPA3_gp139     AKTSKAHNKSLIGDSTRLIHASIAEVGQYKNQPKNNPDGRGRLNLFVGVRYDGMIERRDD
PCH45_gp039      VMTANGKSKINVDDPGYHLHPSIFDSGSMVNFPKPNPDGRTKLNPFITTSESGKILPNPK
RAY_gp002        TQSGGRRNESPMTDDSKGLDVSIADCASYLHITKPDPTGRSLFNPFKLLVRDK.LLPSVK
Goslar_gp039     AGG......AALADESRLVSGSIPFCASYLNLPHSEPTGRQKFSMYAPIDERGRLIITPQ

                 530       540
PhiKZ_gp123      VREIIDNAQLMFRAK..................
201phi2-1_gp203  DREFLDSVQARFNR...................
PhiPA3_gp139     DRELLDATQKRFAR...................
PCH45_gp039      YYRMLKDEVG...SEIG.FDN............
RAY_gp002        YEKLFSVTQA..............IIYRRNI..
Goslar_gp039     IANDVAATNAGLKKDVGRFDEEIVEINDRDIID
```

ii

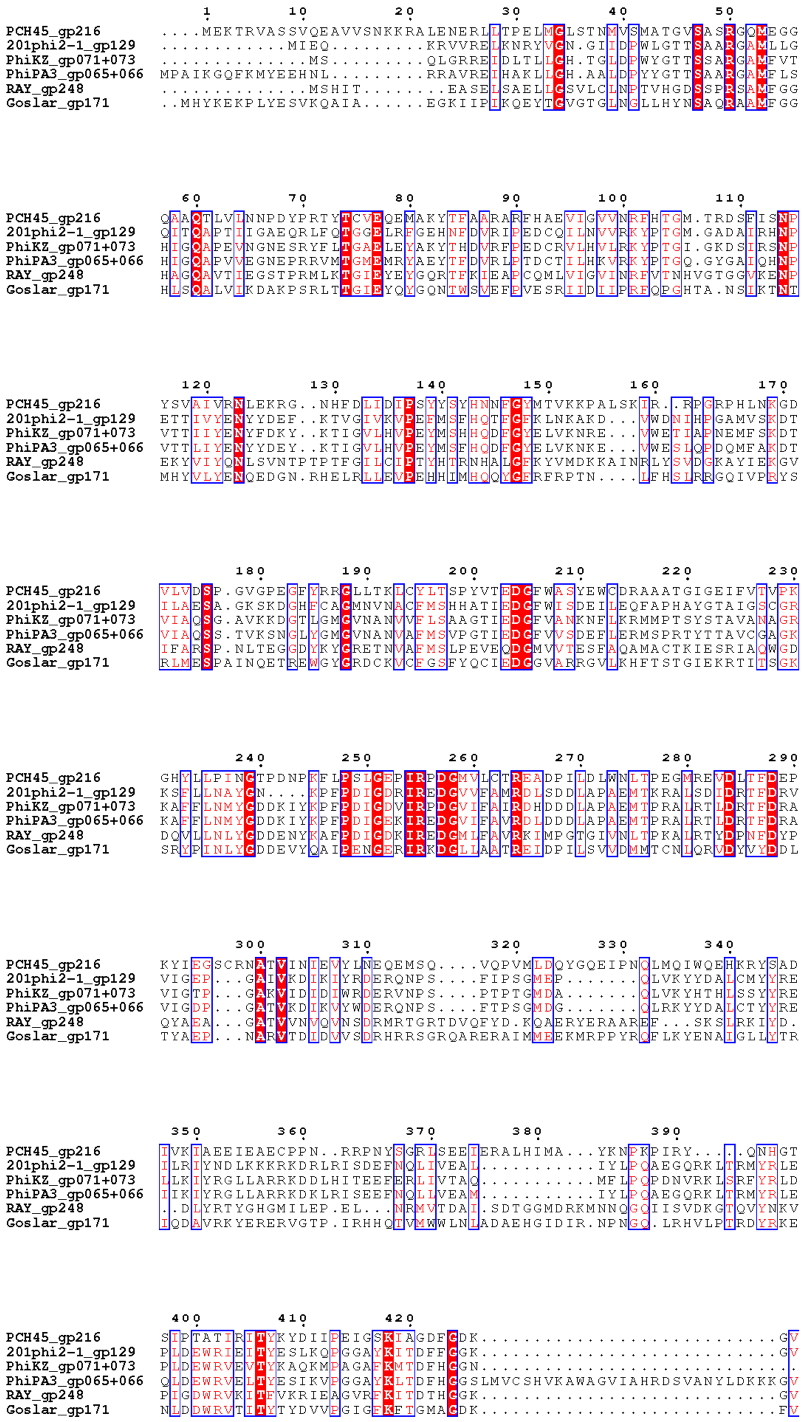
PCH45_gp216
201phi2-1_gp129
PhiKZ_gp071+073
PhiPA3_gp065+066
RAY_gp248
Goslar_gp171

```
                   430       440       450       460       470       480
PCH45_gp216        LCRKTPGSHMPLDMYGTQAELVSHANATINRMISVRLDDNYLGAQCILREKEI.......
201phi2-1_gp129    VCKTSPRSEMPRDEFGNIADVVIFGGSTMRRSNYGRIYEHGFGATVRDLCQRLRVEAGFD
PhiKZ_gp071+073    .........MPIDENGNRADLIIFGGSTMRRSNYGRIYEHGFGAAARDLAQRLRVEAGLD
PhiPA3_gp065+066   VCEVRPKADMPVDEFGNVVDAIIFGGSTMRRSNYGRIYEHGFGAASRDLAQRLRVEAGLP
RAY_gp248          IVNVIPDADAPTDDYGNRADVIMDDVSITKRMNLGKPTEQYINGASVYAAKIA.......
Goslar_gp171       VTDIMEDDEMPVDQWGNVADFMFDDSTVIKRMSLSRVNAPYINGVGDQIMREL.......

                                490       500       510       520
PCH45_gp216        .............RELYNAGRWEDAFSIISRFYEVATPRAFKEKF.IPYMTTPERRKRHV
201phi2-1_gp129    RHAELNNIDFAQSKAFNDPAWIEYAFNEFQELWWIIAPTMHEIMK......EHPNHKEYV
PhiKZ_gp071+073    RHAKPTQQQLN..SVMGNTQWVDYAFKELLGFYEIIAPTMHSKMM......EHPNPAEHV
PhiPA3_gp065+066   RHGVVPEQDLN..RVCSNREWVTYAFAELQEFYYIIAPTMHEILR......EHPSPAEYV
RAY_gp248          .............RQMAASGDLDGAWNHLMSYYHAAAYEQWEMMQSPTYLDNKPNRDHHV
Goslar_gp171       .............KPVMDAGDLETAWNTLMRYYYIVSPEFVEQVV.EKYCITDEDKWDHL

                   530       540        550        560       570       580
PCH45_gp216        ESVVNGHIMLELKHNDFPNH.IYLTEAL..EAEWPYEKGPVLFTDTRGVRRKTKVPVMIG
201phi2-1_gp129    KSALRQGTTVVYTPIDDTTHLPTAMNTIINT.KFRPNYTPVTYTDPGGRVVTTKNNVLIG
PhiKZ_gp071+073    KTVLMDGFPYIYAPVDDPVDLMAAVNKLINSDKYRPHYGKVSYRDQAGKWVTTKDNVLMG
PhiPA3_gp065+066   KTVLRDGFSYIYSPVDDPVDLMSSLNCIMNS.RFCPNHTRVTYRGQDGKMVTTKDKVLVG
RAY_gp248          RWVCDHGIE.LFAPTDRRYFGAEQVRRIMKE..HDFPVTPVTYRAPDGRMVRTRDPVVIA
Goslar_gp171       RYVQRHGTE.VYIPTDSIAAGSERMENLIRE..FPLKKGPVRYRGRSGQWRTTKNDIAIG

                      590       600       610       620       630       640
PCH45_gp216        TSYIRVLEKTGHDWGAVDSPSRQAHGIAAKISHRDRHARPYRKQPYRY.GEAEIRPLIAL
201phi2-1_gp129    PLYMMLLEKIGDDWSSVASVKVQQFGLPSKLNNFDRSSTPGRESAVRSFGESETRSYNCT
PhiKZ_gp071+073    PLYMMLLEKIGEDWSAAASVKTQPFGLPSKLNNADRASTPGRETAIRSFGESETRSYNCT
PhiPA3_gp065+066   PLYMMLLEKIGEDWSAVASVKVQQFGLPSKLNNSDRSSTPGRESAIRSFGESETRSYNCT
RAY_gp248          PIYIILLEKMGEYWASCAIPKLTHFGTLSSLTQADKFALPWRNTPTRF.GESELRLFLAA
Goslar_gp171       DTYIMMLEKTANDWSAVGIPKLQAHGLPSKLSNSDKYSSPGKEQPTRYCGESEHREVTAF

                     650       660       670       680       690       700
PCH45_gp216        AGEDFAADLLDRSNNPKASEEIFERIMEADRPSDMKFVLDRKKLPVGSSVTHQYLNNAIY
201phi2-1_gp129    VGPEPTMELVDQTNNPLAHVEVVEQFLTQEKPTRIDRAVDRKKIPFGNSRPVSLLNHMMQ
PhiKZ_gp071+073    VGPGPTAEILDQTNNPLAHAAVIESWLTAEKPSSVPVAVDREKIPFGGSRPVAMFDHLLE
PhiPA3_gp065+066   VGPEATVELLDQTNNPRAHLAVINSILTADKPSNIERAVDRTKVPFGSSRPVDLLEHLLE
RAY_gp248          CRGYYANRLQSMANTPAMQKEAALMFLRHDTPMNIPNVIDETKTPLGRARPLQMYKHNQG
Goslar_gp171       AGGWFASVFMDYANNPKQVDSITESIYATDKPSAIPIATNFEKYPIGGNRALRILYHQLN

                     710       720
PCH45_gp216        TAGARFNRIIADKKGGKR..................
201phi2-1_gp129    TRGIEFKYASSPSASH....................
PhiKZ_gp071+073    CSGIALEYAPDH........................
PhiPA3_gp065+066   CRGLKFEYATTDGVQPVHTAVPIRAQQKVKSEAIEE
RAY_gp248          CRGIEFETTVLNK.......................
Goslar_gp171       VAGGQIIDADDCES......................
```

iii

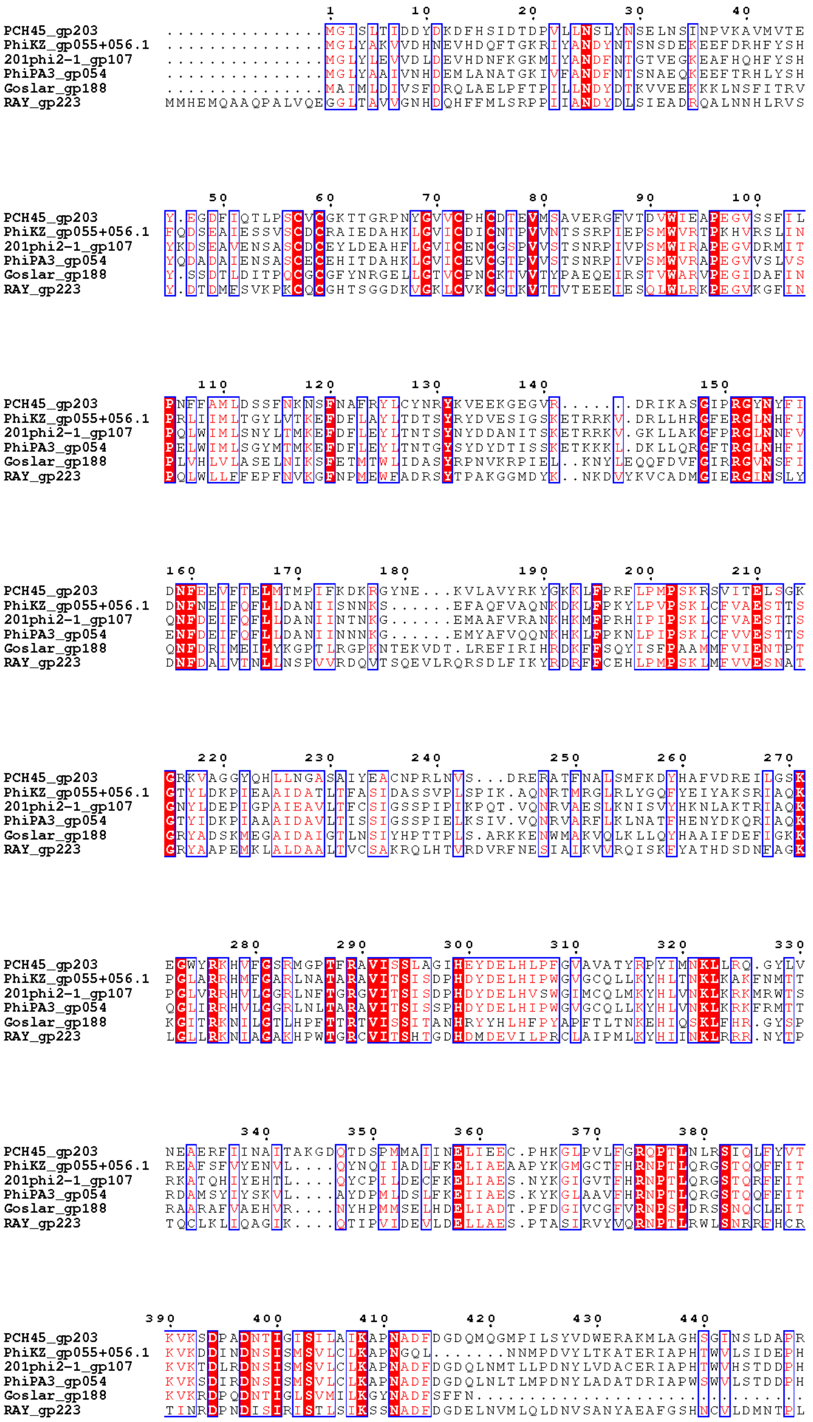

PCH45_gp203 ...............MGISLTIDDYDKDFHSIDTDPVLLNSLYNSELNSINPVKAVMVTE
PhiKZ_gp055+056.1 ...............MGLYAKVVDHNEVHDQFTGKRIYANDYNTSNSDEKEEFDRHFYSH
201phi2-1_gp107 ...............MGLYLEVVDLDEVHDNFKGKMIYANDFNTGTVEGKEAFHQHFYSH
PhiPA3_gp054 ...............MGLYAAIVNHDEMLANATGKIVFANDFNTSNAEQKEEFTRHLYSH
Goslar_gp188 ...............MAIMLDIVSFDRQLAELPFTPILLNDYDTKVVEEKKKLNSFITRV
RAY_gp223 MMHEMQAAQPALVQEGGLTAVVGNHDQHFFMLSRPPIIANDYDLSIEADRQALNNHLRVS
PCH45_gp203 Y.EGDFIQTLPSCVCGKTTGRPNYCVVCPHCDTEVMSAVERGFVTDVWIEAPEGVSSFIL
PhiKZ_gp055+056.1 FQDSEAIESSVSCDCRAIEDAHKLCVICDICNTPVVNTSSRPIEPSMWVRTPKHVRSLIN
201phi2-1_gp107 YKDSEAVENSASCDCEYLDEAHFLCVICENCGSPVVSTSNRPIVPSMWIRAPEGVDRMIT
PhiPA3_gp054 YQDADAIENSASCECEHITDAHKLCVICEVCGTPVVSTSNRPIVPSMWVRAPEGVVSLVS
Goslar_gp188 Y.SSDTLDITPQCGCGFYNRGELCTVCPNCKTVVTYPAEQEIRSTVWARVPEGIDAFIN
RAY_gp223 Y.DTDMFSVKPKCQCGHTSGGDKVCKLCVKCGTKVTTVTEEEIESQLWLRKPEGVKGFIN
PCH45_gp203 PNFFAMLDSSFNKNSFNAFRYLCYNRYKVEEKGEGVR......DRIKASGIPRGYNYFI
PhiKZ_gp055+056.1 FRLIIMLTGYLVTKEFDFLAYLTDTSYRYDVESIGSKETRRKV.DRLLHRGFERGLNHFI
201phi2-1_gp107 FQLWIMLSNYLTMKEFDFLEYLTNTSYNYDDANITSKETRRKV.GKLLAKGFPRGLNNFV
PhiPA3_gp054 FELWIMLSGYMTMKEFDFLEYLTNTGYSYDYDTISSKETKKKL.DKLLQRGFTRGLNHFI
Goslar_gp188 FLVHLVLASELNIKSFETMTWLIDASYRPNVKRPIEL..KNYLEQQFDVFGIRRGVNSFI
RAY_gp223 PQLWLLFFEPFNVKGFNPMEWFADRSYTPAKGGMDYK..NKDVYKVCADMCIERGINSLY
PCH45_gp203 DNFEEVFTELMTMPIFKDKRGYNE...KVLAVYRKYGKKLFPRFLPMPSKRSVITELSGK
PhiKZ_gp055+056.1 DNFNEIFQFLLDANIISNNKS......EFAQFVAQNKDKLFPKYLPVPSKLCFVAESTTS
201phi2-1_gp107 QNFDEIFQFLLDANIINTNKG......EMAAFVRANKHKMFPRHIPIPSKLCFVAESTTS
PhiPA3_gp054 ENFDEIFQFLLDANIINNNKG......EMYAFVQQNKHKLFPKNLPIPSKLCFVVESTTS
Goslar_gp188 QNFDRIMEILYKGPTLRGPKNTEKVDT.LREFIRIHRDKFFSQYISFPAAMMFVIENTPT
RAY_gp223 DNFDAIVTNLLNSPVVRDQVTSQEVLRQRSDLFIKYRDKFFCEHLPMPSKLMFVVESNAT
PCH45_gp203 GRKVAGGYQHLLNGASAIYEACNPRLNVS...DRERATFNALSMFKDYHAFVDREILGSK
PhiKZ_gp055+056.1 GTYLDKPIEAAIDATLTFASIDASSVPLSPIK.AQNRTMRGLRLYGQFYEIYAKSRIAQK
201phi2-1_gp107 GNYLDEPIGPAIEAVLTFCSIGSSPIPIKPQT.VQNRVAESLKNISVYHKNLAKTRIAQK
PhiPA3_gp054 GTYIDKPIAAAIDAVLTISSIGSSPIELKSIV.VQNRVARFLKLNATFHENYDKQRIAQK
Goslar_gp188 GRYADSKMEGAIDAIGTLNSIYHPTTPLS.ARKKENWMAKVQLKLLQYHAAIFDEFIGKK
RAY_gp223 GRYAAPEMKLALDAALTVCSAKRQLHTVRDVRFNESIAIKVVRQISKFYATHDSDNFAGK
PCH45_gp203 EGWYRKHVFGSRMGPTFRAVISSLAGIHEYDELHLPFGVAVATYRPYIMNKLLRQ.GYLV
PhiKZ_gp055+056.1 PGLARRHMFGARLNATARAVITSISDPHDYDELHIPWGVGCQLLKYHLTNKLKAKFNMTT
201phi2-1_gp107 PGLVRRHVLGGRLNFTGRGVITSISDPHDYDELHVSWGIMCQLMKYHLVNKLKRKMRWTS
PhiPA3_gp054 QGLIRRHVLGGRLNLTARAVITSISSPHDYDELHIPWGVGCQLLKYHLVNKLKRKFRMTT
Goslar_gp188 KGITRKNILGTLHPFTRTVISSITANHRYYHLHFPYAPFTLTNKEHIQSKLFHR.GYSP
RAY_gp223 LGLLRKNIAGAKHPWTGRCVITSHTGDHDMDEVILPRCLAIPMLKYHIINKLRRR.NYTP
PCH45_gp203 NEAERFTINAITAKGDQTDSPMMAIINEELIEEC.PHKGLPVLFGRQPTLNLRSIQLFYVT
PhiKZ_gp055+056.1 REAFSFVYENVL....QYNQIIADLFKELIAEAAPYKGMGCTFHRNPTLQRGSTQQFFIT
201phi2-1_gp107 RKATQHIYEHTL....QYCPILDECFKELIAES.NYKGIGVTFHRNPTLQRGSTQRFFIT
PhiPA3_gp054 RDAMSYIYSKVL....AYDPMLDSLFKEIIAES.KYKGLAAVFHRNPTLQRGSTQQFFIT
Goslar_gp188 RAARAFVAEHVR....NYHPMMSELHDELIADT.PFDGIVCGFVRNPSLDRSSNQCLEIT
RAY_gp223 TQCLKLIQAGIK....QTIPVIDEVLDELLAES.PTASIRVYVQRNPTLRWLSNRRFHCR
PCH45_gp203 KVKSDPADNTIGISILAIKAPNADFDGDQMQGMPILSYVDWERAKMLAGHSGINSLDAPR
PhiKZ_gp055+056.1 KVKDDINDNSISMSVLCLKAPNGQL.......NNMPDVYLTKATERIAPHTWVLSIDEPH
201phi2-1_gp107 KVKTDLRDNSISMSVLCLKAPNADFDGDQLNMTLLPDNYLVDACERIAPHTWVHSTDDPH
PhiPA3_gp054 KVKSDIRDNSISMSVLCLKAPNADFDGDQLNLTLMPDNYLADATDRIAPWSWVLSTDDPH
Goslar_gp188 KVKRDPQDNTIGLSVMILKGYNADFSFFN...........................
RAY_gp223 TINRDPNDISIRISTLSIKSSNADFDGDELNVMLQLDNVSANYAEAFGSHNCVLDMNTPL

```
                      450       460       470
                      .         .         .
PCH45_gp203           SLNRNLVMPDTDFSTLNNFMYGNSR...........
PhiKZ_gp055+056.1     EISGNLELQGPVVETIINWAHEKYLPPLEEWLKAK.
201phi2-1_gp107       QLSGNLELQGPVVETVVNYGHEDYLPPLEEWLAGH.
PhiPA3_gp054          EISGNLELQGPVVETIINFAHEDYLPPLEEWLKMAA
Goslar_gp188          ....................................
RAY_gp223             KISGDVGLPGTLISTMNRWMYSNDD...........
```

iv

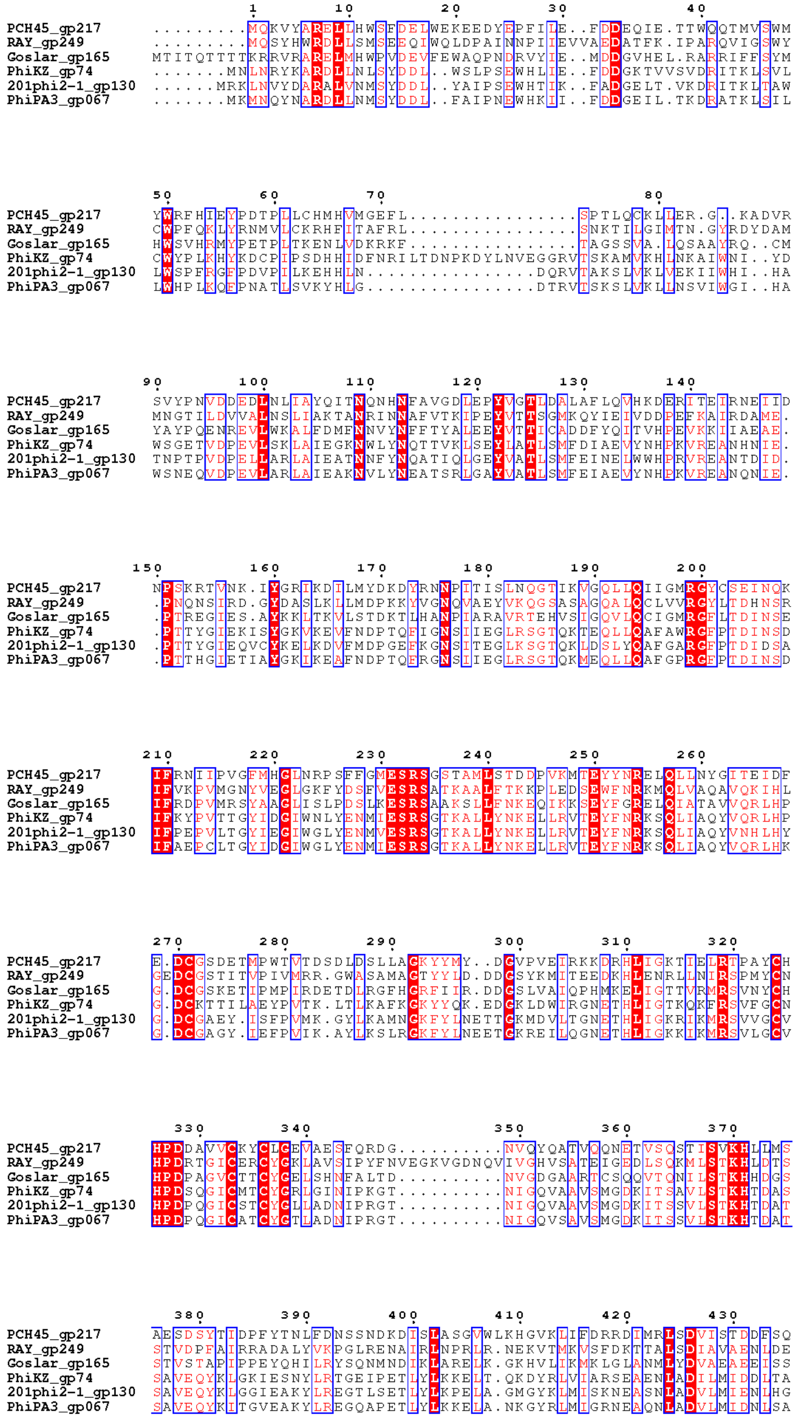

PCH45_gp217 .........MQKVYARELLHWSFDELWEKEEDYEPFILE..FDDEQIE.TTWQQTMVSWM
RAY_gp249 .........MQSYHWRDLLSMSEEQIWQLDPAINNPIIEVVAEDATFK.IPARQVIGSWY
Goslar_gp165 MTITQTTTTKRRVRARELMHWPVDEVFEWAQPNDRVYIE..MDDGVHEL.RARRIFFSYM
PhiKZ_gp74 .......MNLNRYKARDLLNLSYDDL..WSLPSEWHLIE..FDDGKTVVSVDRITKLSVL
201phi2-1_gp130 ......MRKLNVYDARALVNMSYDDL..YAIPSEWHTIK..FADGELT.VKDRITKLTAW
PhiPA3_gp067 .......MKMNQYNARDLLNMSYDDL..FAIPNEWHKII..FDDGEIL.TKDRATKLSIL
PCH45_gp217 YWRFHIEYPDTPLLCHMHVMGEFL................SPTLQCKLLER.G..KADVR
RAY_gp249 CWPFQKLYRNMVLCKRHFITAFRL................SNKTILGIMTN.GYRDYDAM
Goslar_gp165 HWSVHRMYPETPLTKENLVDKRKF................TAGSSVA.LQSAAYRQ..CM
PhiKZ_gp74 CWYPLKHYKDCPIPSDHHIDFNRILTDNPKDYLNVEGGRVTSKAMVKHLNKAIWNI..YD
201phi2-1_gp130 LWSPFRGFPDVPILKEHHLN................DQRVTAKSLVKLVEKIIWHI..HA
PhiPA3_gp067 LWHPLKQFPNATLSVKYHLG................DTRVTSKSLVKLLNSVIWGI..HA
PCH45_gp217 SVYPNVDDEDLNLIAYQITNQNHNFAVGDLEPYVGTLDALAFLQVHKDERITEIRNEIID
RAY_gp249 MNGTILDVVALNSLIAKTANRINNAFVTKIPEYVTTSGMKQYIEIVDDPEFKAIRDAME.
Goslar_gp165 YAYPQENREVLWKALFDMFNNVYNFFTYALEEYVTTICADDFYQITVHPEVKKIIAEAE.
PhiKZ_gp74 WSGETVDPEVLSKLAIEGKNWLYNQTTVKLSEYLATLSMFDIAEVYNHPKVREANHNIE.
201phi2-1_gp130 TNPTPVDPELLARLAIEATNNFYNQATIQLGEYVATLSMFEINELWWHPRVREANTDID.
PhiPA3_gp067 WSNEQVDPEVLARLAIEAKNVLYNEATSRLGAYVATLSMFEIAEVYNHPKVREANQNIE.
PCH45_gp217 NPSKRTVNK.IYGRIKDILMYDKDYRNNPITISLNQGTIKVGQLLQIIGMRGYCSEINQK
RAY_gp249 .PNQNSIRD.GYDASLKLLMDPKKYVGNQVAEYVKQGSASAGQALQCLVVRGYLTDHNSR
Goslar_gp165 .PTREGIES.AYKKLTKVLSTDKTLHANPIARAVRTEHVSIGQVLQCIGMRGFLTDINSE
PhiKZ_gp74 .PTTYGIEKISYGKVKEVFNDPTQFIGNSIIEGLRSGTQKTEQLLQAFAWRGFPTDINSD
201phi2-1_gp130 .PTTYGIEQVCYKELKDVFMDPGEFKGNSITEGLKSGTQKLDSLYQAFGARGFPTDIDSA
PhiPA3_gp067 .PTTHGIETIAYGKIKEAFNDPTQFRGNSIIEGLRSGTQKMEQLLQAFGPRGFPTDINSD
PCH45_gp217 IFRNIIPVGFMHGLNRPSFFGMESRSGSTAMLSTDDPVKMTEYYNRELQLLNYGITEIDF
RAY_gp249 IFVKPVMGNYVEGLGKFYDSFVESRSATKAALFTKKPLEDSEWFNRKMQLVAQAVQKIHL
Goslar_gp165 IFRDPVMRSYAAGLISLPDSLKESRSAAKSLLFNKEQIKKSEYFGRELQIATAVVQRLHP
PhiKZ_gp74 IFKYPVTTGYIDGIWNLYENMIESRSGTKALLYNKELLRVTEYFNRKSQLIAQYVQRLHP
201phi2-1_gp130 IFPEPVLTGYIEGIWGLYENMVESRSGTKALLYNKELLRVTEYFNRKSQLIAQYVNHLHY
PhiPA3_gp067 IFAEPCLTGYIDGIWGLYENMIESRSGTKALLYNKELLRVTEYFNRKSQLIAQYVQRLHK
PCH45_gp217 E.DCGSDETMPWTVTDSDLDSLLAGKYYMY..DGVPVEIRKKDRHLIGKTIELRTPAYCH
RAY_gp249 GEDCGSTITVPIVMRR.GWASAMAGTYYLD.DDGSYKMITEEDKHLENRLLNIRSPMYCN
Goslar_gp165 G.DCGSKETIPMPIRDETDLRGFHGRFIIR.DDGSLVAIQPHMKELIGTTVRMRSVNYCH
PhiKZ_gp74 G.DCKTTILAEYPVTK.LTLKAFKGKYYQK.EDGKLDWIRGNETHLIGTKQKFRSVFGCN
201phi2-1_gp130 G.DCGAEY.ISFPVMK.GYLKAMNGKFYLNETTGKMDVLTGNETHLIGKRIKMRSVVGCV
PhiPA3_gp067 G.DCGAGY.IEFPVIK.AYLKSLRGKFYLNEETGKREILQGNETHLIGKKIKMRSVLGCV
PCH45_gp217 HPDDAVVCKYCLGEVAESFQRDG..........NVQYQATVQQNETVSQSTISVKHLLMS
RAY_gp249 HPDRTGICERCYGKLAVSIPYFNVEGKVGDNQVIVGHVSATEIGEDLSQKMLSTKHLDTS
Goslar_gp165 HPDPAGVCTTCYGELSHNFALTD..........NVGDGAARTCSQQVTQNILSTKHHDGS
PhiKZ_gp74 HPDSQGICMTCYGRLGINIPKGT..........NIGQVAAVSMGDKITSAVLSTKHTDAS
201phi2-1_gp130 HPDPQGICSTCYGLLADNIPRGT..........NIGQVAAVSMGDKITSSVLSTKHTDAT
PhiPA3_gp067 HPDPQGICATCYGTLADNIPRGT..........NIGQVSAVSMGDKITSSVLSTKHTDAT
PCH45_gp217 AESDSYTIDPFYTNLFDNSSNDKDISLASGVWLKHGVKLIFDRRDIMRLSDVISTDDFSQ
RAY_gp249 STVDPFAIRRADALYVKPGLRENAIRLNPRLR.NEKVTMKVSFDKTTALSDIAVAENLDE
Goslar_gp165 STVSTAPIPPEYQHILRYSQNMNDIKLARELK.GKHVLIKMKLGLANMLYDVAEAEEISS
PhiKZ_gp74 SAVEQYKLGKIESNYLRTGEIPETLYLKKELT.QKDYRLVIARSEAENLADILMIDDLTA
201phi2-1_gp130 SAVEQYKLGGIEAKYLREGTLSETLYLKPELA.GMGYKLMISKNEASNLADVLMIENLHG
PhiPA3_gp067 SAVEQYKITGVEAKYLREGQAPETLYLKKELA.NKGYRLMIGRNEAQNLADVLMIDNLSA

```
                  440       450       460       470       480       490
PCH45_gp217       HDVSTFAKIRTVTLVYQDDHKKDPEVHSVPLNHGSYMPFLTAEFLAYIRDYGYDIV.EGN
RAY_gp249         VA.TRVSGFNEIVLEF.EREDGGKESIPINTTQGSRQGQFTVDFLRYLQRVSWTSADKDY
Goslar_gp165      LIPQRLFNMTLCDMEIYDRKDESYRKLKVNFDFCGRPVVPSKAFIRYLKRHSWEVTSDGI
PhiKZ_gp74        YPATSATELTSLALVY.DDEVNGECGDVLTVSLYNRRASLSIEMLKHIKMVRWELDQRDN
201phi2-1_gp130   YPPSSATEMTKIGLVR.QVD.GVDVGDVLTVSLYNRKASLSLEVLKHVKKVQWQPDDRGN
PhiPA3_gp067      YPPTSASELTRIGLVR.TVD.GIDEGDVLTVSLYNRKASLSIELLQHVKRVRWELDNRDN

                    500       510       520       530         540
PCH45_gp217       IEVSLAHWPEGEVIFRLPERRSTVLEAAIMLKKEIFAIGDEAK.........EARVRLRM
RAY_gp249         ISIRLDQFEYDCDVVELPLVHEDMMAYQKQIESYIRFSKESANWKNKFVTPDEV......
Goslar_gp165      VIFDLKHWDNSRTLFQMPLIHKNMMEYAKEIEREFKFGKGSFS..AGFLSEDEATPE...
PhiKZ_gp74        IVISLRGFDFNLPFLTLPNKHVNMYEVMKRFQSFLHSGSDSAE..AGKLSTEKVGYTSKT
201phi2-1_gp130   IVIDLDGFDHTQPFLTLPYKHVNMYEVMKRIQSFLHSGSDT.E..GSKLSSDKVGFTSKT
PhiPA3_gp067      IVIDLNGFDFSLPFLTLPYKHVNMYEVMKRIQSFLHSGSDT.E..GSKLSSDKVGFTSKT

                  550       560       570       580       590       600
PCH45_gp217       NLRDPMLMAKAIRDIAELTNARFHVSLPIIELVMLAMMARDPENGDFRLPRKGTGARFAP
RAY_gp249         ........GVVLDEFFSLLRQRLGVNIVHAQIMLYSVMTMDPAKGDYRLPRAYEPRQFSS
Goslar_gp165      ......EVANVLYYWHRLCAQRLRMNLSHLDVILYASMIRSPHTKDYRMPKQGTTRYFST
PhiKZ_gp74        YLKNYKSPIEALPVFATMANEKISLNISHCEILIYAMMIRSAQYRDYRLPKPGINGQFEK
201phi2-1_gp130   YLKNYENVIEGVVATASLINEKINLPLVHCEVLAYAMTIKSAQRKDYNLPKPGYLGQFEK
PhiPA3_gp067      YLKNYNDPIDAVAAFASLVNEKIQLPMPHCEVLVYAMMVRSTQQRDYRLPKPGISGQFEK

                  610       620       630       640       650       660
PCH45_gp217       QRAIMNGRSLGGKAPYERQYEMVVSPDSYLNEDRPDHPMDELLVPLGRRSSKWRPRAPIE
RAY_gp249         YHECIEYRSLSVQLVYQEQAAVMLKPSTFLNDKRQPHPMDEIFK................
Goslar_gp165      FKKNMNMRSLSMKFAHQEQLDAFQNPNSYIPTMKPDHPIDYMLLPRKEPKP....EDVIT
PhiKZ_gp74        YNRLMQCRSLGGAMAFEKQHEPLNNPGSFLNKMRNDHPYDLLVKGGKLR...........
201phi2-1_gp130   YNKLMHSRDLAGTMAFEKQHEPFANPASFLYTERNDHPYGLMVRGGQLN...........
PhiPA3_gp067      YNKLMQSRSLAGAMAFEKQHEPLNNPGSFLYTLRNDHPYDLAVKGGKLY...........

PCH45_gp217       KS
RAY_gp249         ..
Goslar_gp165      L.
PhiKZ_gp74        ..
201phi2-1_gp130   ..
PhiPA3_gp067      ..
```

E

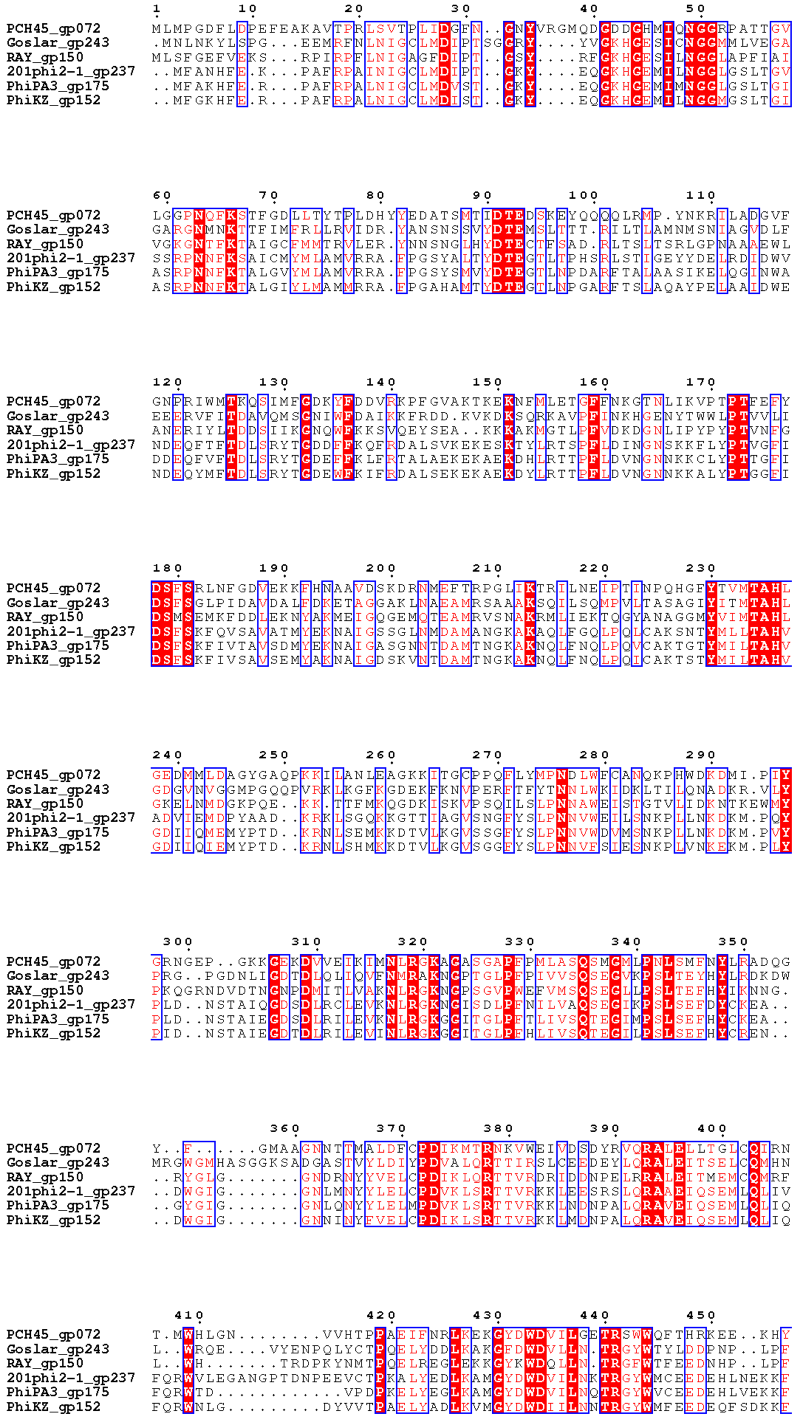
1 10 20 30 40 50
PCH45_gp072 MLMPGDFLDPEFEAKAVTPRLSVTPLIDGEN..CNYVRGMQDCDDGHMIQNGGRPATTGV
Goslar_gp243 .MNLNKYLSPG...EEMRFNLNIGCLMDIPTSGGKY....YVGKHGESICNGGMMLVEGA
RAY_gp150 MLSFGEFVEKS...RPIRPFLNIGAGFDIPT..GSY....RFGKHGESILNGGLAPFIAI
201phi2-1_gp237 ..MFANHFE.K...PAFRPALNIGCLMDIPT..GKY....EQGKHGEMILNGGLGSLTGV
PhiPA3_gp175 ..MFAKHFE.R...PAFRPALNIGCLMDVST..GKY....EQGKHGEMIMNGGLGSLTGI
PhiKZ_gp152 ..MFGKHFE.R...PAFRPALNIGCLMDIST..GKY....EQGKHGEMILNGGMGSLTGI
60 70 80 90 100 110
PCH45_gp072 LGGPNQEKSTFGDLLTYTPLDHYYEDATSMTIDTEDSKEYQQQQLRMP.YNKRILADGVF
Goslar_gp243 GARGNMNKTTFIMFRLLRVIDR.YANSNSSVYDTEMSLTTT.RILTLAMNMSNIAGVDLF
RAY_gp150 VGKGNTEKTAICMFMMTRVLER.YNNSNGLHYDTECTFSAD.RLTSLTSRLGPNAAAEWL
201phi2-1_gp237 SSRPNNFKSAICMYMLAMVRRA.FPGSYALTYDTEGTLTPHSRLSTIGEYYDELRDIDWV
PhiPA3_gp175 ASRPNNFKTALGVYMLAMVRRA.FPGSYSMVYDTEGTLNPDARFTALAASIKELQGINWA
PhiKZ_gp152 ASRPNNFKTALGIYLMAMMRRA.FPGAHAMTYDTEGTLNPGARFTSLAQAYPELAAIDWE
120 130 140 150 160 170
PCH45_gp072 GNPRIWMTKQSIMFGDKYFDDVRKPFGVAKTKEKNFMLETGFFNKGTNLIKVPTPTFEFY
Goslar_gp243 EEERVFITDAVQMSGNIWFDAIKKFRDD.KVKDKSQRKAVPFINKHGENYTWWLPTVVLI
RAY_gp150 ANERIYLTDDSIIKGNQWFKKSVQEYSEA..KKKAKMGTLPFVDKDGNLIPYPYPTVNFG
201phi2-1_gp237 NDEQFTFTDLSRYTGDDFFKQFRDALSVKEKESKTYLRTSPFLDINGNSKKFLYPTVGFI
PhiPA3_gp175 DDEQFVFTDLSRYTGDEFFKLFRTALAEKEKAEKDHLRTTPFLDVNGNNKKCLYPTTGFI
PhiKZ_gp152 NDEQYMFTDLSRYTGDEWFKIFRDALSEKEKAEKDYLRTTPFLDVNGNNKKALYPTGGFI
180 190 200 210 220 230
PCH45_gp072 DSFSRLNFGDVEKKFHNAAVDSKDRNMEFTRPGLIKTRILNEIFTINPQHGFYTVMTAHL
Goslar_gp243 DSFSGLPIDAVDALFDKETAGGAKLNAEAMRSAAAKSQILSQMPVLTASAGIYITMTAHL
RAY_gp150 DSMSEMKFDDLEKNYAKMEIGQGEMQTEAMRVSNAKRMLIEKTQGYANAGGMYVIMTAHL
201phi2-1_gp237 DSFSKFQVSAVATMYEKNAIGSSGLNMDAMANGKAKAQLFGQLPQLCAKSNTYMLLTAHV
PhiPA3_gp175 DSFSKFIVTAVSDMYEKNAIGASGNNTDAMTNGKAKNQLFNQLPQVCAKTGTYMILTAHV
PhiKZ_gp152 DSFSKFIVSAVSEMYAKNAIGDSKVNTDAMTNGKAKNQLFNQLPQICAKTSTYMILTAHV
240 250 260 270 280 290
PCH45_gp072 GEDMMLDAGYGAQPKKILANLEAGKKITGCPPQFLYMPNDLWFCANQKPHWDKDMI.PIY
Goslar_gp243 GDGVNVGGMPGQQPVEKLKGFKGDEKFKNVPERFTFYTNNLWKIDKLTILQNADKR.VLY
RAY_gp150 GKELNMDGKPQE..KK.TTFMKQGDKISKVPSQILSLPNNAWEISTGTVLIDKNTKEWMY
201phi2-1_gp237 ADVIEMDPYAAD..KRKLSGQKKGTTIAGVSNGFYSLPNNVWEILSNKPLLNKDKM.PQY
PhiPA3_gp175 GDIIQMEMYPTD..KRNLSEMKKDTVLKGVSSGFYSLPNNVWDVMSNKPLLNKDKM.PVY
PhiKZ_gp152 GDIIQIEMYPTD..KRNLSHMKKDTVLKGVSGGEYSLPNNVFSIESNKPLVNKEKM.PLY
300 310 320 330 340 350
PCH45_gp072 GRNGEP..GKKGEKDVVEIKIMNLRGKACASGAPEPMLASQSMGMLPNLSMFNYLRADQG
Goslar_gp243 PRG..PGDNLIGDTDLQLIQVFNMRAKNCPTGLPFPIVVSQSEGVKPSLTEYHYLRDKDW
RAY_gp150 PKQGRNDVDTNGNPDMITLVAKNLRGKNGPSGVPWEFVMSQSEGLLPSLTEFHYIKNNG.
201phi2-1_gp237 PLD..NSTAIQGDSDLRCLEVKNLRGKNGISDLPENILVAQSEGIKPSLSEFDYCKEA..
PhiPA3_gp175 PLD..NSTAIEGDSDLRILEVKNLRSKGGITGLPETLIVSQTEGIMPSLSEFHYCKEA..
PhiKZ_gp152 PID..NSTAIEGDTDLRILEVINLRGKGGITGLPEHLIVSQTEGILPSLSEFHYCREN..
360 370 380 390 400
PCH45_gp072 Y..F.....GMAAGNNTTMALDFCPDIKMTRNKVWEIVDSDYRVQRALELLTGLCQIRN
Goslar_gp243 MRGWGMHASGGKSADGASTVYLDIYPDVALQRTTIRSLCEEDEYLQRALEITSELCQMHN
RAY_gp150 ..RYGLG.......GNDRNYYVELCPDIKLQRTTVRDRIDDNPELRRALEITMEMCQMRF
201phi2-1_gp237 ..DWGIG.......GNLMNYYLELCPDVKLSRTTVRKKLEESRSLQRAAEIQSEMLQLIV
PhiPA3_gp175 ..GYGIG.......GNLQNYYLELMPDVKLSRTTVRKKLNDNPALQRAVEIQSEMLQLIQ
PhiKZ_gp152 ..DWGIG.......GNNINYFVELCPDIKLSRTTVRKKLMDNPALQRAVEIQSEMLQLIQ
410 420 430 440 450
PCH45_gp072 T.MWHLGN........VVHTPPAEIFNRLKEKGYDWDVILGETRSWWQFTHRKEE..KHY
Goslar_gp243 L..WRQE....VYENPQLYCTPQELYDDLKAKGFDWDVILN.TRGYWTYLDDPNP..LPF
RAY_gp150 L..WH.......TRDPKYNMTPQELREGLEKKGYKWDQLLN.TRGFWTFEEDNHP..LPF
201phi2-1_gp237 FQRWVLEGANGPTDNPEEVCTPKALYEDLKAMGYDWDVILNKTRGYWMCEEDEHLNEKKF
PhiPA3_gp175 FQRWTD............VPDPKELYEGLKAMGYDWDVILNQTRGYWVCEEDEHLVEKKF
PhiKZ_gp152 FQRWNLG.........DYVVTPAELYADLKVMGYDWDIILNNTRGYWMFEEDEQFSDKKF

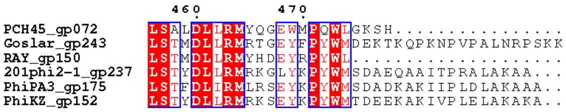
460
470
PCH45_gp072 LSALDLLRMYQGEWMPQWLGKSH................
Goslar_gp243 LSTMDLLRMRTGEYFPYWMDEKTKQPKNPVPALNRPSKK
RAY_gp150 LSTMDLLRMYHDEYRPYWL....................
201phi2-1_gp237 LSTYDLLRMRKGLYKPYWMSDAEQAAITPRALAKAA...
PhiPA3_gp175 LSTFDLIRMLRSEYKPYWMSDADKAKIIPLDLAKAAA..
PhiKZ_gp152 LSTYDLLRMRKSEYKPYWMTDEEKAKIVPLELAKAKA..

F

```
Goslar_gp241  ............................................................
RAY_gp153     MSDNARVLPDPREDGKTHINVYSRGASWLGQQLSNMSYYDFAHPRYGVFASLEGFWYWLS
AH06_gp160    .MSDVRVLPDPREDGKSHMNVYSRGATWLGQQLSNMAYYNFAHPKYGVFASLEGFWYWLA

Goslar_gp241  ............................................................
RAY_gp153     TGKQHEELRKLAGVKAKMTGREFETIPNENFEEEFKEAMRLRLEQHPIIANALAESLLPL
AH06_gp160    TGKQHEELRNLAGVKAKMVGRDFEAIPLDTFEEEFKEAMRLRLEQHPIIANALAESILPL

Goslar_gp241  ............................................................
RAY_gp153     KHYYCYGGKVIDLYDRHKWQMDFYEEWRKANAPEDTTLVLLISGSRKEKDYDAFKHIVMT
AH06_gp160    EHYYCYGGKVIDLYERHKWQMEFYEQWRKENAPEDHSIVLLISGSRKEKDYDSFKNIVMT

                1        10        20        30        40        50
Goslar_gp241  ..MNQMLHNLPHDKVHLIAGGARSGADLHVRRYAKDYGFKYTTFPADWDGPYRKGAGFRR
RAY_gp153     YLQPYIDKIKDTYKITLLSGLAWEGPDDMAIRLCREEGFMLIGLPAKWKEQ.GKAAGMIR
AH06_gp160    YLQPYIERNKDTYKITLLSGLAWEGPDDMAIRLCREEGFMLVGLPAKWKEQ.GKAAGMIR

              60        70        80        90       100
Goslar_gp241  NEWMGDVITHLIAFWDFKSPGTKHMIDIANREDKNIIVQVFRVFPQQTRQ....
RAY_gp153     NGAMGRLCNKALVFWDGESPGTKGMIDYLKKNTIDHLVYLHGKHEPDWKAPETS
AH06_gp160    NGAMGRLCNKALVFWDGESPGTKGMIDYLKKNTIDHLVYMHGKHEPDWKAPEAA
```

G

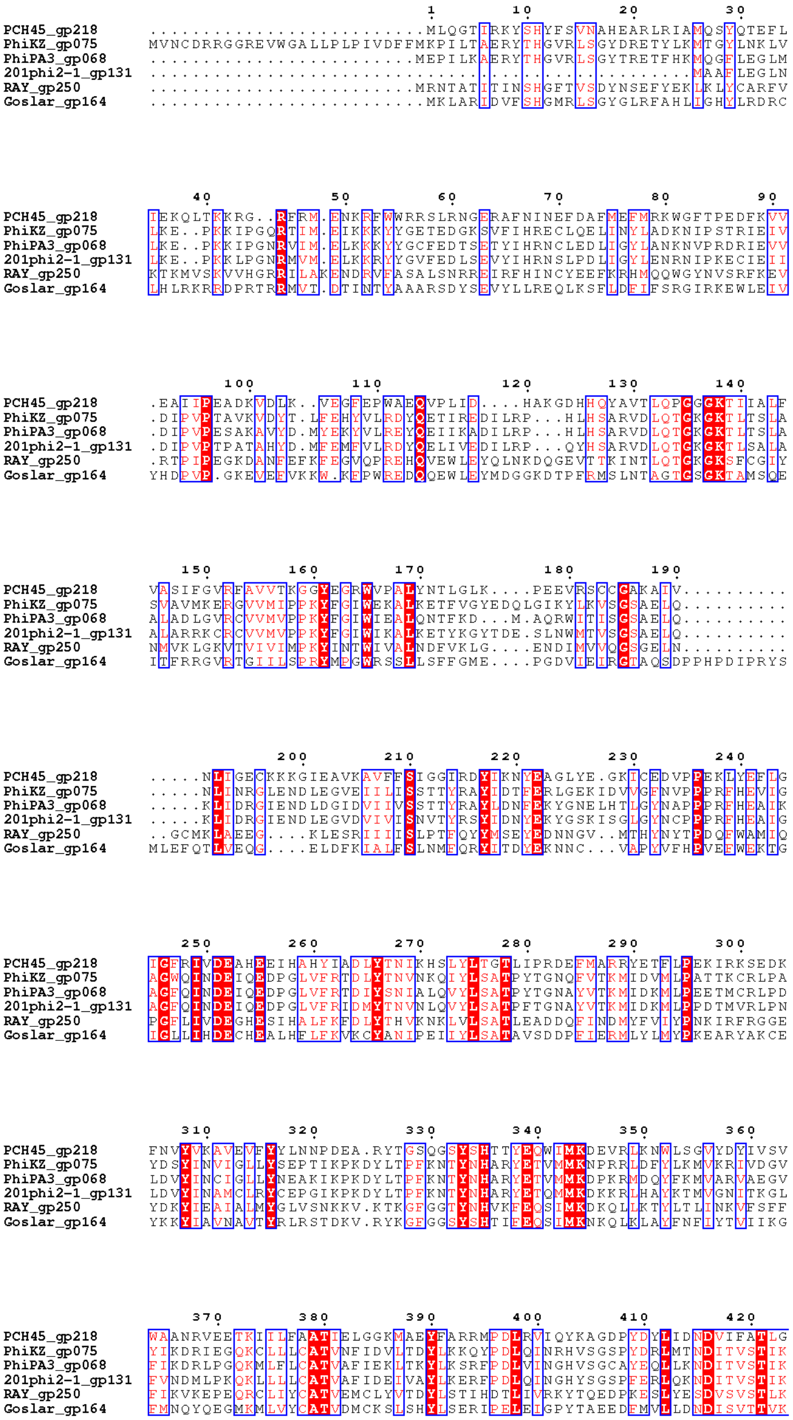
PCH45_gp218
PhiKZ_gp075
PhiPA3_gp068
201phi2-1_gp131
RAY_gp250
Goslar_gp164

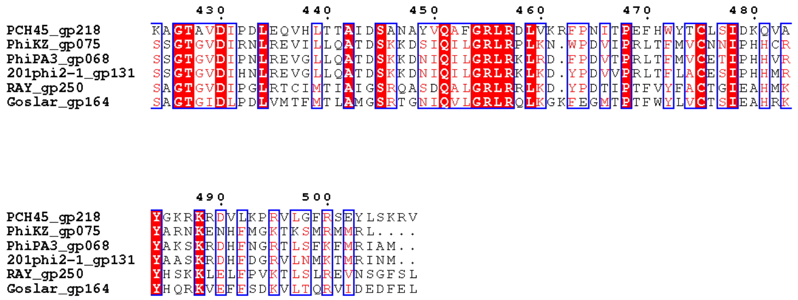
430 440 450 460 470 480
PCH45_gp218 KAGTAVDIPDLEQVHLTTAIDSANAYVQAFGRLRDLVKRFPNITPEFHWYTCLSIDKQVA
PhiKZ_gp075 SSGTGVDIRNLREVILLQATDSKKDSIQILGRLRPLKN.WPDVIPRLTFMVCNNIPHHCR
PhiPA3_gp068 SSGTGVDIPNLREVGLLQATDSKKDNIQILGRLRKLRD.FPDVTPRLTFMVCETIPHHVR
201phi2-1_gp131 SSGTGVDIHNLREVGLLQATDSKKDNIQILGRLRKLKD.YPDVVPRLTFLACESIPHHMR
RAY_gp250 SAGTGVDIPGLRTCIMTIAIGSRQASDQALGRLRRLKD.YPDTIPTFVYFACTGIEAHMK
Goslar_gp164 SAGTGIDLPDLVMTFMTLAMGSRTGNIQVLGRLRQLKGKFEGMTPTFWYLVCTSIEAHRK
490 500
PCH45_gp218 YGKRKRDVLKPRVLGFRSEYLSKRV
PhiKZ_gp075 YARNKENHFMGKTKSMRMMRL....
PhiPA3_gp068 YAKSKRDHFNGRTLSFKFMRIAM..
201phi2-1_gp131 YAASKRDHFDGRVLNMKTMRINM..
RAY_gp250 YHSKKLELFPVKTLSLREVNSGFSL
Goslar_gp164 YHQRKVEFFSDKVLTQRVIDEDFEL

H

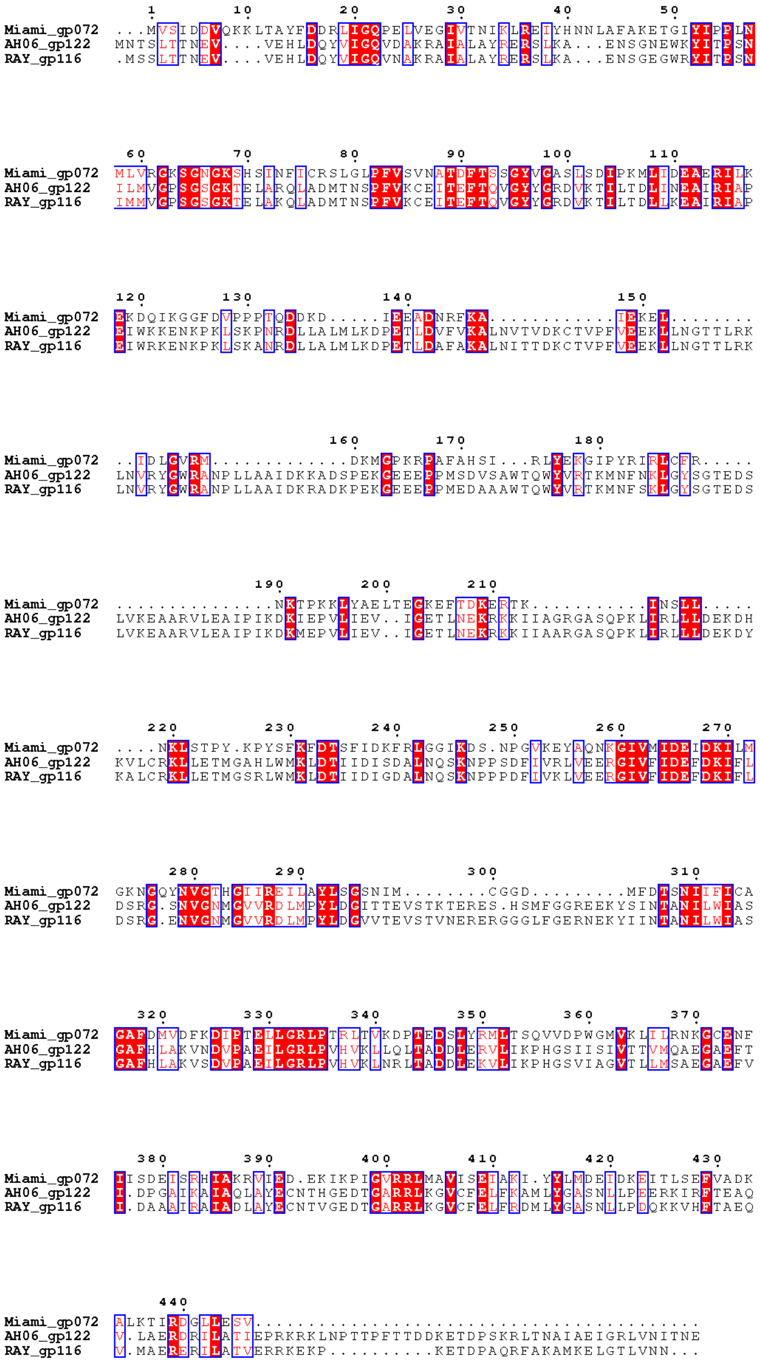
Miami_gp072 ...MVSIDDVQKKLTAYFDDRLIGQPELVEGIVTNIKLREIYHNNLAFAKETGIYIPPLN
AH06_gp122 MNTSLTTNEV....VEHLDQYVIGQVDAKRAIALAYRERSLKA...ENSGNEWKYITPSN
RAY_gp116 .MSSLTTNEV....VEHLDQYVIGQVNAKRAIALAYRERSLKA...ENSGEGWRYITPSN
Miami_gp072 MLVRGKSGNGKSHSINFICRSLGLPFVSVNATDFTSSGYVGASLSDIPKMLIDEAERILK
AH06_gp122 ILMVGPSGSGKTELARQLADMTNSPFVKCEITEFTQVGYYGRDVKTILTDLINEAIRIAP
RAY_gp116 IMMVGPSGSGKTELAKQLADMTNSPFVKCEITEFTQVGYYGRDVKTILTDLLKEAIRIAP
Miami_gp072 EKDQIKGGFDVPPPTQDDKD.....IEEADNRFKA............IEKEL........
AH06_gp122 EIWKKENKPKLSKPNRDLLALMLKDPETLDVFVKALNVTVDKCTVPFVEEKLLNGTTLRK
RAY_gp116 EIWRKENKPKLSKANRDLLALMLKDPETLDAFAKALNITTDKCTVPFVEEKLLNGTTLRK
Miami_gp072 ..IDLGVRM.............DKMGPKRPAFAHSI...RLYEKGIPYRIRLCFR.....
AH06_gp122 LNVRYGWRANPLLAAIDKKADSPEKGEEEPPMSDVSAWTQWYVRTKMNFNKLGYSGTEDS
RAY_gp116 LNVRYGWRANPLLAAIDKRADKPEKGEEEPPMEDAAAWTQWYVRTKMNFSKLGYSGTEDS
Miami_gp072 ...............NKTPKKLYAELTEGKEFTDKERTK...........INSLL.....
AH06_gp122 LVKEAARVLEAIPIKDKIEPVLIEV..IGETLNEKRKKIIAGRGASQPKLIRLLLDEKDH
RAY_gp116 LVKEAARVLEAIPIKDKMEPVLIEV..IGETLNEKRKKIIAARGASQPKLIRLLLDEKDY
Miami_gp072 ....NKLSTPY.KPYSFKFDTSFIDKFRLGGIKDS.NPGVKEYAQNKGIVMIDEIDKILM
AH06_gp122 KVLCRKLLETMGAHLWMKLDTIIDISDALNQSKNPPSDFIVRLVEERGIVFIDEFDKIFL
RAY_gp116 KALCRKLLETMGSRLWMKLDTIIDIGDALNQSKNPPPDFIVKLVEERGIVFIDEFDKIFL
Miami_gp072 GKNGQYNVGTHGIIREILAYISGSNIM........CGGD.........MFDTSNIIFICA
AH06_gp122 DSRG.SNVGNMGVVRDLMPYLDGITTEVSTKTERES.HSMFGGREEKYSINTANILWIAS
RAY_gp116 DSRG.ENVGNMGVVRDLMPYLDGVVTEVSTVNERERGGGLFGERNEKYIINTANILWIAS
Miami_gp072 GAFDMVDFKDIPTEILGRLPTRLTVKDPTEDSLYRMLTSQVVDPWGMVKLILRNKGCENF
AH06_gp122 GAFHLAKVNDVPAEHLGRLPVHVKLLQLTADDLERVLIKPHGSIISIVTTVMQAEGAEFT
RAY_gp116 GAFHLAKVSDVPAEHLGRLPVHVKLNRLTADDLEKVLIKPHGSVIAGVTLLMSAEGAEFV
Miami_gp072 IISDETSRHIAKRVIED.EKIKPIGVRRLMAVISEIAKI.YYLMDEIDKEITLSEFVADK
AH06_gp122 I.DPGAIKAIAQLAYECNTHGEDTGARRLKGVCFELFKAMLYGASNLLPEERKIRFTEAQ
RAY_gp116 I.DAAAIRAIADLAYECNTVGEDTGARRLKGVCFELFRDMLYGASNLLPDQKKVHFTAEQ
Miami_gp072 ALKTIRDGLLESV..
AH06_gp122 V.LAERDRILATIEPRKRKLNPTTPFTTDDKETDPSKRLTNAIAEIGRLVNITNE
RAY_gp116 V.MAERERILATVERRKEKP..........KETDPAQRFAKAMKELGTLVNN...

I

```
                   1        10        20        30        40        50        60
                   .         .         .         .         .         .         .
RAY_gp311          MTAEEIRKAMWVYDALSNSHRGLYVKAEGPDGSGKSTFVKLLTEWLQTQLGNSRAVMTRE
PCH45_gp050        ....................MAKLIVAEGLDFAGKSSFVEKFVEKLNAS..GYPTIHTYE
Goslar_gp008       ..................MSRNKYIVIEGLDYSGKSSVISSLHSRLN......NVCVVRE
PhiKZ_gp188        ...................MNGLFVCIEGTEGVGKTTVTKMVVEELRKR..NYDVVAMRE
201phi2-1_gp287    .................MSDNRTFIVLDGPDFSGKSTLMKAFLARVEAE..GIDHVAMRE
PhiPA3_gp223       ...............MFNPAVQKFVVLEGDEFSGKSSVRKALVERLEAL..NVPHQVFRE

                              70        80         90       100       110
                               .         .          .         .         .
RAY_gp311          MGGTAQ....GRVFREMVLFPKE..TQELDLQTETLLCWADRVEGQ.KQVKAWLNAGIAV
PCH45_gp050        PGGTNF....GNEISKITKYPRRLFDQDLTELTKITLFNASRLENLDKVILPALAEGKNV
Goslar_gp008       PGGTLY....GEAIRKLILEDQT.PTNRFDPETELLAMMSQRNHLFNKVIHPALNEGKTV
PhiKZ_gp188        PGGTAT....SEKIRQMLIWPEF.KDEVRPPEFDVLMHSAGRMLNVENIIRPALKDDKIV
201phi2-1_gp287    PGGIPGSGSLAEEIRVALIA..N.RDERVHPETDVLLHMAYRVQNVKDIILPALADNKWV
PhiPA3_gp223       PGGTPF....GEEIRELLR..K.GDEQINQVSDILLHQAYRAQNVREIIKPALEAGKLV

                       120       130        140            150       160
                         .         .          .              .         .
RAY_gp311          VQDRTYFSTYAYQGMLYGQ.SPLVSKIH.....SGLDIMNADIVFVLDVDPEDILARVGR
PCH45_gp050        VVDRFWWTTLVYAD.......PSIENEVMRMHKLLQNDIRADFTFLCDIDYSTFIERRGF
Goslar_gp008       LSDRSIASTYVYQVNDA...N...VKLFDALLPNLPSIIMPSYYVYLDIDYPTYLRRFR.
PhiKZ_gp188        ITERFIASTYALNVVPFEETNPYLSKLFMDILQGTLHSIPEPATFFL..TAPEEVRKKRI
201phi2-1_gp287    ISDRFVFSTWCLNVQAHLETHPYLTDMLYGLMPYVLQGLPEPLTFIL..DTPREIRDQRA
PhiPA3_gp223       LSERFIISTYCLNVVPYLETNPELQDLFMGTLPFVTQGIPEPITFML..RLPEEERMKRA

                   170       180       190       200          210       220
                   .         .         .         .            .         .
RAY_gp311          REAKGNEDPTGNDRMDVMTDRQLKDLCYFYRGIEH....QMPACANIKQSKVIHLDGRQT
PCH45_gp050        .......RG.QCDEIEDSLFQRFDLMRSRYLDLADK....DSNSMVLSQSLS........
Goslar_gp008       ......VRHAKHQMFDNIDEATFNKRRYRYMDFMDYLGDAKTLCIDTSNTHS........
PhiKZ_gp188        .....EMRDRGLDYYESKGSSYFNKVDEGYGRLIN...Q..PSSIVVDTNRD........
201phi2-1_gp287    .....NSDERKKDRYESQPAAVHDRIEAAYEQLRS...S..PSCKFIDGTLP........
PhiPA3_gp223       .......DGRKLDRYEDMPADHVAKVSKAYRELQL......PSMVEVDATQS........

                       230                240                 250
                         .                  .                   .
RAY_gp311          QEQLLQDAIVHLT..........RFLDEKEREAND........LCRAIC...........
PCH45_gp050        VDEKVMVAYTRLRR.........RLSDDRRRTAVSTADNVRGELARA.............
Goslar_gp008       PVDVANRVYDNLLNAGFVE.........................................
PhiKZ_gp188        LDIIVNEIVESII..FLRDKQKNKQSSFDEQLAKTTTSDQFDDSVSSEIPWNDEQQEDTV
201phi2-1_gp287    IEEQVDIMFAYIK..GFKEQVLGQMAEAEER.......SGL..MKDEREQAR.DMAEQIR
PhiPA3_gp223       IEKIVDFILDVLT..QFDDRETQRLADEAQR.......KAL..MAEAEVPTPEEPVAKGS

RAY_gp311          ............................................................
PCH45_gp050        ............................................................
Goslar_gp008       ............................................................
PhiKZ_gp188        ATTDDTINFDLDAEISKYLDENIIPQLFE....G.DDRFLASGKVVAQKYC....QRVYD
201phi2-1_gp287    DELNADESWDLETEVAKYVETNITA.MADQLFQGADPEALAKETEFAKEFAANIARTTFE
PhiPA3_gp223       SEAEESQPFDLETALEAYAQTNIVPELFPRLTQEGDAEYQANAMKYWRELAKKIVAKIFK

RAY_gp311          ............................................................
PCH45_gp050        ............................................................
Goslar_gp008       ............................................................
PhiKZ_gp188        QLPDSEKHKMFLGSNKERLNQQIHSLLYYGHQLDAFIAENDLIGKTLWK...........
201phi2-1_gp287    R..TNGDRTVFHPTRVGQINQKVHSMLSWGFARDSWAKHFANGGDNGANQTQQA......
PhiPA3_gp223       Q..TGEDQSIFQPHRVGQLNSEIHSLFHYHARFEYMMDELRKDGAFGEDSTETGVTIEDP

RAY_gp311          ..
PCH45_gp050        ..
Goslar_gp008       ..
PhiKZ_gp188        ..
201phi2-1_gp287    ..
PhiPA3_gp223       AV
```

J

```
                      1        10        20        30         40
201phi2-1_gp347 .......MSIQRITFEGVLNWLSTKQSDKWHMVFQASQFD..LNDF....VDSVSFGHLT
PhiKZ_gp232     MLKVKS..LLTGHILRATYDWFLENNFRDILIVAHT.QLVPDLSEVALKH..AKEDHTIT
PhiPA3_gp270    MITLKK..SLTQCTLDSWYTFMAENSLSRFDCLIDIGYLDEPLRKILEKHPMYRGDGTIL
AH06_gp038      MSENKKIIPLVHFQIDAFRNFLIANGFTPYAVFALPKGLDPVLDQ....FIN..DGQII
RAY_gp039       MSEQKPLIPFLHFQIDAFRNYLVANDLTPYAVFTLPKGLDPVLDR....YADPASGQLI

                 50         60        70        80        90        100
201phi2-1_gp347 LNLSPNATRDM.ALYDEHIYFKICKHGIPQEMMIPYTALMIIQDPDDPSGSMP..WPYFL
PhiKZ_gp232     FNIHP.RCAKNFYISDEYISFNVTVNGVGVSIKLPLYAVLGVVTPIDDNSNAFFEMPLVD
PhiPA3_gp270    LNLSVNACKHINYRGDAFTG.EIGLQGRPTSLYIPYHAFVSLQIPLSETATVEASFPIYD
AH06_gp038      LNVGLTACGHYEITDDGYMVMEQRFNGKPHRSFVPVKYLLAMYARE..DVKHAMMFPDLA
RAY_gp039       LNLSPNSCGHYEITDDGYMVLHQRFSGKAHEAFLPVQHLLAMYARE..RQDQAMVFADIE

                   110        120           130        140        150        160
201phi2-1_gp347 DHGEDYTPDEDEELDQGT...VVKKSNVIELPNSGVKLELRIPTLEDYNNLNFDNVLQFP
PhiKZ_gp232     RYLNA...DTIRATLEGTVNNVVDKNPVL.............TTVGNVTEVNFKNKADT.
PhiPA3_gp270    RLVFD...AQNPTFV.............................................
AH06_gp038      ELDV........................................................
RAY_gp039       HTCD........................................................

                      170        180        190             200        210
201phi2-1_gp347 KKDADGELPSAEEIA.ELNQLMSDNGMDINLVEFKRN.....DKGEFEVSLSLNDNPVVE
PhiKZ_gp232     ...TNGKLVVRNTSENEHDQMI.ESAIN.RVTEFSNPELDAHIRVAVTVIKQNAPELIMD
PhiPA3_gp270    ...................................HPYAEA....GQQSLLTIAPEAVVK
AH06_gp038      ........................................S....G..............
RAY_gp039       .............................................D..............

                  220                230
201phi2-1_gp347 WDGKDKHLKQLSDN........LPKG...............LVSD...............
PhiKZ_gp232     WCERVIN..CTRN..................ENYQDNINVLMMELLSYPISNVVSESTVD
PhiPA3_gp270    WMHNEGHTECNVHNVGEFIKYVWPNGVPMEVQEYLVEETANMLRDHQMAVLQKQSAQAPV
AH06_gp038      ......................................ETEMLPDDQVVMQA.DVEAEAA
RAY_gp039       ......................................DVKFVEDETVPEV........D

                           240        250        260        270        280
201phi2-1_gp347 ...........DGIIDVVKLASKAQRLALAAPPTLQQRMAERGMSVIIGGAKPAEASMPF
PhiKZ_gp232     SGDNTSNSFDNDVNKMVEDFNNSSNSQVIINKP..SRPTGTPKLTVIKGGKK........
PhiPA3_gp270    PSNDDRIPFEEAEIKPLLEFPDLSANKVVLQQPVASKFKGKPTLTLIKGGKK........
AH06_gp038      PSNVTPL.RRG..............................PSLSIVK............
RAY_gp039       DTNVTALPFKK..............................PTLTVVK............

                 290        300        310        320        330        340
201phi2-1_gp347 IDEVYRAKRERREAIQKAADEALFKERALPAGTTLGEMLQSPGMTIRSDGSKGNSVFFPD
PhiKZ_gp232     ............................................................
PhiPA3_gp270    ............................................................
AH06_gp038      ............................................................
RAY_gp039       ............................................................

                 350        360        370
201phi2-1_gp347 LDVRKCYFHTRRIVRPEWLQVHEGGLK
PhiKZ_gp232     ...........................
PhiPA3_gp270    ...........................
AH06_gp038      ...........................
RAY_gp039       ...........................
```

K

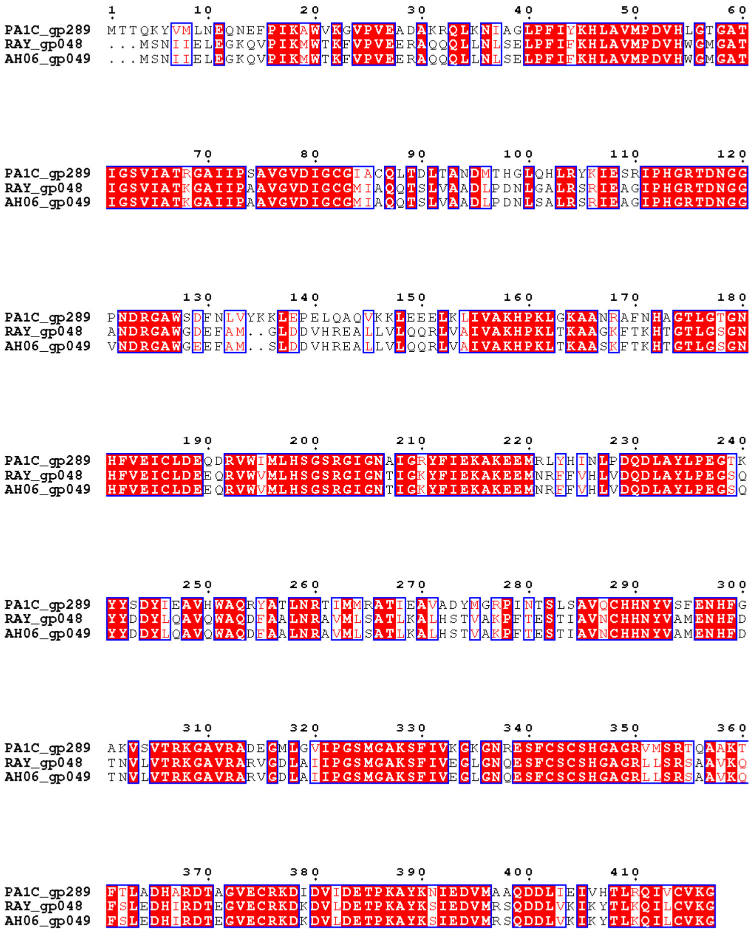
1 10 20 30 40 50 60
PA1C_gp289 MTTQKYVMLNEQNEFPIKAWVKGVPVEADAKRQLKNIAGLPFIYKHLAVMPDVHLGTGAT
RAY_gp048 ...MSNIIELEGKQVPIKMWTKFVPVEERAQQQLLNLSELPFIFKHLAVMPDVHWGMGAT
AH06_gp049 ...MSNIIELEGKQVPIKMWTKFVPVEERAQQQLLNLSELPFIFKHLAVMPDVHWGMGAT
70 80 90 100 110 120
PA1C_gp289 IGSVIATRGAIIPSAVGVDIGCGIACQLTDLTANDMTHGLQHLRYKIESRIPHGRTDNGG
RAY_gp048 IGSVIATKGAIIPAAVGVDIGCGMIAQQTSLVAADLPDNLGALRSRIEAGIPHGRTDNGG
AH06_gp049 IGSVIATKGAIIPAAVGVDIGCGMIAQQTSLVAADLPDNLSALRSRIEAGIPHGRTDNGG
130 140 150 160 170 180
PA1C_gp289 PNDRGAWSDFNLVYKKLEPELQAQVKKLEEELKLIVAKHPKLGKAANRAFNHAGTLGTGN
RAY_gp048 ANDRGAWGDEFAM..GLDDVHREALLVLQQRLVAIVAKHPKLTKAAGKFTKHTGTLGSGN
AH06_gp049 VNDRGAWGEEFAM..SLDDVHREALLVLQQRLVAIVAKHPKLTKAASKFTKHTGTLGSGN
190 200 210 220 230 240
PA1C_gp289 HFVEICLDEQDRVWIMLHSGSRGIGNAIGRYFIEKAKEEMRLYHINLPDQDLAYLPEGTK
RAY_gp048 HFVEICLDEEQRVWVMLHSGSRGIGNTIGKYFIEKAKEEMNRFFVHLVDQDLAYLPEGSQ
AH06_gp049 HFVEICLDEEQRVWVMLHSGSRGIGNTIGKYFIEKAKEEMNRFFVHLVDQDLAYLPEGSQ
250 260 270 280 290 300
PA1C_gp289 YYSDYIEAVHWAQRYATLNRTIMMRATIEAVADYMGRPINTSLSAVQCHHNYVSFENHFG
RAY_gp048 YYDDYLQAVQWAQDFAALNRAVMLSATLKALHSTVAKPFTESTIAVNCHHNYVAMENHFD
AH06_gp049 YYDDYLQAVQWAQDFAALNRAVMLSATLKALHSTVAKPFTESTIAVNCHHNYVAMENHFD
310 320 330 340 350 360
PA1C_gp289 AKVSVTRKGAVRADEGMLGVIPGSMGAKSFIVKGKGNRESFCSCSHGAGRVMSRTQAAKT
RAY_gp048 TNVLVTRKGAVRARVGDLAIIPGSMGAKSFIVEGLGNQESFCSCSHGAGRLLSRSAAVKQ
AH06_gp049 TNVLVTRKGAVRARVGDLAIIPGSMGAKSFIVEGLGNQESFCSCSHGAGRLLSRSAAVKQ
370 380 390 400 410
PA1C_gp289 FTLADHARDTAGVECRKDIDVIDETPKAYKNIEDVMAAQDDLIEIVHTLRQIVCVKG
RAY_gp048 FSLEDHIRDTEGVECRKDKDVLDETPKAYKSIEDVMRSQDDLVKIKYTLKQILCVKG
AH06_gp049 FSLEDHIRDTEGVECRKDKDVLDETPKAYKSIEDVMRSQDDLVKIKYTLKQILCVKG

L

```
                       1        10        20        30        40        50        60
Goslar_gp147   MALTTTIDNKELNPAETLMYAAIATLPNLDVEKLFKVHGDDLLNIIRDQIDPITPEEIKG
RAY_gp064      ............................................................
AH06_gp067     ............................................................

                                70        80        90       100       110       120
Goslar_gp147   NKELHEEIKRGEYYTYHGFMTLFQEDTSHLHEITLRHYLRCAIFQLMNHREETHKALAQS
RAY_gp064      ............................................................
AH06_gp067     ............................................................

                               130       140       150       160       170       180
Goslar_gp147   FQTHIFEAPYKPLDIVHLFYSISCCVNDQVMLLIQQNDRELIPLLSKLGIDLVAENITFS
RAY_gp064      ...........................................MITPLSLLY........
AH06_gp067     ............................................................

                               190       200       210       220       230       240
Goslar_gp147   QFIRETFLGAEPAPRYEGRFDLSTVEKPAFCLDVENLSLEKNGVLLEIGIVFFDLANPES
RAY_gp064      ..VKESYMI...................VVVVDTESTALDELAGILSLSAIRTNVADMSC
AH06_gp067     .......MI...................IGVVDAESTDLVDSAGLFTISGIIMNVTDLAD

                                         250       260       270       280
Goslar_gp147   I...........ANGPRIDIFPDVHGQIADGGRISESTVEWWQTKAPKEARDYCFQHSKV
RAY_gp064      IKHLIGVHQAGKTHDDIFHAVLNLNEQYVMGRTFLQSTQDWWMKKTTPAAKASVTGPTQ.
AH06_gp067     IEKQIADYKAGKRPEGCVHVVLNITEQYLLGRTFLASTQAWWVKKTTPAARAAILEQPT.

                    290       300       310       320       330       340
Goslar_gp147   MDYKEAIAEVNEFINTIREVFTDYYGKDT.FYYLARGETDWPQLEHWFRKAGFKPVCRYN
RAY_gp064      .SAKDALTAFVAFIEKAIEATINERGEKAKVQLYYRGDFDAKAIASLAKAVGVELPYIFN
AH06_gp067     .PLKEAMELYAAFIQKLIDLSKEERGPNALVNLYYRGDFDGRAIASLAKATGVQLPSGYR

                     350       360       370       380       390       400
Goslar_gp147   QVQDIRSMIAAYQDKPVSILGTKEYPDMGIPLIRHTAIGDAIMDAYDVAKARSMAASK..
RAY_gp064      QNKEIRTYIDAKLDTDIGYIPWLGYP...EGLGRHSSLDDVLIDGFEMAVAYRINHLEKF
AH06_gp067     SNKEIRTYIDAKLDTDIGYIPWLGYP...AGLERHNSLEDVLIDGFEMAVAWRINDMQKN

Goslar_gp147   ....LPK....................
RAY_gp064      DKKSVPGEIAKLKETYPVPEKTHAKSK
AH06_gp067     AAKAVEAEIAKLSKKYPPKENQHV...
```

M

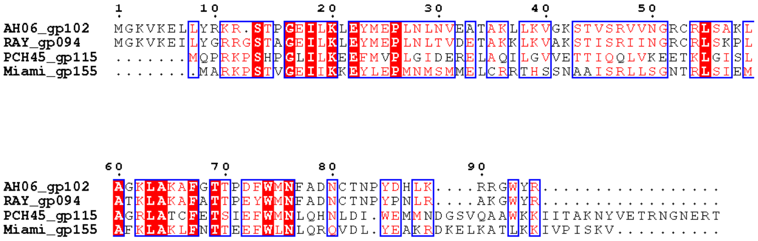

1 10 20 30 40 50
AH06_gp102 MGKVKELLYRKR.STPGEILKLEYMEPLNLNVEATAKLLKVGKSTVSRVVNGRCRLSAKL
RAY_gp094 MGKVKEILYGRRGSTAGEILKLEYMEPLNLTVDETAKKLKVAKSTISRIINGRCRLSKPL
PCH45_gp115 .......MQPRKPSHPGLILKEEFMVPLGIDERELAQILGVVETTIQQLVKEETKLGISL
Miami_gp155 ........MARKPSTVGEIIKKEYLEPMNMSMMELCRRTHSSNAAISRLLSGNTRLSIEM
60 70 80 90
AH06_gp102 AGKLAKAFGTTPDFWMNFADNCTNPYDHLK....RRGWYR.................
RAY_gp094 ATKLAKAFATTPEYWMNFADNCTNPYPNLR....AKGWYR.................
PCH45_gp115 AGRLATCFETSIEFWMNLQHNLDI.WEMMNDGSVQAAWKKIITAKNYVETRNGNERT
Miami_gp155 AFKLAKLFNTTEEFWLNLQRQVDL.YEAKRDKELKATLKKIVPISKV..........

N

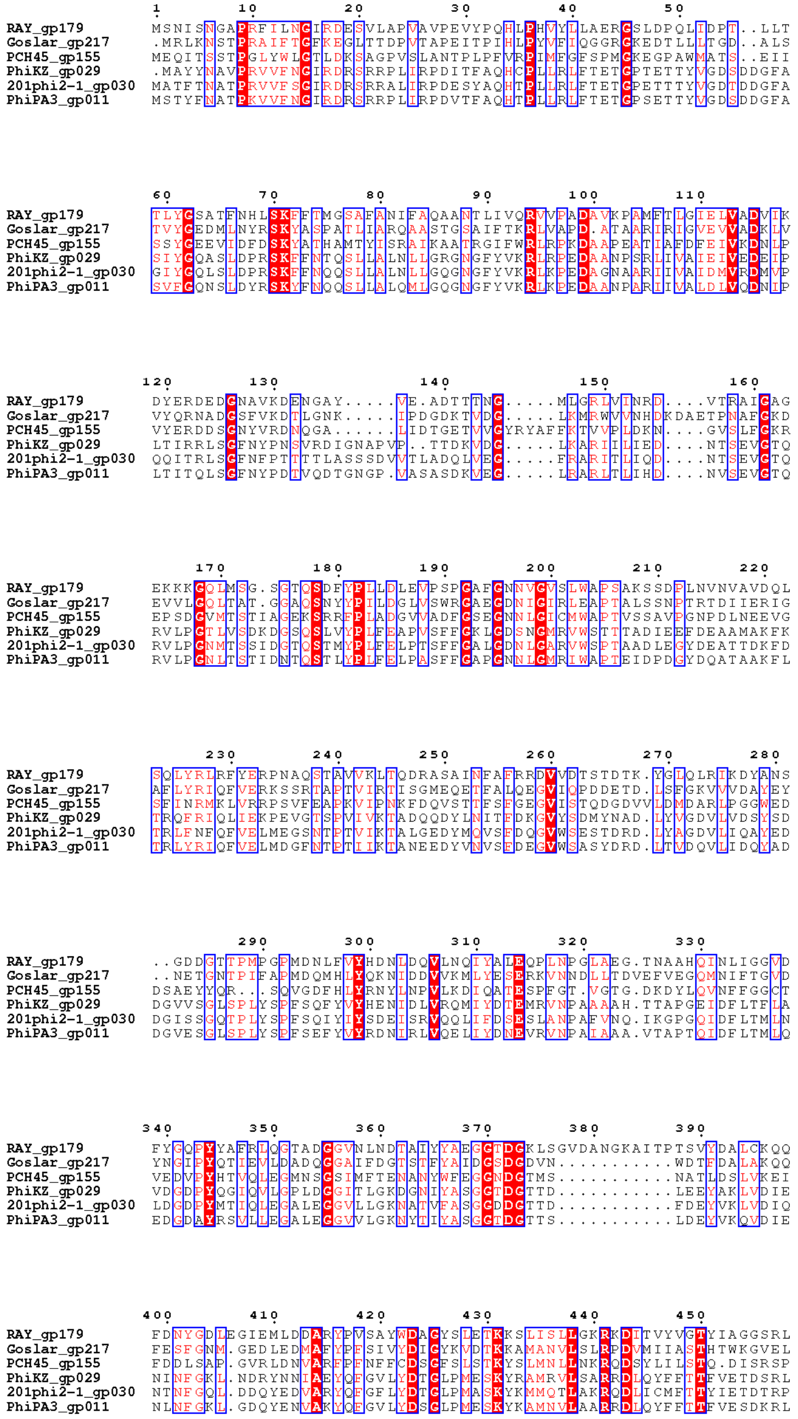
1 10 20 30 40 50
RAY_gp179 MSNISNGAPRFILNGIRDESVLAPVAVPEVYPQHLPHVYLLAERGSLDPQLIDPT..LLT
Goslar_gp217 .MRLKNSTPRAIFTGFKEGLTTDPVTAPEITPIHLPYVFIQGGRGKEDTLLLTGD..ALS
PCH45_gp155 MEQITSSTPGLYWLGTLDKSAGPVSLANTPLPFVRPIMFGFSPMGKEGPAWMATS..EII
PhiKZ_gp029 .MAYYNAVPRVVFNGIRDRSRRPLIRPDITFAQHCPLLRLFTETGPTETTYVGDSDDGFA
201phi2-1_gp030 MATFTNATPRVVFSGIRDRSRRALIRPDESYAQHTPLLRLFTETGPETTTYVGDTDDGFA
PhiPA3_gp011 MSTYFNATPKVVFNGIRDRSRRPLIRPDVTFAQHTPLLRLFTETGPSETTYVGDSDDGFA
60 70 80 90 100 110
RAY_gp179 TLYGSATFNHLSKEFTMGSAFANIFAQAANTLIVQRVVPADAVKPAMFTLGIELVADVIK
Goslar_gp217 TVYGEDMLNYRSKYASPATLIARQAASTGSAIFTKRLVAPD.ATAARIRIGVEVVADKLV
PCH45_gp155 SSYGEEVIDFDSKYATHAMTYISRAIKAATRGIFWRLRPKDAAPEATIAFDFEIVKDNLP
PhiKZ_gp029 SIYGQASLDPRSKFFNTQSLLALNLLGRGNGFYVKRLRPEDAANPSRLIVAIEIVEDEIP
201phi2-1_gp030 GIYGQLSLDPRSKFFNQQSLLALNLLGQGNGFYVKRLKPEDAGNAARIIVAIDMVRDMVP
PhiPA3_gp011 SVFGQNSLDYRSKYFNQQSLLALQMLGQGNGFYVKRLKPEDAANPARLIVALDLVQDNIP
120 130 140 150 160
RAY_gp179 DYERDEDGNAVKDENGAY.....VE.ADTTTNG.....MLGRLVINRD....VTRAIGAG
Goslar_gp217 VYQRNADGSFVKDTLGNK.....IPDGDKTVDG.....LKMRWVVNHDKDAETPNAFGKD
PCH45_gp155 VYERDDSGNYVRDNQGA......LIDTGETVVGYRYAFFKTVVPLDKN....GVSLFGKR
PhiKZ_gp029 LTIRRLSGFNYPNSVRDIGNAPVP..TTDKVDG.....LKARIILIED....NTSEVGTQ
201phi2-1_gp030 QQITRLSGFNFPTTTTLASSSDVVTLADQLVEG.....FRARITLIQD....NTSEVGTQ
PhiPA3_gp011 LTITQLSGFNYPDTVQDTGNGP.VASASDKVEG.....LRARLTLIHD....NVSEVGTQ
170 180 190 200 210 220
RAY_gp179 EKKKGQLMSG.SGTQSDFYPLLDLEVPSPGAFGNNVGVSLWAPSAKSSDPLNVNVAVDQL
Goslar_gp217 EVVLGQLTAT.GGAQSNYYPILDGLVSWRGAEGDNIGIRLEAPTALSSNPTRTDIIERIG
PCH45_gp155 EPSDGVMTSTIAGEKSRRFPLADGVVADFGSEGNNLGICMWAPTVSSAVPGNPDLNEEVG
PhiKZ_gp029 RVLPGTLVSDKDGSQSLVYPLFEAPVSFFGKLGDSNGMRVWSTTADIEEFDEAAMAKFK
201phi2-1_gp030 RVLPGNMTSSIDGTQSTMYPLFELPTSFFGALGDNLGARVWSPTAADLEGYDEATTDKFD
PhiPA3_gp011 RVLPGNLTSTIDNTQSTLYPLFELPASFFGAPGNNLGMRIWAPTEIDPDGYDQATAAKFL
230 240 250 260 270 280
RAY_gp179 SQLYRLRFYERPNAQSTAVVKLTQDRASAINFAFRRDVVDTSTDTK.YGLQLRIKDYANS
Goslar_gp217 AFLYRIQFVERKSSRTAPTVIRTISGMEQETFALQEGVIQPDDETD.LSFGKVVVDAYEY
PCH45_gp155 SFINRMKLVRRPSVFEAPKVIPNKFDQVSTTFSFGEGVISTQDGDVVLDMDARLPGGWED
PhiKZ_gp029 TRQFRIQLIEKPEVGTSPVIVKTADQQDYLNITFDKGVYSDMYNAD.LYVGDVLVDSYSD
201phi2-1_gp030 TRLFNFQFVELMEGSNTPTVIKTALGEDYMQVSFDQGVWSESTDRD.LYAGDVLIQAYED
PhiPA3_gp011 TRLYRIQFVELMDGFNTPTIIKTANEEDYVNVSFDEGVWSASYDRD.LTVDQVLIDQYAD
290 300 310 320 330
RAY_gp179 ..GDDGTTPMPGPMDNLFVYHDNLDQVLNQIYALEQPLNPGLAEG.TNAAHQINLIGGVD
Goslar_gp217 ..NETGNTPIFAPMDQMHLYQKNIDDVVKMLYESERKVNNDLLTDVEFVEGQMNIFTGVD
PCH45_gp155 DSAEYYQR...SQVGDFHLYRNYLNPVLKDIQATESPFGT.VGTG.DKDYLQVNFFGGCT
PhiKZ_gp029 DGVVSGLSPLYSPFSQFYVYHENIDLVRQMIYDTEMRVNPAAAAH.TTAPGEIDFLTFLA
201phi2-1_gp030 DGISSGQTPLYSPFSQIYIYSDEISRVQQLIFDSESLANPAFVNQ.IKGPGQIDFLTMLN
PhiPA3_gp011 DGVESGLSPLYSPFSEFYVYRDNIRLVQELIYDNEVRVNPAIAAA.VTAPTQIDFLTMLQ
340 350 360 370 380 390
RAY_gp179 FYGQPYYAFRLQGTADGGVNLNDTATYYAEGGTDGKLSGVDANGKAITPTSVYDALCKQQ
Goslar_gp217 YNGIPYQTIEVLDADQGGAIFDGTSTFYAIDGSDGDVN...........WDTFDALAKQQ
PCH45_gp155 VEDVPYHTVQLEGMNSGSIMFTENANYWFEGGNDGTMS...........NATLDSLVKEI
PhiKZ_gp029 VDGDPYQGIQVLGPLDGGITLGKDGNIYASGGTDGTTD...........LEEYAKLVDIE
201phi2-1_gp030 LDGDPYMTIQLEGALEGGVLLGKNATVFASGGDDGTTD...........FDEYVKLVDIQ
PhiPA3_gp011 EDGDAYRSVLLEGALEGGVVLGKNYTIYASGGTDGTTS...........LDEYVKQVDIE
400 410 420 430 440 450
RAY_gp179 FDNYGDLEGIEMLDDARYPVSAYWDAGYSLETKKSLISILGKRKDITVYVGTYIAGGSRL
Goslar_gp217 FESFGNM.GEDLEDMAFYPFSIVYDIGYKVDTKKAMANVLSLRPDVMIIASTHTWKGVEL
PCH45_gp155 FDDLSAP.GVRLDNVARFPFNFFCDSGFSLSTKYSLMNLLNKRQDSYLILSTQ.DISRSP
PhiKZ_gp029 NINFGKL.NDRYNNIAEYQFGVLYDTGLPMESKYRAMRVLSARRDLQYFFTTFVETDSRL
201phi2-1_gp030 NTNFGQL.DDQYEDVARYQFGFLYDTGLPMASKYKMMQTLAKRQDLICMFTTYIETDTRP
PhiPA3_gp011 NLNFGKI.GDQYENVAKYQEGVLYDSGLPMESKYKAMNVLAARRDLQYFFTTFVESDKRL

```
                     460       470       480       490       500       510
RAY_gp179           SS.LEQRSMAASLRSYAMLFPESSLYGTSTCRAVFFMQSGQLASEDSDDFYPHLLDHAYK
Goslar_gp217        NV.DEESSMGAMLRNYYRLHPESVLYNTPACRAAVFMQYGESIEAPSLGKVPLTLEVANK
PCH45_gp155         NDEESEESIASSILTRLQMFPDSVEYATPPFRGALVGHVGQLAGTSRRVSAVLAIDLAYK
PhiKZ_gp029         PDEATELSRVQQIITRLKAFPESTLYGTGVCRAMIVMQSGKLMDGTYRKYVPQLLDVAMS
201phi2-1_gp030     LTTGDEVSRTIALMTRLKAFPESTLYGTPVARAQIVLQSGKLMAGGYSKRVPQLLDTAMK
PhiPA3_gp011        PTASEEVSRVQALMTRLKAFPESTLYGTPVCRAMIVMQSGRLVGGGYNKFVPQLLDVAVM

                     520       530       540         550       560       570
RAY_gp179           RARYAGSGDGVLKNAFAYDAAENN..KVQLMKNMDALWEPNEVRNTEWGLGLNYAQTYNR
Goslar_gp217        LATYMGASDGVIRGTRI.DNNPGN..VVTTMRKINKTYRNATVRDRDWENGLNYVQTYNR
PCH45_gp155         LMRYGALDSGILSPDRAPDTSDNGNYIVTEVKGLNVIDKTWRNKRRLWSQSVVYVEDYDH
PhiKZ_gp029         WARYAGAGTGNLVPGMEMDVSPNN..RVTFVKDLNVKFFDDRVRAQAWANGATWSQSYDH
201phi2-1_gp030     WAIYAGAGNGILRPGFEMDVSPNN..QVTFVKGLNVPFFNARTASNIWQNGGTYSSTYDK
PhiPA3_gp011        WAKYAGAGTGVLRPGLEMDVDPNN..RVTMVKNLNVPFFNARTSANLWANGATYSSSYDH

                        580       590       600       610       620       630
RAY_gp179           R.QAYVPHVQTVYDTESSVLRDEIALTICVDIEKQCQIAYRMLGTDTRRTTAQTLSELNR
Goslar_gp217        N.SNFFPAWGTIYNNDRSVWRSLINVIIGCDLTRVCYRLWAETTGTTGMDETLFLKLMGE
PCH45_gp155         NNRLFYPGMQSFYTDQTSVLNSAIVGLCVAQLARYAWETWRDLTGNQKLKDAQLIERSDE
PhiKZ_gp029         R.SSYYPCLRSVMLDDTSVLLSPITVNICCVLIRLIHKVHAQFSGNATLTPEQLVERCDE
201phi2-1_gp030     R.RQYYPCQRSVYLDDTSVLLSPLTVNICCVIMRLIHRVHAKFAGNATLSKEQLVARCDQ
PhiPA3_gp011        R.SSYYPCLRSVYLDDTSVLLSPVTVNICCVIMRLIHKIHAKFSGNAFLTKEQLVERCDQ

                        640       650       660       670       680       690
RAY_gp179           ILENLTKDRYDNRVVISVNSFRTIADQNSRVRYSCEVSAAFNKGMYIGSFTVVSRNQEDL
Goslar_gp217        RFLEYTTGRYNDHVTVKGRFYLDKEDGYNGFSYHGDISLIGDPGMTVGTFTITGLRRNTE
PCH45_gp155         SIKNKSAGAMDDRMDVIPHSQLTRLDQNRGYSWTCPIDIGANGMRTVMSAYTVAYRREDL
PhiKZ_gp029         YILDLVRDMFGTRVNIIPRTEITPIDANNGTSWTCNVTVEANNPRTTLNFNLETVRIETP
201phi2-1_gp030     EILDQTRDLFGARVDITPQTEITAADDQNGFSWTCTVTVAANNPRTVMDFKLETVRRETV
PhiPA3_gp011        EILDRTRDMFGGRVDIIPRTEITPIDENNGFSWTCTVTVAANNPRTVLDFRLETVRRENV

RAY_gp179           V.....
Goslar_gp217        AAEAA.
PCH45_gp155         NG....
PhiKZ_gp029         PAQQ..
201phi2-1_gp030     TAGTVQ
PhiPA3_gp011        TADQQ.
```

O

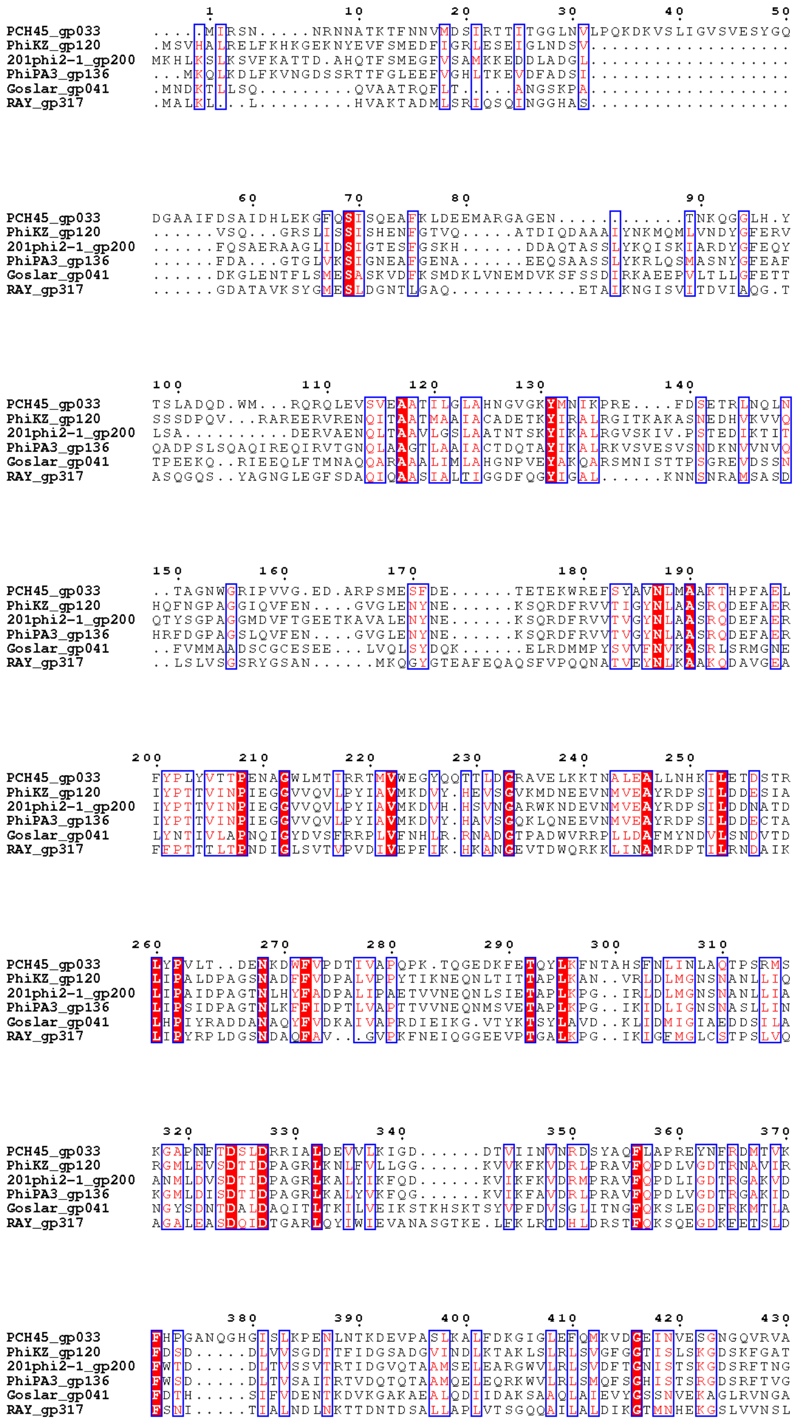
PCH45_gp033
PhiKZ_gp120
201phi2-1_gp200
PhiPA3_gp136
Goslar_gp041
RAY_gp317

```
                             440       450       460       470       480
PCH45_gp033        NAQ.FTTAFRLDKPEEKIALDDAEVKALVP..AKFSAEGFSLEARLTNLNQLERGKLVDS
PhiKZ_gp120        DT.YVDKVLNEDGQVM..DNADPAVKAILDQLTDLAVIGFELDTRFTNTNRRQRGHLLQT
201phi2-1_gp200    DA.TIAKILNADRREI..DITAGDGKVLADTITDLEIVGFELQVRFTNTNRRQRGHLLQT
PhiPA3_gp136       EV.SVDRITDEDRRNV..SLETGAGKTLVDSITDLEIVGFDLDTRFTNTNRRQRGHLLQT
Goslar_gp041       NA.ELYRLVDQDN.IE.QPRKSEEFKQLAEDL.SFKLVGYELRIRRNNSNRRERGKHLDF
RAY_gp317          TNPSIIRVYDTVAKED.ANTKSGALRDAIDAL.TITFKGYFVRANLRNSNFRTREQRVDR

                   490       500       510       520       530       540
PCH45_gp033        DVQKEGFIIPTLSPICIVKPSQMEDEKVY.PKIESLQFVYRTMLRNDAVTSLLNRADTLE
PhiKZ_gp120        RALQFRHPIPMHAPVTLPMDTMTDEGPGE..VVKALTVNTNIRNSNNAVKRMLNYLAQLR
201phi2-1_gp200    RALQFRHPIPMHAPVTLPLSTMEDEGPGE..VVKALTVNTNIRNSNNAVKTLLNYLAQLR
PhiPA3_gp136       RALQFRHPIPMHAPVTLPLSTMDEEGPGE..VVKALTVNTNIRNSNNAVKRLLNYLAQLK
Goslar_gp041       NMKQERYAIGLLAPFSIPRP..LDWEENA.ADLDVLIQAANARFDNLMVEKLFSYKDALK
RAY_gp317          QTSVYKYMVQLGAPITAIAPVTDAWGPKESIAVETLSNATYARNSAMAISRLLDYVETIR

                      550       560       570       580       590       600
PCH45_gp033        SY...LGNGITHGLESNLGMEGLGQYYYRPHFERIKINVAQELNNLTSANKLGDIQGLIV
PhiKZ_gp120        EV...VHNGYN..RPKFGIIEGALSAVMRPTYRYKELDLEKVIDTIKSKDRWDDVCAAIL
201phi2-1_gp200    EL...TGNGFN..RPVINVYEGALAAVMRPTYHYDTMHLPDMIDTLKSKDRWDDVCSAIL
PhiPA3_gp136       EV...TANGFD..RPQFGAVEGALSAVMRPTYRYKELHLPDVIDTLKSQDRWEDVCSAIL
Goslar_gp041       AV...ATSRFR..QFGDIQIEGFGRYLIRPWFEEINLDLEKSVQGTTTAERRAEVSAAIM
RAY_gp317          DAGVTNNGLWQ..RYDENSIEGVGKAVVDPWYGEGSFDISKVVNGVKSSQTRDDVSEKIL

                      610       620       630       640       650       660
PCH45_gp033        SRMNEVIYRVNKVTGYSAALEEQFPGSTPKPHVAIGTDLYLPQFLMIQGDDRTVAIGFDH
PhiKZ_gp120        NCVKAELFPAHRDSNIEAAFRVISGNQDETPMYLFCSDKEIANYLMTKGDDRTLGAYLKY
201phi2-1_gp200    NKVKGILFPAYRNSNIEAAFRVISGNQDERPIFLFCTDKEIANYLMTKGDDRTLGAYLEY
PhiPA3_gp136       NAVKGELFPAYRESNIEAAFRVISGNQDERPMFLFCSDKEIANYLMTKGDDRTLGAYLEY
Goslar_gp041       NVIREMAYRMYSESGYQPALQLLSSNADEKPRCVVATDNYIPQFLQTDGDFRTVGVSFNF
RAY_gp317          NILRTYAYRMNAESGYNVALQMLYN..EVQPTLVIATDNIIAQYLMEVGDLRTAGISFKV

                      670       680       690       700       710       720
PCH45_gp033        TIASISDLRMSNKIVMQFTLPGEKEPHPMQSGVCGMIPEYLANFQMIRDQRI.NTEMRLT
PhiKZ_gp120        DIVSTNNQLFDGKLVVIPTRAVQQENDILSWGQFFYVSTVIADLPITRGGHQVTREIAAI
201phi2-1_gp200    DIVSTNNELFDGKLVVVPTRKTPVENDILSFGQFFYVSTIIADLPITRGGNQITREIAAV
PhiPA3_gp136       DLVSTNNELFDSKLVVIPTRKNPQENDILSFGQFFYVSTVIADLPITRGGQQVTREIAAI
Goslar_gp041       VVVDNPNVMLRDKIFVTLSRGIQGSPDPLTFGMFLYVPELVSVAQVTRNGQV.SKETMVQ
RAY_gp317          HVATTNNIEMRGKIIMTLSRMKSGGLDLLTFGMHLWVPEIVATVNVMRNGTY.IEETMVQ

                       730       740       750       760                   770
PCH45_gp033        PRYRYFNFTPVMIVIEIEGLEEAVAQRTAMNVNEKAVTAFPG.........EPVAPTPPV
PhiKZ_gp120        PFNLHVNNIPFALEFKITGFQKVMGETQFNGKLADLKP......................
201phi2-1_gp200    PFNLHINNVPFALEFDVTGFEQVMGHSMWNKPVVDALGA.....................
PhiPA3_gp136       PFNLHVNNIPFALEFKITGFQEVMGESQWNKTVASLKP......................
Goslar_gp041       PRCRFVNNCPLLGLINVVNLRKVYTENIKLPVQTN.........................
RAY_gp317          PRNLHVVVCPIVANITITGLEEAYTGKITVPVDIVSEPESAGAQDLGGLNTTPATTTNPA

PCH45_gp033        TDGE
PhiKZ_gp120        ....
201phi2-1_gp200    ....
PhiPA3_gp136       ....
Goslar_gp041       ....
RAY_gp317          A...
```

P

i

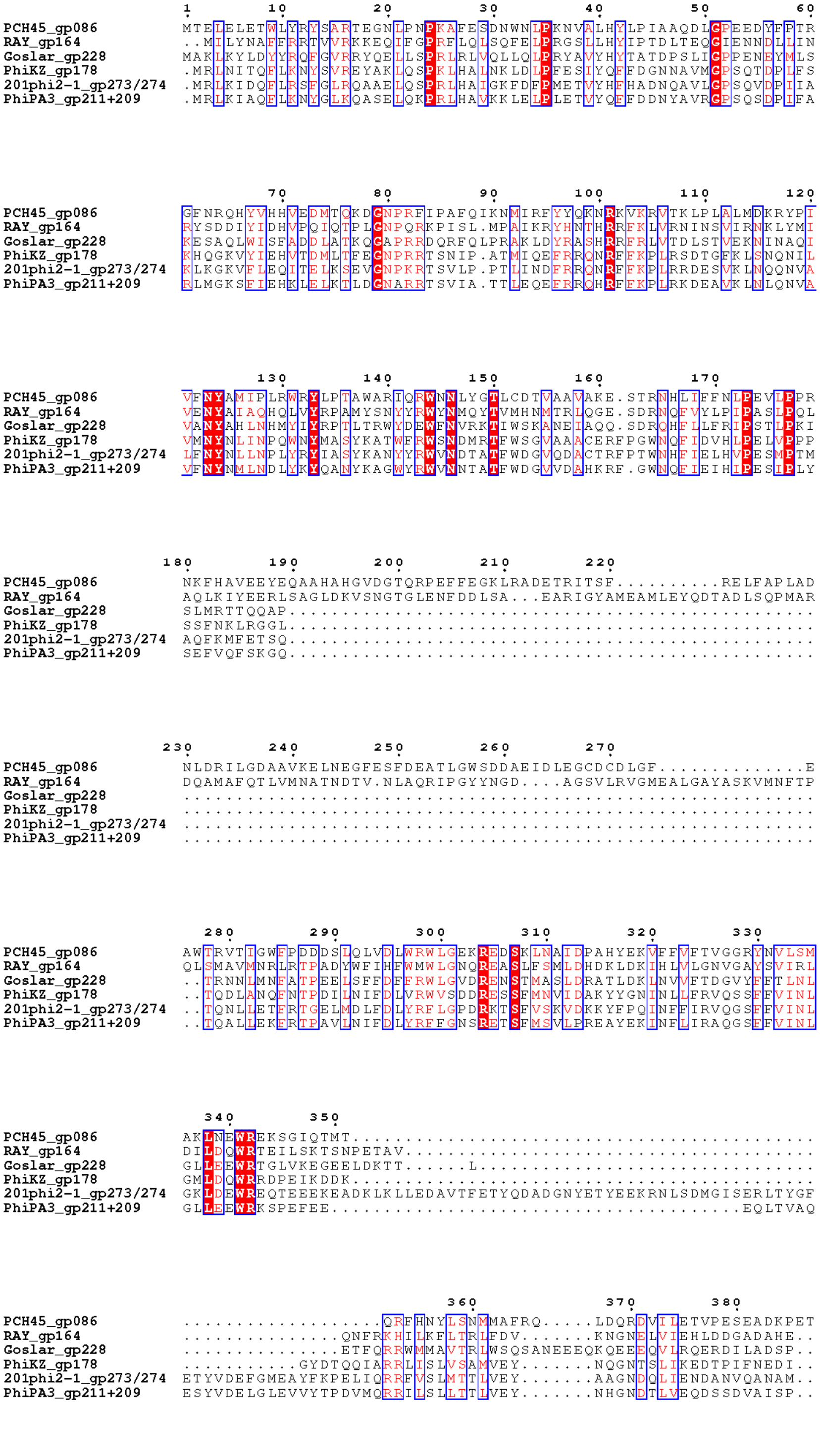
PCH45_gp086
RAY_gp164
Goslar_gp228
PhiKZ_gp178
201phi2-1_gp273/274
PhiPA3_gp211+209

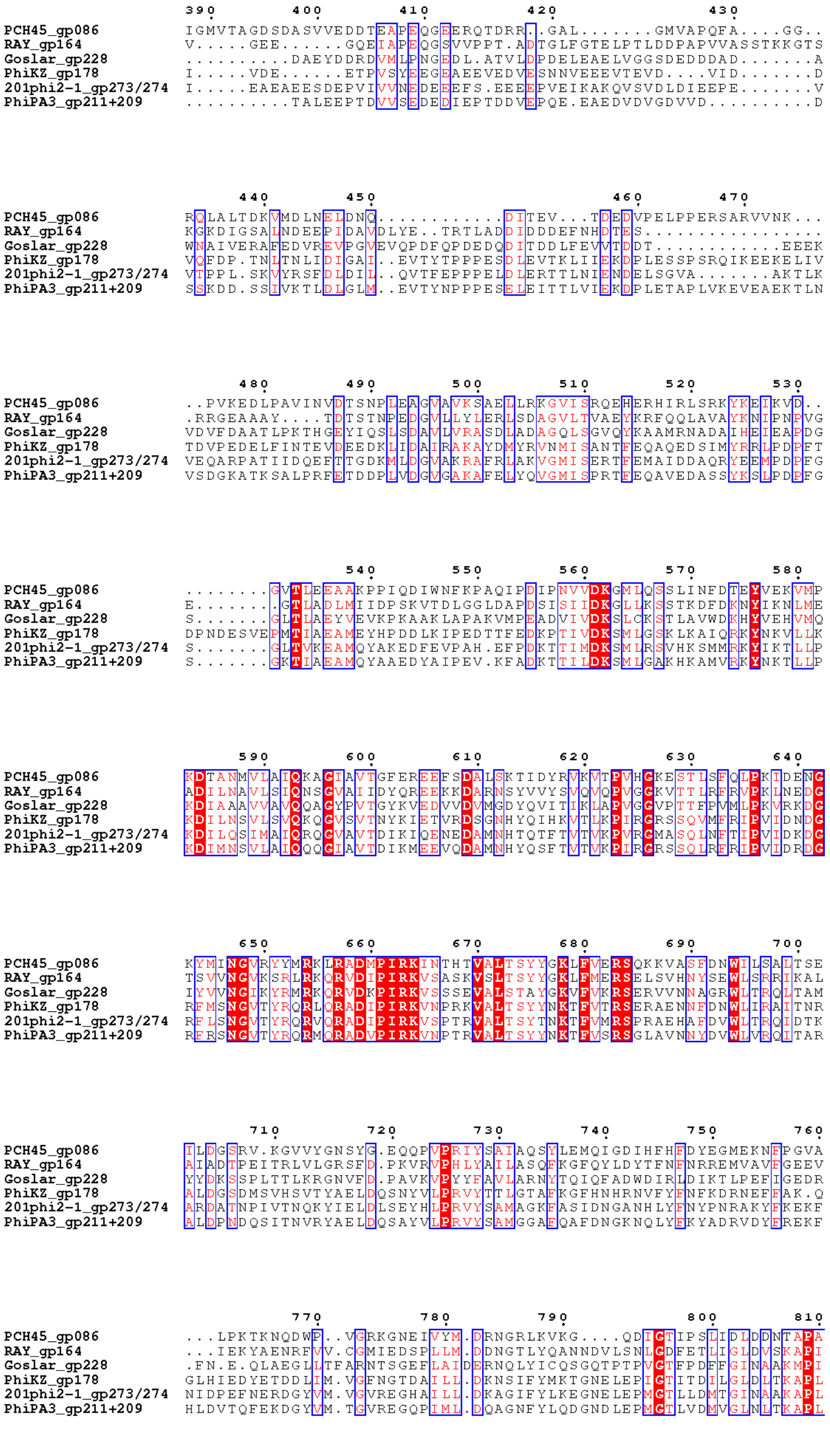
PCH45_gp086
RAY_gp164
Goslar_gp228
PhiKZ_gp178
201phi2-1_gp273/274
PhiPA3_gp211+209

```
                             820       830       840       850       860       870
PCH45_gp086         EIVVLRVMGHEIPLGIVLAYMFGLNKLANRLYGSPVSTESYSEYYSEQYGYISFEHDNTV
RAY_gp164           ETVVLGVFRQKVPIGIILAYYYGLGAMIEQFNL...........................
Goslar_gp228        EAADLEFMGVRIPVGVVLSYLGGLTPLLEHLKA...........................
PhiKZ_gp178         EAILMSIGGKELPLGFILAYHHGLNNLLKKLNV...........................
201phi2-1_gp273/274 EAVDVKISGKDIPIGFVLGYQLGLTNLLDLLGV...........................
PhiPA3_gp211+209    EAIVMSVSNKEIPLAFILGFHGGLTNLLNKLGV...........................

                             880       890       900       910       920       930
PCH45_gp086         PNGVYPRRRIKGAKKDTAPNEFEIVFADEVWVLPKDQSYASLIFASLNQWKKQLRTIQVA
RAY_gp164           ....QVRTANRGERYQLTKDERAILFNDEVLIFNRTDRLGAMLLNGFNSYAKEVSRFSRY
Goslar_gp228        ....NYRTVPRGSRMGLAADEFAVSFDDESLIFSKDDRMTTLLLGGFNEYRNY.IGYARG
PhiKZ_gp178         ....NYQRHQRGTRIVVNSTDYTLAFADEILVFDRADYKAMLVLSGLKRYHKSLRRYSVY
201phi2-1_gp273/274 ....SYQAHSRGKSIVVTGDDYTLAFNDEILVFPRDDYRSMLVLGGLRRYHKVLRNYSRY
PhiPA3_gp211+209    ....KYDKHQRGTRIQVSQDDYTLAFADEILVFSRQDYKAQLVLAGFKRYHQSLKKFSRY

                             940       950       960       970       980       990
PCH45_gp086         ELNDKAYYTPMFMANGLGNRHLNEIGLIEDMFLDPIAIENLTMMKEPTNMRDLFIRAADM
RAY_gp164           DFDRKSVYLNVLSSAGMGVRWLREFDLMREMYIDPITREELLKMNAPVKFDLLLVHGTKM
Goslar_gp228        AFDNRDIYFNVVDGAGLKVRHLRELELMERLFVDPVSRTLLQNMGEPTDFAMLLLRAVEL
PhiKZ_gp178         DFNKRDVYFRVLEEAGLSNRFTRDIDTLFSAWVDPITEGLLKEMGEPTTFEGLLYRSVEL
201phi2-1_gp273/274 DFDKKDVYHRVLIEQDMGNRFIREIDFLFEAFVDPITEGMLIEMGEPTDFQGLLFRSVEL
PhiPA3_gp211+209    DFDRRDVYYRVLEEAGLSSRYAKEITHLFNSWVDPITKGILEEMGEPTDFEGLLYRSVEL

                            1000      1010      1020      1030      1040      1050
PCH45_gp086         LVDDNYKSDLDTSLMLVKGYERMSGAVYKAIVDSVRNYRNKPVSVKTSVDLNPFEVLTSI
RAY_gp164           LLNDQHIRETSFREQRVRGAERISGAIYLELVRSMRIQKARATSSKVGLELHPDAVWFDL
Goslar_gp228        LLDDWAPNTNDVNYQRFKTYDKFAELVYKDLVGAIRQNENSSQGNRQKISISPVSVWNQI
PhiKZ_gp178         LTNDWSPAEVDGAYMRYRGYERMAGAIFNELNRAVRVFNMRNGGSVQTVELDPHVIWRKI
201phi2-1_gp273/274 LMEDWSPGEVDGQYMRYRGYERIAGQIFSSLSKAIKRYNAREGSTDVKVILDRHEVWTKL
PhiPA3_gp211+209    LMNDWSPGEVDGAYMRYRGYERMAGAIYKSLNQAAKVYNNREGSADQQIILDQHEIWRKI

                            1060      1070      1080      1090      1100
PCH45_gp086         QKDPSVSLVEDSNVIHNLKEKENMTYSGTGGRSKVAM.TRRTRAFHKTDLGVRSEAGVDS
RAY_gp164           MQDTSAIPVEECNPIHNIKDMEVITYGGSGGRTSRSM.TKPTREYIKDNMGMISEASVDN
Goslar_gp228        LDDSASIVIDETNPMLDIKQGEEATMTGIGGRSKTSLRRARDREYDKSDVGIVSEGTKDS
PhiKZ_gp178         VQDPTVATIEDSNPIANIREQEAMTYRGDGGRGPTSM.VARTRIYGEADVGVVSESTVDS
201phi2-1_gp273/274 .NDPTVATIEDSNPIANLREQEAMTYRGDGGRAAVSM.VARTRIYGESDLGVVSESTVDS
PhiPA3_gp211+209    VQDPTVATIEDSNPIANLREQEAMTYRGDGGHGPTSM.VARTRIYGEADVGVVSESTVDS

                 1110      1120      1130      1140      1150
PCH45_gp086         GDVGINMFMPPDPNITSLRGTVQTVDPEKVSPTQLVSTSMLMSPFAEYDD..........
RAY_gp164           ADVGYTAYMSMDPLMTDLRGNTQAATDD.TSPTHIVSTSALLAPAADRDS..........
Goslar_gp228        GDVGISAYLAPNANIVSVYGETRPFDEKTDGISSVMSTSALAVPAVDRDD..........
PhiKZ_gp178         GDVGVIAYLVPDANFVNMRGVTRMFDPKTDGPARLLSTSALLGVATEHDD..........
201phi2-1_gp273/274 GDVGIIAYMTPDANFTSMRGLTRLYDPSKDGKSKLLSTSALLGVGIAHDD..........
PhiPA3_gp211+209    GDVGVIAYLSPDANFVSMRGTTRMFDKETDGHAKMLSTSALLAVGSMNDDCYCLLNVCEV

                      1160      1170      1180      1190      1200      1210
PCH45_gp086         .....GKRVNFINIQQDHVVAIAESQEMAVRTGYDQVVAHRTDDLFSVTAKDDGKVVSVT
RAY_gp164           .....PNRTNFISIQHSQAMYADGYQTTPYRTGAERMIAHRASKLFAYAAEEDGVITEIS
Goslar_gp228        .....GKRLVFVGIQHNRMIAAEGYDVQPWRTGGETVIAHRVGKLFAAAAQGDGVVKKVT
PhiKZ_gp178         .....PKRINFISIQQQQGIYADGYTVTPVRTGYEQIIAQRTSSIFAVAAEQDGEVVAID
201phi2-1_gp273/274 .....MKRIGFASIQQQQGLYADGYDLSPVRTGYEQVVGQRTSSIFATAAEQDGQVVALD
PhiPA3_gp211+209    YTPMLMKRVGFISIQQQQGIYADGYEVTPVRTGYEQIVAQRTSSIFATAAEQDGEVISVD

                      1220        1230      1240      1250      1260      1270
PCH45_gp086         DRVITVQYKNGKQ..KAFQIGRRFGVVTGHVVPHNIVPRMKAGDSFKKGAVICHNEGFYT
RAY_gp164           AKHLMAQYKDRRV...GVELGIRYGNASGTTYVHPIITDMAVGQAFKKGDILCWNRNYFE
Goslar_gp228        KTALTIQYDDPELGEEVFQLGTIIGKGPGKYYPHDLITDYKVGDRVKDQDVVVFHRLYFK
PhiKZ_gp178         EYGIQVKYADGNV..FGYQLGTVHGTAAGVNYPHILVTSLKKGDKFKRGDTITYNKRYFS
201phi2-1_gp273/274 KHGITVQYADGEV..ESMPLGTIHGSAAGVVYPHELVTKFKKGDKFKRGDTITYNRKYFK
PhiPA3_gp211+209    EHGITVKYADGEI..VSSPLGIVHGTAAGVNYPHDLVTDLKKGDKFKHGDTIAYNRKYFT
```

```
                          1280      1290      1300      1310      1320      1330
PCH45_gp086               PDRRDPTSSALKQGCYPYTVLIEKNETWEDSSLMSPELAYKMRANVTHIRTLFVRFDNAI
RAY_gp164                 RDFMEPMQCSWKAGAMVRVALMEEEFTFEDSSVISQTTAKRLGTRTAKPIAITVDFTQEV
Goslar_gp228              RDWWNPGQVVWRAGVLSLVGILDIPETHEDSSYIFDNLADRLVTPSIKSRTLIVDAHLNI
PhiKZ_gp178               EDRYTPGQVLWKAGCMAVVAFDDNLDTLEDGSVISEDLAKRLNTQTTAIKNIDVRFDQTI
201phi2-1_gp273/274       EDRYNPDQVSLMPGVIGVVAFDDNLDTLEDGSVVSESIAKKLNTQTTDVKNVIVRFDQHV
PhiPA3_gp211+209          ADRYAPGQVLWKAGCMATVAFCDNLDTLEDGSTISKELAVRLQTQTTDIKNITVRFDQHV

                          1340      1350      1360      1370      1380
PCH45_gp086               HNLVSVGEEVDIDTVLAVIEDKVSAGVG...SDNLDALNLLSRPTPRANNSGKVEEIQVI
RAY_gp164                 RNLLPVGTQVDPETILCTLEDPVTANLGQFDDESFDSLRILANKNPKAKVTGEIARIEVT
Goslar_gp228              RGLVKIGDHVEPRTILATLEEPIATDLSDESQEAVEALRRMSQYNPRADVHGEVVQIDVL
PhiKZ_gp178               RDMVKVGDHVDLNSILCIIEDPETAAHSLYDEASIETLRKLSAYSPRAKLVGTVSKIECF
201phi2-1_gp273/274       SELVQPGDHLDLSSILCIIEDPETAAGSLFDDASIETLKRLSSSTPTAKVVGTVSKIECF
PhiPA3_gp211+209          SELVKRGDHVDLNSILCIIEDPETAENTLFGDAAIETLRRLAAHTPRAKVVGTVSKIECY

                       1390      1400      1410      1420      1430      1440
PCH45_gp086               YFGDKDDMSPSVRKVADKYDRVRGQLARELNNGDA.TTGEITTPARVDGISVDIDMMAIR
RAY_gp164                 YRGSVDDMSESLGLIANRSDRERTKLNRQLGNNEA.NTGESLTPIRVQGTPLEANQAIIE
Goslar_gp228              YRAQIEDMSETMAALVGDADKARAKRVKELKLNEATTGRINNSNVRIDGNPLGENQIAIR
PhiKZ_gp178               YHGEIDDMTPSLQALANTSDKQRAERAKSLKEPT..FTGQVDANYRVKGRALEYDHMIIR
201phi2-1_gp273/274       YHGDIEDMSDNLRELAEASDKARAEIAQQTGRNA..YSGEVDSSFRIKGQILEPDSMVIR
PhiPA3_gp211+209          YHGDIEDMSENLQAIARASDKQRADIAKSLNQPV..FTGEVDSSFRVKGQILEPDHMVIR

                        1450      1460      1470      1480      1490      1500
PCH45_gp086               IFITEERIMSGGDKLVLSNQLKSVSAGEQRGPTYTASELWPGSGVRKIDLEFSYRAEEAR
RAY_gp164                 VYVITPMPTLSGDKGVFANQMKSTFGSIMPDGITTA.......SGKRLDAKFSNKSIANR
Goslar_gp228              IFMRYDLGAGMGDKGTFAAQAKSVVSSVGHGVNKTE.......DGHTLDAIFSGTSIANR
PhiKZ_gp178               VYIDHDIPCGVGDKGVVANQMKTVFSRVMTGRNETE.......DGRNIDLLFGNTSVEER
201phi2-1_gp273/274       IYIDHDIPFGTGDKAVLCNQMKTVISRVMTGTNTLE.......DGTPIDIIFGNTSVEER
PhiPA3_gp211+209          IYIDHDISCGVGDKGVLGNQMKTVISRVMEGRNETE.......DGTPIDLIFGNTSVEER

                        1510      1520      1530
PCH45_gp086               IVHSSIMAGMSIICQEFIGFSMVQAYDS.......
RAY_gp164                 MVTSPYVMGTTNVLLDTITRKACAMYKRLKG....
Goslar_gp228              IIGSPEVVGTVNLGLLYATERAVDIYFNDAK....
PhiKZ_gp178               MVLSPKIIATTTGILWAAGKHLVAVYRGNTNAKAK
201phi2-1_gp273/274       MVMSPKLIATTSILLKALSKRVAGVYRGTVNAKAK
PhiPA3_gp211+209          MVMSPKLISTTSILLRALSKHIAGVYRGTTNAKAK
```

ii

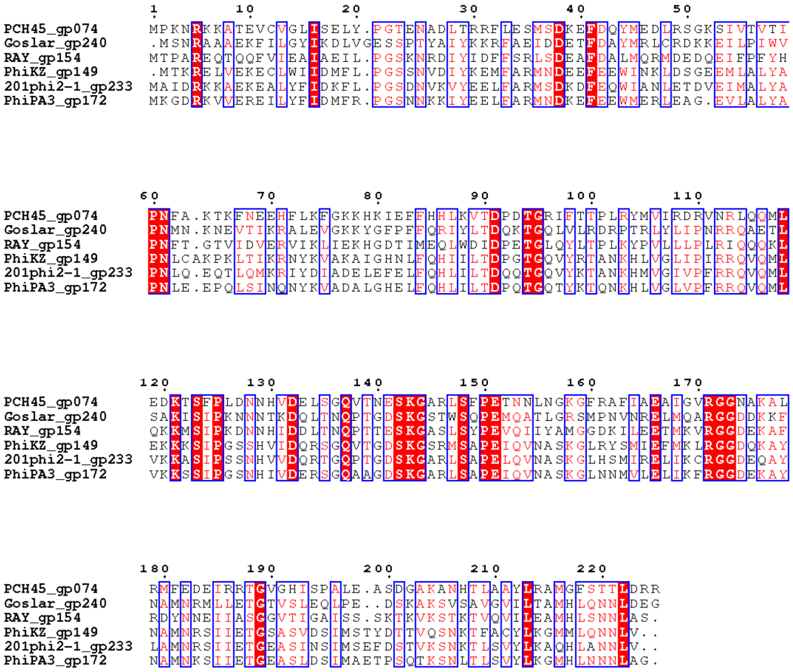
PCH45_gp074
Goslar_gp240
RAY_gp154
PhiKZ_gp149
201phi2-1_gp233
PhiPA3_gp172

iii

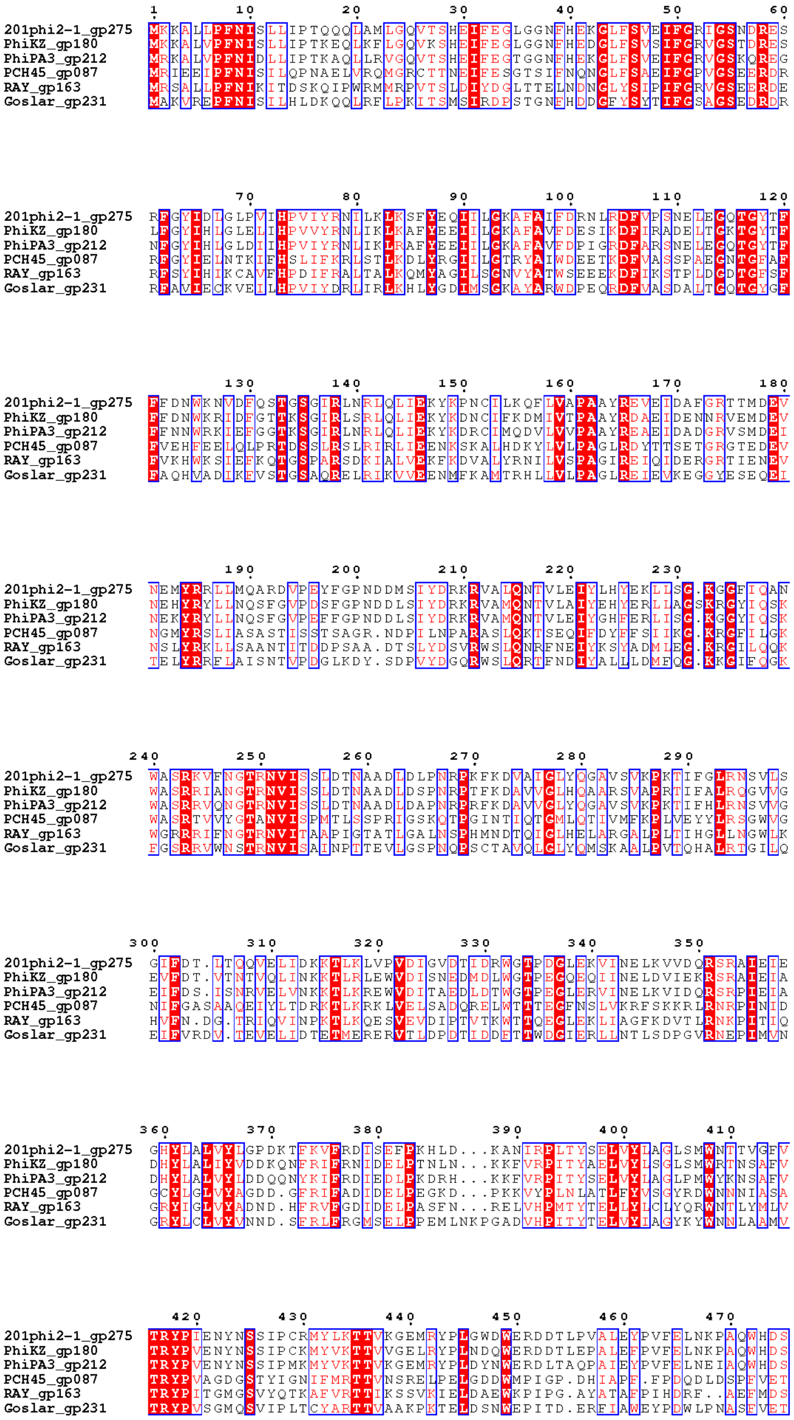
201phi2-1_gp275
PhiKZ_gp180
PhiPA3_gp212
PCH45_gp087
RAY_gp163
Goslar_gp231

```
                       480       490         500        510        520        530
201phi2-1_gp275   ISLPPATLAPLTADFDGDTT.SWIACYSEESIAECNKFFKSRLAYIRAGGGIAFSLNIHT
PhiKZ_gp180       VSISPSILAPLGAD..............................................
PhiPA3_gp212      TSVSPAVLAPLTADFDGDTV.SFNAVYSKEAIEESDKFFKSRLAYIKAGGGLAFSINIHT
PCH45_gp087       MSPHTARIKAQGGDFDGDRESC.IGAMSKEARDEYQRLKSKRDWYVGADNKLVLSAANDN
RAY_gp163         LSVHPSRLGLLGGDYDGD.TMSATILLGDDVLRECAMLLSRRESYITGRGEFLIDVTNET
Goslar_gp231      TIVPAPYLAGLGGDQGKANKMPVFWQI.................................

                        540        550
201phi2-1_gp275   LNLTLRYMTGDPIPRK
PhiKZ_gp180       ................
PhiPA3_gp212      LNLTLRYMTGDPVARD
PCH45_gp087       IELLLLQITGGPTK..
RAY_gp163         LDFTLKALTS......
Goslar_gp231      ................
```

iv

```
                  1         10         20         30         40
RAY_gp270         ..MNIRDYLIMAFRAGAYKQRQWVLSVMSITQTPDGKSVADS.................
Goslar_gp078      ..MTPREYFLYAMREGHYRYLEWINDAFAFTEGGAE..................K...
PCH45_gp003       ..MDRIQYLKHAVKAGACYYKTWLIDSMGIVDLPPKPETVDATINPSPGLRKDEPPRYPH
201phi2-1_gp139   MLMKIRDYFLMALKDGMGRKRIWMNSLVSVIYNN...........DAT......ADYN.
PhiPA3_gp077      ..MKLRDYFLLGLNKGLGKKRVWMNVLFNIVYNA...........NEG......PEYT.
PhiKZ_gp080       ..MKLRDYFLMGINAGLDKKRAWVNVLFNIVYNP...........NDG......GEYL.

                     50         60         70         80         90
RAY_gp270         .MIPYQAHRINIGATLDWAYWDDFNKTFVPIEGVDVNFPLFRPEDQYTLQPGDFANVTE.
Goslar_gp078      ....YQPKLRRQGNVV.TVTIDGEEHQW...ETEPTKPLLSFWEPLELAPGDLPSVKN.
PCH45_gp003       EEHPYQLFDFK.GTPV.FLNPT..TNEWEPVDGYKKGAPFFRFKEEINLEPGDIANVKE.
201phi2-1_gp139   ....YKIHYEG.DKAY.FFKPGT..DTPIYLEGFVPGEPFITFRDEFILQPGELVNYPEG
PhiPA3_gp077      ....LKPYFKD.DQMY.FYKEGDSNDVIEYIEDYIPNRAPLHFRDEFILQPGEIANYKGT
PhiKZ_gp080       ....YKPYFKD.GKMF.FYKEGDSNDEVVYIDDYIEGKQPLAFRDEFILQPGEIENYKGP

                  100        110        120        130        140        150
RAY_gp270         .EVTTTFGRILANHYYFIDVVGERVPFQNVEFNRGMLEKIYKARVLEDAQDLRR....SD
Goslar_gp078      .TTTTTFGEALFNMYVLYDAFGIQIEYQAGRLTSGTLESLIAPRVADDPEEGIPDPNERP
PCH45_gp003       .PITTWIGNLLVNQCVLCYPFGDLIPFQMGQFSIKKVEAIIASKLESLPDVGQ....ARD
201phi2-1_gp139   VPLRTCYGNVFFNQLCLVEPFGSLIPFATGTIVPSKFEDFILERMIDDREDGNVT.EMAP
PhiPA3_gp077      EPLRTTYGNVFANHLILVEAFGDLFPFQAGKFDPAKLEDQILERMIDDREDGDVT.TMAG
PhiKZ_gp080       GALRTTYGNVFVNHLVLCLPFGDLFPFQSGYFDIGKVEKEILNRMIDDPIDDDNP.PRAT

                      160        170        180        190        200        210
RAY_gp270         PFYLTVDEFK.RSVDNGFALGGLSKLCVPTACPETMYPPKFIIEMRDRLFEE.HKDELNN
Goslar_gp078      IGPLYASEVK.KFTNNISALTAINRLIVPSMSDKALTVDDDIIAYRDKELARLQEEGKFT
PCH45_gp003       ETKIYVDTLENYYYDAAYSLSGWTQLAVPAASPHTLITDPKIRKRRIELLNE.YKDQLDD
201phi2-1_gp139   DGKLYVWQYL.MFCDHCLAIPGYADGLVTSTTRKSLLSSPEWPAIRSTWIEK.NKHRLND
PhiPA3_gp077      DGKLYVWQYL.KFADACLAIPGYADSLVTSVTKKSLTSSPERDAVREKWVKD.NAHRLHD
PhiKZ_gp080       DGKLYVWQYR.MFADHCLALPAYASTLVTSITEKSMTSAPDRDKLRKELEEK.YKDRLDD

                       220        230        240         250        260
RAY_gp270         PVVMGRIQDELLKHYREFLMSTPSAKFFV.IK..KTIDQAFNNMFLTGGIAGAFGG...N
Goslar_gp078      PEELIKLEATLTKMDKESFKGDESEGFYINPG..KSFGNTRKKTHIMMGVEADFVDPGKR
PCH45_gp003       PAIIAKIMQELIDMDVAFQADDPEGGYLQ.PG..KSFDVVRAKAFLMYGIERDFNDPNKI
201phi2-1_gp139   PAAVAELSVIAAQVDAEWLKDDPAWTFYS.AK..KKLEGARRKVHYMFGGESPFSDGTTV
PhiPA3_gp077      PAAIAELGVLMGKVDDEYLEGDEANEFYK.SK..KKLAGARRKVHYFFGGESPFSDGTTV
PhiKZ_gp080       PAVIAEMGNALIKLDEDYLKDDPSYEFYD.TKHSKLFGGVRKKVFGMFGGEAPFQDGTSV

                     270        280        290        300        310        320
RAY_gp270         TVITNSLYEGWNLKNFPALVNGSIEASFDRGAATADGGEKVKMLIRATQNIRITDDDCGT
Goslar_gp078      NVITHSLAEGMRVDHWAEYNNSARAGSFSRGALTALAGQTAKESQRATQNLKITMDDCGT
PCH45_gp003       VLIDRPLSEGWDITKLPYMVNALIEGSFYRGAMTALGGEGAKFIQRFFLNTNIIEEDCGV
201phi2-1_gp139   EFISKSLEEGIDTDHMPVMNNSLRFGSYNRGSQTALGGESTKTIYRMMGTSRIIEQDCET
PhiPA3_gp077      TFIKKSLEEGIDTDFMPVMNNSLRFGSYNRGAQTAMGGESTKTIYRMVGTVRIIEPDCKT
PhiKZ_gp080       EYIGKSLEEGIDTDHMPVMNNSLRYGSYNRGAQTALGGESTKTIYRMVGTVRIIMEDCNT

                     330        340        350        360        370        380
RAY_gp270         QLGVPWVIED.AGKARFI.NSYILDKGVTILLTPENIDKFVGKPVMTRSPSFCKKSH...
Goslar_gp078      QEHILFTVNRDHIKDKLLVGRYMIDGAALKLIDAELLASLEGQTIKLRDPQRCKAGP...
PCH45_gp003       KYGKTTILKP.KDKDDYLGASVVGPGGSRIELTEENYASYAGKILQVFSPQYCKTKR...
201phi2-1_gp139   WLGVPTLVTK.LNAKGLVGYSYVVDG.KSIEITKENIDGLIGSVIAIRGPMVCKAGRDRA
PhiPA3_gp077      WIGIPTMVDD.FNAKTLIGYSYVDKG.RSILIEKDDLESLKGRIIDIRGPMTCKSGRGE.
PhiKZ_gp080       WIGIPTNVHQ.FNKKDFIGLSYVKNG.KSILIEETNIDELVGTEIELRGPMTCKAGGDPA

                            390        400        410        420        430
RAY_gp270         .......GDSCKFCVGAHNATNPRGMSSGTSKIGSTLMLLSMKAMHKGSKIDLIEYNLDE
Goslar_gp078      .......LEYCATCCGNAMAPTPDAIGAEITGVNNVFMNTMMKAMHNAS.VSLAPYDPEL
PCH45_gp003       .......PNFCVKCLGARYRGKPNSPAALASVIGSVLMYIFMKKMHGVA.LKTKKWDWRA
201phi2-1_gp139   RGTIGKGKNICAVCAGKDLAENPYGIPAAVAGVGGRFLMVFMSKMHSST.LRTVEWDMDA
PhiPA3_gp077      GDALGKGKNICAVCAGKALAENPNGIPAAAAGVGGRFLMIFMSKMHSSV.LRTVHWDMRR
PhiKZ_gp080       NGVIGRGKNVCAVCMGKQMSENPNGIPAAAAGVGGRFLSIFMSKMHSAV.LKTVKWSYRD
```

**RAY_gp270**	VFN
**Goslar_gp078**	YIS
**PCH45_gp003**	TAE
**201phi2-1_gp139**	RLT
**PhiPA3_gp077**	RIT
**PhiKZ_gp080**	RIS

Q

```
                  1           10         20        30        40        50
Miami_gp214       MLN....YIFGPDVKQG.PTITEINHVNFRNFNDDIQTAYGTSTPGRYMFRRVGSNKFVT
Goslar_gp160      MLDRINRLMNRITVKNQSNNSVHIGGIDAWRLSDDIQRIWGTSRFMKHMFRNFSSSGLSL
RAY_gp131         MFDYLRRALGFINVEEK.NDIITITGFNAPLATRDILKVWKTSKIAGYLFREVTQNKISF
AH06_gp136        MFDYLRRALGFINVEEK.NDVITITGFNAPLATRDILKVWKTSKVAGYLFREVTQNKISF
PhiKZ_gp203       MIDSFRKLMGSLSITET.DQEIIISGFDGAAFIRDINKYWRTTKLATQLFNTVSRRSISF
201phi2-1_gp300   MLDQFRNVFGGVTVKET.NTEIVVSGIRAKDIVRDMDKHWKTTRITQNIFNTVSGNSFSF
PhiPA3_gp233      MFDTFRTLFSGLDVKET.DTEIIISGVAANEIIRDMDKFWKTTKLTGNMFNNVSRRSMSF

                     60        70        80        90       100          110
Miami_gp214       DNFFLLELDHLITKILQSRNTWSSRKNLNELQIKLRTDTWIKDTVTPTAYPIDK...ASF
Goslar_gp160      HNFYLLDFVYILETIVEAKNTRSNKRMLKHLIEVLLQETWLQNTTIEQPAILDK...ARM
RAY_gp131         NSFFAIEVEYILKQLYEHDKTWSDRRGLGKILELLRKNTWMRNLEEQ....YEDIIDLGQ
AH06_gp136        NSFFAIEVEYIFKQLLDHDKTWTDRRGIGKILDLLRKNTWMRNLDEQ....YEDIIDLSQ
PhiKZ_gp203       YKFFAPEIYYMLEAVKNYRSRYISIKTVNAIREAMLQYTWLKNTRPVDTNSVPGRLNFKM
201phi2-1_gp300   YKFFAPEIAYILDGIKKYRHRWTSIRAINGIIEGLNEHTWLKGTIPPDTTTIKGRLDFRK
PhiPA3_gp233      YKFFAPEVMYMLENIKHYRNRWTSIKAINAIREALMEHTWLKGTIPVDPNTVPGRLDLRK

                       120       130       140       150       160
Miami_gp214       LKQFKIKPYDTQWGFLSTYKMVKKSHHLKGLLLDAKAGSGKTFTSLMWTRL.........
Goslar_gp160      NKMLTLSLFDYQDEFIDIYNRNVPKYRLKGYVCAMGAGAGKTITALA.............
RAY_gp131         LKYVKKTPLPEQRNWLNYYNTVIPRYGLRGALLSAAPGAGKTLCSIM.............
AH06_gp136        LKLVKKSPLPEQRNWLNYYNTAVPRYSLRGALLSAAPGAGKTLCSIM.............
PhiKZ_gp203       LDKLTFTPDESQKAYFENYNYRIDQYGLRGDIVAGKPGTGKTF.................
201phi2-1_gp300   LKNLHFTPKPYQMEYFKNYSYRLDQYNLKGDLIAAAAGTGKTYSM...............
PhiPA3_gp233      LQNLHFDAMTYQMEYFQNYSYRLDQYGLKGDLIAAAAGTGKAQPLTSMVKVPGGWKAMGN

Miami_gp214       ............................................................
Goslar_gp160      ............................................................
RAY_gp131         ............................................................
AH06_gp136        ............................................................
PhiKZ_gp203       ............................................................
201phi2-1_gp300   ............................................................
PhiPA3_gp233      IQVGDVVTAWDGTPTKVTGVYPQGKKQTFTVTFKDGRTTKACDEHLWNVYCQDWTRYGGT

Miami_gp214       ............................................................
Goslar_gp160      ............................................................
RAY_gp131         ............................................................
AH06_gp136        ............................................................
PhiKZ_gp203       ............................................................
201phi2-1_gp300   ............................................................
PhiPA3_gp233      GWRVINTLELFGRIQSGKQRLYVQLCKSEEGIDVELPIDPYNLGVILGDGCISSNCVSVT

Miami_gp214       ............................................................
Goslar_gp160      ............................................................
RAY_gp131         ............................................................
AH06_gp136        ............................................................
PhiKZ_gp203       ............................................................
201phi2-1_gp300   ............................................................
PhiPA3_gp233      SGDPQLFTEFAKALPENLELITRDDITMGVINKKGERNPYTSALREMGLLGENSLTKFIP

Miami_gp214       ............................................................
Goslar_gp160      ............................................................
RAY_gp131         ............................................................
AH06_gp136        ............................................................
PhiKZ_gp203       ............................................................
201phi2-1_gp300   ............................................................
PhiPA3_gp233      QNYLMASTAQRLALVQGLMDTDGTVDVNSSLSFSTSSYMLAKQFQYLIRSLGGIAKISFK
```

```
Miami_gp214        ............................................................
Goslar_gp160       ............................................................
RAY_gp131          ............................................................
AH06_gp136         ............................................................
PhiKZ_gp203        ............................................................
201phi2-1_gp300    ............................................................
PhiPA3_gp233       EPTYTYNGVKQYGNMSYRVLVRFDVPSALFRLDRKLARCNDNHQYTENLRLQIKHVNVSD

                                                             170       180
Miami_gp214        ....................................TGNKKHIIICPDAGINTVWKLHME
Goslar_gp160       ................................LSAAMNVDVFVVCCPKNTMRSAWQSDVN
RAY_gp131          ................................TMLCRKKDYVIVIAPKKATRDVWERTIT
AH06_gp136         ................................TMLCRKKDFVIVIAPKKATRDVWERTIT
PhiKZ_gp203        ............................MTMAIAEMVESDIIIVVCEKKSIDLIWKPSII
201phi2-1_gp300    ..............................SALAEMLGAELIVVFCPKAVLESVWVESVN
PhiPA3_gp233       RVECQCIQVEHQDHLYVTDDFIVTHNTYMTSAIAEMRSAELIVVICPKQALETVWLESIP

                     190         200        210       220       230       240
Miami_gp214        EKV..FVDPPKYWSTRQNK.PFDPSCEYFLIHIDYTRNPDFFKILDQVFEAAGKGGVSLV
Goslar_gp160       KAIVDLATETAFWSDTMPPSQLQKSDKYYFLHYES..MGLLNDVMIKH..KRTAKRVLIV
RAY_gp131          TE...LTTEESVWVAEYDQ.PYRKGTKWIVAHYER..LDEVVKMVKELR.L...PNVGII
AH06_gp136         TE...LTTEQDVWVAEYGQ.PYRKGTKWIVAHYEK..LDEIVKLVKEHR.L...PNVGII
PhiKZ_gp203        EM...YKERQKVWSTID.D.KAYNGQRILISHYQA..QDKIIDLLRS.G.IFKGKNITVI
201phi2-1_gp300    EM...FKSPQSIWHSGE.P.MAYEGQRWIMCHYDA..MSKLQELFQDPS.VYQGKKIVTI
PhiPA3_gp233       DM...FKAKQSVWSSANKH.APYKGERWIVCHYDA..MDRLIELLRDQR.VYKGKRVVTI

                        250       260              270       280       290
Miami_gp214        VDESHNFNELSSKQTQN.....IIKA..ADHYPFNDALPMSGTPLKAMAREAYSIFALID
Goslar_gp160       VDESHNFNDK..NSQRSQRLVDLVQTMRQTMNADVHVLFMSGTPVKQMGSEMIPCLGCID
RAY_gp131          LDESHNLNQKTKESIRTNLFVELCQA.....SGSQDIVWASGTALTAMGTEAVPLFRTLI
AH06_gp136         LDESHNLNQKTKESTRTGLFVELCQV.....SGAQDIVWASGTALTAMGTEAVPLFRTLI
PhiKZ_gp203        LDESHNMNDP..NSAQSLKFQQICFM.....SNSNNRLLASGTPVKALGSELVSALRVLD
201phi2-1_gp300    LDESHNMNDP..NSARSMLYQSLVKL.....LDSENNIQGSGTPVKALGAEIITLLRVVD
PhiPA3_gp233       LDESHNLNDP..NSARSLYYQTICKI.....LGSEDNLFASGTPVKALGSELIGLLRVVD

                   300       310       320       330        340        350
Miami_gp214        PFFKGNARERFMKSYGLSRDKLNTLLAHRLGRTRYVIDSL.FDMGE.EPPVEMVPVTVPN
Goslar_gp160       PMFQGQVVDSFKAIYGLTSSRANDILRHRLGFMMHVVPKEAY..RKTRPEVQDVYVKLPN
RAY_gp131          PGFTNDVELAMRKIWGKTATKANDILANRLGIVSFSVPKSKF..MTTKPIEMTVKIKIKN
AH06_gp136         PGFTADVELAMRKIWGKTATKANDILANRLGIVSFSVPKSKF..MSTKPIEATVKVKIKN
PhiKZ_gp203        DLFTNEVEERFKKAFGKETQKGLDIVQHRFGLIAYVIEKKD..TEVLPPRIKAYRIKIPN
201phi2-1_gp300    PLFTPAVEAKFKKMYGKEASKGLDIIRHRLGLVSYKVEKSEADESLLPPIMRPYPIKVPD
PhiPA3_gp233       DMFTPAVEERFRKMYGKEASKGLDIIRHRLGLMSFKVEKKE..TGLQPPIMKPYPIKIPN

                     360       370       380       390       400       410
Miami_gp214        GERFTLKAIRLEMLSYIQNRVKFYEQNMPMFLAFFNHVLDDYESYVKDDNGKLGELVKYK
Goslar_gp160       GSDYTISSIRDDMAIFVRDRAKYYKDNWGHYRKIFDDALDYYRKTIRTDG.ERKELSRYE
RAY_gp131          GAHYTLDNIQRIMMAFIQERFKFYTDNKPAYQKIYDDALGWYEKTLHSPK.EKEDFKLYN
AH06_gp136         GAHYTLDNIQRIMMAFIQERFKFYTENKATYQKIYDDALAWYEKTLHSPK.EKEDFKKYN
PhiKZ_gp203        GSQYTLNAIKRDCEAFIRERVKFYAARRPEDEKYWAKLMAIHENSLKTQA.QKDGFARYK
201phi2-1_gp300    GERFTLPAIRKMMEAFIKERTAYYQKRRDTDMRKWENFIKIARSKLKDRQ.ALIQFDEYL
PhiPA3_gp233       GELFTLPMIKKDMEKFINERWAYYKSRRPNDEKLWNQMLDIHRKSLKTRD.QVKAFEDYL

                     420       430       440        450       460       470
Miami_gp214        QIVNRFRTHGYNNFTDSADSQYAKNVEI.DIEARLRGEQLKQFRNIKSAVKYVGLKIRGE
Goslar_gp160       SIVA.RFQKGYDPVRDKEDAMFCNVFEKRAIMPVLPSDLRVAFKDAKSVIKYVGLKIQGE
RAY_gp131          QYVNTFVTQGFDPYSMAYMSQYCNEYELKRIIPVIPDHMRKTFKDARSVIKYVDLKIKGE
AH06_gp136         DYVEMFIKQGYQAEWMGPYSQYCNDYELKRIIPVIPDHMRKPFKDARSVIKYVDLKIKGE
PhiKZ_gp203        NLIK.IIQKNQDPRYIGEEIKESNQYEKMFIEPTLPRNEIAAFRDIKSVIKYVMLKIQGE
201phi2-1_gp300    HTVH.LISRTPDPRYLGDEMKRANAYEKDIFEKLLPQTMVKDFRDIKSVVKYVNLKIQGE
PhiPA3_gp233       RLVE.LVKRTPDPRYIGEEIKATNVYEKRIFEPTLPRTMIHQFRDIKSVIKYTNLKIQGE
```

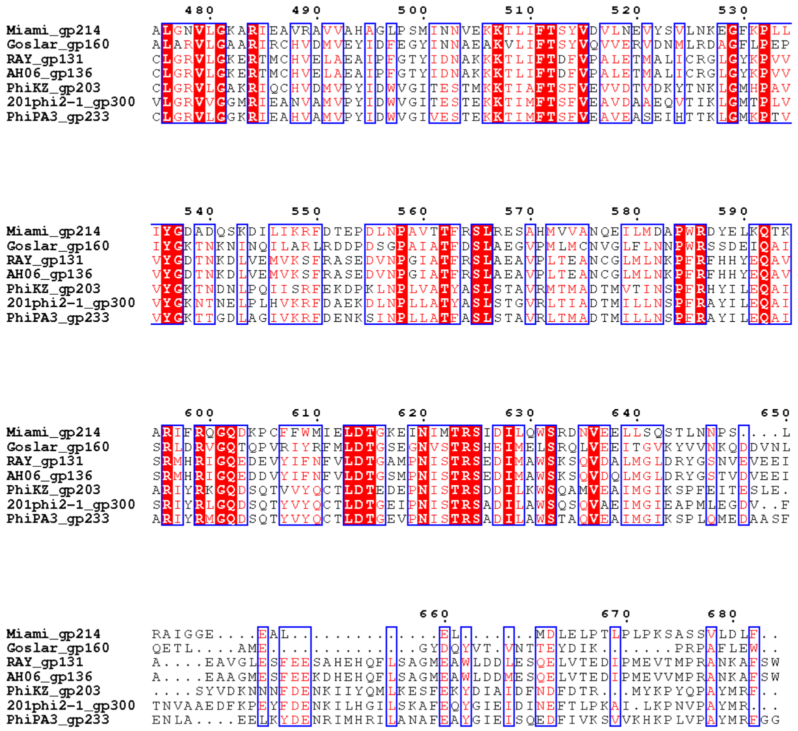
480 490 500 510 520 530
Miami_gp214 ALGNVLGKARIEAVRAVVAHAGLPSMINNVEKKTLIFTSYVDVLNEVYSVLNKEGFKPLL
Goslar_gp160 ALARVLGAARIRCHVDMVEYIDFEGYINNAEAKVLIFTSYVQVVERVDNMLRDAGFLPEP
RAY_gp131 CLGRVLGKERTMCHVELAEAIPFGTYIDNAKKKTLIFTDFVPALETMALICRGLGYKPVV
AH06_gp136 CLGRVLGKERTMCHVELAEAIPFGTYIDNAKKKTLIFTDFVPALETMALICRGLGYKPVV
PhiKZ_gp203 CLGRVLGAKRIQCHVDMVPYIDWVGITESTMKKTIAFTSFVEVVDTVDKYTNKLGMHPAV
201phi2-1_gp300 VLGRVVGGMRIEANVAMVPYIDWVGITESTEKKTIMFTSFVEAVDAAEQVTIKLGMTPLV
PhiPA3_gp233 CLGRVLGGKRIEAHVAMVPYIDWVGIVESTEKKTIMFTSFVEAVEASEIHTTKLGMKPTV
540 550 560 570 580 590
Miami_gp214 IYGDADQSKDILIKRFDTEPDLNPAVTTFRSLRESAHMVVANQEILMDAPWRDYELKQTK
Goslar_gp160 IYGKTNKNINQILARLRDDPDSGPAIATFDSLAEGVPMLMCNVGLFLNNPWRSSDEIQAI
RAY_gp131 VYGDTNKDLVEMVKSFRASEDVNPGIATFRSLAEAVPLTEANCGLMLNKPFRFHHYEQAV
AH06_gp136 VYGDTNKDLVEMVKSFRASEDVNPGIATFRSLAEAVPLTEANCGLMLNKPFRFHHYEQAV
PhiKZ_gp203 VYGKTNDNLPQIISRFEKDPKLNPLVATYASLSTAVRMTMADTMVTINSPFRHYILEQAI
201phi2-1_gp300 VYGKNTNELPLHVKRFDAEKDLNPLLATYASLSTGVRLTIADTMILLNSPFRAYILEQAI
PhiPA3_gp233 VYGKTTGDLAGIVKRFDENKSINPLLATFASLSTAVRLTMADTMILLNSPFRAYILEQAI
600 610 620 630 640 650
Miami_gp214 ARIFRQGQDKPCFFWMIELDTGKEINIMTRSIDILQWSRDNVEELLSQSTLNNPS....L
Goslar_gp160 SRLDRVGQTQPVRIYRFMLDTGSEGNVSTRSHEIMELSRQLVEEITGVKYVVNKQDDVNL
RAY_gp131 SRMHRIGQEDEVYIFNFVLDTGAMPNISTRSEDIMAWSKSQVDALMGLDRYGSNVEVEEI
AH06_gp136 SRMHRIGQEDDVYIFNFVLDTGSMPNISTRSEDIMAWSKSQVDQLMGLDRYGSTVDVEEI
PhiKZ_gp203 ARIYRKGQDSQTVVYQCTLDTEDEPNISTRSDDILKWSQAMVEAIMGIKSPFEITESLE.
201phi2-1_gp300 SRIYRLGQDSQTYVYQCTLDTGEIPNISTRSADILAWSQSQVAEIMGIEAPMLEGDV..F
PhiPA3_gp233 ARIYRMGQDSQTYVYQCTLDTGEVPNISTRSADILAWSTAQVEAIMGIKSPLQMEDAASF
660 670 680
Miami_gp214 RAIGGE....EAL..............EL.......MDLELPTLPLPKSASSVLDLF..
Goslar_gp160 QETL....AME..............GYDQYVT.VNTTEYDIK.......PRPAFLEW..
RAY_gp131 A....EAVGLESFEESAHEHQFLSAGMEAWLDDLESQELVTEDIPMEVTMPRANKAFSW
AH06_gp136 A....EAAGMESFEEKDHEHQFLSAGMEAWLDDMESQELVTEDIPMEVVMPRANKAFSW
PhiKZ_gp203 ....SYVDKNNNFDENKIIYQMLKESFEKYDIAIDFNDFDTR...MYKPYQPAYMRF..
201phi2-1_gp300 TNVAAEDFKPEYFDENKILHGILSKAFEQYGIEIDINEFTLPKAI.LKPNVPAYMR...
PhiPA3_gp233 ENLA....EELKYDENRIMHRILANAFEAYGIEISQEDFIVKSVVKHKPLVPAYMRFGG

R

```
               1        10        20        30        40        50        60
AH06_gp315     MTTMSELGPAHTTLLAEEKDVQKDAATDATETALAVLKKTSKSKPEKDDEDEPTISFGAE
RAY_gp299      MSTMTELGPSQGTLLADEKAVHDDAATEATEVALHVLKKIGKKKEKDDEDEEVVLSFGSE
Miami_gp025    ....................................................MSTPYTQK

                        70                 80        90       100       110
AH06_gp315     GYTMLTSMSFDLTE.NTDLA......IQELLDKISKIVIEANGNLTDKDLEAIKN.VGNE
RAY_gp299      GYAMLTSMSFDLTE.NSDLA......VQELLDKITTIVIEANGNLTQKDLEKIKS.VGNE
Miami_gp025    GMSMSLAEEYEGDKKKLDRYIEGTPDNVERYKKVAAVIGKEPGHVTDDK.DTIDNTAGTE

                                  120       130       140       150
AH06_gp315     .............ALLDAGNEDYKEVMNLVMRKIMQFIRWAINQGKEGIKRISDRLSRLS
RAY_gp299      .............SLLGDGNEDYKEVVTMVMKRIMQFIRWAINQGKEGIKRVADRLSRLS
Miami_gp025    EFDIDEAVLNFPEDENVAGMEDFVEFAKKAIVRLMRMIKNAAIFIYELVFNKVSRIKHIA

             160       170       180       190       200       210
AH06_gp315     VKAMYVERKIDVSADNSLPNDKFVLPRTYPLLMMANKPPANAMEVINVLNRTKYLFTTLH
RAY_gp299      VKTMYVERKIDISTDNSLPTDKFVLPRSYPMLMMVNKPPANAMEVINAVNRTKYLFTTLH
Miami_gp025    RKAEYQLRT.....QSLIPT.LVPYPKGTRKIFIGPALQTNANWTLKSIQQVQLFYTRLM

             220       230       240       250       260       270
AH06_gp315     NDYQNFQNLFKSAVATGSRADTLEMINSYLTSLASKLSARPNPLFDNRLSF.........
RAY_gp299      NDYQGFQALFKAAVATGSRADTLEMINNYLNGLISKLSARPNPLFDNRNTF.........
Miami_gp025    KAHGN...LLDAA.KSG..................NWRNNPNFIFETAATFANEICQNHH

                                280       290       300       310       320
AH06_gp315     ......NQLPGG..YRLVFSQGQAFADCSATLTRTSERYDMAPAVQRPDKASLMRLVAEV
RAY_gp299      ......NQLPGG..YRLVFSEGQSFADCGATILRTPEKYEAAPAVQRPDKASLVRLVSEI
Miami_gp025    SDYSETQVLPGGKIYRVNYSKGSGKNRPGVSLSQKL.SVQLKDKSFKPDRAIADDLMTAL

                      330       340       350       360       370
AH06_gp315     KTYLRIINEVY...GRVSGRLEADFRAIVKSAEREVKS.FDSTADIRTASTTIEWFTEQQ
RAY_gp299      KTYLRVINEVY...GKVSSRLEVDFRNIVKAAEKEVKG.FDSTADIRTASTTIEWFTEQQ
Miami_gp025    QGFADKIGNDHRSQTSTSRKMEAAIR........DLERYYVDPEAKRHAAAYFTWLIDFQ

              380       390       400       410       420       430
AH06_gp315     SKLYTRSMMLSCTVLNASLDYCLSAIGAKPAAGTESFDITETSSISYAIESLDEQLERLD
RAY_gp299      SRLYTRSMMLSCTVLNAALDYCLGAIGAKPAVGTESFDILDTSSMGYAIESLGEQMERLD
Miami_gp025    R...........TVLSTGLQYVFGTLFGYAEFISANVK......................

              440       450       460       470       480       490
AH06_gp315     AGLCSLEIDSRSMQSIADVEELVDVDNDQVINLLMNQTPASYYNPLYGISFAGLQGVLEE
RAY_gp299      AGLCELEIDARTMQSITDVKEMVDVDNDHVIQLLMQQRPSSYFNPPDGLSEYSLGDAFNG
Miami_gp025    ............................................................

              500       510       520       530       540           550
AH06_gp315     KKGAEYIASRLGGIVDITKQLDNTAEVIKQIMTDLPDGEVGAVKPGLK......PTVADV
RAY_gp299      SATARYIGRRLTSIVNLTNQLSGTTDLMKGLLAEMPTGEIK...PGSDADVIERNSLLDR
Miami_gp025    ............................................................

                    560       570       580       590       600       610
AH06_gp315     EFTSDHPWCAFLHRVDRQSIYASDVVRYVGDTYEKLRGVNNTLSVLAEQMTKTVVDGEMN
RAY_gp299      EMFEGHPLCAFLHRVDREGIYATDVIRYVTDTYEKLRQVRTTLAVQAEQMTLTVADGEMN
Miami_gp025    ............................................................
```

```
                   620       630       640       650       660       670
                     .         .         .         .         .         .
AH06_gp315    VSLLKEYMFNAPTEPFTASSLCGGFTISPVAEKLGKLVVRGYVLESPPPLKPMVTIHRPS
RAY_gp299     VSLLKEFLFNAPQEPFTDSVLCGGFRLQPVAEKLGELVIHGAVMDSPPPLKPMTTIHRPS
Miami_gp025   ............................................................

                   680       690       700       710       720       730
                     .         .         .         .         .         .
AH06_gp315    YDEYNEINSLAARFNAEQARLAEITRLLQIGTGHLRYITASVADNLTADGLKGGGDGWFS
RAY_gp299     YDEYNGVNQLASLFGNELRELAKIAERLQIGTGYLRYITTSVADNLNEDGLKGGGDGWFN
Miami_gp025   ............................................................

                   740       750       760       770       780
                     .         .         .         .         .
AH06_gp315    TALEYLAISARQYRWMYRLTVQLAIYQRTMISALRQYNNEGGWDGDKQ
RAY_gp299     TALEYLAVSARQYRWMYRLTVQLAVYEHTMINALRHYNNSGGWHD...
Miami_gp025   ................................................
```

Figure S4. Multiple sequence alignments, Related to Figures 3, 4, and 5. (A) Multiple sequence alignment of RAY gp222 with chimallin from previously published nucleus-forming phages. (B) Multiple sequence alignment of RAY gp220 and DNA polymerases from previously published nucleus-forming phages. (C) Multiple sequence alignment of RAY gp315 with DNA helicases from previously published nucleus-forming phages. (D) Multiple sequence alignments of RAY gp002 (i), gp248 (ii), gp223 (iii), and gp249 (iv) with nvRNAP subunits from previously published nucleus-forming phages. These RAY proteins are homologs of known phage msRNAP subunits ΦKZ gp123, gp71-73, gp55-56.1, and gp74, respectively [88]. (E) Multiple sequence alignment of RAY gp150 with UvsX from previously published nucleus-forming phages. UvsX is a RecA homolog. (F) Multiple sequence alignment of RAY gp153 with DprA from previously published nucleus-forming phage Goslar and RAY close relative AH06. DprA is a RecA-associated protein involved in recombination. Since it is not part of the core genome, it was not present in most of the previously-studied nucleus-forming phages used for other MSA analysis. (G) Multiple sequence alignment of RAY gp250 with SF2 helicase homologs from previously published nucleus-forming phages. RAY gp250 localized in the phage nucleus (Fig. 4B) as opposed to RAY gp131, another SF2 helicase homolog which localized similarly to capsids (Fig. 5B), leading us to call gp250 and its homologs the “non-virion” SF2 helicase. (H) Multiple sequence alignment of RAY gp116 with HslUV-like protease homologs from RAY’s close relative AH06 and its distant relative Miami. HslUV is not part of the core genome and was not present in any of the previously-studied nucleus-forming phages used for other MSA analysis. (I) Multiple sequence alignment of RAY gp311 with TMK from previously published nucleus-forming phages. (J) Multiple sequence alignment of RAY gp039 with SspB from previously published nucleus-forming phages and RAY close relative AH06. Since SspB is not part of the core genome, it was not present in some of the previously-studied nucleus-forming phages used for other MSA analysis. (K) Multiple sequence alignment of RAY gp048 with RtcB from previously RAY close relative AH06 and ΦPA3 close relative *Pseudomonas* phage PA1C. RtcB is not part of the core genome and was not present in any of the previously-studied nucleus-forming phages used for other MSA analysis. However, despite its uncommon occurrence in Chimalliviridae accessory genomes, its amino acid sequence conservation is remarkably high, even between distantly-related phages like RAY and PA1C. (L) Multiple sequence alignment of RAY gp064 with related exonucleases from previously published nucleus-forming phage Goslar and RAY close relative AH06. Since this exonuclease is not part of the core genome, it was not present in most of the previously-studied nucleus-forming

phages used for other MSA analysis. (M) Multiple sequence alignment of RAY gp094 with XRE superfamily transcriptional repressors from previously published nucleus-forming phage PCH45, RAY close relative AH06, and RAY distant relative Miami. Since this transcriptional regulator is not part of the core genome, it was not present in most of the previously-studied nucleus-forming phages used for other MSA analysis. (N) Multiple sequence alignment of RAY gp179 with tail sheath proteins from previously published nucleus-forming phages. (O) Multiple sequence alignment of RAY gp317 with MCP from previously published nucleus-forming phages. (P) Multiple sequence alignments of RAY gp164 (i), gp154 (ii), gp163 (iii), and gp270 (iv) with vRNAP subunits from previously published nucleus-forming phages. These RAY proteins are homologs of known phage msRNAP subunits ΦKZ gp178, gp149, gp180, and gp80, respectively [17]. (Q) Multiple sequence alignment of RAY gp131 with SF2 helicase homologs from previously published nucleus-forming phages, as well as RAY close relative AH06 and RAY distant relative Miami. RAY gp131 localized similarly to capsids (Fig. 5B) and was seen in virion mass spec experiments [16] as opposed to RAY gp250, another SF2 helicase homolog which localized in the phage nucleus (Fig. 4B), leading us to call gp131 and its homologs the "virion" SF2 helicase. While this virion SF2 helicase is not part of the core genome (and not found in PCH45), it is widely conserved in a majority of Chimalliviridae. (R) Multiple sequence alignment of RAY gp299 with homologs from RAY's close relative AH06 and its distant relative Miami. This protein is found in capsids [16] but is not part of the core genome and was not present in any of the previously-studied nucleus-forming phages used for other MSA analysis. As there is very little information about this protein, we are not able to make any hypotheses concerning its potential function, and it remains an area of future study.

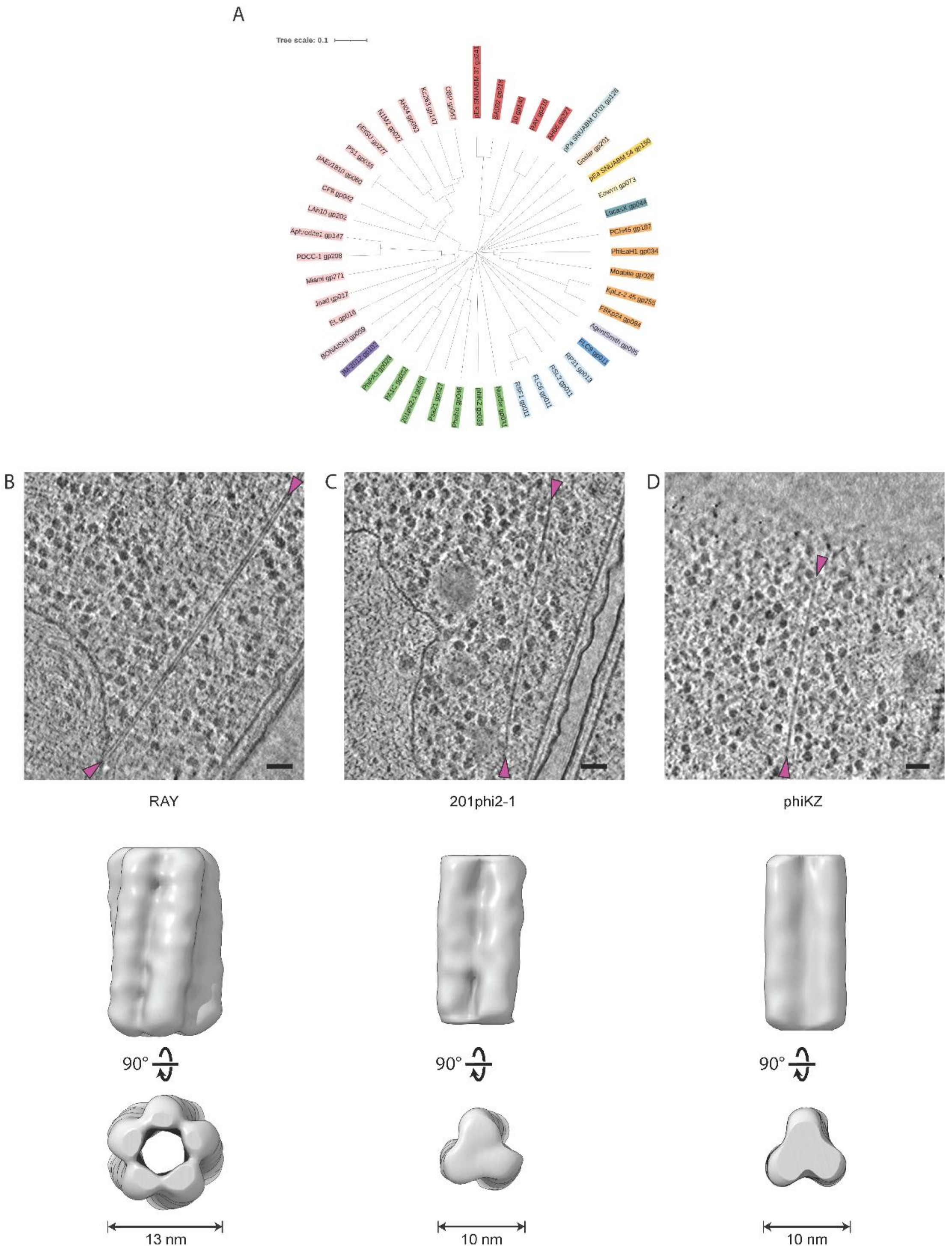

Figure S5. PhuZ protein phylogeny and filament structural comparison, Related to Figure 6. (A) A protein tree of PhuZ homologs from Chimalliviridae. While PhuZ is not a member of the core

genome, it is present in a majority of Chimalliviridae. Chimalliviridae are color-coded by predicted genus as in Figure 1. (B,C,D) Top, tomographic slices from jumbo phage infected host cells with the ends of putative PhuZ filaments marked with magenta arrows. Bottom, orthogonal views of subtomogram averages from the corresponding tomographic datasets for (B) RAY, (C) 201ϕ2-1 (EMPIAR-10859), and (D) ΦKZ. Scale bars: 50 nm.

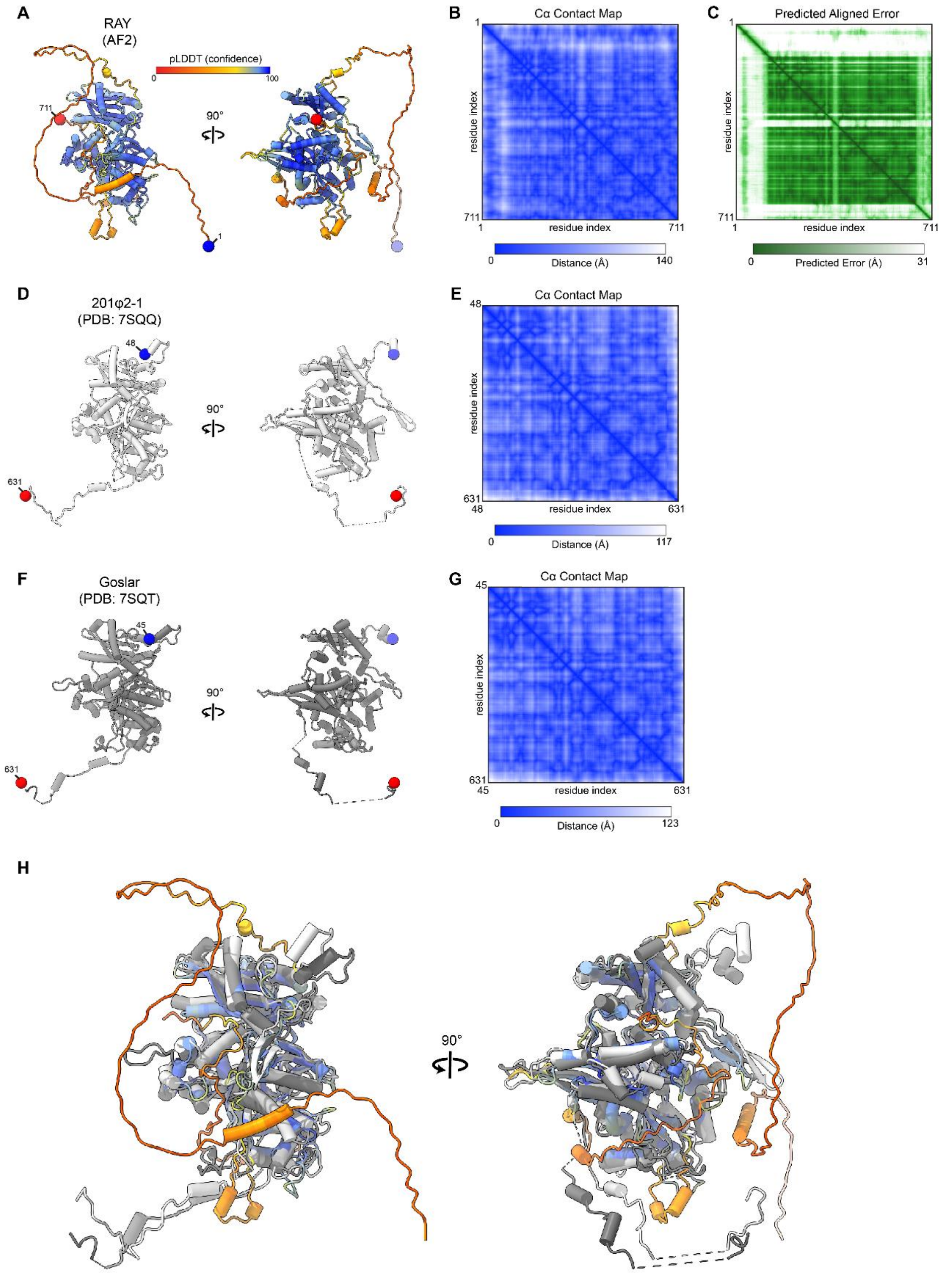

Figure S6. Structural comparison of RAY, 201ϕ2-1, and Goslar chimallin protomers, Related to Figure 7. (A) Orthogonal views of the AF2 predicted RAY chimallin protomer model colored by pLDDT. N- and C-termini are labeled and shown as blue and red spheres, respectively. (B) Pairwise C-alpha distance map and (C) predicted aligned error plot for the RAY chimallin protomer model. (D)

Orthogonal views of the 201ϕ2-1 chimallin protomer model (PDB ID: 7SQQ) colored white with termini indicated as in A and (E) corresponding pairwise C-alpha distance map. (F) Orthogonal views of the Goslar chimallin protomer model (PDB ID: 7SQT) colored gray with termini indicated as in A and (G) corresponding pairwise C-alpha distance map. (H) Orthogonal views of superimposed chimallin protomer models from A,D, and F.

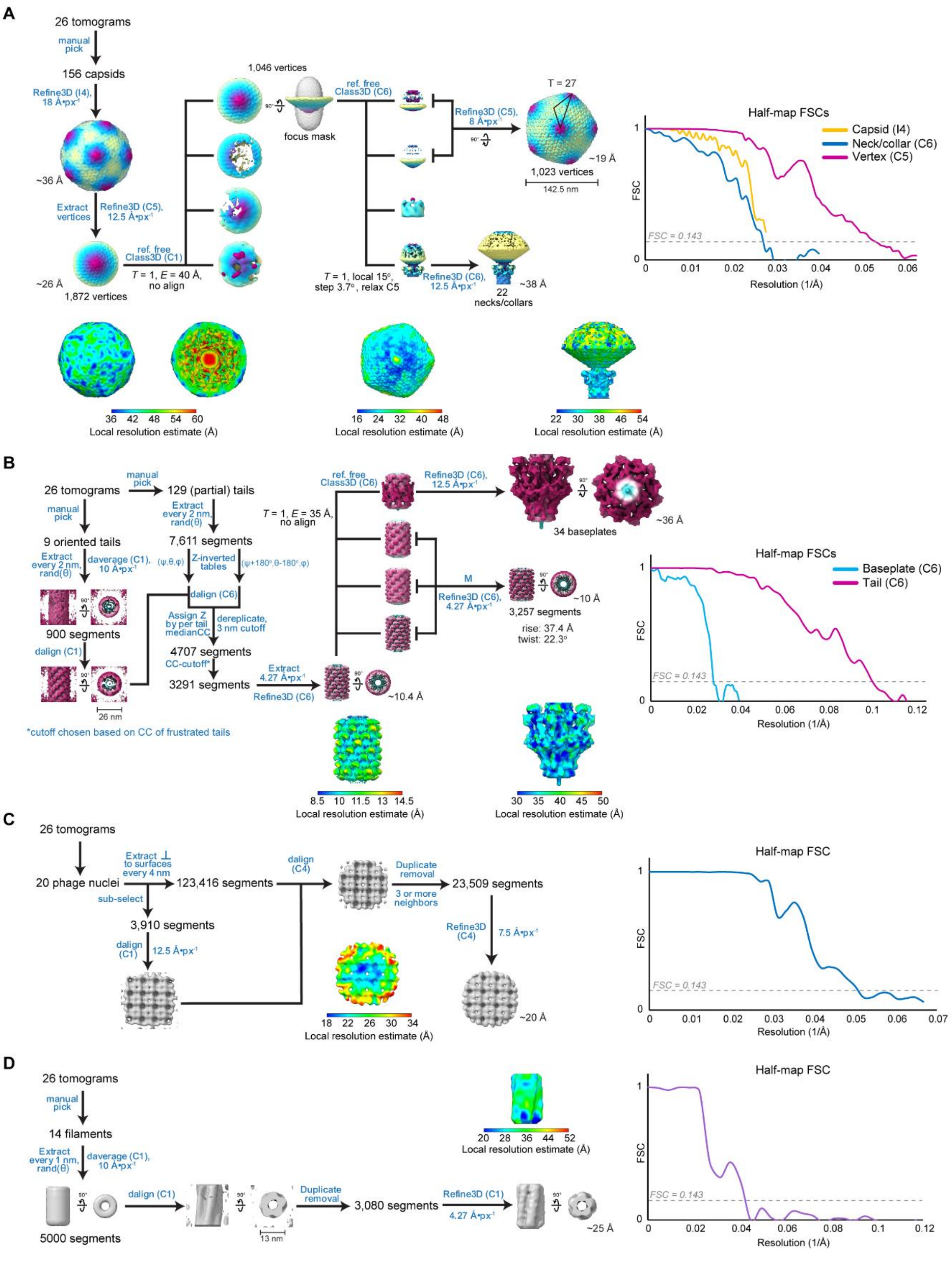

A
26 tomograms
manual pick
156 capsids
Refine3D (I4), 18 Å•px⁻¹
~36 Å
Extract vertices
Refine3D (C5), 12.5 Å•px⁻¹
~26 Å
1,872 vertices
ref. free Class3D (C1)
T = 1, E = 40 Å, no align
1,046 vertices
focus mask
ref. free Class3D (C6)
T = 1, local 15°, step 3.7°, relax C5
Refine3D (C5), 8 Å•px⁻¹
T = 27
~19 Å
1,023 vertices
142.5 nm
Refine3D (C6), 12.5 Å•px⁻¹
~38 Å
22 necks/collars
Half-map FSCs
Capsid (I4)
Neck/collar (C6)
Vertex (C5)
FSC = 0.143
FSC
Resolution (1/Å)
Local resolution estimate (Å)
B
26 tomograms
manual pick
9 oriented tails
Extract every 2 nm, rand(θ)
daverage (C1), 10 Å•px⁻¹
900 segments
dalign (C1)
26 nm
129 (partial) tails
Extract every 2 nm, rand(θ)
7,611 segments
(ψ,θ,φ)
Z-inverted tables
(ψ+180°,θ-180°,φ)
dalign (C6)
Assign Z by per tail medianCC
dereplicate, 3 nm cutoff
4707 segments
CC-cutoff*
3291 segments
Extract 4.27 Å•px⁻¹
Refine3D (C6)
~10.4 Å
T = 1, E = 35 Å, no align
ref. free Class3D (C6)
Refine3D (C6), 12.5 Å•px⁻¹
34 baseplates
~36 Å
M
Refine3D (C6), 4.27 Å•px⁻¹
3,257 segments
~10 Å
rise: 37.4 Å
twist: 22.3°
*cutoff chosen based on CC of frustrated tails
Half-map FSCs
Baseplate (C6)
Tail (C6)
FSC = 0.143
Resolution (1/Å)
Local resolution estimate (Å)
C
26 tomograms
20 phage nuclei
Extract ⊥ to surfaces every 4 nm
123,416 segments
sub-select
3,910 segments
dalign (C1)
12.5 Å•px⁻¹
dalign (C4)
Duplicate removal
3 or more neighbors
23,509 segments
Refine3D (C4)
7.5 Å•px⁻¹
~20 Å
Local resolution estimate (Å)
Half-map FSC
FSC = 0.143
Resolution (1/Å)
D
26 tomograms
manual pick
14 filaments
Extract every 1 nm, rand(θ)
daverage (C1), 10 Å•px⁻¹
5000 segments
dalign (C1)
13 nm
Duplicate removal
3,080 segments
Refine3D (C1)
4.27 Å•px⁻¹
~25 Å
Local resolution estimate (Å)
Half-map FSC
FSC = 0.143
Resolution (1/Å)

Figure S7. Subtomogram analysis workflows of RAY components, Related to Figure 7. Workflow schematics, local resolution estimates, and half-map Fourier shell correlation curves for the RAY (A) capsid and collar, (B) tail sheath and baseplate, (C) chimallin, (D) putative PhuZ filament.

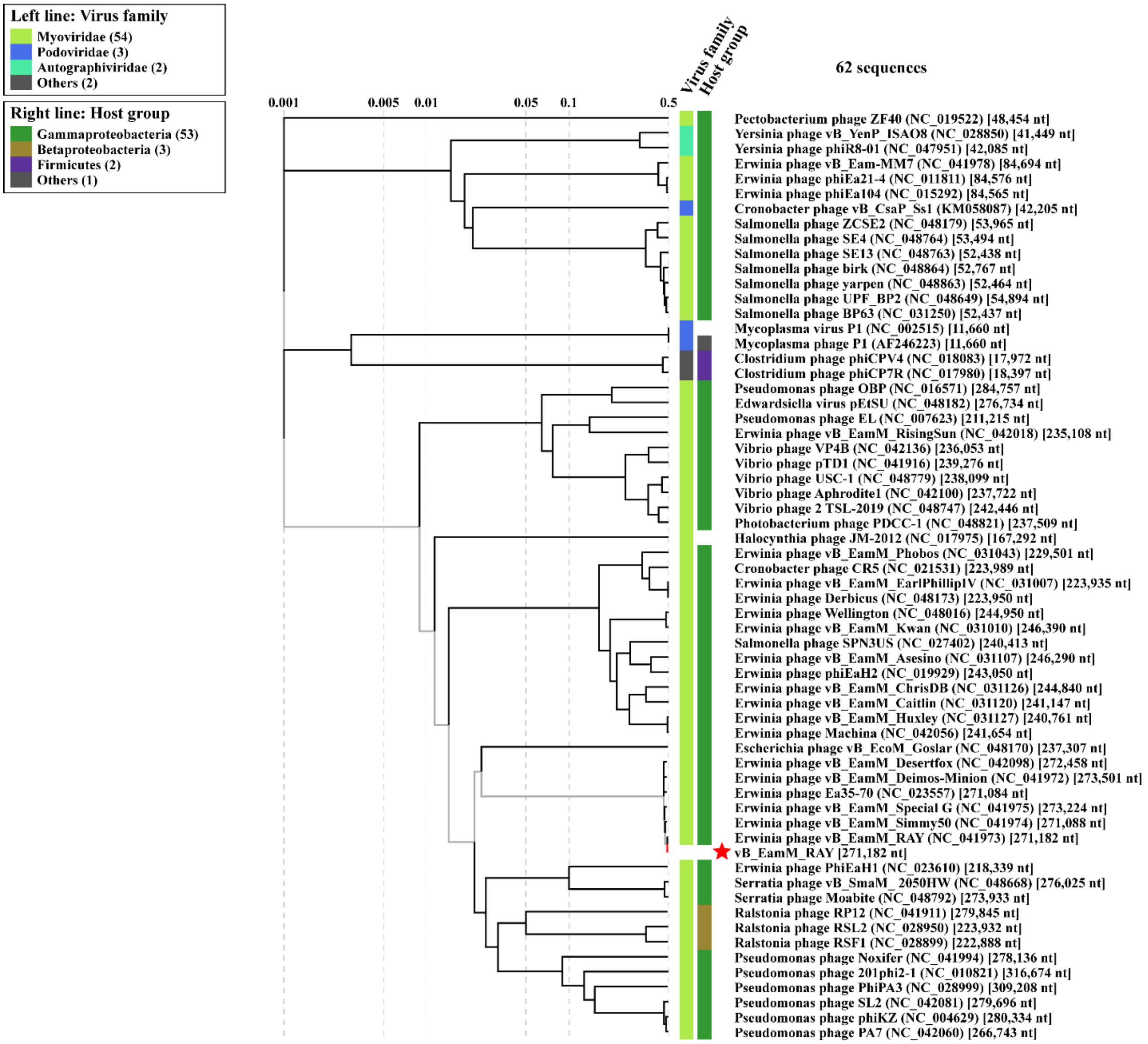

Figure S8. ViPTree-predicted relatives of RAY, related to STAR Methods. Relatives of RAY were attempted to be found using ViPTree to identify similar genomes. This whole genome tree represents possible RAY relatives found by ViPTree[64].
